# Insights into the Friedel–Crafts Benzoylation of *N*-Methylpyrrole inside the Confined Space of the Self-Assembled Resorcinarene Capsule

**DOI:** 10.1021/acs.orglett.3c01935

**Published:** 2023-08-29

**Authors:** Veronica Iuliano, Carmen Talotta, Margherita De Rosa, Annunziata Soriente, Placido Neri, Antonio Rescifina, Giuseppe Floresta, Carmine Gaeta

**Affiliations:** † Laboratory of Supramolecular Chemistry, Dipartimento di Chimica e Biologia “A. Zambelli”, Università di Salerno, Via Giovanni Paolo II 132, I-84084 Fisciano, Salerno, Italy; ‡ Dipartimento di Scienze del Farmaco, Università di Catania, Viale Andrea Doria 6, 95125 Catania, Italy

## Abstract

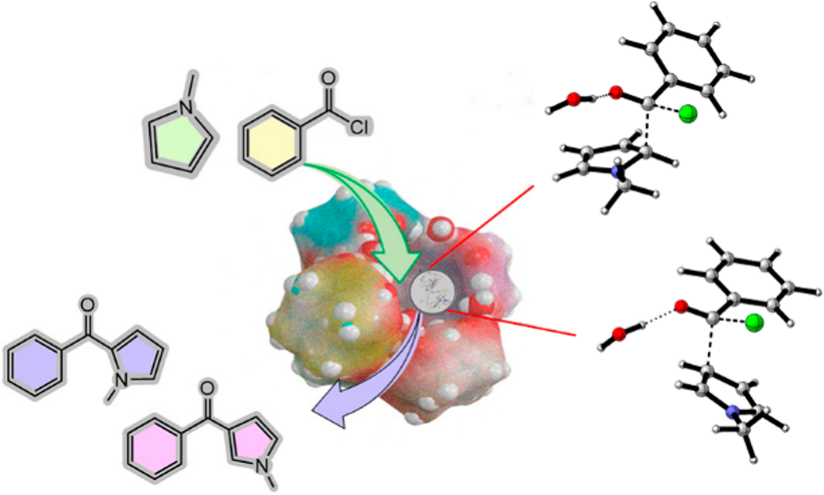

Friedel–Crafts benzoylation of *N*-methylpyrrole **2** can run inside the confined space of the hexameric resorcinarene capsule **C**. The bridged water molecules at the corner of **C** act as H-bonding donor groups to polarize the C–Cl bond of benzoyl chlorides **3a**–**f**. Confinement effects on the regiochemistry of the FC benzoylation of *N*-methylpyrrole are observed. The nature of the *para*-substituents of **3a**–**f** and their ability to establish H-bonds with the water molecules of **C** work synergistically with the steric constrictions imposed by the capsule to drive the regiochemistry of products **4a**–**f**. QM investigations indicate that inside the cavity of **C**, the FC benzoylation of **2** has a bimolecular concerted S_N_2 mechanism, appropriately, above-plane nucleophilic vinylic substitution (S_N_Vπ)—supported by H-bonding interactions between water molecules and both the leaving Cl atom and the carbonyl group.

In the last decades, the Friedel–Crafts (FC) acylation of pyrroles has been widely investigated, enabling the synthesis of active pharmaceutical compounds and fine chemicals.^1^ As known, metal-catalyzed Friedel–Crafts acylation of pyrroles is considered poorly sustainable, and many efforts have been focused on studying greener FC strategies.^2^ In this regard, Aubé and colleagues reported examples of FC acylation promoted by hexafluoro-2-propanol (HFIP), which acts as a strong hydrogen bond donor to activate the C–Cl bond.^3^

In 2018,^4^ we reported an organocatalytic example of FC benzylation of *N*-methylpyrrole by exploiting the confined space of the hexameric resorcinarene^5^ capsule **C** (Figure 1). The supramolecular capsule **C**(5) is formed by self-assembling 6 resorcinarene macrocycles **1**(6) and 8 waters, sealed by 60 H-bonding interactions (Figure 1).^5a^ The capsule **C** shows a π-electron-rich cavity of 1375 Å^3^. The H-bonding donor abilities of the bridging water molecules (green in Figure 1) were exploited to polarize the C–Cl bond of the benzyl chloride^4^ hosted inside the capsule. In the confined space, the molecular motions are slowed down;^7^ consequently, more compact transition states are formed in which the collisional orientation of reagents may differ with respect to the bulk medium.^8^

**Figure 1:**
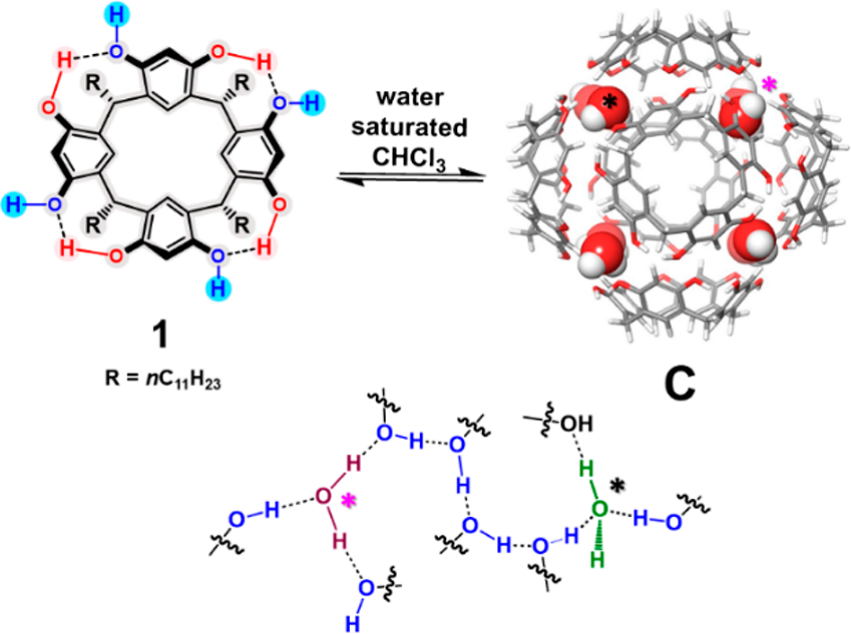
(Top) Self-assembly of **C**. (Down) Detailed H-bonding network among bridged water molecules and resorcinarene–OH groups in **C**.

Consequently, the regio- and stereochemistry of reactions inside the confined space can diverge from the analogous reactions in bulk medium.^8,9^

Concerning the FC benzylation of the *N*-methylpyrrole reported by us,^4^ the confinement of reagents inside the cavity of **C** led to uncommon regiochemistry, in which the β-regioisomer was preferentially obtained.

Now, the question arises of whether the hexameric capsule **C** can act as an organocatalyst for the FC benzoylation of *N*-methylpyrrole. Can the C–Cl bond polarization result from its H-bonding interaction with the bridged water molecules (green in Figure 1)? What happens to the regiochemistry of the reactions in Scheme 1 when reactants **2** and **3** are confined in the restricted space inside **C**?

**Scheme 1:**
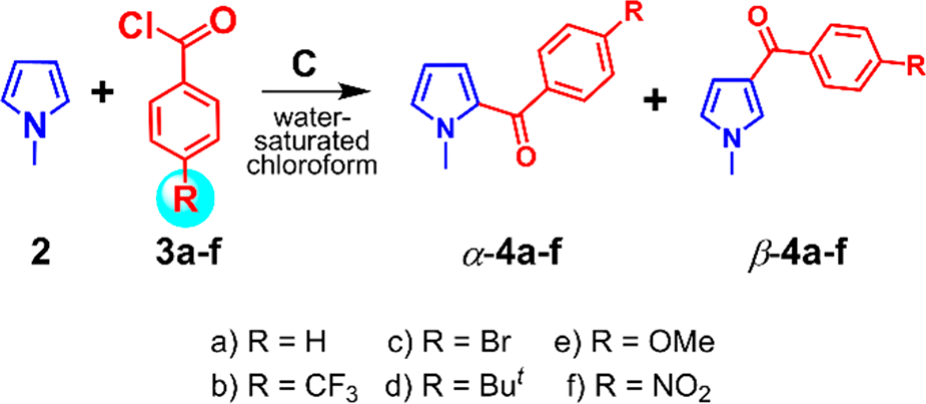
Friedel–Crafts Benzoylation of *N*-Methylpyrrole **2** and Benzoyl Chlorides **3a**–**f** Promoted by **C**

In the first instance, using water-saturated^5,6^ CHCl_3_ as the solvent, we investigated the reaction between *N*-methylpyrrole **2** and benzoyl chloride **3a** in the presence of **C** (Scheme 1). When *N*-methylpyrrole **2** was stirred with benzoyl chloride **3a** in water-saturated CHCl_3_ at 30 °C for 20 h, using a **2**/**3a** ratio of 1:4, in the presence of **C** (26 mol %), the product **4a**(10) was obtained in 99% yield (Table 1, entry 1). As in the case of the FC benzylation of **2** inside **C**,^4^ we observe a confinement effect on the regiochemistry. In fact, the β-regioisomer was preferentially formed with respect to α-**4a** with a β/α ratio of 60:40 (Table 1, entry 1). When the temperature is increased to 50 °C, the reaction time is shortened. In fact, in the presence of **C** (26 mol %) using a **2**/**3a** ratio of 1:4, product **4a** was obtained in 99% yield after 5 h (Table 1, entry 6). An increase in the amount of **2** (**2**/**3a** ratio of 1:1, entries 4 and 5 in Table 1) led to lower yields.

**Table 1:** FC Benzoylation of *N*-Methylpyrrole **2** under Different Reaction Conditions

Entry^a^	**C** (mol %)	**3**/**2**	*T* (°C)	*t* (h)	Yield (%)^b^	(α-**4**/β-**4**)
1	26	(**3a**) 4	30	20	99	40/60
2^c^	—	(**3a**) 4	30	20	—	—
3^c^^,^^d^	26	(**3a**) 4	30	20	—	—
4^e^	52	(**3a**) 1	30	20	70	45/55
5^f^	26	(**3a**) 1	30	20	70	50/50
6	26	(**3a**) 4	50	5	99	40/60
7^g^	26	(**3a**) 4	50	5	99	40/60
8	26	(**3b**) 4	50	5	99	50/50
9	26	(**3c**) 4	50	5	99	60/40
10	26	(**3d**) 4	50	5	99	60/40
11	26	(**3e**) 4	50	5	99	70/30
12	26	(**3f**) 4	50	5	99	100/–

a Unless noted otherwise, reaction conditions are **2** (84.9 μmol, 1 equiv), **3a**–**f** (339.8 μmol, 4.0 equiv), **C** (26 mol % corresponding to 127.4 μmol of **1**), H_2_O-saturated CHCl_3_ (0.55 mL).

b Yield of the product isolated by column chromatography.

c Only starting materials were recovered.

d The reaction was performed in the presence of tetraethylammonium tetrafluoroborate (0.76 M).

e The reaction was performed using **2** (1 equiv), **3a** (1 equiv), **C** (52 mol %), H_2_O-saturated CHCl_3_ (0.55 mL).

f The reaction was performed using **2** (1 equiv), **3a** (1 equiv), **C** (26 mol %), H_2_O-saturated CHCl_3_ (0.55 mL).

g Experiments on the reusability of **C**; the activity was maintained after different cycles.

In agreement with a standard protocol previously reported by us and others,^4,7,8^ a series of control experiments were performed to clarify the role of the capsule **C** in the FC benzoylation in Scheme 1. When the FC benzoylation of **2** with benzoyl chloride was performed in the presence of **C** and tetraethylammonium tetrafluoroborate (Table 1, entry 3, see SI), a known^4,7,8^ competitive guest with high affinity for the inner cavity of **C**, then no hint of product **4a** was observed. Analogously, no hint of the product was detected in the reaction mixture when the reaction was performed in the absence of **C** (Table 1, entry 2).

With these results in hand, we studied the scope of the FC benzoylation of *N*-methylpyrrole in the presence of **C** by exploring a variety of *p*-substituted benzoyl chlorides bearing electron-withdrawing (EW) or electron-donating (ED) groups at the *para* position of the benzyl ring. When *N*-methylpyrrole **2** was reacted with *p*-CF_3_-benzoyl chloride **3b** in the presence of **C** (26 mol %) at 50 °C for 5 h (Table 1, entry 8), the product **4b**(11) was obtained in 99% yield, with a 50/50 β/α ratio. Under the same conditions, by using as starting material *p*-Br-benzoyl chloride **3c** and *p*-Bu^*t*^-benzoyl chloride **3d**, the α-regioisomer was preferentially formed with a β-**4**/α**-4** regioselectivity of 40/60 for both (entries 9,10). Interestingly, with *p*-MeO-benzoyl chloride **3e**, marked regioselectivity for α-**4e** was observed with a β/α ratio of 30/70 (entry 11).^12^ When *N*-methylpyrrole **2** was reacted with *p*-NO_2_-benzoyl chloride **3f** in the presence of **C** (26 mol %) at 50 °C for 5 h (Table 1, entry 12), only the product α**-4** was obtained in 99% yield. Based on these results, we can conclude that when the FC benzoylation of **2** occurs inside the confined space of **C**, the regiochemistry of product **4** is driven by the confinement effects of the substrates.

At this point, we performed a quantum mechanical (QM) investigation to gain insight into the regiochemistry of FC benzoylation of **2** inside the confined space of **C**. In agreement with a standard protocol previously reported by us,^4^ a reduced capsule (**C**_**R**_) with shorter feet and the ONIOM method (M06-2X/PM6) were used to investigate the reaction between **2** and **3a** inside the confined space.^4^ First, the inclusion complex formation between the reactants and the supramolecular catalyst has been investigated. An energy stabilization of −5.57 and −5.84 kcal/mol was calculated for the encapsulating equilibrium of **2** and **3a** inside **C**_**R**_, respectively (Figures 2 and S27). These results suggest that the first species to enter the capsule is **3a**, followed by **2**. Then, the involvement of the bridge water molecules in **C**_**R**_ has been examined. The oxygen atom of the carbonyl group of **3a** establishes a hydrogen bonding interaction with a water molecule of **C**_**R**_ (MC in Figure 2). Differently, inside the capsule, the **3f** derivative establishes two different H-bonds (Figure S26), the first between a capsular water molecule and the O=C group (same as **3a**) of **3f** and the second one between the opposite capsular water molecule and the nitro group of **3f**.

**Figure 2:**
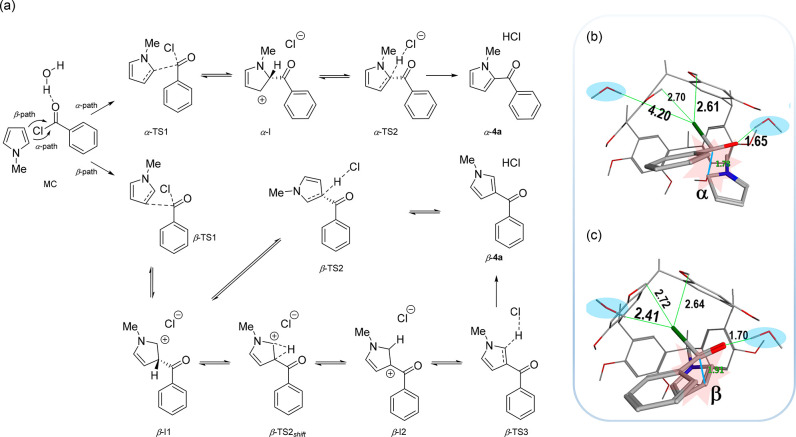
(a) Alpha and beta channels for the in silico studies of the FC and nomenclature adopted. The capsule **C**_**R**_ is always present. (b,c) O–H···Cl distances, measured in Å, between the leaving Cl and the closest resorcinarene–OH and capsular water (in light blue) in α-TS1 (b) and β-TS1 (c).

The formation of the molecular complex [**2**+**3a**]@**C**_**R**_ was looked into, and QM calculations indicate that in the heterocomplex (MC in Figure 2) pyrrole **2** points its *N*-methyl group inside a resorcinarene cavity to establish CH···π interactions (Figure S26). Furthermore, the aromatic pyrrole unit is close to the reactive carbonyl group of **3** (Figure 2b,c) with a Gibbs free energy 13.31 kcal/mol lower than those of the three separate entities (Figure S27). The mechanism of the Friedel–Crafts benzoylation was then investigated. The calculations indicate that the reaction may proceed through two paths for the α and β products (Figure 2). Concerning the α path, an activation energy of 25.29 kcal/mol was calculated for α-TS1 (Figure S27), which leads to the corresponding Wheland intermediate, α-I, located 4.60 kcal/mol below TS1 (Figure S27). The loss of the hydrogen atom from the intermediate proceeds very quickly with a low energetic barrier of 5.01 kcal/mol. The product α-**4a** is located 7.87 kcal/mol below the starting reagents, making the reaction exergonic. Concerning the β-path, the Wheland intermediate β-I1 is allocated inside **C**_**R**_ with a geometry analogous to that described previously by us^4^ for the FC benzylation of **2**, in which the hydrogen atom and the Cl^–^ are located on opposite orientations with respect to the pyrrole ring, and direct extraction of the hydrogen seems difficult without a C–C bond rotation. Consequently, a TS for a [1,2]-H shift was initially calculated (β-TS2_*shift*_, 17.75 kcal/mol; see also Figure S27) to produce another intermediate β-I2. The loss of the hydrogen atom from β-I2 proceeds very quickly with an energetic barrier of only 1.51 kcal/mol, and the β-**4a** derivative is located 10.37 kcal/mol below the starting reagents. An additional β-TS2 was also calculated for direct deprotonation of β-I1 after a (β-pyrrole) C–CO rotation. This β-TS2 resulted in a lower energy (β-TS2, 4.81 kcal/mol) than the former calculated β-TS2_*shift*_, suggesting that the reaction may proceed after this geometry rearrangement for the formation of the β product.

QM calculations indicate that the ***β*****-4a@C**_**R**_ complex is thermodynamically more stable than ***α*****-4a@C**_**R**_ by 2.5 kcal/mol (Figure S27). However, the reaction produces both ***β*****-4a**/***α*****-4a** regioisomers because the retro-FC from ***α*****-4a** to ***α*****-I** and ***β*****-4a** to ***β*****-I1** is slowed down by a very high energy barrier (33.58 and 27.17 kcal/mol, Figure S27). Contrarily, DFT calculations clearly showed that the formation of the α-regioisomer during the FC benzylation of **2** with benzyl chloride^4^ was reversible (the retro-FC occurred with a low energy barrier), and in the long run, the reaction only gave the thermodynamic β-regioisomer.^4^

The regiochemistry of the FC benzoylation of **2** inside the confined space of **C**_**R**_ was then studied. Usually, electrophilic substitution in pyrrole occurs faster at the 2-position than at the 3-position. The standard explanation for the attack at C-2 is based on the relative energies of the intermediates. In agreement with the valence-bond description, the conjugated system of the α intermediate (Figure 2) is linearly conjugated with the *N*-lone pair, overlapping with the π system of an allyl cation, whereas the conjugated system of the β intermediate has the *N*-lone pair overlapping with the π bond and an isolated cation. Hence, the α linearly conjugated system is lower in energy than the cross-conjugated β intermediate. In agreement with the frontier molecular orbital (FMO) theory, the C-2 attack is favored. The frontier orbitals of the reactants have been analyzed when inside **C**_**R**_. The HOMO of **2** has a node running through the heteroatom with a higher orbital coefficient at C-2 and an estimate of the C-2 charge lower than C-3. Despite the insertion of **2** inside the capsule lowering the energy of its HOMO, the geometry of the orbital coefficients is not influenced; hence, the reactivity is still very similar to that of an isolated **2** with a predicted selectivity of C-2 over C-3. Considering this result, the different regioselectivities observed for the reactions in Scheme 1, inside the confined space of **C**, could be controlled by the LUMO energies of **3a**–**f** derivatives (see Table S2). As pointed out by the calculated FMO energies of the isolated **3a**, **3e**, and **3f**, the ΔLUMO(**3**)–HOMO(**2**) energies are ranked as expected: **3f** < **3a** < **3e** for isolated systems reflecting the electron-withdrawing and -donating effects of the substituents. FMO energies were then calculated for **3a**, **3e**, and **3f** inside the supramolecular catalyst. Inside the capsule, benzoyl chlorides **3a**–**f** establish secondary interactions with the aromatic cavity and capsular water molecules, adopting different geometries concerning their functional groups. In fact, while **3a** engages an H-bond with a capsular water molecule (green in Figure 1), *para*-substituted derivatives **3e** and **3f** form an additional H-bonding interaction between another water molecule and the methoxy and nitro groups, respectively. In detail, the FMO calculations indicate that inside the capsule, the LUMO energies of **3a**–**f** derivatives are higher than in the bulk solvent; however, due to the different interaction geometries of the three analyzed reagents **3a**–**f**, a greater LUMO energy increase was observed for **3a** with respect to **3e** (**3f** < **3e** < **3a**). For **3f**, the electron-withdrawing (EW) NO_2_ group in the *para*-position of the molecule is interacting through an H-bond with a water molecule, which does not significantly change the reactivity of **3f**, which still reacts under FMO control. For **3a** and **3e**, an electronic control seems relevant to form the β-products. **3a** and **3e** engage an H-bond between a water molecule and their carbonyl group. In addition, **3e** forms an H-bond between the MeO group in the *para*-position and a water molecule. The C=O···HOH H-bond increases the LUMO energy, whereas in the presence of the MeO···HOH H-bond, the electron-donating ability of the OMe group is lowered and the LUMO energy is lowered at a value lower than **3a**. The overall result is that the β-product is formed for both **3a** and **3e** derivatives, which explains the experimentally measured data.

With these results in hand, we hypothesized that the reaction occurs under FMO control for the more reactive **3f** and under electronic control for the less reactive **3a** and **3e**; moreover, the product distribution is ruled by the first transition state of the reaction path (TS1). The structure of the transition state ruling the product selectivity (TS1) and the reaction mechanism is worth discussing due to the peculiarity of this TS if compared with classical nucleophilic substitutions at the carbonyl. Indeed, nucleophilic substitutions at the carbonyl group are considered to proceed via an addition–elimination reaction with a tetrahedral intermediate.^13^ Nevertheless, there is evidence that bimolecular concerted S_N_2 processes can also occur during the hydrolysis of benzoyl chlorides bearing EW (4-NO_2_) or ED (4-MeO) groups. In the proposed mechanism,^13^ a network of water is establishing H-bonding interactions with the leaving chlorine atom. Based on these considerations, it is not unreasonable to ask if an analogous bimolecular concerted S_N_2 mechanism occurs between **2** and **3** inside the confined space of **C**, supported by H-bonding interactions with the bridged water molecules.

Our QM calculations suggest that the bimolecular concerted S_N_2 mechanism and a relevant contribution to the H-bonding interactions with the bridged water molecules could be ascribed. Concerning the structure of α-TS1 (Figures 2b and 3), calculations clearly indicated the formation of two H-bonding interactions, C(O)–Cl···HO–resorcinarene with Cl···H distances of 2.61 and 2.70 Å (Figures 2b and 3). Analogous results were obtained for β-TS1 (Figures 2c and 3). Calculations also indicated that the bridged water molecules played a crucial role in determining the greater stability of β-TS1 with respect to α-TS1. In fact, in β-TS1, the leaving chlorine atom establishes a stronger H-bonding interaction than in α-TS1, with a capsular water molecule to a Cl···H distance of 2.41 Å, while in α-TS1, the closest water molecule is at 4.21 Å from the chlorine atom (Figures 2b,c, and 3). This result further corroborates the preferential formation of β-**4a** and the lowest calculated energy for the β-TS1.

**Figure 3:**
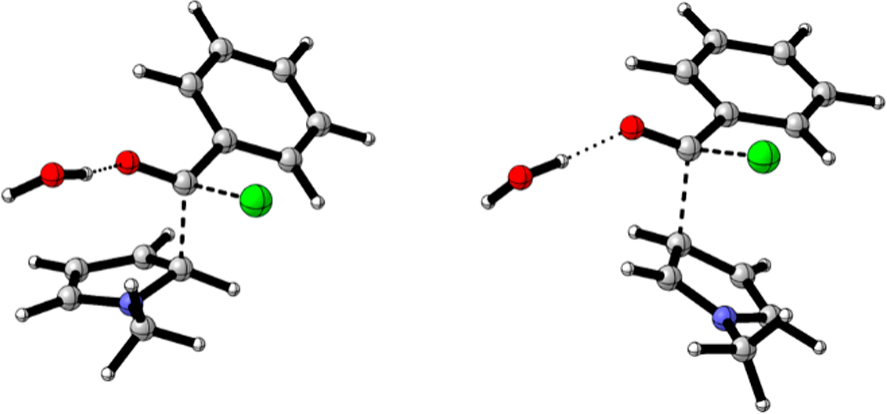
Geometries for TS states associated with the α and β path of the bimolecular concerted S_N_2 processes are shown in Figure 2. The capsule has been omitted for clarity. Carried out with CYLview.

The TS1 was then calculated for the formation of **4f**. The geometries of the two α/*β–*TS1 are shown in Figure S28c,d. The free Gibbs activation energy involved in adding **2** to **3f** can justify the experimentally measured ratio between α-**4f** and β-**4f**. The calculated TS1 energies are 7.35 and 11.57 kcal/mol for α-**4f**-TS1 and β-**4f**-TS1, respectively. The −4.21 kcal/mol of the α-**4f**-TS1 well explains the only formation of the α-**4f** product, according to our proposed mechanism.

In conclusion, the resorcinarene capsule can work as an organocatalyst for a sustainable, metal-free Friedel–Crafts benzoylation of *N*-methylpyrrole. Our calculations indicate that the supramolecular catalyst can catalyze the benzoylation of **2**, as reported by the experimental data. The regiochemistry of the reaction may be explained by FMO theory, and electronic control of the reaction seems relevant for product formation for the studied systems **3a** and **3e**. QM calculations confirm the catalytic role of the capsular water molecules of **C**, which act as H-bond donor groups to polarize the C–Cl bond and activate the carbonyl group. The confined space inside **C** plays a crucial role in determining the regiochemistry of the FC benzoylation of *N*-methylpyrrole. Calculations suggest that the shape and size of the substituent at the *para*-position of **3** and its ability to engage H-bonds with the bridged water molecules work synergistically with the steric constrictions imposed by the hexameric capsule to drive the regiochemistry of FC benzoylation of **2**. Finally, QM calculations suggest that inside the confined space of **C**, the FC benzoylation of **2** occurs by a bimolecular concerted S_N_2 mechanism in which both the leaving chlorine atom and the carbonyl group establish H-bonding interactions with the capsular water molecules of **C**.

## Data Availability

The data underlying this study are available in the published article and its Supporting Information

## Supporting Information Available

Detailed synthetic procedures, proofs of the encapsulation of **3a** inside **C**, NMR and HR MS spectra of new compounds. Details of QM calculations (PDF)
