# Supplementary material for: Insights into the Friedel–Crafts Benzoylation of N-Methylpyrrole inside the Confined Space of the Self-Assembled Resorcinarene Capsule
Source: Org Lett. 2023 Aug 29;25(35):6464–8. doi: 10.1021/acs.orglett.3c01935 (PMC10496122; doi:10.1021/acs.orglett.3c01935)
Supplement: Supplementary file 1 — ol3c01935_si_001.pdf [file ol3c01935_si_001.pdf]

Supporting Information

Insights into the Friedel-Crafts Benzoylation of *N*-Methylpyrrole Inside the Confined Space of the Self-Assembled Resorcinarene Capsule

Veronica Iuliano,[a] Carmen Talotta,[a,*] Margherita De Rosa,[a] Annunziata Soriente,[a] Placido Neri,[a] Antonio Rescifina,[b] Giuseppe Floresta[b,*] Carmine Gaeta[a,*]

[a]Laboratory of Supramolecular Chemistry, Dipartimento di Chimica e Biologia "A. Zambelli", Università di Salerno, Via Giovanni Paolo II 132, I-84084 Fisciano, Salerno, Italy; [b]Dipartimento di Scienze del Farmaco, Università di Catania, Viale Andrea Doria 6, 95125 Catania, Italy

Table of Contents

1. General Remarks S3

2. General Procedure S3

3. ^{1}H and ^{13}C NMR Spectra and HR MS Spectra of Derivatives **4a-f**....S8

Derivative α-**4a** S8

Derivative β-**4a**....S9

Derivative α-**4b** S10

Derivative β-**4b** S11

Derivative α-**4c** S14

Derivative β-**4c**....S15

Derivative α-**4d** S16

Derivative β-**4d** S19

Derivative α-**4e** S22

Derivative β-**4e**....S23

Derivative α-**4f** S24

4. Encapsulation Proofs of **3a** inside the resorcinarene capsule **C** S25

5. Computational Studies S32

6. References S210

1. General Remarks

All chemicals were reagent grade and were used without further purification. Solvents were purchased from Aldrich. Reaction temperatures were measured externally; reactions were monitored by ^{1}H NMR and by TLC on Merck silica gel plates (0.25 mm) and visualized by UV light. Flash chromatography was performed on Merck silica gel (60, 40-63 μm). NMR spectra were recorded on Bruker Avance-600 spectrometer [600.13 MHz (1H) and 150.03 MHz (^{13}C)], Bruker Avance-400 spectrometer [400 (1H) and 100.57 MHz (13C)], Bruker Avance-300 spectrometer [300 (^{1}H) and 75.48 MHz (^{13}C)], or Bruker Avance-250 spectrometer [250 (1H) and 62.80 MHz (^{13}C)]; chemical shifts are reported relative to the residual solvent peak ($CHCl_3$: δ 7.26, $CDCl_3$: δ 77.23). HR ESI mass spectra were recorded on a Bruker Solarix FT-ICR mass spectrometer equipped with a 7T magnet. The samples recorded in ESI were prepared by mixing analyte in chloroform (10 μg/mL). The mass spectra were calibrated externally, and a linear calibration was applied. The final compounds purity was found to be >95% when analyzed. Resorcinarene **1** was synthesized according to literature procedures.[1]

2. General Procedure

Typical procedure for Friedel-Crafts Acylation Reaction inside the confined space of the hexameric capsule ***C***.

1st Step. Self-assembling of the hexameric resorcinarene capsule **C.**

C-Undecylresorcin[4]arene (140.9 mg, 127.4 μmol, 1.5 equiv, corresponding to 0.26 equiv of hexameric capsule **C**), were mixed in 0.55 mL of water saturated chloroform (6 uL of H_2O in 0.55 mL of $CHCl_3$) , and the mixture was sonicated in an ultrasonic water bath at 50 °C until clarification (ca. 10 min).[2]

2nd Step. Subsequently, *N*-methylpyrrole **2** (84.9 μmol, 1 equiv) and benzoyl chloride **3a-f** (339.8 μmol, 4.0 equiv) were added, and the solution was stirred at 50 °C in an oil bath for 5 h. Then the reaction mixture was poured into a 50 mL Eppendorf conical tube and diluted with 40 mL of a solution of DMSO in *n*-hexane (0.13 % (v/v)). The solution was cooled at -20 °C until precipitation of C-undecylresorcin[4]arene which was removed by centrifugation (1750 rpm for 10 min). The solution was dried under vacuum and the resorcinarene in excess was removed by precipitations from *n*-hexane (3x). The crude product was purified by flash chromatography on silica gel using a gradient of *n*-hexane/$CHCl_3$ (80/20→50/50). All products were isolated and characterized.

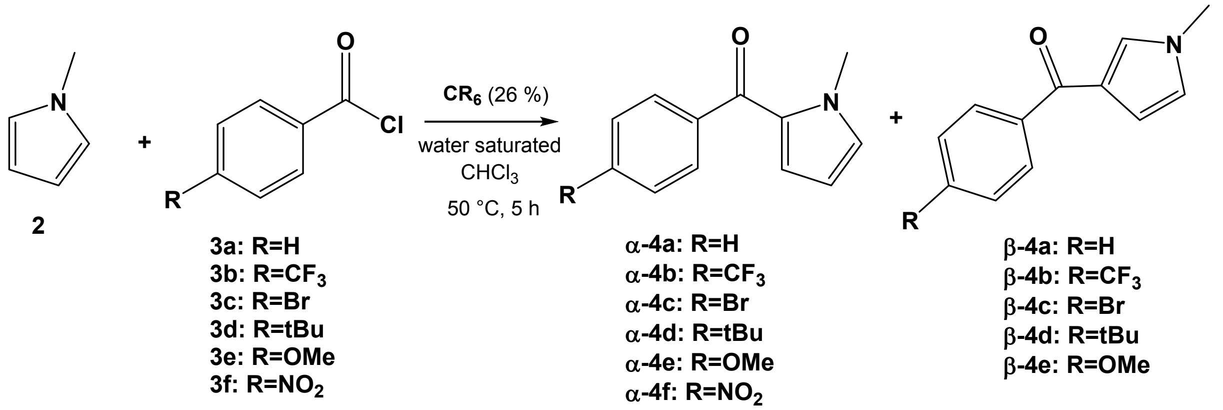

Scheme S1. Friedel-Crafts acylation of *N*-methylpyrrole with *para*-substituted benzoyl chlorides performed in the presence of **C**.

The regioisomeric ***α*-4a-f**/***β*-4a-f** ratio was calculated by integration of N-CH_3 singlets of ***α*-4a-f** and ***β*-4a-f** in ^{1}H NMR spectra of their crude reaction mixtures in acetone-D_6 (see Figure S1 as representative example).

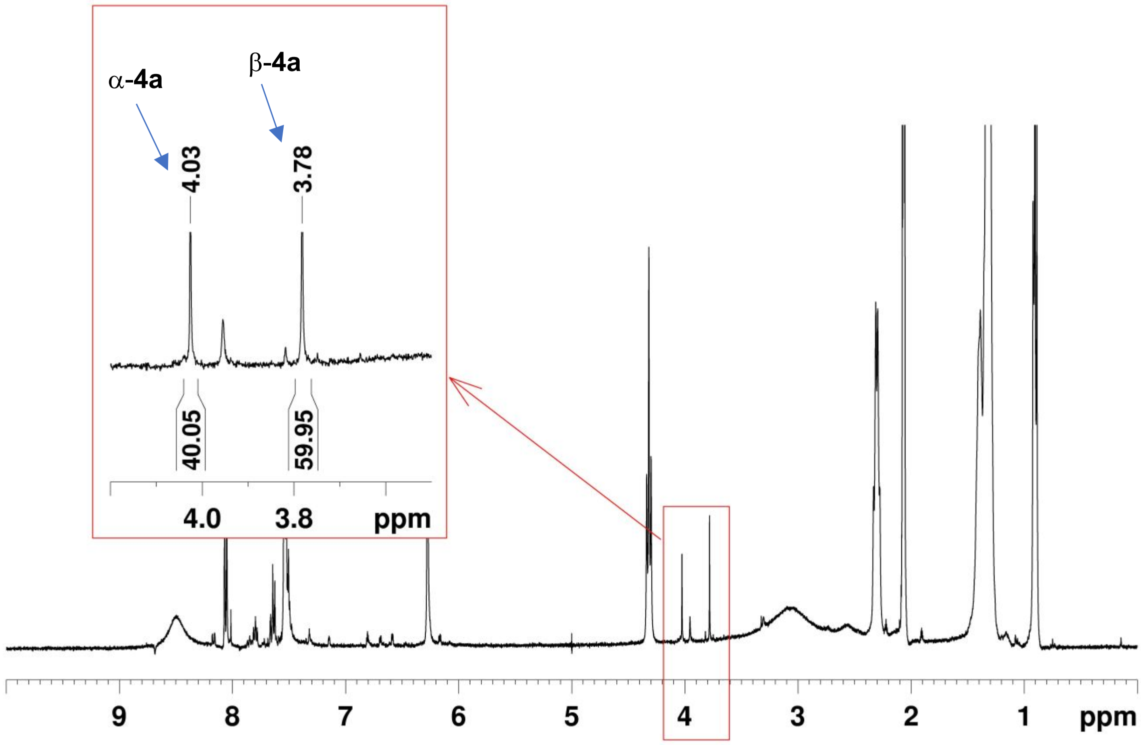

Figure S1. ^{1}H NMR spectrum of the crude reaction mixture (Acetone-d6, 400 MHz, 298 K), entry 1, Table 1 in the main text.

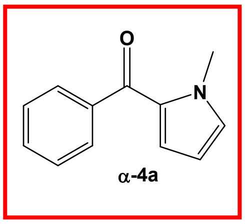

Derivative *α*–4a. Following the general procedure reported in previously paragraph, the pure derivative ***α*–4a** was isolated by flash chromatography on silica gel using a gradient of *n*-hexane/$CHCl_3$ (80/20→50/50) as a clear oil (6.2 mg, 40 % yield). **^{1}H NMR** (600 MHz, $CDCl_3$, 298K): δ 7.79 (d, J = 7.6 Hz, 2H), 7.53 (t, J=7.6 Hz, 1H), 7.44 (dd, $J_1 = J_2$ = 7.6 Hz, 2H), 6.92 (broad, 1H), 6,73 (broad, 1H), 6.16 (broad, 1H), 4.04 (s, 3H).[3]

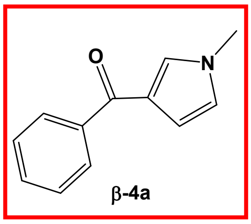

Derivative *β*–4a. Following the general procedure reported in previously paragraph, the pure derivative ***β*–4a** was isolated by flash chromatography on silica gel using a gradient of *n*-hexane/$CHCl_3$ (80/20→50/50) as a

clear oil (9.4 mg, 60 % yield). **^{1}H NMR** (600 MHz, $CDCl_3$, 298K): δ 7.81 (d, *J*=7.4 Hz, 2H), 7.52 (t, *J*=7.4 Hz, 1H), 7.45 (dd, $J_1 = J_2$ = 7.4 Hz, 2H), 7.18 (broad, 1H), 6.68 (broad, 1H), 6.64 (broad, 1H), 3.71 (s, 3H).[4]

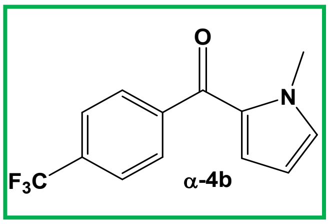
α-4b

Derivative *α*-4b Following the general procedure reported in previously paragraph, the pure derivative ***α*-4b** was isolated by flash chromatography on silica gel using a gradient of *n*-hexane/$CHCl_3$ (80/20→50/50) as a clear oil (10.5 mg, 50 % yield). **^{1}H NMR** (600 MHz, $CDCl_3$, 298K): δ 7.87 (d, *J*=8.48 Hz, 2H), 7.71 (d, *J*=8.48 Hz, 2H), 6.96 (broad, 1H), 6.70 (broad, 1H), 6.17 (broad, 1H), 4.05 (s, 3H).[5]

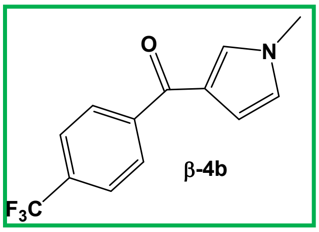
β-4b

Derivative *β*-4b Following the general procedure reported in previously paragraph, the pure derivative ***β*-4b** was isolated by flash chromatography on silica gel using a gradient of *n*-hexane/$CHCl_3$ (80/20→50/50) as a clear oil (10.3 mg, 50 % yield). **^{1}H NMR** (600 MHz, $CDCl_3$, 298K): δ 7.89 (d, *J*=8.48 Hz, 2H), 7.71 (d, *J*=8.48 Hz, 2H), 7.17 (broad, 1H), 6.66 (overlapped, 2H), 3.72 (s, 3H). **^{13}C NMR** (150 MHz, $CDCl_3$, 298K): δ 189.3, 143.2, 132.6, 129.1, 129.0, 125.2, 125.2, 124.2, 123.7, 111.1, 36.7. **HR-MS**: m/z $[M+H]^+$ calcd for $C_{13}H_{11}NOF_3^+$ 254.0787; found 254.0794.

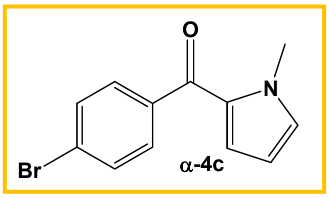
α-4c

Derivative *α*-4c. Following the general procedure reported in previously paragraph, the pure derivative ***α*-4c** was isolated by flash chromatography on silica gel using a gradient of *n*-hexane/$CHCl_3$ (80/20→50/50) as a clear oil (13.0 mg, 60 % yield). **^{1}H NMR** (400 MHz, $CDCl_3$, 298K): δ 7.67 (d, J=8.3 Hz, 2H), 7.59 (d, J=8.3 Hz, 2H), 6.93 (broad, 1H), 6,70 (broad, 1H), 6.16 (broad, 1H), 4.03 (s, 3H).[3]

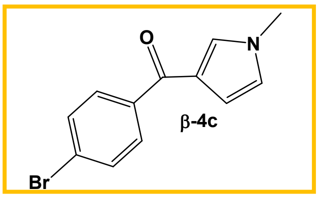
β-4c

Derivative *β*-4c. Following the general procedure reported in previously paragraph, the pure derivative ***β*-4c** was isolated by flash chromatography on silica gel using a gradient of *n*-hexane/$CHCl_3$ (80/20→50/50) as a clear oil (9.0 mg, 40 % yield). **^{1}H NMR** (300 MHz, $CDCl_3$, 298 K): δ 7.70 (d, J=8.6 Hz, 2H), 7.58 (d, J=8.6 Hz, 2H), 7.17 (broad, 1H), 6,65 (overlapped, 2H), 3.72 (s, 3H).[4]

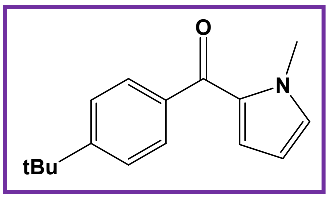

Derivative *α*-4d. Following the general procedure reported in previously paragraph, the pure derivative ***α*-4d** was isolated by flash chromatography on silica gel using a gradient of *n*-hexane/$CHCl_3$ (80/20→50/50) as a clear oil (12.0 mg, 60 % yield). **^{1}H NMR** (400 MHz, $CDCl_3$, 298K): δ 7.76 (d, *J*=8.5 Hz, 2H), 7.46 (d, *J*=8.5 Hz, 2H), 6.91 (broad, 1H), 6.77 (broad, 1H), 6.15 (broad, 1H), 4.03 (s, 3H), 1.36 (s, 9H). **^{13}C NMR** (150 MHz, $CDCl_3$, 298K): δ 186.0, 155.0, 137.1, 131.2, 130.6, 129.2, 125.0, 122.5, 107.9, 37.3, 35.0, 31.2. **HR-MS**: m/z $[M+H]^+$ calcd for $C_{16}H_{20}NO^+$ 242.1539 found 242.1539; $[M+Na]^+$ calcd for $C_{16}H_{19}NONa^+$ 264.1359; found 264.1358.

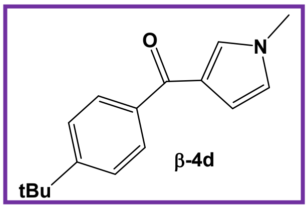

β-4d

Derivative *β*-4d. Following the general procedure reported in previously paragraph, the pure derivative ***β*-4d** was isolated by flash chromatography on silica gel using a gradient of *n*-hexane/$CHCl_3$ (80/20→50/50) as a clear oil (8.0 mg, 40 % yield). **^{1}H NMR** (600 MHz, $CDCl_3$, 298K): δ 7.77 (d, *J*=8.5 Hz, 2H), 7.46 (d, *J*=8.5 Hz, 2H), 6.91 (broad, 1H), 6.77 (broad, 1H), 6.15 (broad, 1H), 4.03 (s, 3H), 1.36 (s, 9H). **^{13}C NMR** (150 MHz, $CDCl_3$, 298K): δ 190.3, 154.8, 137.4, 128.9, 128.8, 125.1, 124.8, 123.1, 111.2, 36.7, 35.0, 31.3. **HR-MS**: m/z $[M+H]^+$ calcd for $C_{16}H_{20}NO^+$ 242.1539 found 242.1539; $[M+Na]^+$ calcd for $C_{16}H_{19}NONa^+$ 264.1359; found 264.1358.

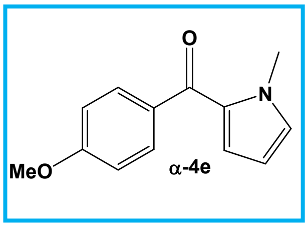

α-4e

Derivative *α*-4e. Following the general procedure reported in previously paragraph, the pure derivative ***α*-4e** was isolated by flash chromatography on silica gel using a gradient of *n*-hexane/$CHCl_3$ (80/20→50/50) as a clear oil (13.0 mg, 70 % yield). **^{1}H NMR** (400 MHz, $CDCl_3$, 298K): δ 7.84 (d, *J*=8.5 Hz, 2H), 6.95 (d, *J*=8.5 Hz, 2H), 6.89 (broad, 1H), 6.72 (broad, 1H), 6.15 (broad, 1H), 4.01 (s, 3H), 3.88 (s, 3H). [3]

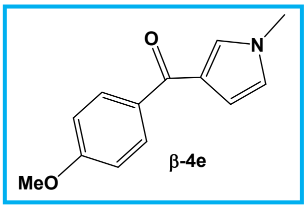

Derivative *β*–4e. Following the general procedure reported in previously paragraph, the pure derivative ***β*–4e** was isolated by flash chromatography on silica gel using a gradient of *n*-hexane/$CHCl_3$ (80/20→50/50) as a clear oil (5.0 mg, 30 % yield). **^{1}H NMR** (300 MHz, $CDCl_3$, 298K): δ 7.86 (d, *J*=8.5 Hz, 2H), 7.19 (broad, 1H), 6.96 (d, *J*=8.5 Hz, 2H), 6.65 (overlapped, 2H), 3.88 (s, 3H), 3.72 (s, 3H).[6]

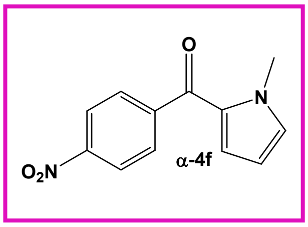

Derivative *α*-4f. Following the general procedure reported in previously paragraph, the pure derivative ***α*-4f** was isolated by flash chromatography on silica gel using a gradient of *n*-hexane/$CHCl_3$ (80/20→50/50) as a clear oil (19.0 mg, 99 % yield). **^{1}H NMR** :(300 MHz, $CDCl_3$, 298K): δ 8.30 (d, *J*=8.6 Hz, 2H), 7.91 (d, *J*=8.6 Hz, 2H), 7.00 (broad, 1H), 6.68 (broad, 1H), 6.19 (broad, 1H), 4.06 (s, 3H).[3]

3. ^{1}H and ^{13}C NMR Spectra and HR MS Spectra of Derivatives 4a-f

Derivative *α*-4a

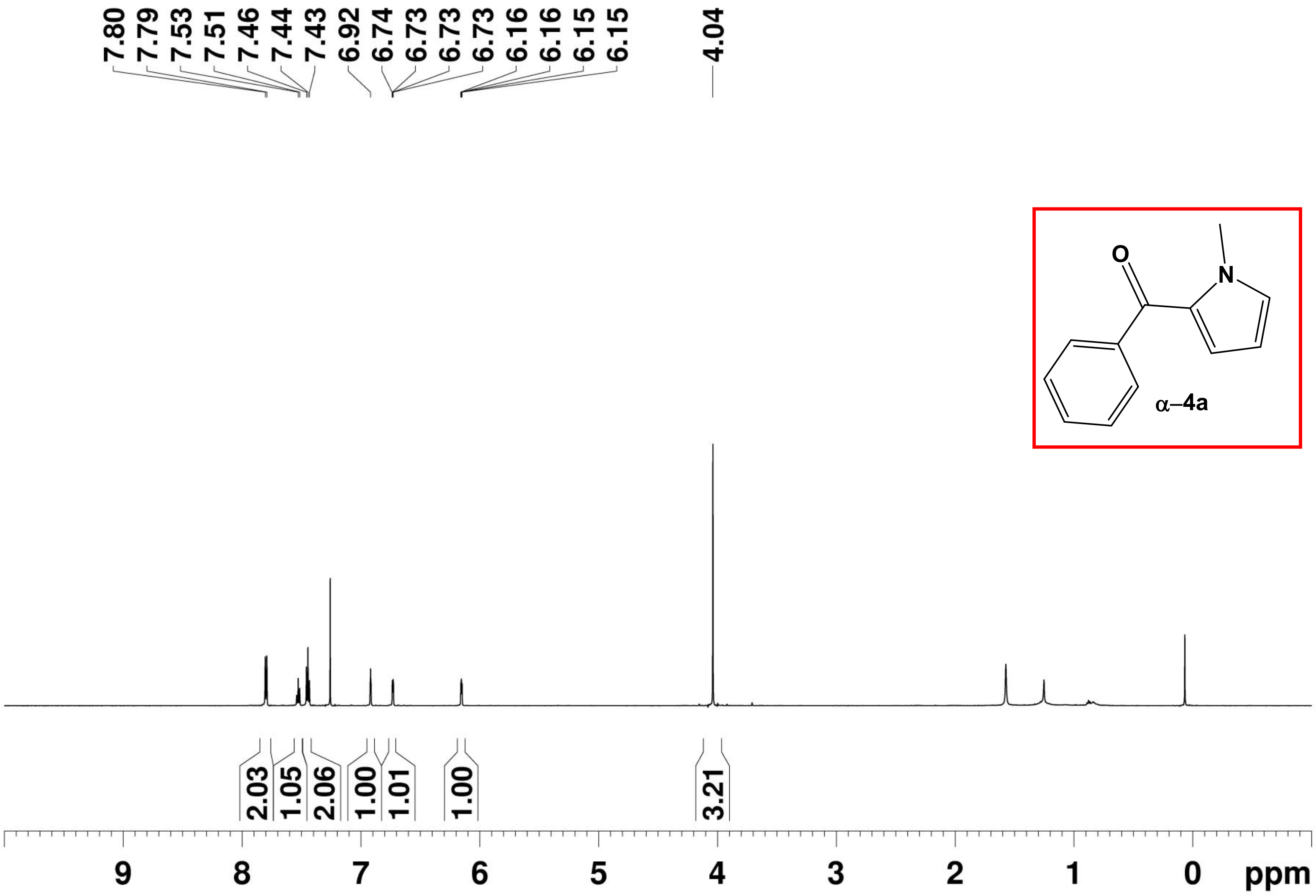

Figure S2: ^{1}H NMR spectrum of derivative ***α*-4a** (600 MHz, $CDCl_3$, 298 K).

Derivative *β*-4a

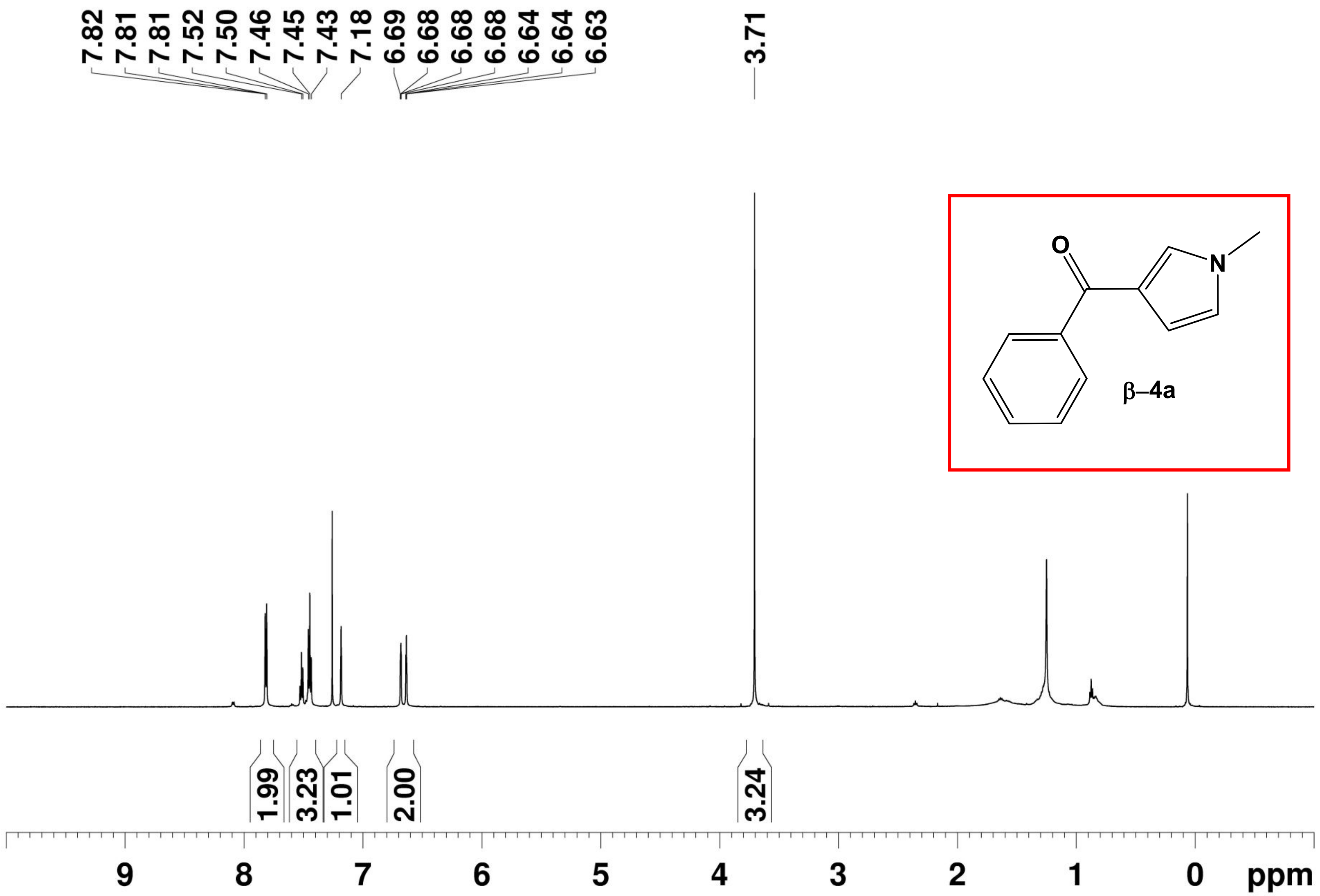

Figure S3: ^{1}H NMR spectrum of derivative ***β*-4a** (600 MHz, $CDCl_3$, 298 K).

Derivative α-4b

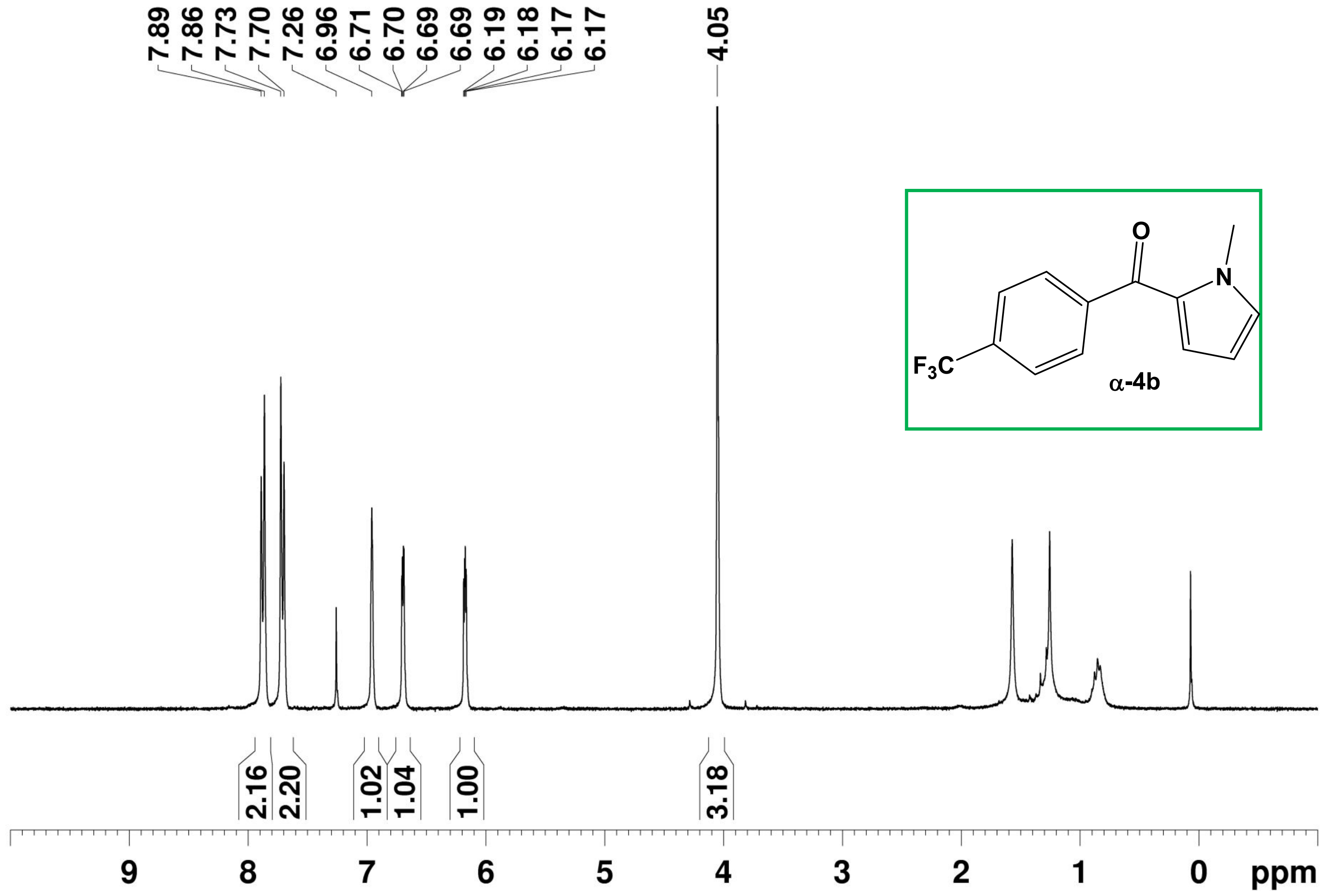

Figure S4: ^{1}H NMR spectrum of derivative α**-4b** (600 MHz, $CDCl_3$, 298 K).

Derivative β-4b

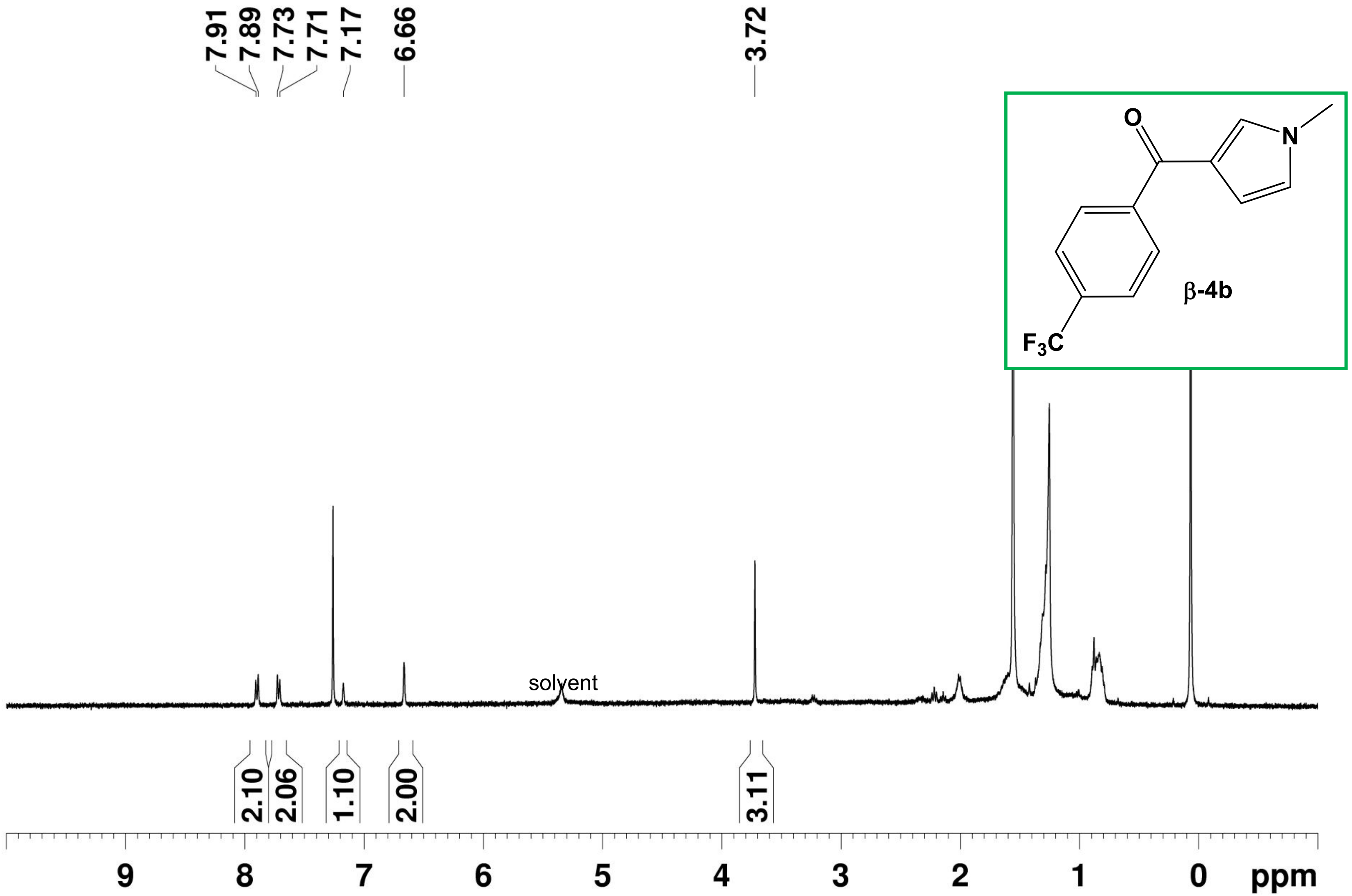

Figure S5: ^{1}H NMR spectrum of derivative ***β*-4b** (600 MHz, $CDCl_3$, 298 K).

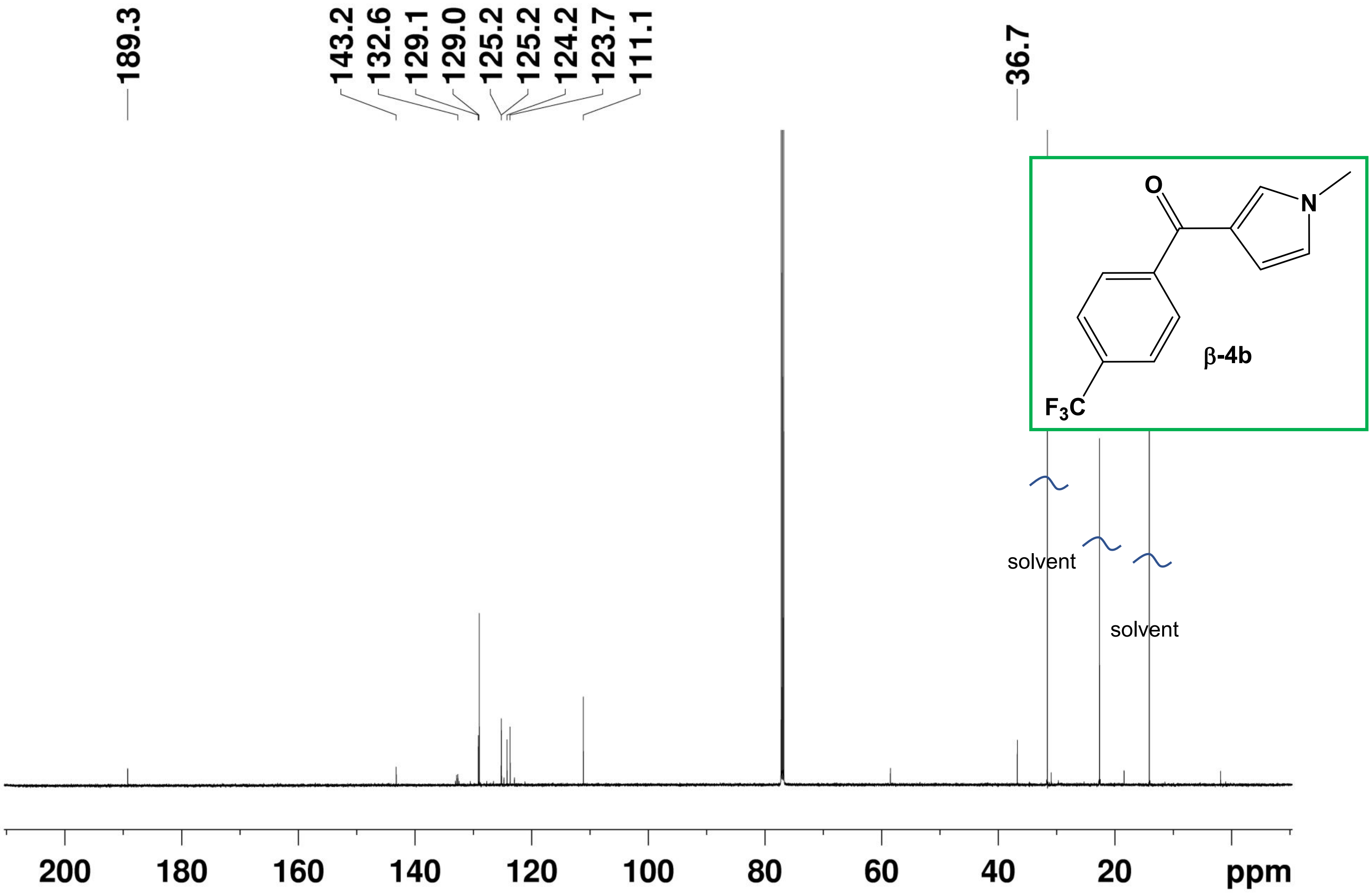

Figure S6. ^{13}C NMR spectrum of derivative ***β*-4b** (150 MHz, $CDCl_3$, 298 K).

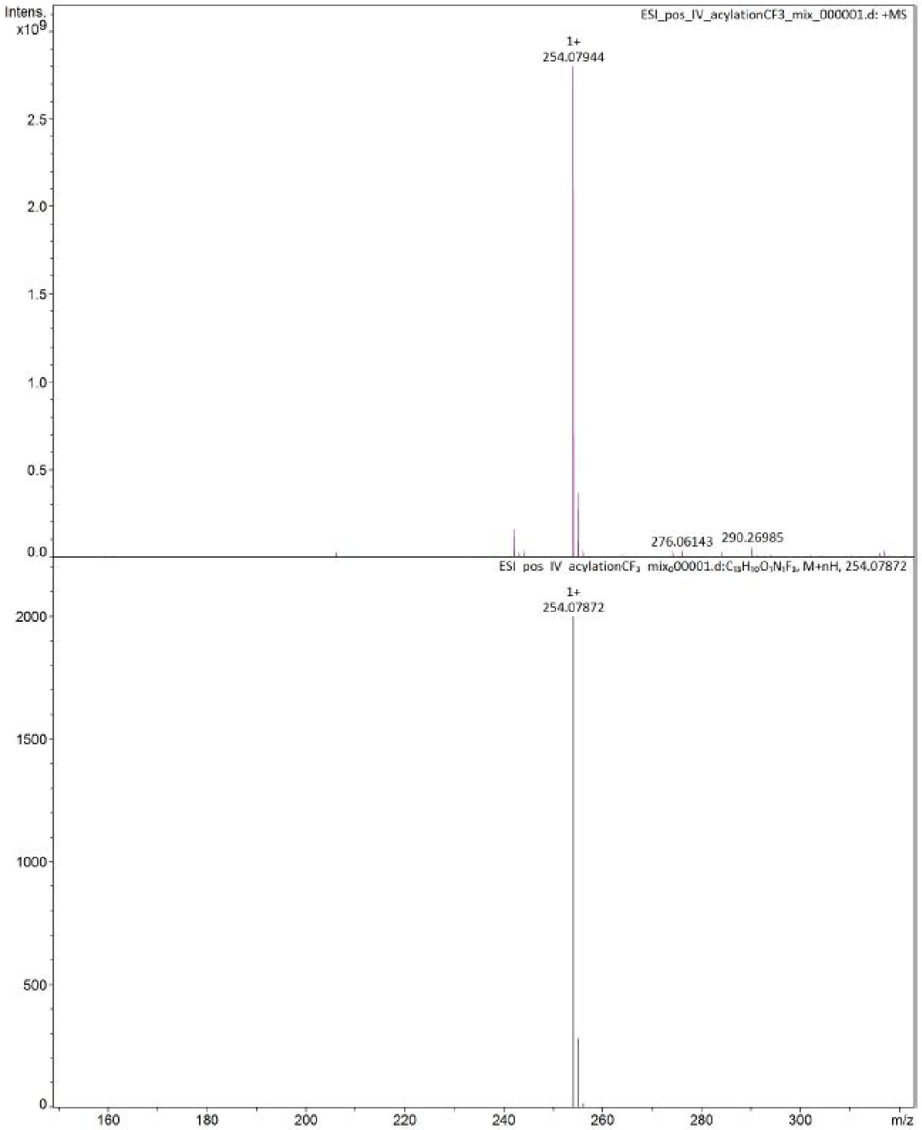

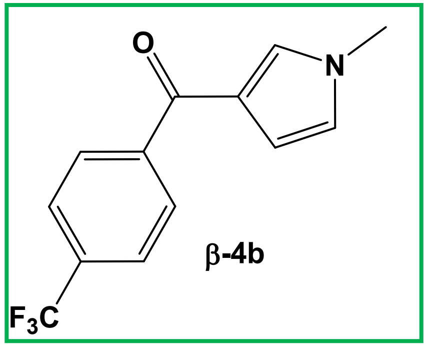

Figure S7: (top) HR-MS spectrum of derivative *β*-**4b**; (bottom) Simulated HR-MS spectrum of derivative *β*-**4b;**

m/*z* $[M+H]^+$ calcd for $C_{13}H_{11}NOF_3^+$ 254.0787 found 254.0794.

Derivative α-4c

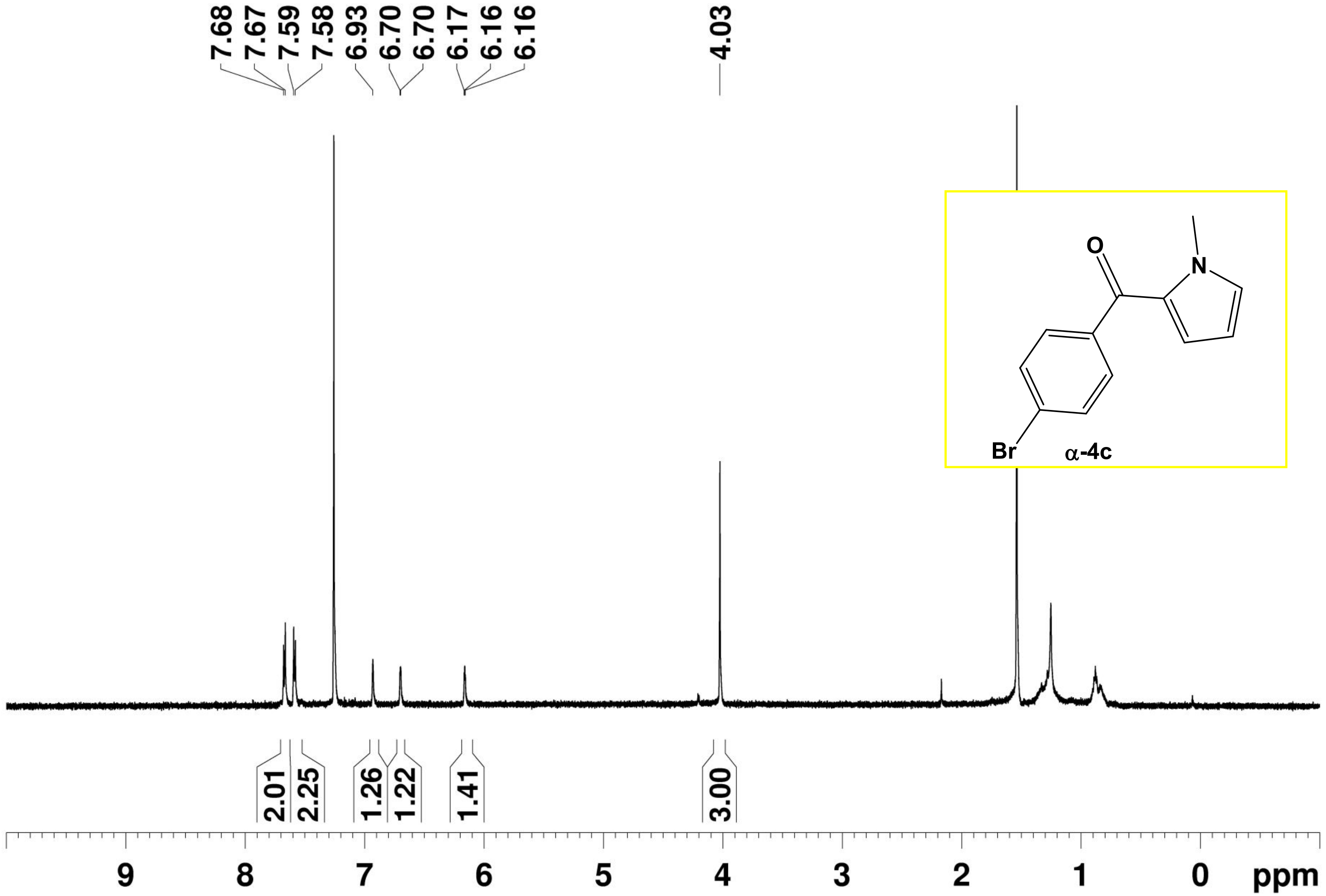

Figure S8: ^{1}H NMR spectrum of derivative *α*-**4c** (300 MHz, $CDCl_3$, 298 K).

Derivative β-4c

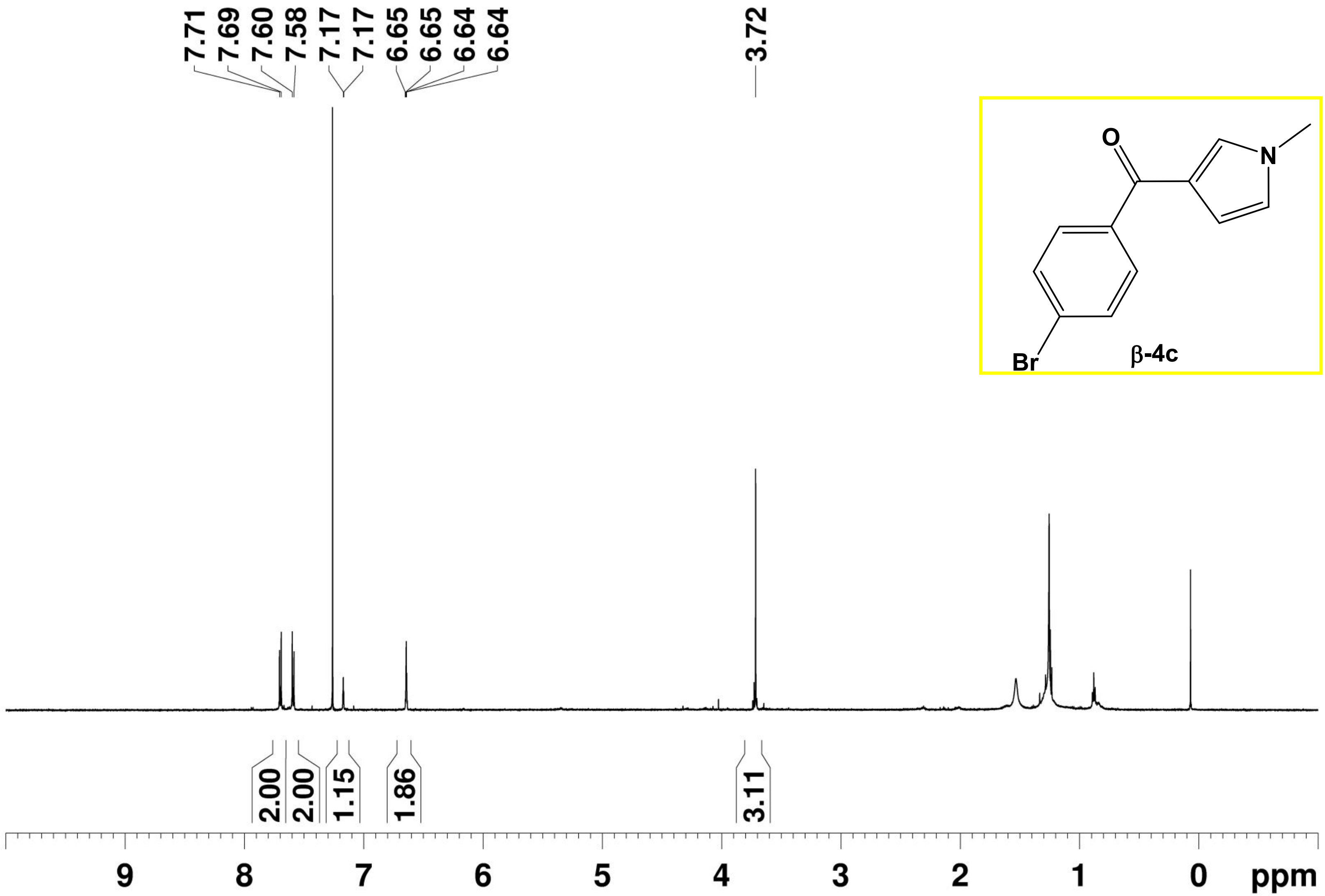

Figure S9: ^{1}H NMR spectrum of derivative ***β*-4c** (300 MHz, $CDCl_3$, 298 K).

Derivative α-4d

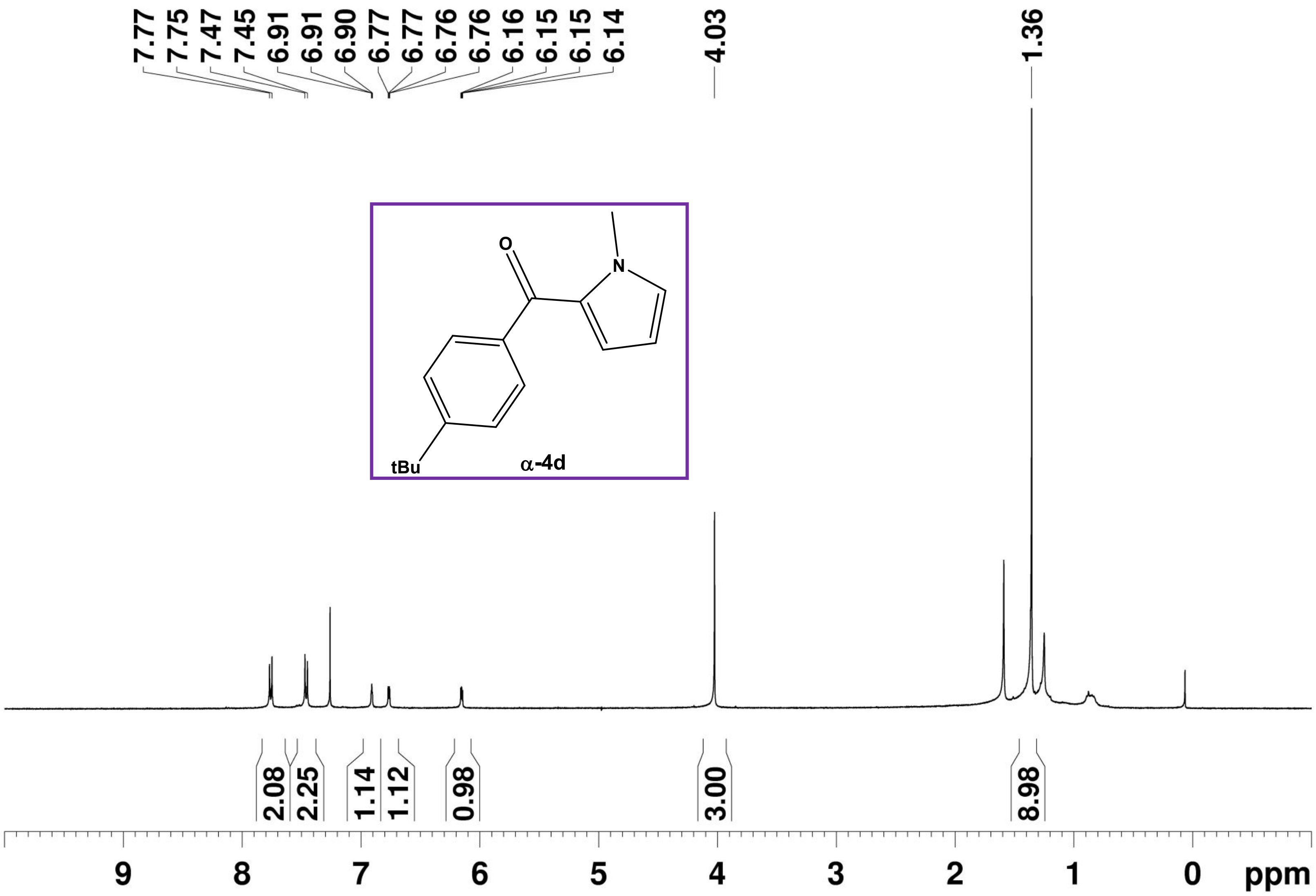

Figure S10: ^{1}H NMR spectrum of derivative ***α*-4d** (600 MHz, $CDCl_3$, 298 K).

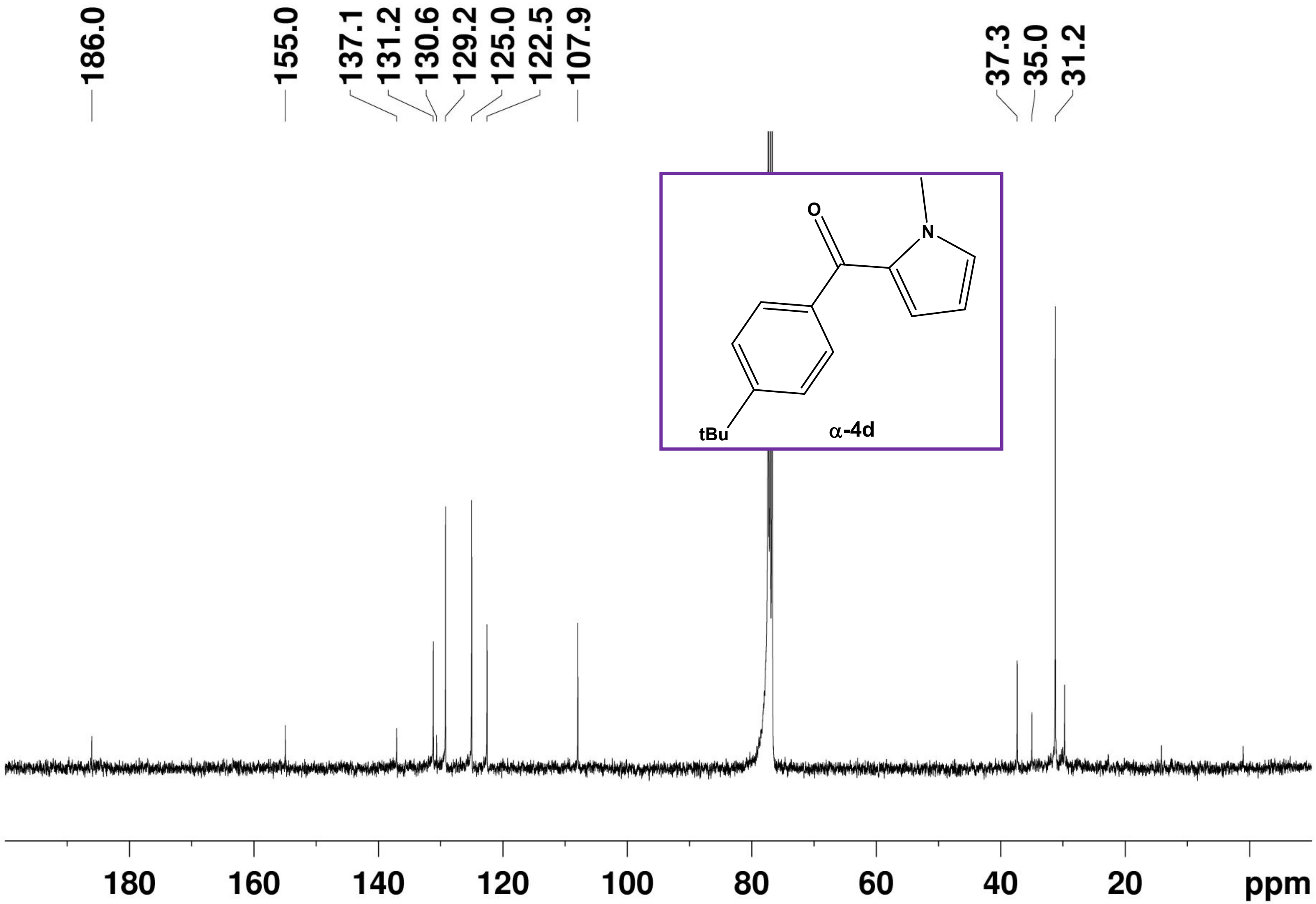

Figure S11. ^{13}C NMR spectrum of derivative *α*-**4d** (150 MHz, $CDCl_3$, 298 K).

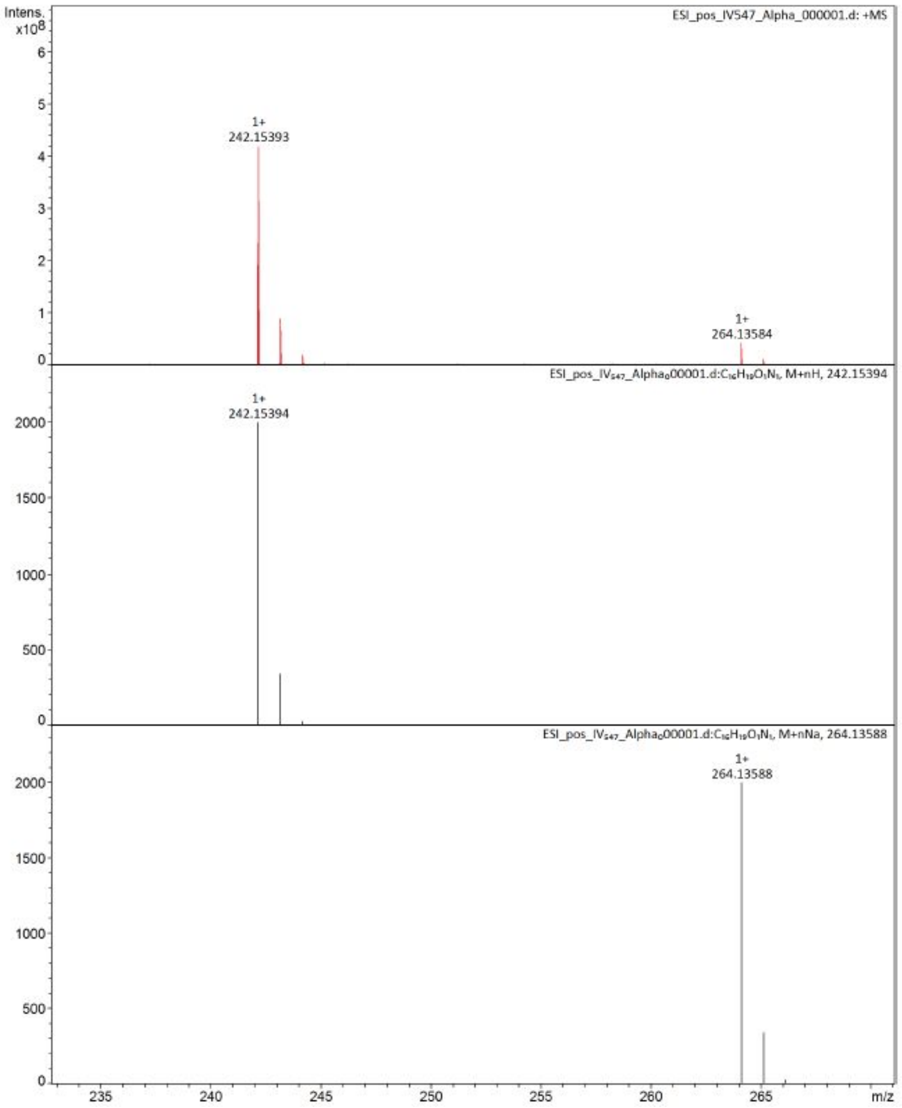

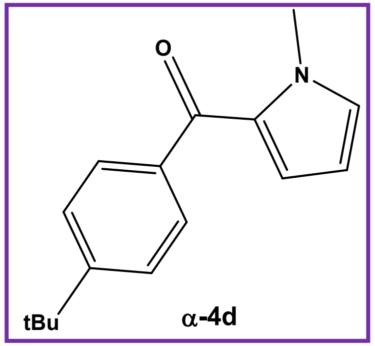

α-**4d**

Figure S12: (top) HR-MS spectrum of derivative α-**4d**; (bottom) HR-MS simulate spectrum of derivative α-**4d;**

m/*z* $[M+H]^+$ calcd for $C_{16}H_{20}NO^+$ 242.1539 found 242.1539; $[M+Na]^+$ calcd for $C_{16}H_{19}NONa^+$ 264.1359 found 264.1358

Derivative *β*-4d

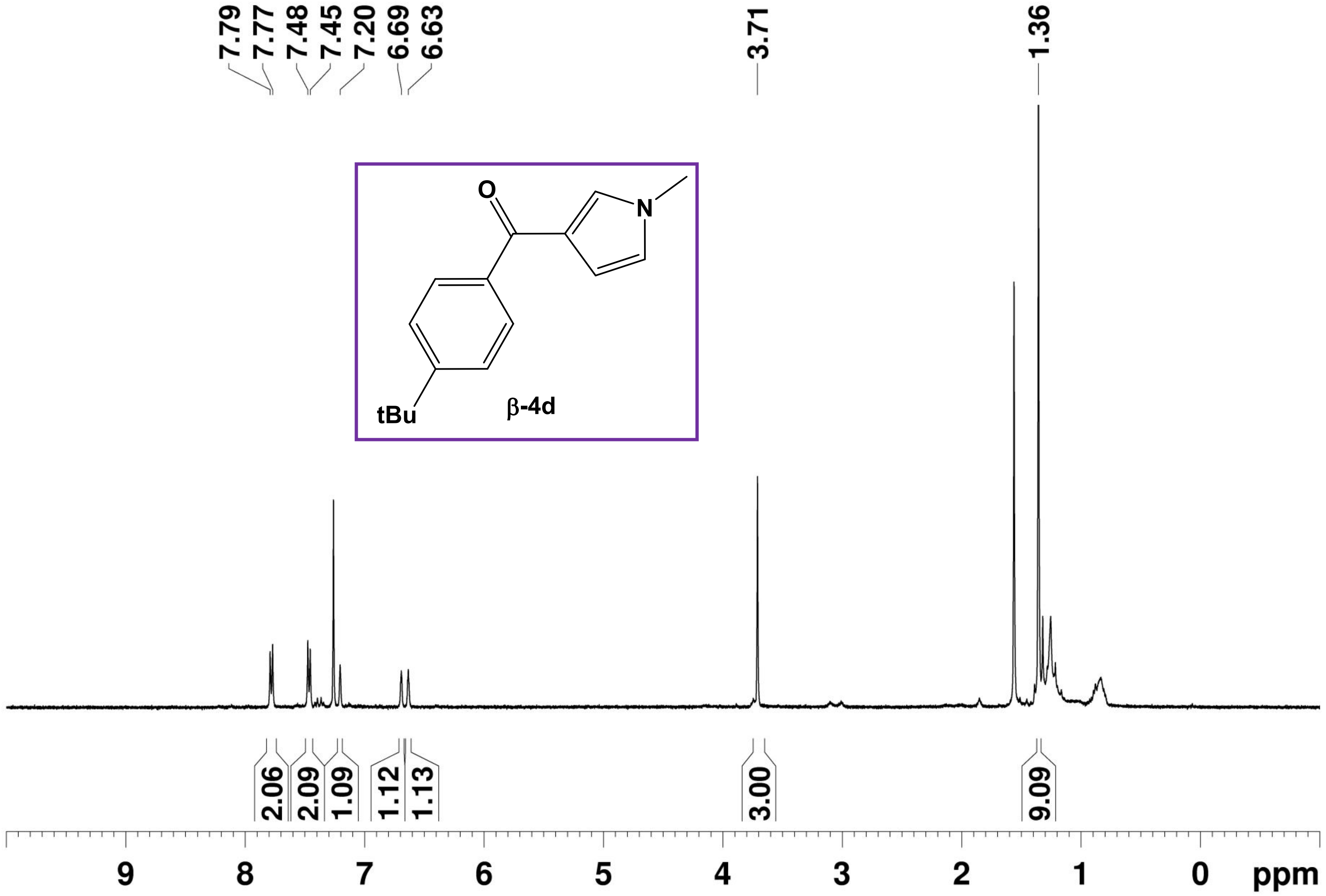

Figure S13: ^{1}H NMR spectrum of derivative ***β*-4d** (400 MHz, $CDCl_3$, 298 K).

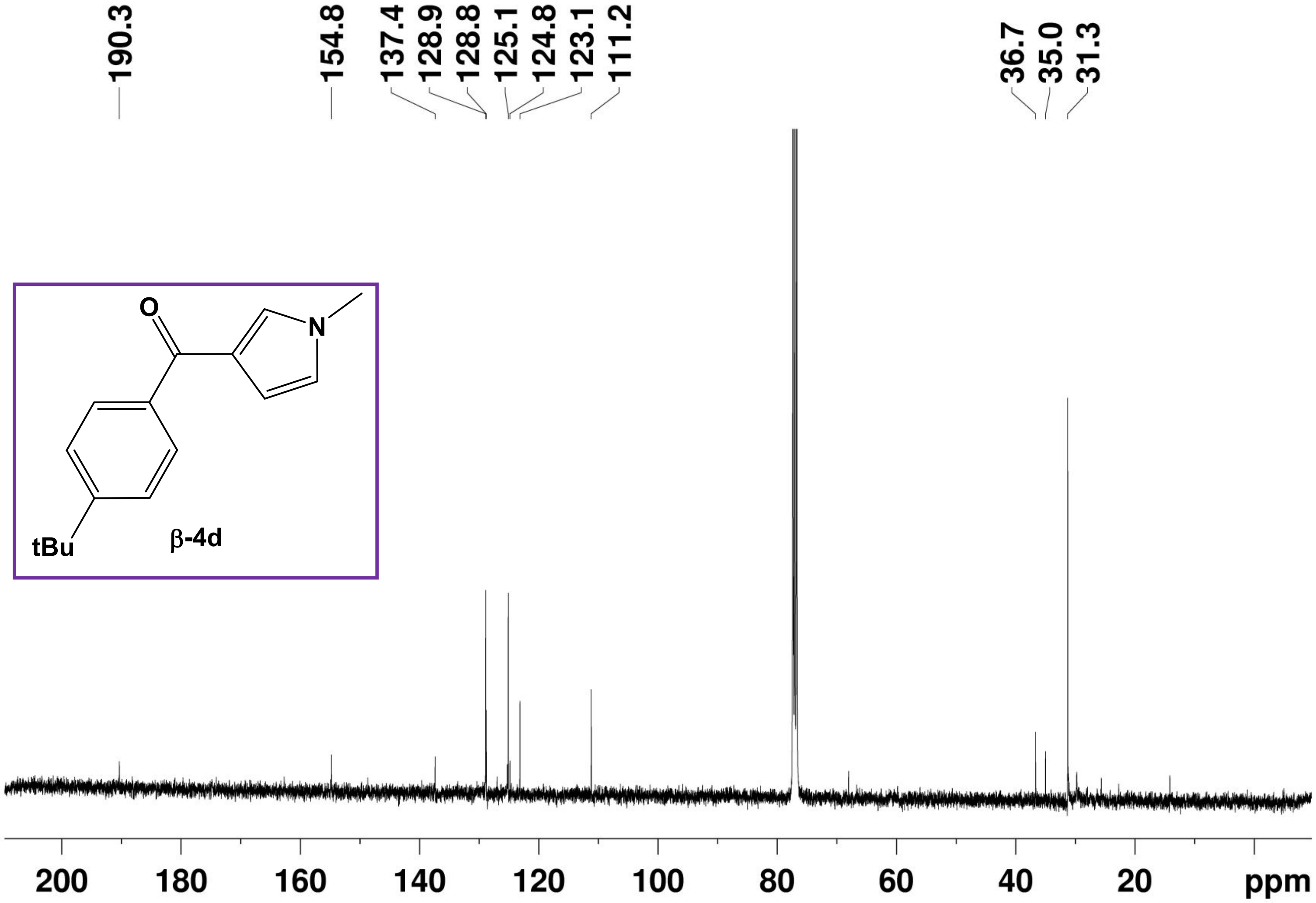

Figure S14: ^{13}C NMR spectrum of derivative ***β*-4d** (100 MHz, $CDCl_3$, 298 K).

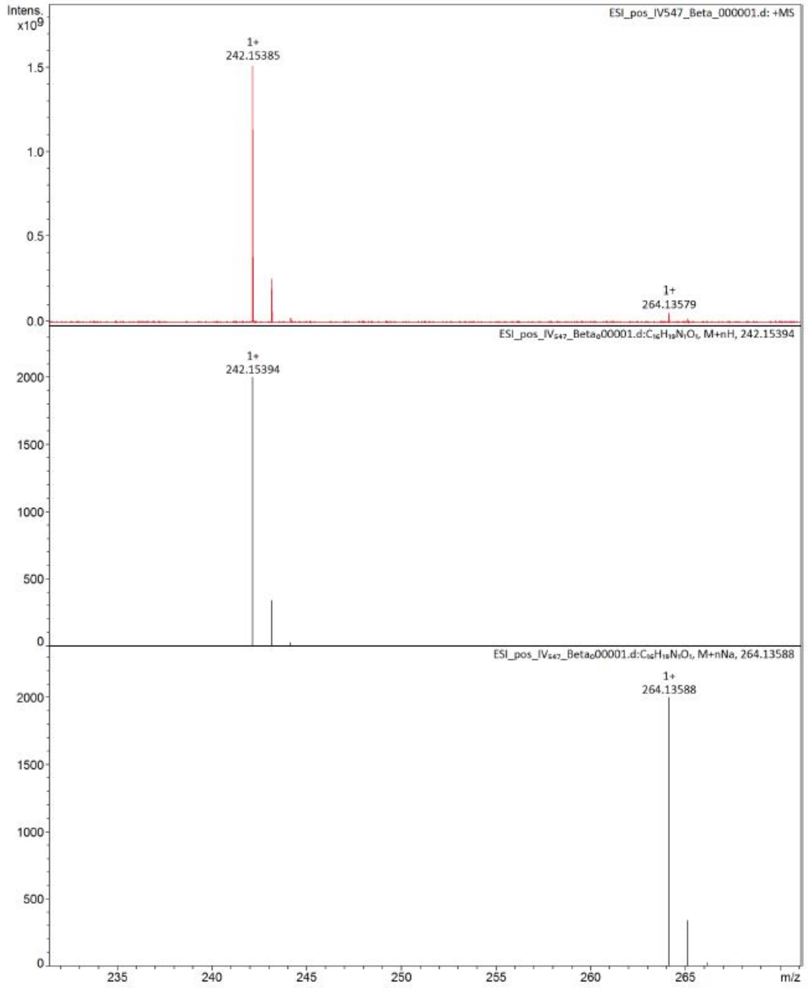

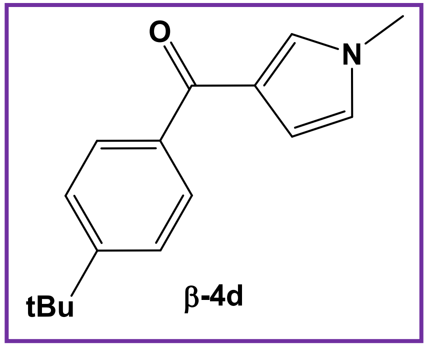

Figure S15: (top) HR-MS spectrum of derivative β-**4d**; (bottom) Simulated HR-MS spectrum of derivative β-**4d;**
m/*z* $[M+H]^+$ calcd for $C_{16}H_{20}NO^+$ 242.1539; found 242.1539; $[M+Na]^+$ calcd for $C_{16}H_{19}NONa^+$ 264.1359 found 264.1358

Derivative α-4e

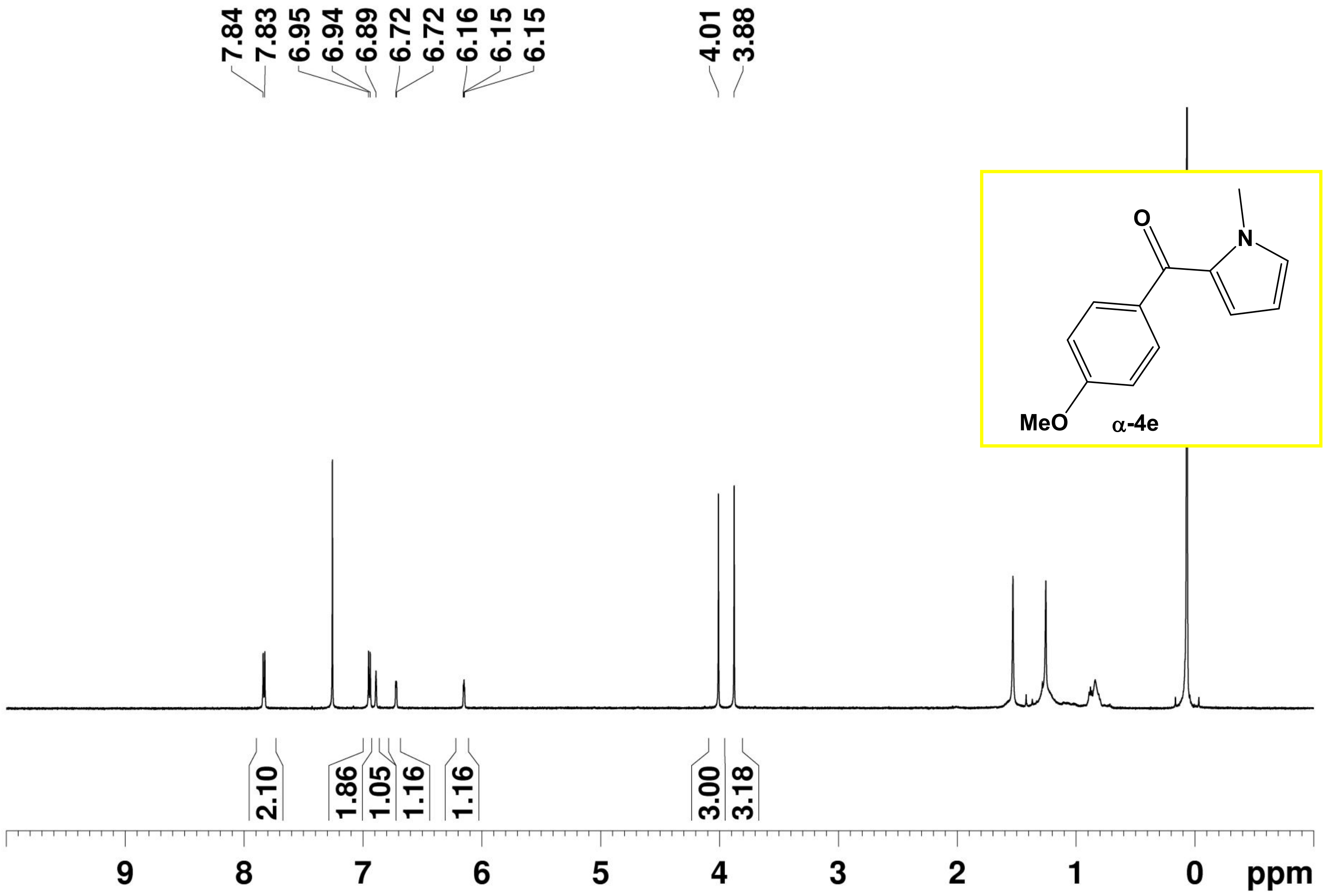

Figure S16: ^{1}H NMR spectrum of derivative α**-4e** (600 MHz, $CDCl_3$, 298 K).

Derivative β-4e

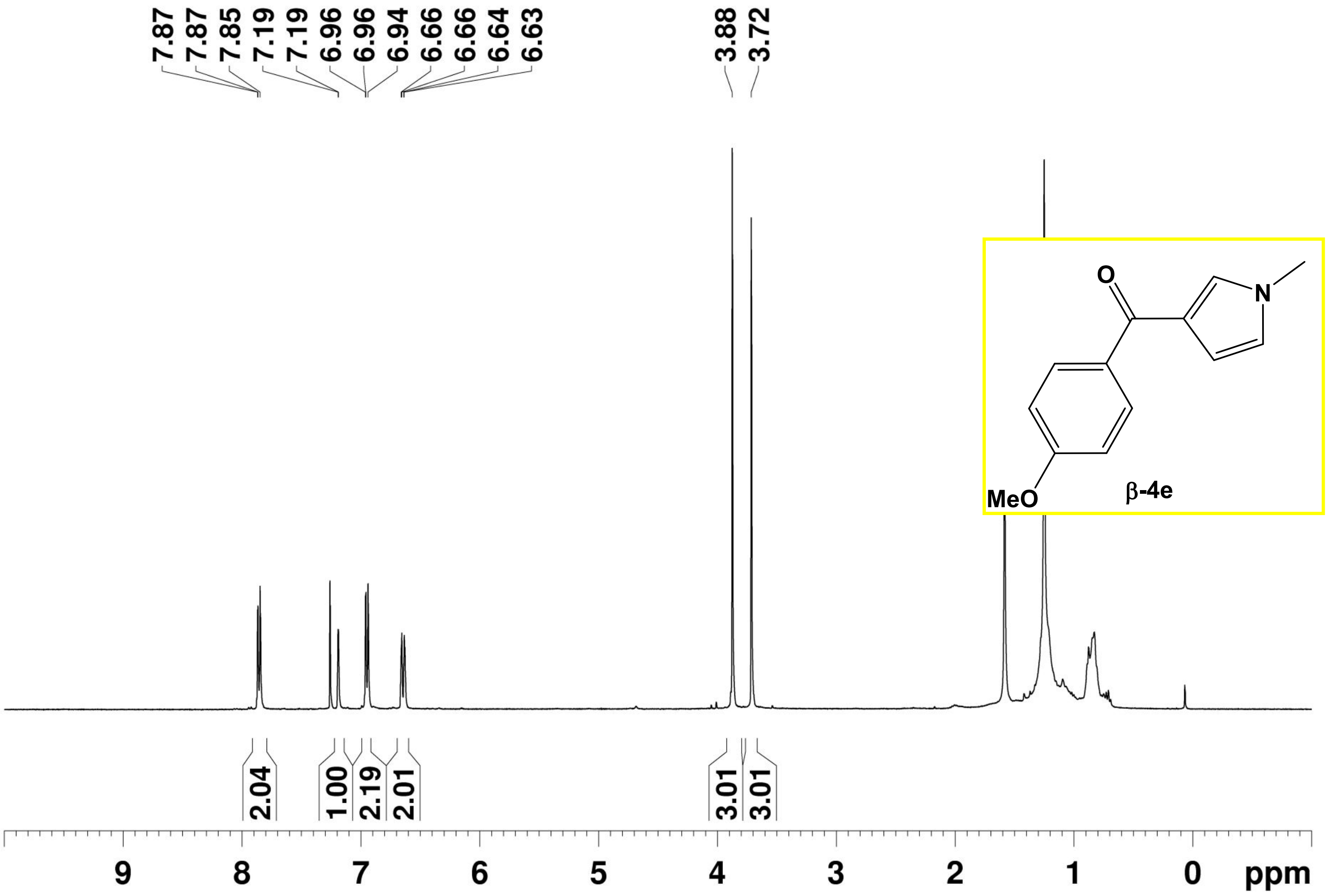

Figure S17: ^{1}H NMR spectrum of derivative ***β*-4e** (400 MHz, $CDCl_3$, 298 K).

Derivative *α*-4f

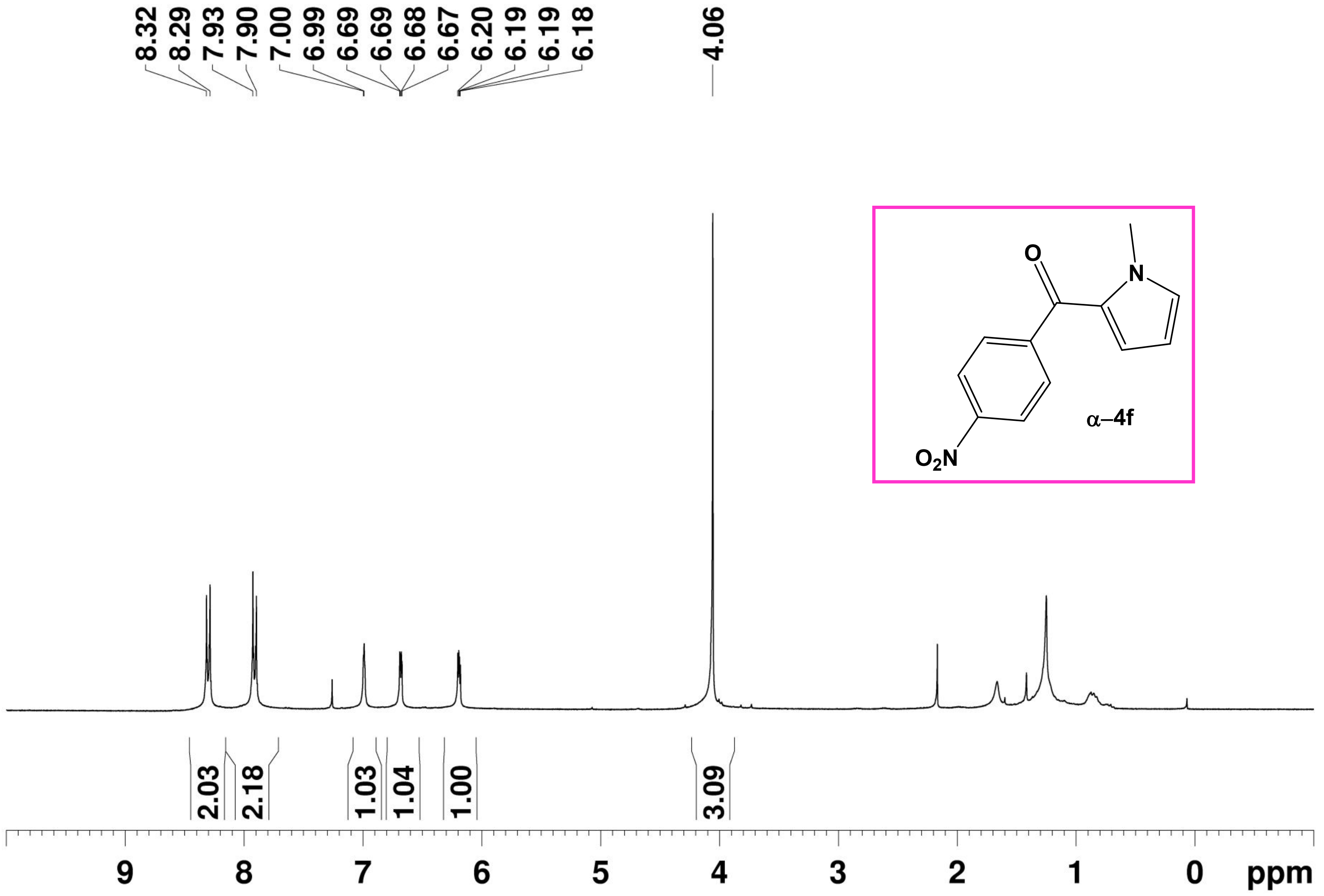

Figure S18: ^{1}H NMR spectrum of derivative ***α*-4f** (300 MHz, $CDCl_3$, 298 K).

4. Encapsulation Proofs of 3a inside the resorcinarene capsule C

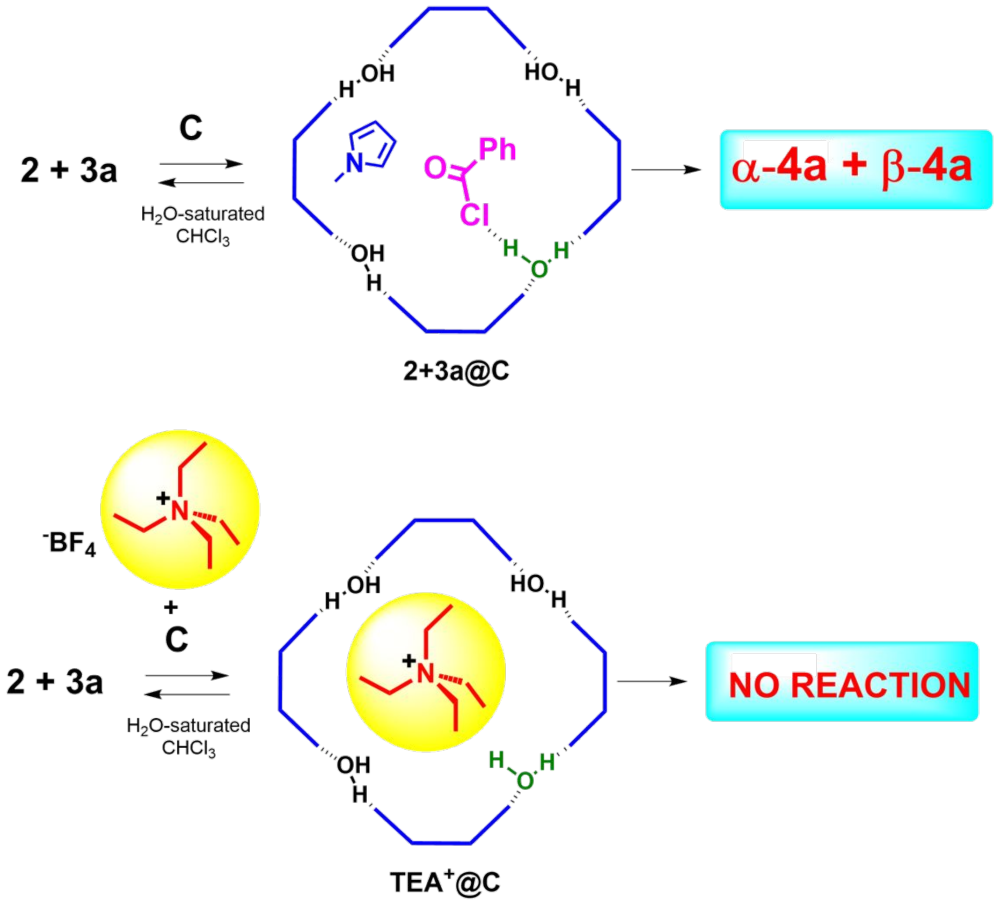

Figure S19. Inhibition experiments with tetraethylammonium tetrafluoroborate.

With the aim to demonstrate that the FC benzoylation of **2** with benzoyl chloride **3a** occurs inside the resorcinarene capsule **C**, the reaction was performed in the presence of the tetraethylammonium cation as BF_4^- salt, as reported in literature the tetraethylammonium guest can occupy the inner cavity of **C**, acting as competitive inhibitor. [1,2]

The inhibition experiment was performed by mixing **2** and **3** under the conditions reported on page S3 in the presence of $Et_4N^+BF_4^-$, (10 equiv respect to the capsule; 0.76 M).[2] By ^{1}H NMR analysis of the crude reaction mixture, no signal attributable to the reaction products *β*-**4a**/*α*-**4a** was detected.

Further indirect proofs of the encapsulation of **3a** inside **C** have been obtained by hydrolysis experiments of **3a** inside the confined space of the hexameric capsule **C**, and by comparison of the same hydrolysis reaction performed in the presence of tetraethylammonium as competitive inhibitor.

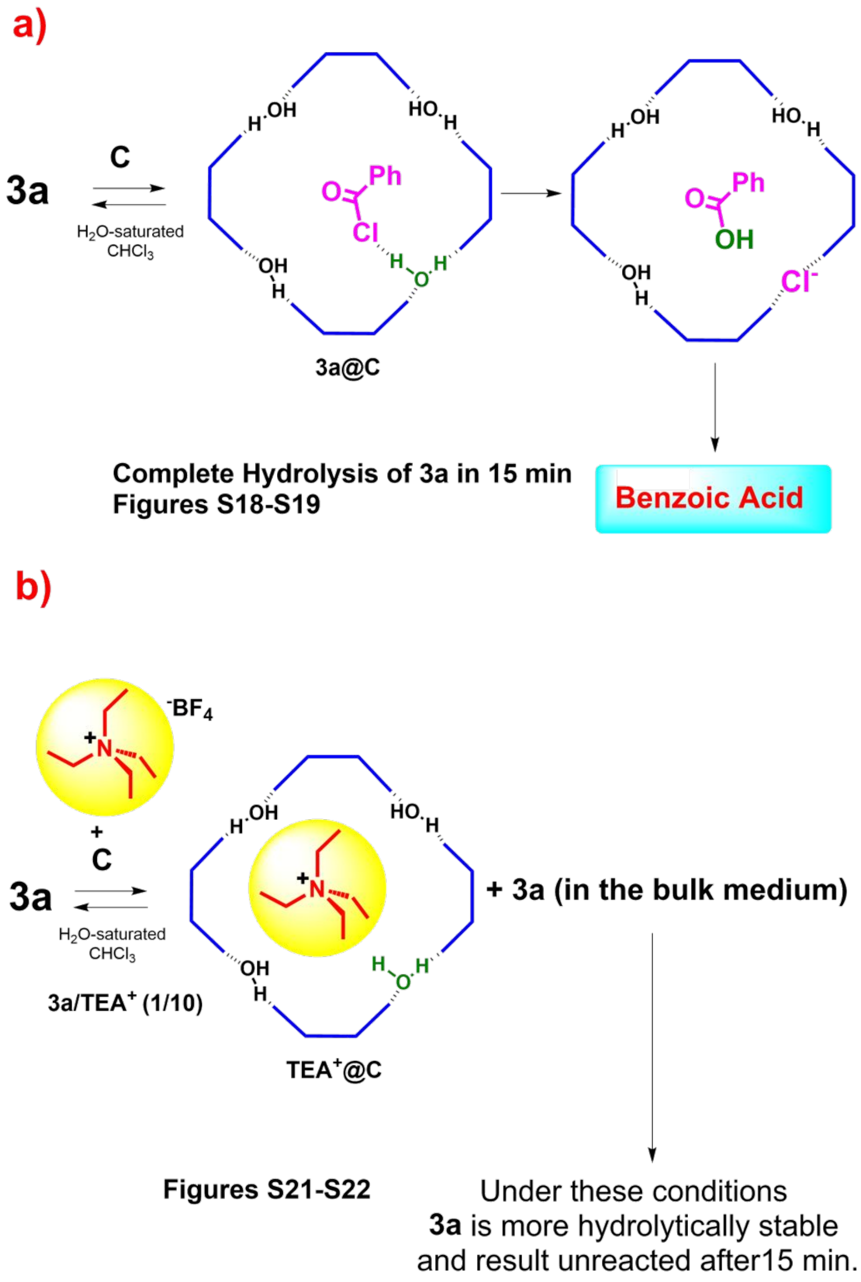

Figure S20. a) Hydrolysis of **3a** to benzoic acid observed inside the confined space of **C.** The conversion to benzoic acid was complete in 15 minutes (Figure S18d). b) *Inhibition experiment*: exploiting the inhibition effect of the competitive guest tetraethylammonium cation, which shows high affinity for the inner cavity of **C**, a negligible conversion of **3a** to benzoic acid was observed after 15 min, because **3a** is hydrolytically more stable in the bulk $CHCl_3$ medium.

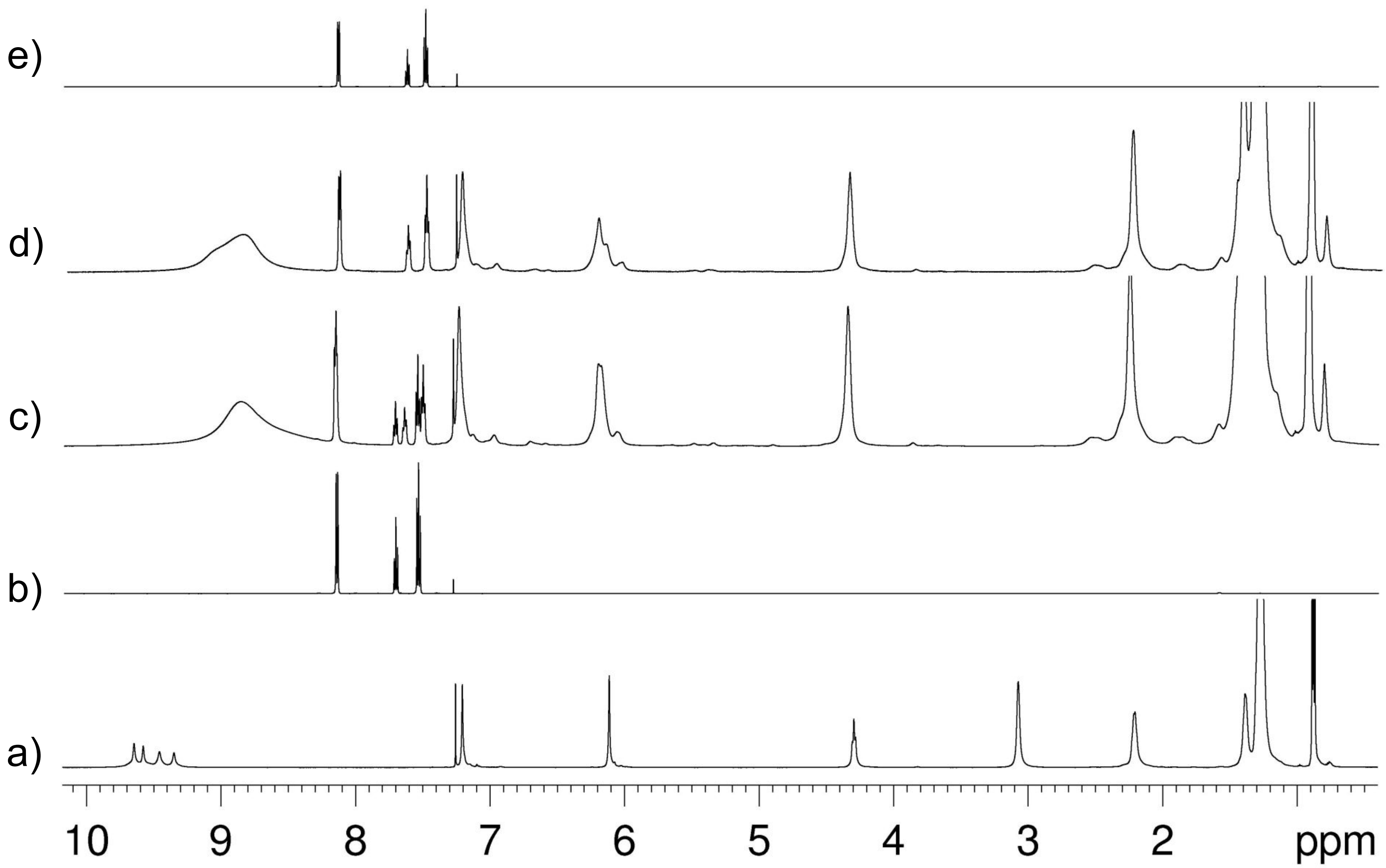

Figure S21. ^{1}H NMR spectra (600 MHz, 298 K, water saturated $CDCl_3$) of: a) **C**; b) benzoyl chloride; c) benzoyl chloride inside **C** (26 %) 10 min after mixing; d) benzoyl chloride inside **C** (26 %) 15 min after mixing; e) benzoic acid.

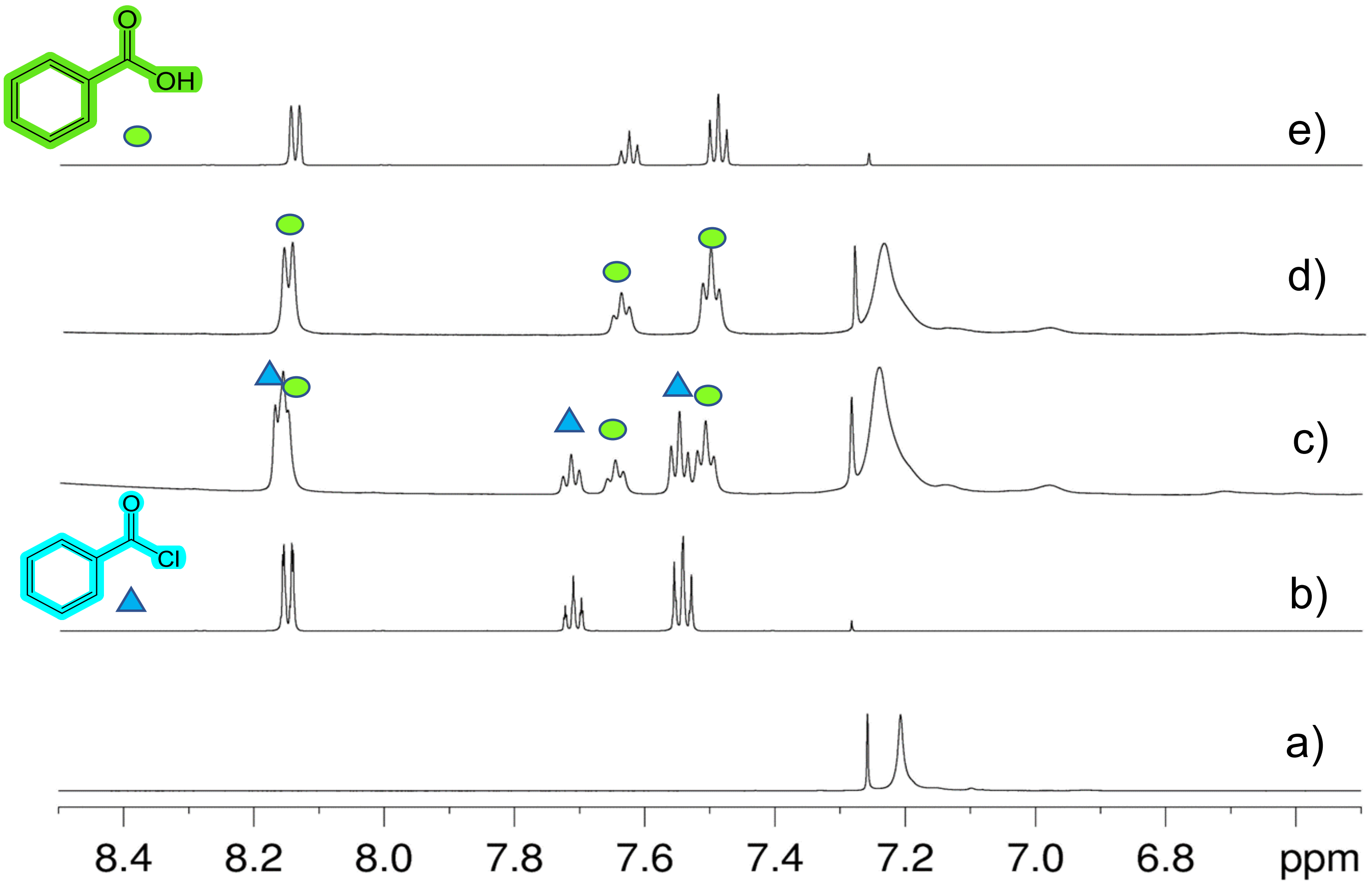

Figure S22. Aromatic regions of the ^{1}H NMR spectra (600 MHz, 298 K, water saturated $CDCl_3$) of: a) Fig. S16a; b) Fig. S16b; c) Fig. S16c; d) Fig. S16d; e) Fig S16e.

c)

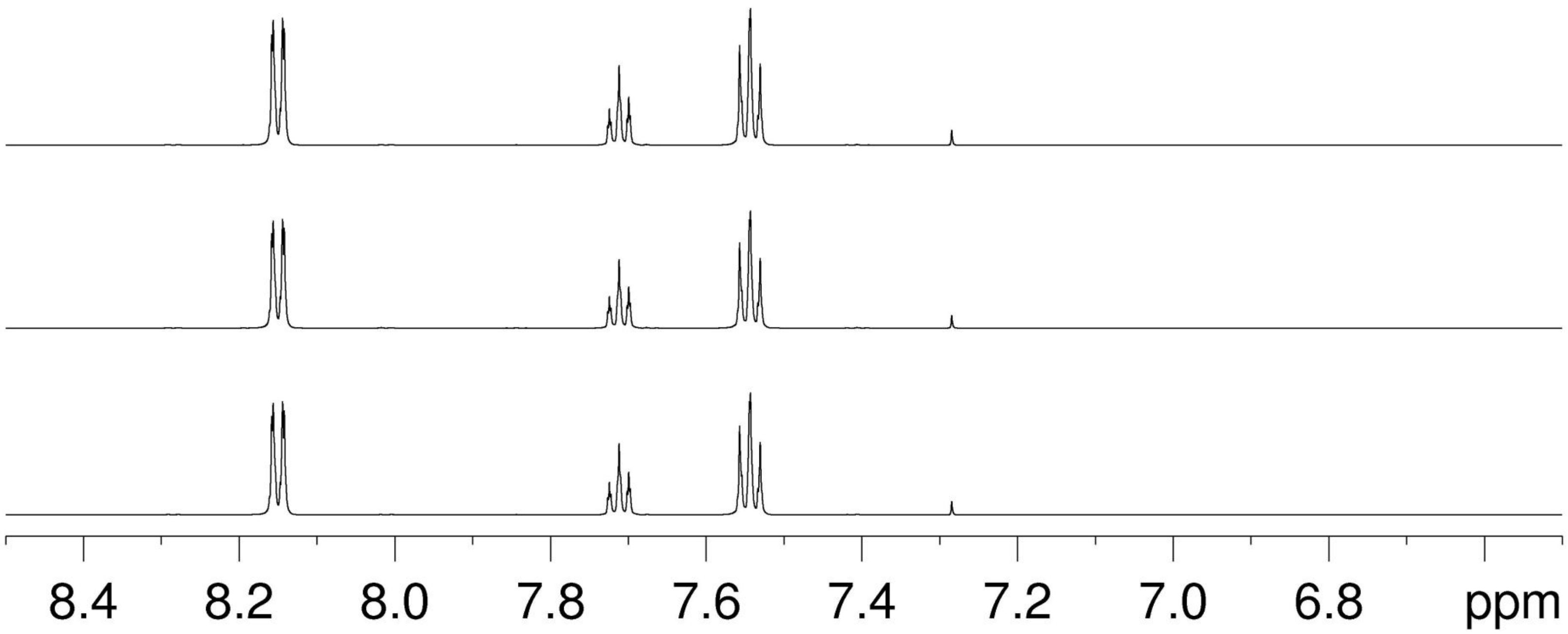

Figure S23. Portions of ^{1}H NMR spectra (600 MHz, 298 K, water saturated $CDCl_3$) of: a) benzoyl chloride; b) benzoyl chloride at room temperature for 12 h, c) benzoyl chloride at 50° C for 12 h.

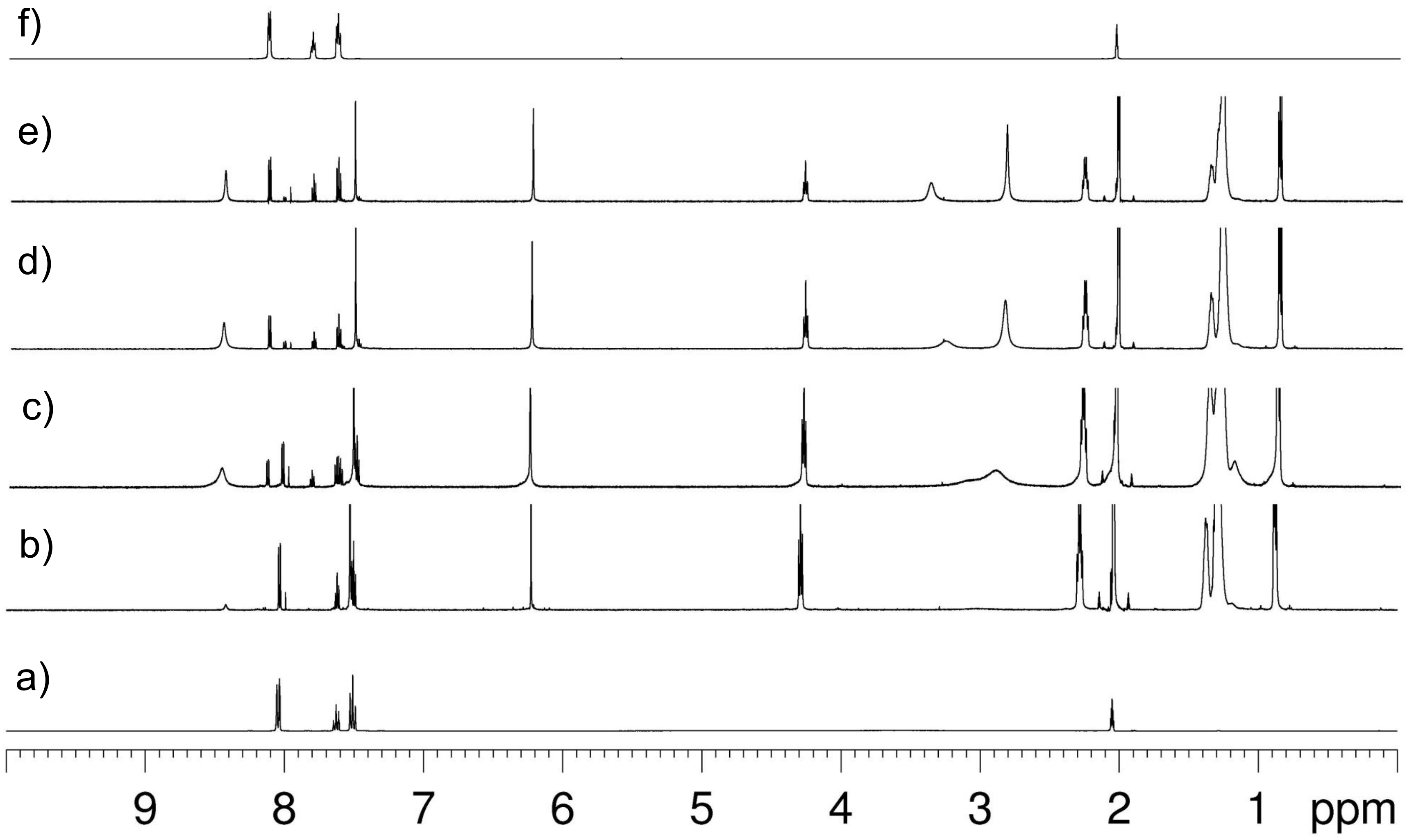

Figure S24. ^{1}H NMR spectra (600 MHz, 298 K, acetone-d6) of: (a) benzoic acid; (b) benzoyl chloride inside **C** (26 %), 15 min after mixing; (c) mixture 1:1 benzoyl chloride/tetraethyammonium BF_4^- inside capsule **C**, 15 min after mixing; (d) mixture 1:4 benzoyl chloride/ tetraethyammonium BF_4^- inside capsule **C** (26 %), 15 min after mixing; (e) mixture 1:10 benzoyl chloride/ tetraethyammonium BF_4^- inside capsule **C** (26 %), 15 min after mixing; (f) benzoyl chloride (600 MHz, Acetone-d6, 298 K).

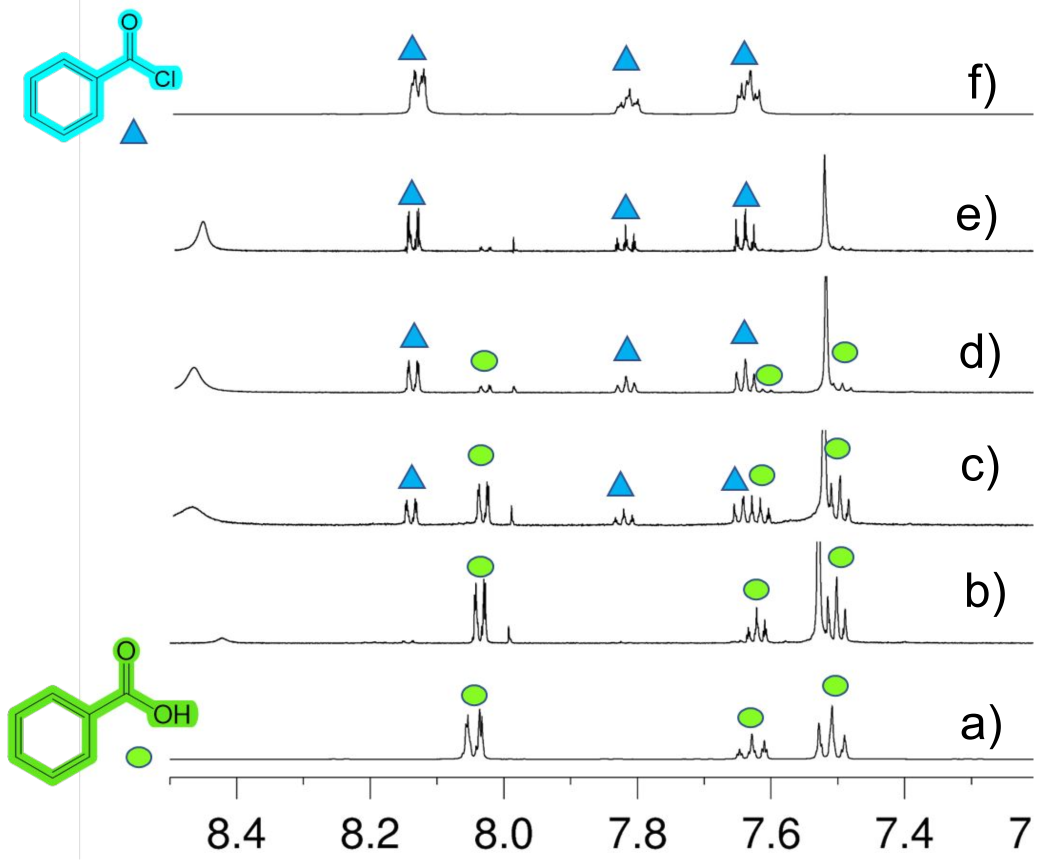

Figure S25. Portions ^{1}H NMR spectra of: (a) Fig S21a; (b) Fig S21b; (c) Fig S21c; (d) Fig S21d; (e) Fig S21e; (f) Fig S21f.

5. Computational Studies

Due to the high computational cost derived from the large number of atoms involved, we choose to conduct an in-silico investigation using the ONIOM method upon a reduced model of **C**, namely $\mathbf{C_R}$ substituting the undecyl residues (the so-called "feet") present in the hexameric capsule with the methyl ones.

The calculations have been performed using the ONIOM method incorporated in the Gaussian 16 package.[7] The reactive species, together with three of the four molecules of water directly involved in the supramolecular assembly and the corresponding phenolic hydroxyl with which it establishes a hydrogen bond, were modeled using the M06-2X DFT functional, employing the cc-pvdz basis set during the optimization and cc-pvtz for single point calculations, while the semiempirical method PM6 was employed for all the other atoms. Continuum Model (PCM) using the integral equation formalism variant (IEFPCM) was used for the solvation (Chloroform) of the transition states.

To determine the activation energy barrier of each step and the reaction energy profile, the reactant complex, transition state, and product complex structures were optimized. All transition structures were characterized by only one imaginary frequency in normal mode analysis and further supported by Intrinsic Reaction Coordinate (IRC) calculations. Thermodynamic corrections were calculated at 298.15 K and 1 atm for the optimized geometries.

Initially, we studied the energies involved in encapsulating the reactant (*i.e.*, **2** and **3a**). The calculated energy stabilization resulted in –5.57 kcal/mol and –5.84 kcal/mol for encapsulating **2** and **3a** inside $\mathbf{C_R}$, respectively. The slightly different calculated energies suggest that the first species to enter the capsule is molecule **3a**, followed by **2**.

The H-bonding donor abilities of the capsular water molecules were then analyzed. QM calculations indicate clearly that the oxygen of the carbonyl group is the one that interacts with the water by the formation of an H-bond (Figure S26). A geometry where the same bond is present between the chlorine and the same water molecule was not found. Interestingly, a different conformation is achieved when the 4-nitro derivative **3f** is inside $\mathbf{C_R}$ (Figure S26). Particularly, **3f** forms two different H-bonds, the first between a water molecule and the oxygen of the carbonyl group and the second one between the opposite water and the nitro group of the aromatic ring. Moreover, when pyrrole **2** is inserted in $\mathbf{C_R}$ with **3a** or **3f**, its *N*-methyl group is allocated inside a resorcinarene ring close to the reactive carbonyl group of **3** (Figure S26). Starting by the [**2**+**3a**]@$\mathbf{C_R}$ molecular complex MC (Figure S26), with a Gibs free energy of 13.31 kcal/mol lower than that of the three separate entities, and obtained the correct geometry with the pyrrole conveniently placed to carry out the addition, the mechanism was investigated.

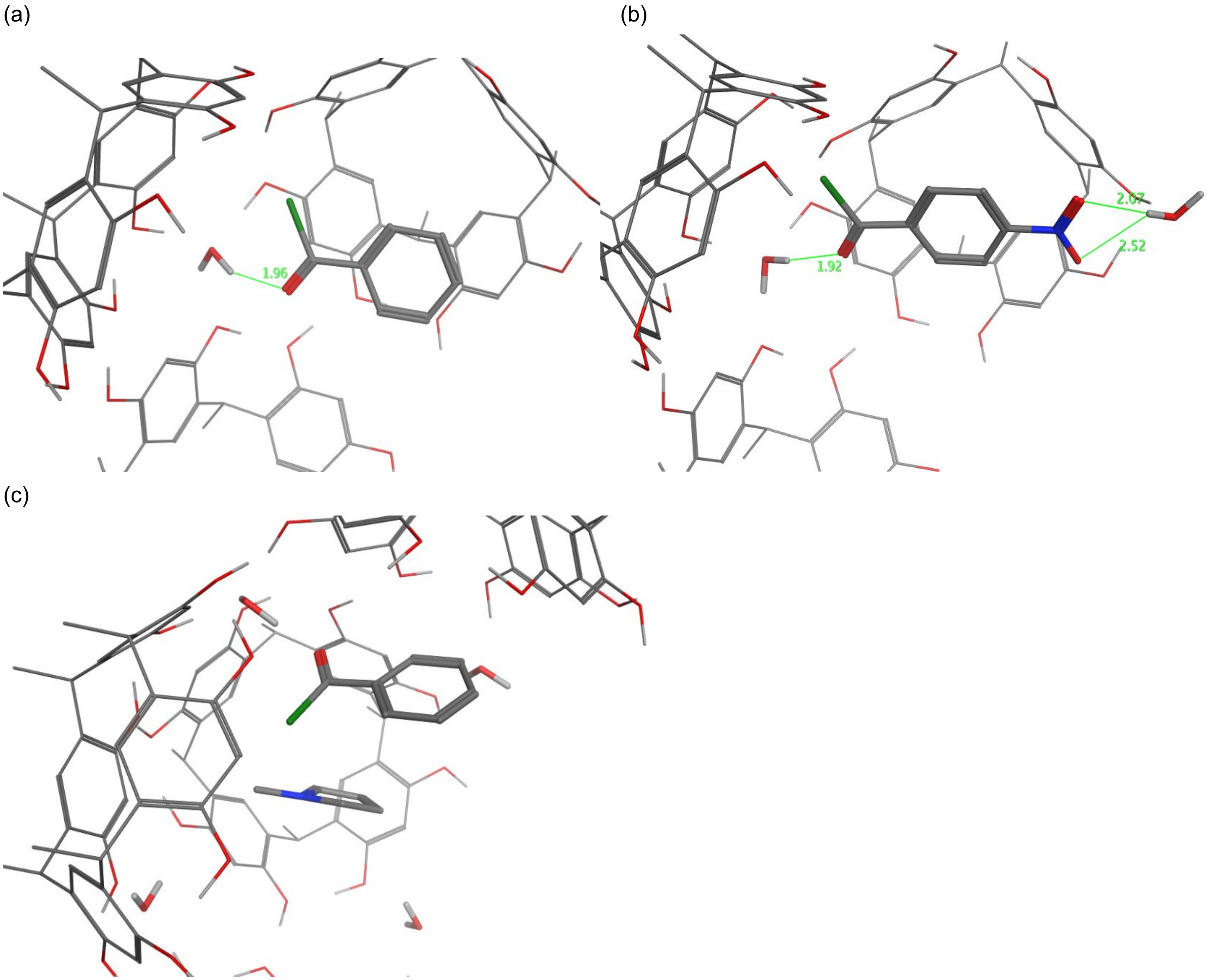

Figure S26. **3a**@C_R (a), **3f**@C_R (a) and [**2**+**3a**]@C_R (c).

The calculations indicate that the reaction may proceed through two paths for *a* and *b* products (Scheme S2). For the first path, an activation energy of 25.29 kcal/mol produces *a*-TS1 (Figure S27, Scheme S2) and leads to the corresponding Wheland intermediate, α-I located 4.60 kcal/mol below TS1 (Figure S27, Scheme S2). Finally, the simple loss of the hydrogen atom from the intermediate proceeds very quickly, thanks to a low energetic barrier of 5.01 kcal/mol (Figure S27, Scheme S2). The obtained product α-**4a** is located 7.87 kcal/mol below the starting reagents making the reaction exergonic (Figure S27, Scheme S2). The path to the production of β-**4a** seems different due to a different geometry of the β-I, where the hydrogen and the Cl^- are opposite to the pyrrole ring, and a direct extraction of the hydrogen seems impossible for the Cl^-. In this path, a first barrier of 25.27 kcal/mol (β-TS1) led to the formation of β-I1 (Figure S27, Scheme S2). Then, the geometry does not allow a direct proton extraction, and a TS for a [1,2]-H shift was calculated (β-TS2$_{shift}$, 17.75 kcal/mol) to produce another intermediate β-I2. The loss of the hydrogen atom from the β-I2 proceeds again very quickly with an energetic barrier of only 1.51 kcal/mol, and the obtained product β-**4a** is located 10.37 kcal/mol below the starting reagents (Figure S27, Scheme S2). Another β-TS2 was calculated after a C–C rotation; in this TS, a direct proton extraction showed a lower activation energy (β-TS2, 4.81 kcal/mol) very similar to the activation energy of α-TS2, suggesting that the reaction may proceed without [1,2]-H shift. Looking at this data, where the two products have a 2.5 kcal/mol difference in energy, and looking at the experimental results, we can conclude that the reaction does not only produce the thermodynamic product. This result was further experimentally proved by the reaction of α-**4a** and $\mathbf{C_R}$, where no conversion to the thermodynamic more stable product β-**4a** was observed.

Moreover, by looking at the experimental results and the energies calculated for the reaction paths, the TS1 is the one that is more likely to influence the formation of the two different products. For **4a**, the two different TS1 are very close in energy, with a slight preference over the β-**4a** and reflecting the 40/60 ratio experimentally observed.

Despite sometimes depending on the Lewis acids and the experimental conditions, electrophilic substitution in pyrrole occurs faster at the 2-position than at the 3-position. The standard explanation for the attack at C-2 is based on the relative energies of the intermediates. This essential valence-bond description is reduced to saying that the conjugated system of the α intermediate is linearly conjugated with the lone pair on the nitrogen atom overlapping with the π system of an allyl cation, whereas the conjugated system of the β intermediate has the lone pair on the nitrogen atom overlapping with the π bond and an isolated cation. Hence the α linearly conjugated system is lower in energy than a cross-conjugated β intermediate. Looking at the same argument, applying the frontier molecular orbital (FMO) theory predicts the C-2 attack. The HOMO of **2** has a node running through the heteroatom with a higher orbital coefficient at the C-2 and an estimate of the C-2 charge lower than C-3. Despite the insertion of the molecule inside the capsule lower the energy of the HOMO, the geometry of the orbitals coefficients is not influenced; hence the reactivity is still very similar with a predicted selectivity of the C-2 over C-3. The different selectivity of the reactions carried out with the different acyl chlorides **3a**–**f** must be controlled by their LUMO. The FMOs of the reactant are resumed in Table S2. As pointed out by the calculated energies, the LUMO energies of the acyl chloride **3** and, therefore, the Δ LUMO(**3**)–HOMO(**2**) energies are ranked as expected: **3f**<**3a**<**3e**. When inserted into the capsule, the acyl chlorides **3** are subjected to different intermolecular forces, but not all are subject to the same. As discussed earlier, molecule **3a** only involves an H-bond with a water molecule. Differently, the substituent to the aromatic ring of **3e** and **3f** allow them to further interact with another water molecule. When into the capsule, the LUMO energies of **3** are all higher. However, due to the different interactions, they do not experience the same increasing magnitude and are ranked as **3f**<**3e**<**3a**. For molecule **3f**, the electron-withdrawing (EW) substituent guarantees a high reactivity even under the H-bond geometry inside the guest, and the sole product formed is still under FMO control. For **3a** and **3e**, an electronic control seems relevant to form the β-product. Both molecules engage an H-bond between a water molecule and the carbonyl group, and **3e** has a similar geometry to that of **3f**, where an additional H-bond is formed. The first H-bond higher the energy of the LUMO, whereas the additional H-bond of **3e** compromise the effect of electron-donating (ED) substituent and lower the LUMO energy at a value lower than **3a**. The overall result is that the β-product is formed for both molecules.

The structure of the TS1 and the reaction mechanism is worth discussing. It is believed that the tetrahedral mechanism is used to carry out nucleophilic replacements at the carbonyl group. The intermediate, which contains both the nucleophile and the leaving group, should result from the nucleophile's attack on the substrate's carbonyl group in step one of the addition-elimination reaction. Nevertheless, there is evidence that bimolecular concerted S_N2-like – appropriately, above-plane nucleophilic vinylic substitution ($S_NV\pi$) – reactions can also occur, mainly when a suitable leaving group exists. Several studies have been proposed to

support the S_N2-type reaction mechanism,[8,9] and this mechanism was proposed to be the one for the hydrolysis of benzoyl chlorides with both EW (4-NO_2) or ED (4-MeO) groups. In this mechanism, TSs are formed directly from the reactants and transformed into products without the intermediacy of any other species. In the proposed mechanism, a network of water molecules (or another water molecule) is believed to participate in the reaction by engaging an H-bond with the leaving chlorine atom. This raises the question, is a similar interaction found in the capsule, considering that the oxygen of the carbonyl group is the one that is interacting with the water by the formation of an H-bond?

Figure S28 reports the structures of α-TS1 and β-TS1 for forming **4a**. Interestingly the distances between the leaving chlorine and the closest resorcinarene-OH are 2.61 and 2.70 Å for the α-TS1 and 2.64 and 2.72 Å for the β-TS1. The closest water molecule is at 4.21 Å from the α-TS1 and 2.41 Å from the β-TS1. This further corroborates the β-regioisomer preferential formation and the lowest calculated energy for the β-TS1.

The TS1 was then calculated for the **4f** product to validate the TS's influence on the reaction behavior. As already said, the geometry of **3f** inside the capsule is different from **3a**, and we were able to find a stationary geometry in which not only the carbonyl group can engage an H-bond with the water but also the NO_2 group forms a supplementary H-bond with another bridging water molecule of the pentameric resorcinarene structure. Still, the three hydrogens of the *N*-methyl group of **2** are symmetrically pointing inside the π-electron-rich cavity of a resorcinarene macrocycle in the complex [**2**+**3f**]@$\mathbf{C_R}$. The geometries of the two TS1 are shown in Figure S28. The free Gibbs activation energy involved in adding the **2** to **3f** can justify the experimentally measured ratio between α-**4f** and β-**4f**. The calculated TS1 energies are 7.35 and 11.57 kcal/mol for the α-**4f**-TS1 and β-**4f**-TS1, respectively. According to our proposed mechanism, the –4.21 kcal/mol of the α-**4f**-TS1 well explains the only formation of the α-**4f** product.

Table S2. Calculated energies for FMOs of **2**, **3a**, **3e**, and **3f**.

Molecule	**HOMO (a.u.)**	**LUMO (a.u.)**
**2**	−0.25566	—
**3a**	—	−0.04126
**3e**	—	−0.02974
**3f**	—	−0.08390
**2@$\mathbf{C_R}$**	−0.32620	—
**3a@$\mathbf{C_R}$**	—	−0.02926
**3e@$\mathbf{C_R}$**	—	−0.03483
**3f@$\mathbf{C_R}$**	—	−0.07729

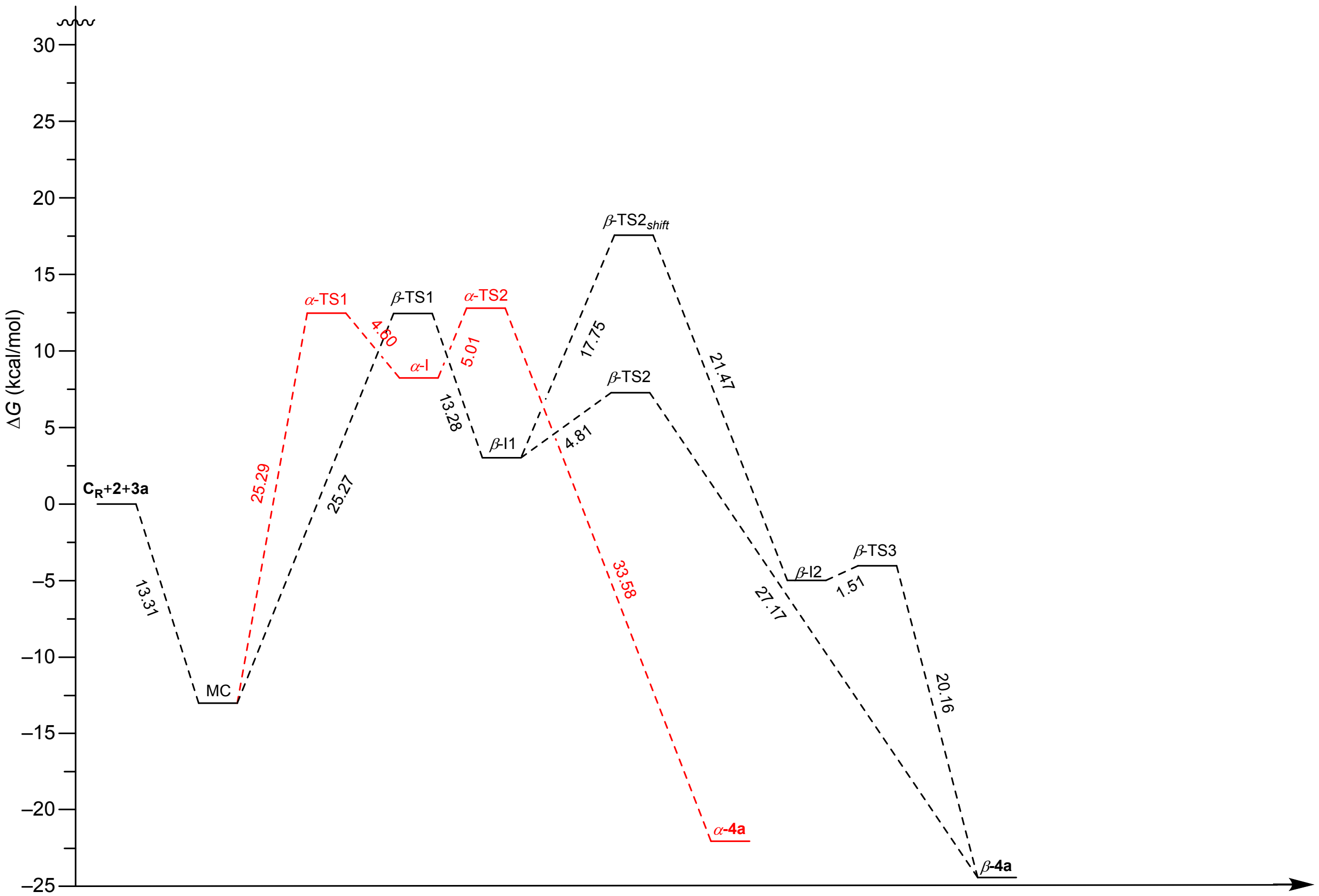

Figure S27. Gibbs free energy profiles for *alpha* (in red) and *beta* (in black) paths within $\mathbf{C_R}$; the term @$\mathbf{C_R}$ has been omitted for clarity.

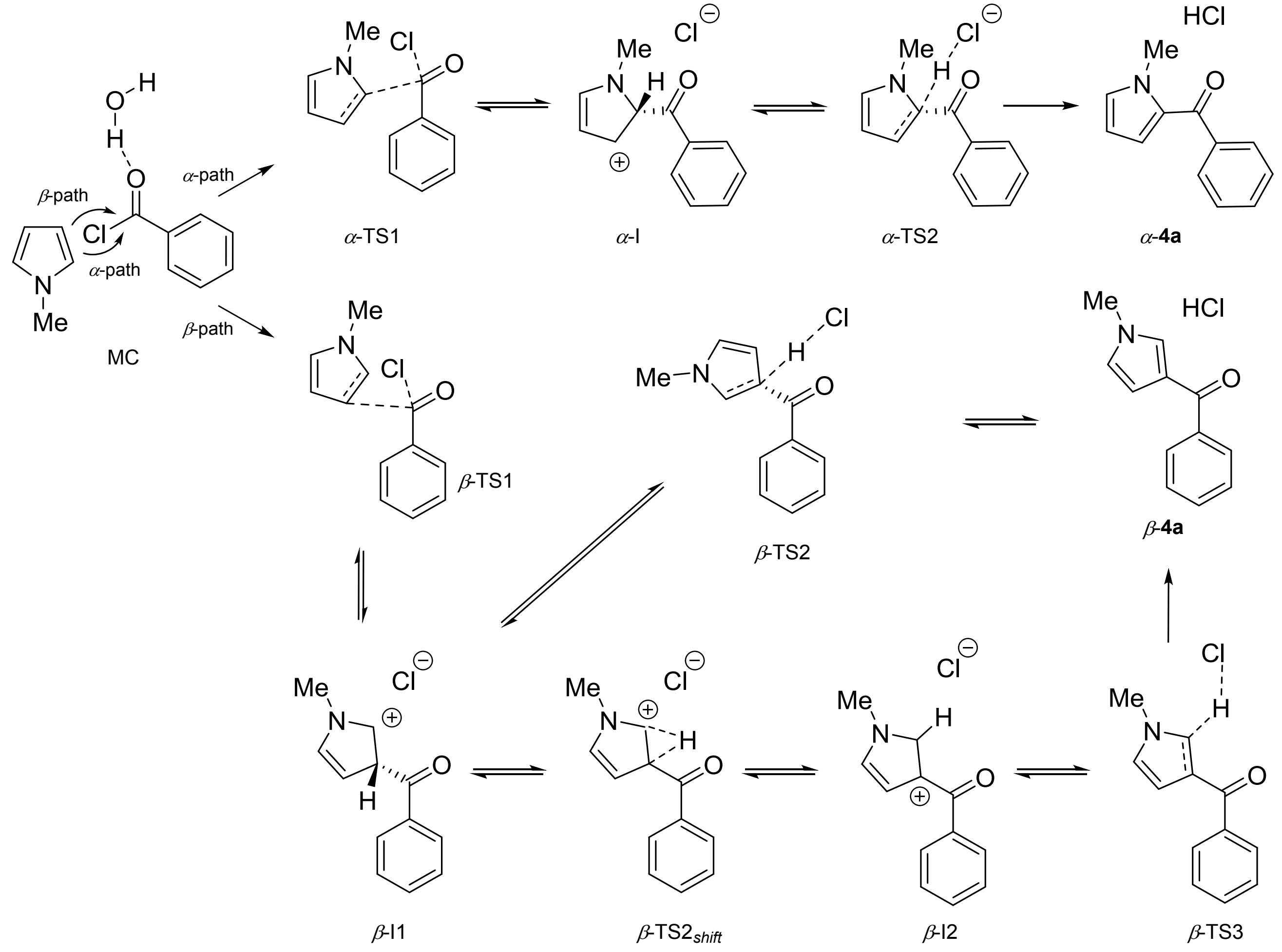

Scheme S2. *Alpha* and *beta* channels for the *in-silico* studies of the FC and nomenclature adopted. The capsule $\mathbf{C_R}$ is always present.

(a) (b) (c) (d)

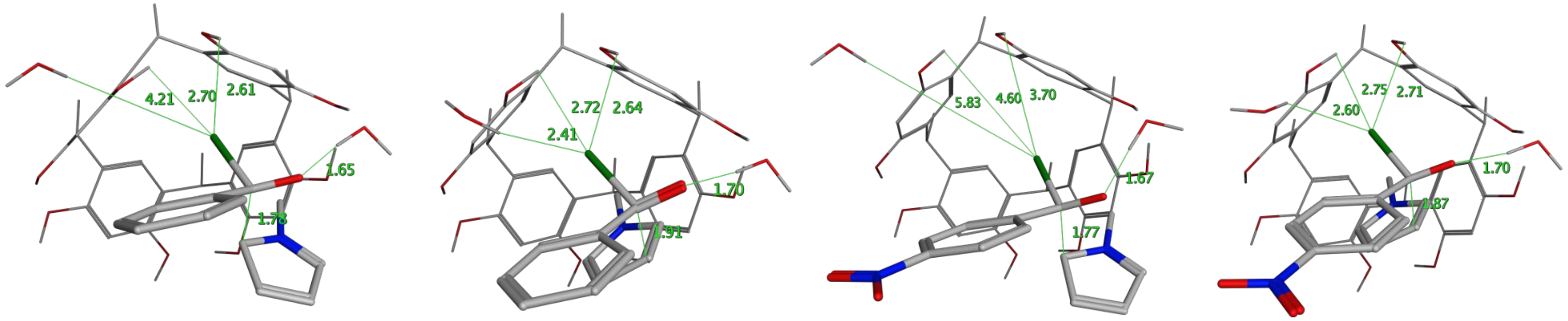

Figure S28. Distances, measured in Å, between the leaving Cl and the closest resorcinarene-OH and capsular water in α-TS1 and β-TS1 of α-**4a** (a) and α-**4f** (c) and β-**4a** (b) and β-**4f** (d).

Computational studies for the reaction

ONIOM(M062X/cc-pvtz:PM6) Hartree

	E(0)	E	H	G	ONIOM Total Energy or EE
**C_R**	-229.688959	-229.434185	-229.433241	-229.979580	-233.018189
**2**	-249.349721	-249.344678	-249.343733	-249.377835	-249.460773
**3a**	-805.076649	-805.069514	-805.068570	-805.109217	-805.177895
**3e**	-919.572279	-919.562849	-919.561905	-919.607755	-919.706561
**3f**	-1009.580788	-1009.571158	-1009.570214	-1009.617453	-1009.684933
**2@C_R**	-479.049102	-478.787805	-478.786861	-479.348529	-482.491080
**3a@C_R**	-1034.778953	-1034.515410	-1034.514466	-1035.079487	-1038.212032
**3e@C_R**	-1149.206263	-1148.940796	-1148.939852	-1149.510470	-1152.690674
**3f@C_R**	-1239.287681	-1239.020449	-1239.019505	-1239.595755	-1242.724675
[**2+3a**]@**C_R**	-1284.140808	-1283.872421	-1283.871477	-1284.445411	-1287.686549
[**2+3e**]@**C_R**	-1398.641852	-1398.370474	-1398.369529	-1398.952147	-1402.220837
[**2+3f**]@**C_R**	-1488.646850	-1488.381215	-1488.380271	-1488.942347	-1492.200854
α-**TS1**	-1284.107884	-1283.842965	-1283.842021	-1284.405101	-1287.652495
α-**I**	-1284.106898	-1283.838550	-1283.837606	-1284.412436	-1287.648921
α-**TS2**	-1284.108122	-1283.845624	-1283.844679	-1284.404437	-1287.645657
α-**4a**	-1284.148576	-1283.878869	-1283.877925	-1284.457956	-1287.689715
β-**TS1**	-1284.099497	-1283.831379	-1283.830435	-1284.405132	-1287.645723
β-**I1**	-1284.118699	-1283.850062	-1283.849118	-1284.426305	-1287.663429
β-**TS2**	-1284.111569	-1283.842719	-1283.841775	-1284.418636	-1287.655490
β-**TS2$_{shift}$**	-1284.092292	-1283.823358	-1283.822414	-1284.398006	-1287.636565
β-**I2**	-1284.126457	-1283.857463	-1283.856519	-1284.432236	-1287.674595
β-**TS3**	-1284.122829	-1283.854929	-1283.853985	-1284.429818	-1287.662767
β-**4a**	-1284.148335	-1283.876771	-1283.875827	-1284.461945	-1287.691731
α-**4f-TS1**	-1488.621297	-1488.351025	-1488.350081	-1488.930626	-1492.167894
β-**4f-TS1**	-1488.618607	-1488.349815	-1488.348870	-1488.923904	-1492.166226

(0)= EE + Zero−point Energy
E= EE + Thermal Energy Correction
H= EE + Thermal Enthalpy Correction
G= EE + Thermal Free Energy Correction
EE= Electronic Energy
[a] ONIOM(M062X/DGTZVP:PM6); Chloroform.

Cartesian coordinates for selected fully optimized compounds and transition states

2@C$_R$

Center Number	Atomic Number	Integrated Forces (Hartrees/Bohr) X	Y	Z
1	6	-0.000456724	0.001375963	-0.001171557
2	6	0.000377044	0.001818506	0.000969137
3	7	0.002308024	0.000331025	0.003549617
4	6	-0.000810520	-0.000451900	0.002156017
5	6	-0.001629965	-0.001390208	0.000081075
6	6	-0.003514814	-0.000028574	-0.004386083
7	1	-0.003105425	-0.004688666	-0.003434345
8	1	-0.000346596	-0.005300361	0.004309604
9	1	0.002916650	0.006048236	0.000152893
10	1	-0.000991664	0.002524277	-0.006245716
11	1	-0.005421142	0.002974156	0.003127954
12	1	0.005176614	0.003425462	-0.000538198
13	1	0.001949772	-0.005420544	0.002887544
14	8	0.000449584	0.000288998	0.000048687
15	8	0.003400447	0.007324576	-0.000138108
16	6	-0.000685855	-0.000589799	-0.000206917
17	6	0.000327300	-0.000014368	-0.000297279
18	6	-0.000662606	-0.004279851	0.001450640
19	6	0.000273820	0.000182093	0.000342763
20	6	0.000022220	-0.000045318	0.000342469
21	6	0.000310971	0.000197925	0.000575023
22	6	-0.000030350	-0.000417693	-0.000293092
23	6	0.000140935	0.000393627	0.001230472
24	1	0.000050338	-0.000014366	0.000072203
25	1	-0.000394642	-0.000171360	-0.000867668
26	1	-0.000131056	-0.000109192	0.000056719
27	1	-0.000067936	-0.000241803	-0.000370664
28	1	-0.000137405	0.000072374	-0.000476972
29	1	0.000099955	-0.000096477	-0.000305615
30	8	-0.000641676	-0.000478479	-0.000010702
31	8	0.001527272	-0.001829921	-0.001369248
32	6	0.000779818	0.000825119	-0.000339133
33	6	0.000419959	-0.000930168	-0.000362323
34	6	-0.000491336	0.001636527	0.000417340
35	6	0.000145348	-0.000390955	0.000253457
36	6	-0.000179865	0.000370410	-0.000057725
37	6	-0.000573644	-0.000024204	0.000431271
38	6	0.000013919	0.000050086	-0.000509630
39	6	-0.000588520	0.000284577	0.001016254
40	1	-0.000270368	0.000340815	0.000119357
41	1	0.000628216	-0.000145138	-0.000367876
42	1	0.000035913	0.000151093	0.000021199
43	1	0.000312958	-0.000255154	-0.000260611
44	1	0.000074457	-0.000087759	-0.000303455
45	1	0.000211557	0.000027974	-0.000292415
46	8	-0.000513508	0.000471383	0.000179236
47	8	-0.000499582	-0.000054243	0.000282126
48	6	0.000365700	-0.000088095	0.000338637
49	6	-0.000237086	-0.000110888	-0.000537739
50	6	0.000972193	0.000036039	-0.000311776

51	6	-0.000422056	0.000043362	0.000646993
52	6	0.000140686	-0.000032927	0.000272584
53	6	-0.000117210	0.000139631	0.000149621
54	6	0.000021166	-0.000299350	-0.000313520
55	6	-0.000797785	0.000214500	0.000973064
56	1	-0.000028771	0.000018407	0.000279686
57	1	0.000276171	0.000131417	-0.000753586
58	1	0.000148605	-0.000012809	0.000065689
59	1	0.000249045	-0.000174624	-0.000262421
60	1	0.000137351	-0.000043230	-0.000332692
61	1	0.000374584	0.000037503	-0.000284951
62	8	0.001316841	-0.000687241	0.001044468
63	8	0.000215680	0.000155627	0.000618501
64	6	-0.000839004	0.000645363	-0.000525918
65	6	0.000312420	-0.000581071	0.000374760
66	6	-0.000503205	-0.000351819	-0.000920138
67	6	0.000117618	0.000416473	0.000912164
68	6	-0.000301702	0.000218468	-0.000067434
69	6	0.000113539	-0.000097849	0.000453353
70	6	0.000196031	0.000152064	-0.000452275
71	6	0.000141845	0.000419627	0.001283363
72	1	0.000039914	0.000405932	-0.000072197
73	1	0.000181316	-0.000618206	-0.000610118
74	1	-0.000144826	0.000050032	-0.000031986
75	1	0.000049313	-0.000140707	-0.000316292
76	1	-0.000193462	-0.000260113	-0.000392211
77	1	-0.000024085	-0.000023238	-0.000438234
78	8	-0.000350803	0.000111638	0.000146227
79	8	-0.000405247	0.002554921	0.001675161
80	6	0.000625504	-0.000212274	0.000447965
81	6	-0.000224339	0.001115525	0.000491861
82	6	0.000673937	-0.001867479	-0.000434025
83	6	0.000065471	0.000576255	-0.000292022
84	6	0.000129344	-0.000493945	0.000072047
85	6	-0.000216224	-0.000188150	-0.000593876
86	6	-0.000113959	-0.000028836	0.000520177
87	6	0.000087563	-0.000485066	-0.001163200
88	1	-0.000049237	-0.000504142	-0.000269328
89	1	0.000204508	0.000538436	0.000601998
90	1	0.000105476	-0.000105957	-0.000011729
91	1	0.000054901	0.000075419	0.000369303
92	1	-0.000015303	0.000371426	0.000319727
93	1	-0.000097279	0.000078989	0.000302675
94	8	0.000282913	0.000069590	0.000269598
95	8	-0.000698499	-0.001260792	0.003054702
96	6	-0.000692809	0.000132409	-0.000079541
97	6	-0.000001612	-0.000560487	0.000868870
98	6	-0.000277857	0.001146730	-0.001778364
99	6	0.000124499	-0.000461996	0.000027630
100	6	0.000009905	0.000343076	-0.000154739
101	6	0.000379025	-0.000190501	-0.000413797
102	6	-0.000125808	0.000348921	0.000371374
103	6	0.000591138	-0.000275746	-0.001122127
104	1	0.000160623	0.000348596	-0.000322756
105	1	-0.000499647	-0.000075275	0.000578758
106	1	-0.000104900	0.000065196	-0.000120378
107	1	-0.000074861	0.000116271	0.000341355
108	1	-0.000277730	0.000163225	0.000286494

109	1	-0.000240284	-0.000130386	0.000403082
110	8	-0.000800805	-0.001955310	0.000147775
111	8	0.000206347	-0.000014573	-0.000186920
112	6	0.000493253	0.001313562	-0.000227746
113	6	0.000089763	-0.000837316	0.000293350
114	6	0.000140525	0.000506223	0.000555249
115	6	-0.000045757	-0.000291408	-0.000921906
116	6	0.000124422	0.000207820	-0.000191757
117	6	0.000052147	-0.000397668	-0.000374800
118	6	-0.000292613	0.000158765	0.000342554
119	6	-0.000049241	-0.000559683	-0.001242724
120	1	-0.000189858	0.000256441	-0.000265140
121	1	0.000041177	-0.000103593	0.000891696
122	1	0.000075075	0.000084938	-0.000055469
123	1	0.000225214	0.000168201	0.000428717
124	1	-0.000030151	0.000277502	0.000309555
125	1	-0.000054769	0.000091092	0.000395993
126	8	0.001098936	0.001695651	-0.000392975
127	8	0.000183180	-0.000454297	-0.000303424
128	6	-0.000640065	-0.001407493	0.000301348
129	6	0.000207133	0.000642407	-0.000348990
130	6	-0.000661320	0.000168803	0.000500695
131	6	0.000543475	-0.000229614	-0.000891944
132	6	-0.000042257	-0.000363315	0.000030291
133	6	0.000196661	0.000109142	-0.000445951
134	6	0.000039838	0.000076832	0.000496161
135	6	0.000679520	-0.000205189	-0.001150850
136	1	0.000018969	-0.000200617	0.000133496
137	1	-0.000464953	0.000490476	0.000587161
138	1	-0.000021485	-0.000084689	0.000038481
139	1	-0.000150190	-0.000024136	0.000391244
140	1	-0.000126027	0.000107351	0.000322832
141	1	-0.000421641	0.000079827	0.000301180
142	8	-0.000217544	0.000061991	-0.001730250
143	1	0.000046603	-0.000332882	0.000622668
144	1	-0.000243419	0.000395582	0.000709231
145	8	-0.000542441	0.000104471	0.000339561
146	1	-0.000266634	0.000997640	-0.000383345
147	1	-0.000890270	0.000146009	0.000438120
148	8	-0.004464231	-0.000170432	0.007035803
149	1	0.003270970	0.002614115	-0.004527233
150	1	0.003094793	-0.002795370	-0.002894455
151	8	-0.000153972	-0.000561856	-0.000166115
152	1	0.000335269	-0.000883426	0.000217984
153	1	-0.000567781	-0.000462813	-0.000724447
154	8	-0.004317448	0.003934724	-0.008242347
155	1	0.002373492	-0.004118231	0.004245438
156	1	0.002833652	-0.000384543	0.005741931
157	1	0.000086856	0.000693626	-0.000869706
158	1	0.000630828	-0.000071381	0.000004292
159	1	0.000053817	0.000409619	0.000136133
160	1	0.000183101	-0.000321497	-0.000533795
161	1	-0.000284383	0.000051084	-0.000347870
162	8	0.000289570	-0.000326890	0.000336427
163	8	0.002906940	-0.000635413	0.000843501
164	6	-0.000298650	-0.000390298	-0.000494242
165	6	0.001289984	-0.000518062	0.000395235
166	6	-0.002909523	0.000682090	-0.001037524

167	6	0.000958390	-0.000113652	-0.000034256
168	6	-0.000667918	-0.000137407	-0.000262902
169	6	0.000095867	0.000880043	0.000187066
170	6	0.000009205	-0.000376553	0.000360043
171	6	-0.000485835	0.000998597	-0.000522195
172	1	-0.000505752	0.000271025	-0.000097141
173	1	0.000416745	-0.000661660	-0.000061918
174	1	-0.000045325	0.000074548	0.000121010
175	1	0.000054537	-0.000329142	0.000064086
176	1	0.000358734	-0.000309990	0.000096092
177	1	0.000111962	-0.000276630	0.000258250
178	8	0.000780444	-0.000634046	0.001373971
179	8	-0.000042985	-0.000132039	-0.000356406
180	6	-0.000786031	0.000205247	-0.000989541
181	6	0.000632242	-0.000299102	0.000451166
182	6	-0.000619112	-0.000352681	0.000301497
183	6	0.000227112	0.000850413	-0.000374914
184	6	-0.000182367	-0.000181607	-0.000455061
185	6	0.000249781	0.000182744	0.000270861
186	6	0.000307480	-0.000471848	-0.000080189
187	6	-0.000042379	0.001331715	-0.000412367
188	1	-0.000143565	0.000132480	-0.000236690
189	1	-0.000218385	-0.000575094	0.000692352
190	1	0.000011911	-0.000039116	-0.000091546
191	1	0.000066913	-0.000446670	0.000039228
192	1	0.000118203	-0.000299025	0.000203164
193	1	-0.000236153	-0.000422088	0.000198801
194	8	0.000536654	-0.001023374	-0.000761530
195	8	-0.000599745	-0.000007413	0.000958351
196	6	-0.000531392	0.000365993	0.000355089
197	6	0.000250559	-0.000149338	0.000097795
198	6	0.000138182	-0.000119650	-0.000575503
199	6	-0.000041710	0.000594906	-0.000168571
200	6	-0.000312318	-0.000025469	-0.000066032
201	6	0.000062009	0.000279511	-0.000326936
202	6	-0.000098455	-0.000527635	0.000047613
203	6	-0.000139834	0.001072380	-0.000664474
204	1	-0.000081379	0.000069225	-0.000096211
205	1	0.000486343	-0.000385982	0.000462337
206	1	-0.000097936	-0.000018615	-0.000035426
207	1	0.000124669	-0.000264441	0.000405600
208	1	-0.000047770	-0.000373898	0.000159857
209	1	0.000118782	-0.000294948	0.000083842
210	8	0.000461796	0.000227043	0.000496200
211	8	0.001159782	-0.000008559	-0.000642336
212	6	-0.000514797	-0.000442492	0.000004051
213	6	0.000139569	0.000189305	0.000152217
214	6	-0.000516096	-0.000412143	0.000054208
215	6	0.000203481	0.000685475	-0.000276535
216	6	-0.000053434	0.000228845	0.000201841
217	6	0.000249868	0.000899806	-0.000153812
218	6	-0.000337853	-0.000394960	0.000415625
219	6	0.000035738	0.001351088	-0.000277811
220	1	0.000054113	-0.000070010	0.000058757
221	1	-0.000235548	-0.000887593	-0.000013750
222	1	0.000031672	0.000030653	-0.000019286
223	1	-0.000006186	-0.000535092	-0.000160054
224	1	0.000131163	-0.000338626	0.000121791

225	1	-0.000050051	-0.000404207	0.000191602
226	1	-0.000925955	-0.000055943	0.000705211
227	1	0.000042789	-0.000294918	-0.000250177
228	1	0.000188804	-0.000440568	-0.000776224
229	1	0.000038010	0.000000678	-0.000031833
230	1	-0.000752887	0.000259543	-0.000668905
231	1	-0.000465644	-0.000208115	0.000263928
232	1	-0.000265275	-0.000215329	-0.000751814
233	1	0.000215911	-0.000142838	-0.000147820
234	1	0.000144364	0.000415819	0.000452299
235	1	0.000153792	-0.000079767	-0.000113026
236	8	-0.000047519	0.000403946	-0.000094567
237	8	0.001606323	0.002109109	0.000255082
238	6	-0.000420894	0.000094112	0.000222934
239	6	0.000627337	0.000982422	0.000016384
240	6	-0.001763308	-0.001964689	0.000166701
241	6	0.000347803	0.000421143	0.000213388
242	6	-0.000410945	-0.000279518	0.000041004
243	6	0.000372878	-0.000560416	0.000076044
244	6	0.000010973	0.000246921	-0.000465802
245	6	-0.000170614	-0.001160204	0.000504937
246	1	-0.000273075	-0.000454226	-0.000132446
247	1	-0.000169468	0.000753872	0.000095141
248	1	0.000136281	-0.000073445	-0.000091476
249	1	0.000212090	0.000458567	-0.000018665
250	1	0.000053872	0.000361399	-0.000308802
251	1	-0.000091684	0.000324639	-0.000083771
252	8	-0.000385662	0.000095520	0.000341914
253	8	-0.002377360	0.000169906	-0.001115073
254	6	0.000727132	0.000305906	-0.000666041
255	6	-0.001130504	0.000276668	-0.000464380
256	6	0.002586681	-0.000390368	0.000904439
257	6	-0.000593790	-0.000229311	-0.000560742
258	6	0.000614121	0.000127620	0.000089098
259	6	-0.000101079	-0.000624794	0.000577691
260	6	-0.000162524	0.000527576	0.000153896
261	6	0.000632020	-0.001014221	0.000320926
262	1	0.000416624	-0.000101088	0.000216991
263	1	-0.000154103	0.000452124	-0.000670418
264	1	-0.000039014	0.000002915	0.000163857
265	1	-0.000213744	0.000356330	-0.000011951
266	1	-0.000054151	0.000274306	-0.000137209
267	1	-0.000344373	0.000242573	-0.000227489
268	8	0.000372868	0.000398774	-0.001183687
269	8	0.000515229	0.000412037	0.000667934
270	6	-0.000062307	0.000025547	0.000583374
271	6	-0.000028879	0.000082076	-0.000286777
272	6	-0.000354005	0.000197573	-0.000163820
273	6	0.000424378	-0.000498991	0.000211716
274	6	0.000292340	0.000053644	0.000074271
275	6	0.000238838	-0.000453607	0.000034087
276	6	-0.000189142	0.000212187	-0.000422914
277	6	0.000623002	-0.001035846	0.000312068
278	1	0.000034239	-0.000099072	0.000065812
279	1	-0.000699185	0.000496197	-0.000152102
280	1	0.000048470	0.000001875	0.000055801
281	1	-0.000215226	0.000259531	-0.000226990
282	1	-0.000276666	0.000297924	0.000140248

283	1	-0.000052903	0.000360340	-0.000061377
284	8	-0.000568872	0.000902649	0.000742608
285	8	-0.000426007	-0.000332450	-0.000419207
286	6	0.000185400	-0.000375497	-0.000499327
287	6	0.000022769	0.000238920	0.000189723
288	6	0.000102933	0.000373156	-0.000177508
289	6	-0.000204466	-0.000618255	0.000218570
290	6	-0.000077001	-0.000286187	-0.000001306
291	6	-0.000130690	-0.000335764	0.000412948
292	6	-0.000044008	0.000473970	0.000107416
293	6	-0.000011289	-0.001102214	0.000585995
294	1	-0.000090908	-0.000065323	-0.000005946
295	1	0.000323723	0.000709892	-0.000302778
296	1	-0.000040864	-0.000033200	-0.000054044
297	1	-0.000115185	0.000264600	-0.000191847
298	1	0.000025742	0.000382941	-0.000057903
299	1	0.000114476	0.000341429	-0.000364239
300	1	-0.000376045	0.000480028	-0.000498341
301	1	0.002354484	0.001994501	-0.000739370
302	1	-0.000075583	-0.000616500	0.000294828
303	1	0.002550724	-0.000176925	0.001453816
304	1	0.001536214	0.000528588	-0.002572254
305	1	-0.000207009	-0.000961929	0.000753835
306	1	0.001219243	-0.000593840	-0.000664901
307	1	-0.000506385	0.000802193	-0.001006180
308	1	0.000753952	0.000897774	0.000734344
309	1	-0.001303752	-0.003103629	-0.002259124
310	8	0.000554081	-0.000570509	0.000321998
311	8	-0.002613670	-0.001110589	0.001723257
312	6	-0.000255121	0.000132986	-0.000395117
313	6	-0.001007348	-0.000587527	0.000810485
314	6	0.001422218	0.000114667	-0.001465019
315	6	0.000118522	-0.000119342	0.000480289
316	6	0.000073225	0.000007848	-0.000202316
317	6	0.000508845	0.000166404	0.000268682
318	6	-0.000536372	0.000079265	-0.000269469
319	6	0.001240248	0.000265295	0.000132401
320	1	0.000594972	0.000353458	-0.000408637
321	1	-0.000516441	-0.000511787	0.000149425
322	1	0.000095839	-0.000056295	-0.000114868
323	1	-0.000290735	-0.000194921	-0.000095657
324	1	-0.000442296	-0.000115457	0.000193396
325	1	-0.000376817	0.000017308	-0.000117333
326	8	0.000098835	0.000632446	-0.000165155
327	8	-0.001896847	-0.000456016	-0.002311897
328	6	-0.000370773	-0.000212070	0.000290636
329	6	-0.000810629	-0.000125528	-0.001146775
330	6	0.000657819	0.000583559	0.001699983
331	6	0.000137724	0.000019364	-0.000432228
332	6	-0.000011909	0.000097101	0.000310377
333	6	0.000571009	-0.000001066	-0.000082235
334	6	-0.000455689	-0.000331424	0.000073650
335	6	0.001124492	0.000465177	0.000353544
336	1	0.000489814	0.000043286	0.000545657
337	1	-0.000703174	0.000018171	-0.000383630
338	1	-0.000005286	0.000101289	0.000105887
339	1	-0.000292243	-0.000220444	-0.000013146
340	1	-0.000378969	-0.000026702	-0.000052920

341	1	-0.000359054	-0.000145059	-0.000375682
342	8	0.000178536	0.001283380	-0.001589578
343	8	-0.000827318	0.000760422	0.000812167
344	6	0.000006729	-0.000676214	0.001274524
345	6	-0.000163611	0.000155589	-0.000414806
346	6	-0.000002637	-0.000917998	-0.000013167
347	6	0.000592535	0.000621729	0.000349085
348	6	0.000030294	-0.000056580	0.000404721
349	6	0.000490828	0.000415178	-0.000270326
350	6	-0.000461091	0.000037877	-0.000336147
351	6	0.001030251	0.000663335	0.000522152
352	1	0.000059863	0.000083762	0.000069098
353	1	-0.000507045	-0.000431271	-0.000385949
354	1	-0.000000085	-0.000023868	-0.000186163
355	1	-0.000312248	-0.000121886	-0.000046954
356	1	-0.000336154	-0.000151639	-0.000218719
357	1	-0.000279073	-0.000395041	-0.000169661
358	8	0.000151492	-0.001200764	0.001863665
359	8	-0.000193467	-0.001022083	-0.000754605
360	6	0.000140571	0.000746744	-0.001324166
361	6	-0.000339823	-0.000316290	0.000452690
362	6	-0.000507146	0.000955652	-0.000014714
363	6	0.001000823	-0.000121793	-0.000030942
364	6	0.000074198	0.000210473	-0.000450588
365	6	0.000393960	-0.000061661	0.000500061
366	6	-0.000368826	-0.000327847	0.000163456
367	6	0.001341333	0.000105048	-0.000081886
368	1	0.000292781	-0.000051465	-0.000110832
369	1	-0.000748233	0.000041706	0.000262551
370	1	-0.000051225	0.000038515	0.000192844
371	1	-0.000508524	0.000208973	-0.000068642
372	1	-0.000336574	-0.000114663	-0.000072252
373	1	-0.000454793	-0.000105363	0.000086403
374	1	0.000076240	0.000015936	0.000202547
375	1	-0.000615024	-0.000822359	-0.000341567
376	1	0.000172013	0.000115704	-0.000080186
377	1	-0.002874864	0.000804206	-0.000389869
378	1	0.000576651	-0.000348493	-0.000460045
379	1	0.000492850	0.001082490	0.000133100
380	1	-0.000557718	-0.000073219	0.000236831
381	1	-0.000081092	0.001314671	0.000063255
382	1	0.000301265	-0.000128101	0.000328200
383	1	-0.001783173	0.001027615	0.001777780
384	8	0.000041323	-0.000603731	0.000112660
385	8	0.000676530	0.000656541	0.001143740
386	6	0.000370650	0.000778475	-0.000647100
387	6	0.000197631	0.000173191	0.000610863
388	6	-0.000249435	-0.000184521	-0.000842644
389	6	-0.000289479	-0.000133271	0.000238405
390	6	0.000018781	-0.000193871	-0.000298098
391	6	-0.000542923	-0.000594753	0.000022117
392	6	0.000534625	0.000124843	-0.000146944
393	6	-0.001115363	-0.000671650	-0.000197555
394	1	-0.000165539	-0.000223834	-0.000191150
395	1	0.000405807	0.000598655	0.000159180
396	1	-0.000020561	0.000037449	-0.000157199
397	1	0.000344813	0.000070676	0.000063252
398	1	0.000343102	0.000236237	-0.000050249

399	1	0.000289824	0.000383490	0.000250694
400	8	0.000081675	-0.000300127	0.000158275
401	8	0.002840008	-0.000712560	-0.000690488
402	6	-0.000032758	0.000141444	0.000231610
403	6	0.001392096	-0.000244486	-0.000312246
404	6	-0.001487559	0.000176491	0.000977166
405	6	0.000095638	-0.000262109	-0.000218837
406	6	-0.000175639	0.000028104	0.000170607
407	6	-0.000377620	-0.000181811	-0.000151725
408	6	0.000354273	0.000433261	0.000009600
409	6	-0.001240174	-0.000341919	-0.000064529
410	1	-0.000797789	0.000186246	0.000101908
411	1	0.000752227	-0.000167776	0.000024923
412	1	-0.000077804	-0.000092442	0.000091617
413	1	0.000347094	0.000234997	0.000039884
414	1	0.000364388	0.000033918	0.000153182
415	1	0.000474442	-0.000090089	-0.000164544
416	8	-0.000766386	0.001230684	0.000744089
417	8	0.000859144	0.000225073	-0.000896578
418	6	0.000397006	-0.001023989	-0.000520527
419	6	0.000172378	0.000456246	0.000196676
420	6	0.000195122	-0.000542407	0.000652295
421	6	-0.000562696	-0.000078893	-0.000597564
422	6	0.000010303	-0.000220060	-0.000147589
423	6	-0.000487631	0.000065174	0.000124156
424	6	0.000305177	0.000400657	0.000122598
425	6	-0.001118167	-0.000331449	-0.000579764
426	1	-0.000131263	-0.000043182	-0.000200054
427	1	0.000566328	0.000214045	0.000524523
428	1	-0.000073884	0.000056258	0.000163776
429	1	0.000320846	0.000204431	0.000199893
430	1	0.000372496	-0.000056910	0.000302651
431	1	0.000360363	0.000098685	0.000021639
432	8	0.000435400	-0.002061936	-0.000732939
433	8	-0.000472340	-0.000358358	0.000462406
434	6	-0.000572333	0.001270701	0.000538384
435	6	0.000020681	-0.000819107	-0.000467425
436	6	0.000513192	0.001116081	-0.000531693
437	6	-0.000901677	-0.000657312	0.000337696
438	6	-0.000393424	0.000369332	0.000035554
439	6	-0.000102270	-0.000519144	-0.000083168
440	6	0.000426303	-0.000020155	0.000246629
441	6	-0.001206877	-0.000698042	-0.000073666
442	1	0.000053631	0.000095152	0.000411166
443	1	0.000721364	0.000393622	-0.000225784
444	1	0.000052519	0.000025685	-0.000116775
445	1	0.000322551	0.000352955	-0.000103900
446	1	0.000313102	0.000195751	0.000149728
447	1	0.000430251	0.000108067	0.000013878
448	1	-0.000009860	0.000335370	0.000853973
449	1	0.000001150	-0.000742795	-0.000263419
450	1	-0.000102956	-0.000171867	-0.000241998
451	1	0.000263977	0.000081237	-0.000314543
452	1	-0.001336048	-0.000462552	0.000634460
453	1	-0.000200013	-0.001805289	-0.002222238
454	1	-0.000705203	-0.000771505	0.000389052
455	1	0.000110758	0.000149479	-0.000033322
456	1	-0.000012732	0.000480695	-0.000050427

457	1	0.000297728	0.000374196	0.000273226
458	1	-0.000168755	0.000285245	0.000051990
459	1	-0.000493944	0.000189744	0.000529697
460	1	-0.000142458	-0.000268021	-0.000180728
461	8	-0.002232737	0.001010491	0.001126298
462	1	0.001021318	-0.000124912	-0.000749473
463	1	0.000944105	-0.000701943	-0.000199200
464	8	0.002009529	0.001085260	-0.001407456
465	1	-0.000658128	-0.000887096	0.000417205
466	1	-0.000944775	-0.000213922	0.000769417
467	8	-0.000913686	-0.002022282	-0.001262272
468	1	0.000321814	0.000633978	0.000812174
469	1	0.000380007	0.001258694	0.000135877

Sum of electronic and thermal Free Energies= -479.348529
0 imaginary frequency

3a@C$_R$

Center Number	Atomic Number	Integrated Forces (Hartrees/Bohr) X	Y	Z
1	8	0.000354327	-0.000212963	-0.000042436
2	8	0.002162364	0.005703497	-0.005502036
3	6	-0.000381131	-0.000311348	0.000053982
4	6	-0.000255641	0.000094085	-0.000340721
5	6	-0.000492639	-0.002905901	0.004426227
6	6	0.000057783	0.000455459	0.000142451
7	6	0.000215877	0.000124703	0.000137267
8	6	0.000149789	0.000445430	0.000469687
9	6	0.000047553	-0.000573753	-0.000048399
10	6	0.000224668	0.001177659	0.000806524
11	1	0.000321337	0.000028539	0.000193242
12	1	-0.000371307	-0.000500478	-0.000545216
13	1	-0.000115387	-0.000038224	0.000083987
14	1	-0.000087464	-0.000440352	-0.000152404
15	1	-0.000201769	-0.000279827	-0.000393717
16	1	0.000029981	-0.000316409	-0.000208316
17	8	-0.000116957	-0.000304205	0.000174046
18	8	0.001520052	-0.002959374	0.000070150
19	6	0.000601191	0.000360673	-0.000545482
20	6	0.000432751	-0.001128608	0.000287514
21	6	-0.000554591	0.002093824	-0.000737777
22	6	0.000234738	-0.000318877	0.000472874
23	6	-0.000118922	0.000368186	-0.000292567
24	6	-0.000450466	0.000349370	0.000467077
25	6	0.000034891	-0.000254520	-0.000480246
26	6	-0.000533721	0.000896595	0.000859089
27	1	-0.000356466	0.000463806	-0.000046309
28	1	0.000552788	-0.000450376	-0.000252286
29	1	0.000058383	0.000156332	-0.000064606
30	1	0.000297623	-0.000393708	-0.000128130
31	1	0.000045343	-0.000269348	-0.000248552
32	1	0.000205993	-0.000180967	-0.000358408
33	8	-0.000741460	0.001056901	-0.000439149
34	8	0.000235780	0.000621930	0.000169219
35	6	0.000378241	-0.000852820	0.000224035

36	6	0.000047798	0.000502178	-0.000570037
37	6	0.000132965	-0.000693533	-0.000143644
38	6	-0.000188337	0.000637150	0.000390521
39	6	-0.000077431	-0.000069316	0.000231780
40	6	-0.000018948	0.000662045	0.000343963
41	6	-0.000097877	-0.000479964	-0.000209048
42	6	-0.000585231	0.001023902	0.000768185
43	1	-0.000023681	-0.000151200	0.000266728
44	1	0.000143784	-0.000487121	-0.000732230
45	1	0.000079985	-0.000022663	0.000103855
46	1	0.000186996	-0.000366175	-0.000121279
47	1	0.000089211	-0.000291599	-0.000253571
48	1	0.000377473	-0.000225242	-0.000340563
49	8	0.001683879	-0.000508896	0.001379953
50	8	0.000075825	0.000169543	0.000537292
51	6	-0.001188732	0.000631186	-0.000996277
52	6	0.000382098	-0.000469118	0.000822219
53	6	-0.000446102	-0.000610857	-0.000772206
54	6	0.000258040	0.000798706	0.000618580
55	6	-0.000418406	0.000222894	-0.000173887
56	6	0.000220786	0.000025137	0.000554394
57	6	0.000085764	-0.000135999	-0.000555668
58	6	0.000322030	0.001060478	0.000988268
59	1	0.000024025	0.000308654	-0.000344986
60	1	0.000041405	-0.000813602	-0.000218619
61	1	-0.000127152	0.000045105	-0.000067145
62	1	-0.000001175	-0.000323679	-0.000237992
63	1	-0.000275088	-0.000435994	-0.000228579
64	1	-0.000070958	-0.000269722	-0.000420583
65	8	-0.000579364	0.000059881	-0.000217393
66	8	-0.000141915	0.003110648	-0.000081140
67	6	0.000952781	0.000098423	0.000651901
68	6	-0.000269233	0.001113816	-0.000422746
69	6	0.000664604	-0.001956249	0.000731051
70	6	0.000031843	0.000378880	-0.000487922
71	6	0.000119629	-0.000438055	0.000512483
72	6	-0.000352875	-0.000708265	-0.000471199
73	6	0.000053815	0.000258959	0.000410371
74	6	-0.000160907	-0.001103977	-0.000644850
75	1	-0.000083716	-0.000441223	0.000162332
76	1	0.000304784	0.000741015	0.000200891
77	1	0.000112674	-0.000083299	0.000046553
78	1	0.000131041	0.000287751	0.000277811
79	1	0.000072709	0.000474511	0.000056818
80	1	-0.000048451	0.000259637	0.000221691
81	8	0.000050611	0.000259510	0.000251725
82	8	-0.000716190	0.000619056	0.002851396
83	6	-0.000439743	0.000055493	-0.000278384
84	6	-0.000253754	0.000117766	0.001147276
85	6	-0.000100699	0.000143314	-0.002050854
86	6	-0.000084205	-0.000433160	0.000440840
87	6	0.000019913	0.000200139	-0.000373715
88	6	0.000317766	-0.000353913	-0.000252590
89	6	0.000046332	0.000511487	0.000179572
90	6	0.000358780	-0.000901175	-0.000803831
91	1	0.000230854	-0.000026866	-0.000530714
92	1	-0.000442505	0.000283879	0.000514964
93	1	-0.000061947	0.000044696	-0.000136388

94	1	-0.000023892	0.000304711	0.000198722
95	1	-0.000188695	0.000282478	0.000141653
96	1	-0.000175512	0.000186848	0.000435273
97	8	-0.001123461	-0.001466015	0.001319032
98	8	-0.000040141	-0.000659165	-0.000168932
99	6	0.000767807	0.000853231	-0.001125218
100	6	-0.000129658	-0.000498027	0.000702660
101	6	0.000477597	0.001063021	0.000277902
102	6	-0.000404712	-0.000791806	-0.000667968
103	6	0.000357783	0.000124927	-0.000376589
104	6	-0.000169747	-0.000518604	0.000112694
105	6	-0.000256916	0.000383525	0.000132551
106	6	-0.000206262	-0.001184215	-0.000672584
107	1	-0.000165796	0.000120926	-0.000406473
108	1	0.000130620	0.000460734	0.000720967
109	1	0.000095644	0.000071132	-0.000076509
110	1	0.000263446	0.000374473	0.000278968
111	1	0.000017757	0.000447918	0.000103313
112	1	-0.000004050	0.000270816	0.000254070
113	8	0.000871774	0.000903081	-0.001203749
114	8	-0.000343594	-0.000382388	0.000156093
115	6	-0.000463432	-0.000794825	0.000857462
116	6	0.000001059	0.000509903	-0.000376802
117	6	-0.000210677	0.000267320	0.000164122
118	6	0.000301421	-0.000613154	-0.000395453
119	6	-0.000069264	-0.000241352	0.000088506
120	6	0.000044366	-0.000215031	-0.000542257
121	6	0.000091886	0.000367852	0.000281540
122	6	0.000561612	-0.000909387	-0.000775957
123	1	0.000114674	-0.000270702	0.000107936
124	1	-0.000371041	0.000760298	0.000164852
125	1	-0.000047465	-0.000059900	0.000058981
126	1	-0.000123903	0.000199533	0.000324899
127	1	-0.000093675	0.000328080	0.000178524
128	1	-0.000383593	0.000286367	0.000212338
129	8	0.000188338	-0.000457425	-0.000843068
130	1	-0.000118021	0.000246184	0.000218092
131	1	0.000157628	0.000157603	0.000252011
132	8	-0.000435744	0.000496081	-0.000082205
133	1	-0.000202146	0.000585299	-0.000805667
134	1	-0.000905875	0.000251062	0.000393413
135	8	-0.003980027	0.000409669	0.009172871
136	1	0.000969158	0.001225907	-0.005823988
137	1	0.003820166	-0.001735996	-0.003714876
138	8	0.000138995	-0.000821328	0.000122310
139	1	0.000181390	-0.000477495	0.000775103
140	1	-0.000797352	-0.000609940	-0.000313594
141	8	-0.004623428	-0.001331787	-0.009095687
142	1	0.002180296	-0.001014496	0.005905828
143	1	0.003589613	0.002489069	0.004965717
144	1	-0.000053166	-0.000096597	-0.000247100
145	1	0.000748678	-0.000119089	0.000135518
146	1	0.000084522	0.000456914	-0.000038589
147	1	0.000100405	-0.000671919	-0.000135769
148	1	0.000144072	0.000121082	-0.000202889
149	8	-0.000016933	-0.000523908	0.000465895
150	8	0.002650377	-0.000523686	0.000628150
151	6	-0.000012734	0.000135051	-0.000202678

152	6	0.001022371	-0.000457102	0.000475960
153	6	-0.002644082	0.000320364	-0.000905912
154	6	0.000595456	-0.000306138	-0.000135241
155	6	-0.000326561	0.000009420	-0.000062298
156	6	0.000039616	0.000238819	-0.000230272
157	6	0.000105368	0.000185576	0.000662977
158	6	-0.000547151	0.000327213	-0.001154061
159	1	-0.000409066	0.000207555	-0.000215712
160	1	0.000295279	-0.000509767	0.000308136
161	1	-0.000023821	0.000181140	0.000072325
162	1	0.000046815	-0.000181878	0.000253309
163	1	0.000394386	-0.000173957	0.000306671
164	1	0.000143745	-0.000048241	0.000459546
165	8	0.000391205	-0.000094755	0.000349241
166	8	-0.001415641	-0.000136810	0.000100179
167	6	-0.000180602	0.000079984	0.000229757
168	6	-0.000221905	0.000119107	0.000220159
169	6	0.000731280	0.000346940	0.000063134
170	6	-0.000179796	0.000161496	-0.000518363
171	6	0.000143482	-0.000033455	-0.000177862
172	6	0.000005627	0.000164148	-0.000621681
173	6	0.000309695	-0.000209406	0.000353340
174	6	-0.000027466	0.000755473	-0.001170099
175	1	0.000258950	-0.000094355	-0.000218445
176	1	-0.000107222	-0.000142969	0.000804750
177	1	-0.000007196	-0.000070161	-0.000075130
178	1	0.000031103	-0.000346242	0.000258965
179	1	0.000116130	-0.000154887	0.000395876
180	1	-0.000212262	-0.000238493	0.000437788
181	8	0.000394179	-0.001627697	-0.000198080
182	8	-0.000406131	0.000314175	0.000254539
183	6	-0.000517732	0.000782457	0.000199258
184	6	0.000247567	-0.000302654	-0.000027432
185	6	0.000197404	-0.000115705	0.000361232
186	6	-0.000296035	0.000164283	-0.000833721
187	6	-0.000368783	0.000142144	0.000054309
188	6	0.000098670	-0.000065882	-0.000534344
189	6	-0.000054825	-0.000423272	0.000261667
190	6	-0.000289674	0.000564544	-0.001215164
191	1	-0.000052105	0.000058291	-0.000168873
192	1	0.000535620	-0.000119368	0.000646294
193	1	-0.000104552	-0.000004931	-0.000002436
194	1	0.000140146	0.000012301	0.000484115
195	1	-0.000019953	-0.000207410	0.000380925
196	1	0.000161596	-0.000245931	0.000264998
197	8	0.000188630	0.000033717	0.000283325
198	8	0.001271991	-0.001138200	-0.000354037
199	6	-0.000646875	-0.000156733	0.000202129
200	6	0.000394432	-0.000308041	0.000187486
201	6	-0.000635230	0.000858771	0.000170695
202	6	0.000247263	-0.000233353	-0.000489570
203	6	-0.000157367	0.000511840	0.000221724
204	6	0.000443879	0.000504106	-0.000707010
205	6	-0.000274316	-0.000120013	0.000524768
206	6	0.000107811	0.000844948	-0.000977435
207	1	0.000005396	0.000069026	-0.000111736
208	1	-0.000278004	-0.000592291	0.000494938
209	1	0.000029006	0.000028484	-0.000002110

210	1	-0.000061177	-0.000428755	0.000215644
211	1	0.000102495	-0.000202888	0.000238658
212	1	-0.000067483	-0.000183416	0.000377030
213	1	-0.000791225	0.000445314	0.000521497
214	1	-0.000080039	0.000017941	-0.000142666
215	1	-0.000155395	-0.000733017	-0.000439926
216	1	0.000109799	0.000098198	0.000089033
217	1	-0.000796456	0.000039428	-0.000789705
218	1	-0.000040041	0.000015847	0.000382667
219	1	-0.000203009	-0.000328325	-0.000716575
220	1	0.000213638	-0.000217109	-0.000103431
221	1	0.000331329	0.000784788	0.000272387
222	1	0.000113572	0.000212903	0.000227961
223	8	0.000193744	-0.000145346	-0.000158419
224	8	0.001937572	0.001490415	-0.000923667
225	6	-0.000619406	0.000480021	0.000112012
226	6	0.000788414	0.000556034	-0.000525572
227	6	-0.001946509	-0.001156715	0.001256332
228	6	0.000480328	0.000356525	0.000047146
229	6	-0.000435409	-0.000190581	0.000021480
230	6	0.000317537	-0.000536574	0.000381731
231	6	-0.000089197	-0.000015613	-0.000521085
232	6	-0.000098145	-0.000659683	0.001101893
233	1	-0.000333260	-0.000275831	0.000195827
234	1	-0.000032901	0.000645347	-0.000245067
235	1	0.000048490	-0.000127030	-0.000057379
236	1	0.000224705	0.000379240	-0.000347926
237	1	0.000009764	0.000113808	-0.000440367
238	1	-0.000095637	0.000233604	-0.000244201
239	8	-0.000243509	0.000143357	-0.000106843
240	8	-0.002484692	-0.000179762	-0.001181920
241	6	0.000497751	-0.000087096	-0.000373768
242	6	-0.001118704	0.000092717	-0.000654464
243	6	0.002619318	-0.000260865	0.001058401
244	6	-0.000674652	-0.000276555	-0.000329228
245	6	0.000542133	-0.000052543	0.000041924
246	6	-0.000107117	-0.000139029	0.000628480
247	6	-0.000162717	0.000560164	-0.000098254
248	6	0.000629840	-0.000783921	0.000781143
249	1	0.000473802	0.000028798	0.000286432
250	1	-0.000218199	0.000060166	-0.000651980
251	1	-0.000048962	0.000095866	0.000146600
252	1	-0.000203752	0.000360582	-0.000202048
253	1	-0.000047921	0.000166476	-0.000288907
254	1	-0.000382644	0.000118402	-0.000352794
255	8	-0.000116196	-0.000544700	-0.001392955
256	8	0.000434893	0.000197271	0.000449212
257	6	0.000189686	0.000523620	0.000710652
258	6	-0.000175225	-0.000246666	-0.000215556
259	6	-0.000198670	0.000366470	-0.000318101
260	6	0.000391349	-0.000483781	0.000479772
261	6	0.000270183	0.000035353	0.000094222
262	6	0.000216022	-0.000447371	0.000108673
263	6	-0.000130393	0.000021316	-0.000498403
264	6	0.000508123	-0.000791686	0.000868926
265	1	0.000136671	-0.000010694	0.000052071
266	1	-0.000533886	0.000430819	-0.000370582
267	1	0.000071131	0.000012446	-0.000004239

268	1	-0.000165522	0.000124964	-0.000310211
269	1	-0.000306210	0.000396337	-0.000157347
270	1	-0.000025788	0.000260133	-0.000257734
271	8	-0.000395615	0.001111597	0.000115496
272	8	-0.000609327	-0.000735635	-0.000422408
273	6	0.000195834	-0.000538051	-0.000178103
274	6	-0.000042484	0.000169975	-0.000020120
275	6	0.000185232	0.000379161	-0.000092442
276	6	-0.000218673	-0.000470625	0.000414448
277	6	-0.000041483	-0.000020268	0.000286929
278	6	-0.000165339	-0.000185253	0.000557986
279	6	0.000141004	0.000468819	-0.000175535
280	6	-0.000150324	-0.000622085	0.001120992
281	1	-0.000097097	0.000010696	0.000085016
282	1	0.000258727	0.000386447	-0.000725730
283	1	-0.000019212	-0.000051680	-0.000004131
284	1	-0.000110049	0.000153814	-0.000320942
285	1	0.000055851	0.000319621	-0.000283652
286	1	0.000165658	0.000041668	-0.000487477
287	1	-0.000403247	0.000169456	-0.000501347
288	1	0.002196007	0.000852149	-0.001806175
289	1	-0.000072671	-0.000110665	0.000521865
290	1	0.002670060	0.000395915	0.001503369
291	1	0.001322181	-0.001174606	-0.002110787
292	1	-0.000221635	-0.000306891	0.001219905
293	1	0.001249095	-0.000757193	-0.000297101
294	1	-0.000622879	0.000271464	-0.001176248
295	1	0.000604326	0.001179599	0.000107849
296	1	-0.001416324	-0.002848229	0.001131825
297	8	0.000450220	-0.000278132	0.000475649
298	8	-0.002203509	0.000233566	0.001912357
299	6	-0.000290146	-0.000129647	-0.000370005
300	6	-0.000811989	0.000139502	0.001045418
301	6	0.001139874	-0.000748873	-0.001033201
302	6	0.000201918	0.000156358	0.000308943
303	6	0.000020164	-0.000062874	-0.000160865
304	6	0.000560954	0.000167846	0.000084366
305	6	-0.000570954	0.000008406	-0.000265253
306	6	0.001300947	0.000077941	-0.000141624
307	1	0.000520589	0.000017596	-0.000584537
308	1	-0.000606503	-0.000304198	0.000411859
309	1	0.000073753	-0.000083974	-0.000038227
310	1	-0.000329927	-0.000149194	0.000051828
311	1	-0.000421010	0.000102951	0.000297548
312	1	-0.000418306	-0.000016669	-0.000078929
313	8	0.000436675	-0.000140759	-0.000458995
314	8	-0.002178477	-0.001578883	-0.001853340
315	6	-0.000742512	0.000469967	0.000291135
316	6	-0.000825308	-0.000782825	-0.001006760
317	6	0.000875324	0.001476468	0.001267423
318	6	0.000174420	-0.000305064	-0.000684064
319	6	0.000054378	0.000366426	0.000295744
320	6	0.000753460	-0.000190874	-0.000104836
321	6	-0.000497314	-0.000131388	0.000307931
322	6	0.001210786	0.000442888	-0.000069220
323	1	0.000540086	0.000323638	0.000507426
324	1	-0.000772479	-0.000112000	-0.000401169
325	1	0.000017457	0.000148422	0.000039222

326	1	-0.000315934	-0.000148734	0.000145494
327	1	-0.000387677	-0.000000884	0.000010828
328	1	-0.000365637	-0.000281738	-0.000176721
329	8	0.000383567	0.000008170	-0.001975109
330	8	-0.000705634	0.000964956	0.000254057
331	6	-0.000159577	0.000266473	0.001329496
332	6	-0.000207053	-0.000202786	-0.000419347
333	6	-0.000021310	-0.000753670	0.000413024
334	6	0.000659061	0.000592221	-0.000092277
335	6	0.000037721	0.000078315	0.000356640
336	6	0.000427965	0.000104444	-0.000366463
337	6	-0.000515923	-0.000061791	-0.000252199
338	6	0.001146121	0.000658955	-0.000071013
339	1	0.000116820	0.000184766	0.000054061
340	1	-0.000571119	-0.000557466	-0.000064997
341	1	0.000020383	-0.000103086	-0.000123626
342	1	-0.000339994	-0.000061942	0.000046723
343	1	-0.000381879	-0.000194229	-0.000068744
344	1	-0.000327735	-0.000384120	0.000189852
345	8	0.000750800	0.000037097	0.001848792
346	8	-0.000138351	-0.001370606	-0.000112962
347	6	-0.000362770	-0.000213272	-0.001249897
348	6	0.000019631	-0.000017426	0.000464405
349	6	-0.000603385	0.000903983	-0.000468108
350	6	0.001012480	-0.000300518	-0.000001765
351	6	0.000145285	0.000007157	-0.000502650
352	6	0.000562065	0.000034982	0.000365650
353	6	-0.000385177	-0.000136252	0.000377789
354	6	0.001380380	-0.000139669	-0.000168134
355	1	0.000044038	-0.000146483	0.000044507
356	1	-0.000831497	0.000361614	0.000245337
357	1	-0.000007676	0.000214910	0.000085969
358	1	-0.000416016	0.000187515	-0.000105889
359	1	-0.000363640	-0.000094640	0.000013862
360	1	-0.000458445	0.000016022	0.000176560
361	1	0.000441057	0.000268025	0.000616284
362	1	-0.000547538	-0.000863446	0.000320553
363	1	-0.000346996	0.000140252	-0.000063921
364	1	-0.002809771	0.000818336	-0.000589043
365	1	0.000526993	-0.000530477	0.000304662
366	1	0.000684843	0.000989414	-0.000469190
367	1	-0.000485254	0.000222350	0.000219690
368	1	0.000119324	0.001198350	-0.000494098
369	1	-0.000895866	0.000023302	0.000576392
370	1	-0.001858904	0.002250137	0.000870734
371	8	-0.000461728	-0.000327942	-0.000901415
372	8	0.000453443	0.000412562	0.000145707
373	6	0.000270744	0.000194695	0.000233966
374	6	-0.000066359	0.000196154	-0.000146795
375	6	0.000107028	0.000267953	-0.000134503
376	6	-0.000812906	-0.000534840	-0.000060130
377	6	-0.000017348	-0.000045740	0.000328957
378	6	-0.000346469	-0.000312533	-0.000025784
379	6	0.000580099	-0.000062278	-0.000013302
380	6	-0.001227804	-0.000625185	-0.000059817
381	1	0.000073568	-0.000247523	0.000086156
382	1	0.000602519	0.000604384	-0.000270576
383	1	0.000011981	-0.000042706	-0.000081152

384	1	0.000396001	0.000069399	0.000073626
385	1	0.000350838	0.000148404	-0.000076160
386	1	0.000328950	0.000436276	0.000101299
387	8	-0.000258834	-0.000831795	-0.000075700
388	8	0.002799662	-0.001550800	-0.000573362
389	6	0.000435773	0.000536963	0.000569570
390	6	0.001265383	-0.000625292	-0.000309569
391	6	-0.001590441	0.001103578	0.000923492
392	6	0.000228830	-0.000576799	-0.000153990
393	6	-0.000171474	0.000352812	0.000448424
394	6	-0.000624085	-0.000370818	-0.000239476
395	6	0.000443309	0.000344935	-0.000159960
396	6	-0.001192513	-0.000278497	0.000211974
397	1	-0.000669834	0.000354422	0.000040413
398	1	0.000702254	-0.000124494	0.000168841
399	1	-0.000081210	0.000092813	0.000130474
400	1	0.000302331	0.000194030	-0.000118821
401	1	0.000349449	0.000101577	0.000055734
402	1	0.000480948	-0.000170898	-0.000143869
403	8	-0.000546781	0.000501012	-0.000622442
404	8	0.000711274	-0.000743773	-0.001115438
405	6	0.000362563	-0.000182412	0.000290391
406	6	0.000315522	-0.000037340	-0.000383304
407	6	0.000174323	-0.000049952	0.000742109
408	6	-0.000439868	-0.000494837	0.000062460
409	6	-0.000077634	0.000015086	-0.000203918
410	6	-0.000621407	-0.000465007	-0.000149041
411	6	0.000221493	0.000646191	-0.000067027
412	6	-0.001125662	-0.000740272	-0.000335061
413	1	-0.000106839	0.000041078	-0.000061314
414	1	0.000597991	0.000484806	0.000359723
415	1	-0.000035961	0.000164467	0.000107106
416	1	0.000370018	0.000313818	0.000024980
417	1	0.000405208	0.000164173	0.000305567
418	1	0.000325709	0.000103709	-0.000008708
419	8	0.000650946	-0.002188060	0.000727866
420	8	0.000285155	-0.000202021	0.000247968
421	6	-0.001175921	0.001755658	-0.000112675
422	6	0.000523178	-0.000669717	0.000190712
423	6	0.000283318	0.000518836	-0.000947304
424	6	-0.001002733	-0.000340847	0.000553341
425	6	-0.000227776	0.000547740	-0.000248142
426	6	-0.000033765	-0.000850024	-0.000102726
427	6	0.000455843	0.000231692	0.000242821
428	6	-0.001202161	-0.000506010	0.000270057
429	1	-0.000133683	0.000081938	0.000122779
430	1	0.000703067	0.000010405	-0.000411002
431	1	0.000040106	-0.000123327	-0.000024602
432	1	0.000324224	0.000223704	-0.000296511
433	1	0.000325034	0.000183435	0.000026180
434	1	0.000380368	0.000039451	-0.000065601
435	1	0.000045304	0.000726834	0.000672212
436	1	0.000400379	-0.000581331	0.000267970
437	1	-0.000430490	-0.000574916	-0.000214626
438	1	0.000246414	0.000039315	-0.000388080
439	1	-0.000968067	0.000264830	0.001112451
440	1	-0.000536614	-0.002604231	-0.000689151
441	1	-0.000513694	-0.000355543	0.000796160

442	1	0.000184240	-0.000015172	-0.000285573
443	1	-0.000107150	0.000858074	-0.000238036
444	1	0.000373085	0.000399155	-0.000113571
445	6	-0.000189203	0.002554574	0.000599546
446	6	0.000009805	0.000628998	0.000322078
447	6	-0.000011996	-0.000752612	0.000514884
448	6	0.000474486	-0.000099870	-0.000260929
449	6	0.000722084	0.000175393	-0.000291467
450	6	0.000213767	-0.000344184	0.000177793
451	1	0.000881488	0.004760071	-0.004330856
452	1	0.002110440	-0.001809266	-0.005845357
453	1	-0.001819232	-0.004416757	0.004566700
454	1	-0.002646262	0.002561574	0.005307549
455	17	-0.001786088	0.001456607	0.003475951
456	1	-0.000131861	0.000265841	-0.000214230
457	1	-0.000319964	0.000907996	0.000610780
458	1	-0.000119412	-0.000060894	0.000132923
459	8	-0.002083893	0.001436526	0.000331880
460	1	0.000885334	-0.000569677	-0.000442538
461	1	0.000846419	-0.000685349	0.000242374
462	8	0.001490741	-0.000669011	-0.001962671
463	1	-0.000443738	0.000030280	0.000958381
464	1	-0.000776460	0.000618988	0.000636434
465	8	-0.000969740	-0.002293345	-0.000011606
466	1	0.000478764	0.000991354	0.000295018
467	1	0.000217342	0.000968846	-0.000322634
468	6	-0.001237185	-0.002078604	0.002093467
469	8	0.001511848	0.004472961	-0.005515659
470	1	0.000812621	-0.006146309	-0.001198903

Sum of electronic and thermal Free Energies= -1035.079487
0 imaginary frequency

3e@C_R

Center Number	Atomic Number	Integrated Forces (Hartrees/Bohr) X	Y	Z
1	8	-0.000009828	-0.000028285	0.000000074
2	8	0.003726513	-0.002880373	-0.005779674
3	6	-0.000001748	0.000011919	0.000006400
4	6	0.000003400	-0.000003860	0.000000335
5	6	-0.001115882	0.003114060	0.003007004
6	6	0.000002306	0.000003447	-0.000006075
7	6	0.000008051	0.000006302	-0.000003358
8	6	-0.000013222	-0.000007378	0.000000977
9	6	0.000004427	-0.000005976	0.000002728
10	6	-0.000000559	0.000005637	0.000000891
11	1	-0.000005184	0.000001141	-0.000005208
12	1	0.000001218	-0.000001384	0.000003462
13	1	0.000001765	-0.000001187	-0.000000485
14	1	-0.000000695	-0.000000644	-0.000000159
15	1	-0.000000018	-0.000000960	0.000000072
16	1	-0.000000542	-0.000002569	0.000000141
17	8	0.000010748	-0.000000384	0.000014232
18	8	0.000004509	0.000001658	-0.000005835
19	6	0.000001567	-0.000004399	-0.000002237

20	6	-0.000001729	0.000001269	-0.000001309
21	6	-0.000000379	0.000002469	-0.000001039
22	6	-0.000001997	0.000002383	0.000001651
23	6	0.000005162	-0.000002208	-0.000000289
24	6	-0.000006848	0.000000494	0.000002132
25	6	0.000003631	-0.000002332	-0.000001247
26	6	-0.000000975	-0.000000376	0.000000136
27	1	0.000000594	-0.000000535	0.000000424
28	1	0.000000105	0.000000900	-0.000000136
29	1	0.000001541	0.000000185	-0.000000158
30	1	0.000000125	0.000000256	0.000000114
31	1	-0.000000123	0.000000093	0.000000296
32	1	-0.000000005	0.000000093	0.000000273
33	8	-0.000018502	0.000014925	-0.000003710
34	8	-0.000008499	-0.000006884	-0.000004190
35	6	0.000004276	-0.000009390	-0.000005966
36	6	-0.000001070	0.000008118	-0.000005289
37	6	-0.000000156	-0.000010320	0.000001918
38	6	-0.000003473	0.000000428	0.000001384
39	6	-0.000006201	-0.000005218	-0.000001143
40	6	0.000000345	0.000006434	0.000003163
41	6	0.000000136	0.000007458	-0.000002032
42	6	0.000001629	-0.000004995	0.000004366
43	1	-0.000001940	-0.000001933	0.000000766
44	1	0.000000511	-0.000000324	-0.000002194
45	1	0.000003421	-0.000000815	0.000000880
46	1	-0.000000693	0.000001580	-0.000000734
47	1	-0.000000195	0.000000685	-0.000000705
48	1	-0.000000529	0.000000096	-0.000001740
49	8	0.000002710	0.000010517	-0.000001478
50	8	-0.000002640	-0.000002516	0.000007204
51	6	0.000000488	-0.000003226	0.000012607
52	6	0.000002691	-0.000001721	-0.000002863
53	6	0.000003489	-0.000002170	0.000004718
54	6	0.000000991	0.000003816	-0.000002192
55	6	0.000005145	0.000000745	0.000000351
56	6	-0.000004534	0.000000021	-0.000005961
57	6	0.000006141	0.000004809	-0.000004107
58	6	-0.000000673	-0.000004647	0.000002762
59	1	-0.000000383	0.000001406	0.000001059
60	1	-0.000001497	0.000000421	0.000001050
61	1	-0.000003180	0.000000193	0.000002275
62	1	-0.000000279	0.000000626	-0.000001556
63	1	0.000000190	0.000001377	-0.000000232
64	1	0.000000411	0.000000788	-0.000001122
65	8	-0.000003139	0.000000224	-0.000001887
66	8	0.000006584	0.000003308	0.000001043
67	6	0.000003826	0.000003667	0.000001617
68	6	-0.000001004	-0.000000101	-0.000000643
69	6	0.000001084	0.000001447	0.000004773
70	6	0.000000590	-0.000000370	0.000000287
71	6	0.000000199	-0.000000135	-0.000000358
72	6	0.000001997	0.000000846	0.000000025
73	6	0.000001017	-0.000000730	0.000000361
74	6	0.000000173	-0.000001214	0.000000207
75	1	0.000000133	0.000000298	0.000000375
76	1	-0.000000299	0.000000121	0.000000314
77	1	0.000000367	-0.000000903	-0.000000137

78	1	-0.000000177	0.000000158	0.000000009
79	1	0.000000111	0.000000314	0.000000032
80	1	-0.000000081	0.000000017	-0.000000032
81	8	-0.000003157	0.000002807	-0.000000815
82	8	-0.000002340	0.000007761	0.000001243
83	6	0.000000832	0.000000645	0.000000742
84	6	-0.000000545	0.000000623	0.000000214
85	6	-0.000000077	0.000001091	0.000000187
86	6	0.000001721	-0.000000742	0.000001175
87	6	-0.000000194	0.000001337	0.000000919
88	6	0.000000986	-0.000001045	0.000000811
89	6	-0.000000280	0.000001360	-0.000002066
90	6	0.000000273	-0.000000908	0.000000126
91	1	0.000000365	-0.000000028	-0.000000081
92	1	-0.000000086	0.000000027	-0.000000052
93	1	-0.000000035	-0.000000644	-0.000000433
94	1	0.000000080	0.000000472	-0.000000021
95	1	0.000000066	0.000000072	-0.000000214
96	1	0.000000009	0.000000187	-0.000000182
97	8	-0.000001671	0.000004154	0.000003728
98	8	0.000004270	-0.000000124	-0.000001226
99	6	-0.000002070	-0.000000909	-0.000000768
100	6	0.000000711	0.000001306	0.000000055
101	6	0.000000455	-0.000001199	0.000000225
102	6	-0.000000151	-0.000000124	-0.000000389
103	6	-0.000001638	0.000000529	0.000000008
104	6	-0.000000147	-0.000000597	0.000001357
105	6	-0.000000202	-0.000000341	-0.000000417
106	6	0.000000624	0.000000057	0.000000485
107	1	-0.000000371	-0.000000242	-0.000000150
108	1	0.000000147	0.000000263	-0.000000223
109	1	0.000000282	0.000000009	-0.000000465
110	1	-0.000000366	0.000000303	-0.000000015
111	1	-0.000000143	-0.000000015	-0.000000222
112	1	-0.000000284	-0.000000053	0.000000030
113	8	-0.000002243	-0.000000020	-0.000003303
114	8	0.000004738	-0.000007334	0.000002212
115	6	-0.000001957	0.000001817	-0.000005172
116	6	0.000000232	-0.000000187	-0.000000745
117	6	-0.000002381	0.000002739	0.000002051
118	6	0.000001184	0.000000512	-0.000000953
119	6	-0.000000816	0.000000126	0.000000840
120	6	-0.000001063	-0.000000698	0.000003891
121	6	-0.000001190	-0.000000285	-0.000000333
122	6	-0.000000154	-0.000000556	0.000000091
123	1	-0.000000346	-0.000000194	0.000000194
124	1	-0.000000095	-0.000000049	0.000000074
125	1	0.000000174	-0.000000001	0.000000336
126	1	-0.000000264	0.000000009	0.000000145
127	1	0.000000006	0.000000064	0.000000052
128	1	0.000000253	-0.000000065	0.000000158
129	8	-0.000003099	0.000003536	0.000004055
130	1	-0.000000032	-0.000003433	0.000002568
131	1	0.000002252	0.000003928	-0.000006939
132	8	0.000008383	0.000000514	0.000003273
133	1	-0.000000643	0.000000427	0.000003343
134	1	-0.000005755	0.000002465	-0.000001456
135	8	-0.004738625	0.006317465	-0.005349297

136	1	0.002785025	-0.005038631	0.001219742
137	1	0.002163687	-0.000609127	0.003574170
138	8	-0.000003174	0.000003140	-0.000007486
139	1	0.000002018	-0.000000564	0.000001906
140	1	0.000000142	0.000005392	0.000001409
141	8	-0.003192137	-0.006365582	0.003171728
142	1	0.002506062	0.002002704	-0.003587309
143	1	0.002190535	0.005052808	-0.000366828
144	1	-0.000001247	0.000015522	0.000009996
145	1	0.000004790	0.000000807	-0.000005910
146	1	0.000001038	0.000005064	0.000001634
147	1	-0.000000646	-0.000000188	-0.000002994
148	1	-0.000007653	0.000012574	0.000013074
149	8	-0.000004426	0.000001091	-0.000003535
150	8	0.000006084	0.000003776	-0.000005415
151	6	-0.000002231	-0.000000939	-0.000000203
152	6	-0.000000139	-0.000000024	0.000001900
153	6	-0.000001240	-0.000002404	-0.000007538
154	6	0.000004007	0.000000883	0.000003965
155	6	-0.000003011	0.000000009	0.000000617
156	6	-0.000001793	-0.000001628	-0.000000699
157	6	-0.000000541	0.000002600	0.000000808
158	6	0.000000818	-0.000000673	-0.000000609
159	1	0.000000699	-0.000000095	0.000000340
160	1	0.000000289	0.000000788	-0.000000181
161	1	-0.000000247	0.000001100	0.000000414
162	1	-0.000000140	-0.000000198	-0.000000049
163	1	-0.000000582	-0.000000138	-0.000000221
164	1	-0.000000216	-0.000000040	0.000000014
165	8	-0.000004779	-0.000006663	-0.000001379
166	8	-0.000010751	-0.000014769	0.000000412
167	6	0.000008401	-0.000005829	-0.000002891
168	6	-0.000002514	0.000000969	0.000002068
169	6	-0.000007349	0.000010271	0.000004443
170	6	0.000007504	-0.000007100	-0.000005033
171	6	-0.000007928	0.000003508	0.000003189
172	6	-0.000001060	0.000002127	0.000001146
173	6	-0.000002341	0.000002187	-0.000000123
174	6	-0.000000197	-0.000000578	-0.000000785
175	1	0.000000208	-0.000000749	-0.000000634
176	1	0.000000563	0.000000257	-0.000002893
177	1	0.000001496	-0.000001043	-0.000000588
178	1	-0.000000114	0.000000060	0.000000561
179	1	0.000000040	-0.000000537	0.000000582
180	1	0.000000102	0.000000047	-0.000000090
181	8	-0.000002144	0.000000906	-0.000001977
182	8	-0.000000204	0.000001626	0.000003068
183	6	0.000001326	-0.000002977	0.000005207
184	6	-0.000000090	0.000000721	-0.000002753
185	6	0.000002730	-0.000004651	0.000003121
186	6	0.000000548	0.000002597	-0.000000883
187	6	0.000000420	-0.000001821	0.000001838
188	6	-0.000000281	0.000000394	-0.000000742
189	6	-0.000000749	-0.000001558	-0.000000506
190	6	0.000002105	0.000000727	-0.000000164
191	1	0.000000249	-0.000000468	-0.000000658
192	1	-0.000000221	0.000000735	0.000000373
193	1	-0.000000409	0.000001811	-0.000000823

194	1	-0.000000005	-0.000000421	0.000000138
195	1	-0.000000292	-0.000000679	0.000000443
196	1	0.000000440	-0.000000159	0.000000205
197	8	0.000006987	-0.000004792	-0.000008154
198	8	0.000004577	-0.000005477	0.000001539
199	6	-0.000001546	-0.000000581	0.000002134
200	6	0.000000488	-0.000000019	-0.000001399
201	6	0.000004942	-0.000002192	-0.000006196
202	6	-0.000001542	-0.000002827	0.000005524
203	6	-0.000002219	0.000002149	-0.000000869
204	6	0.000001353	0.000000090	0.000000071
205	6	0.000000184	0.000004254	0.000002000
206	6	0.000000509	-0.000000755	-0.000001466
207	1	-0.000000079	0.000001055	0.000000924
208	1	0.000001056	-0.000000192	-0.000000299
209	1	0.000000570	0.000001385	-0.000000304
210	1	0.000000105	0.000000112	0.000000187
211	1	0.000000165	0.000000037	0.000000179
212	1	-0.000000052	0.000000002	0.000000446
213	1	-0.000000170	0.000003436	-0.000001266
214	1	-0.000006497	-0.000002601	0.000007504
215	1	0.000002139	0.000001670	-0.000000812
216	1	0.000001930	-0.000003262	0.000006504
217	1	-0.000001315	-0.000005822	-0.000004044
218	1	-0.000009898	0.000019108	0.000024913
219	1	0.000001893	0.000002475	0.000002710
220	1	0.000003379	-0.000005488	0.000000630
221	1	0.000000518	0.000000561	-0.000000628
222	1	0.000017656	0.000025058	-0.000005406
223	8	0.000002567	0.000001010	-0.000002401
224	8	-0.000004752	-0.000000906	0.000000959
225	6	-0.000001726	-0.000001963	0.000002268
226	6	-0.000001864	0.000000617	-0.000001236
227	6	0.000001758	-0.000000877	-0.000002713
228	6	-0.000000568	-0.000000249	-0.000000087
229	6	0.000000227	0.000000900	0.000000135
230	6	-0.000001953	0.000001176	-0.000004967
231	6	0.000001082	0.000001487	0.000000113
232	6	-0.000000342	0.000000138	0.000000963
233	1	0.000000559	0.000000883	0.000000317
234	1	-0.000000318	-0.000000169	0.000000185
235	1	0.000000277	0.000001088	0.000000242
236	1	0.000000189	-0.000000061	-0.000000160
237	1	0.000000301	-0.000000239	-0.000000121
238	1	0.000000115	-0.000000089	0.000000060
239	8	-0.000000710	0.000000646	-0.000005908
240	8	-0.000001498	0.000004412	-0.000000827
241	6	0.000003401	-0.000002996	0.000001587
242	6	-0.000001058	0.000002206	-0.000001173
243	6	0.000002292	0.000003303	-0.000000884
244	6	-0.000003505	-0.000000123	-0.000000528
245	6	0.000000113	-0.000002502	0.000000015
246	6	0.000000113	0.000004161	-0.000000369
247	6	0.000002123	-0.000004433	-0.000002283
248	6	-0.000000244	0.000001426	0.000000813
249	1	-0.000000510	-0.000001126	-0.000001203
250	1	0.000000097	0.000000279	-0.000000161
251	1	0.000001569	-0.000001335	-0.000000333

252	1	-0.000000023	-0.000000011	0.000000141
253	1	-0.000000041	0.000000199	0.000000118
254	1	0.000000138	0.000000268	0.000000215
255	8	0.000006613	-0.000011558	-0.000012451
256	8	-0.000004195	-0.000008136	-0.000004410
257	6	0.000002614	0.000000583	0.000001131
258	6	-0.000001176	0.000000949	-0.000002482
259	6	-0.000002309	0.000002544	-0.000002104
260	6	0.000002263	-0.000000191	0.000003018
261	6	0.000000184	-0.000001064	-0.000000376
262	6	-0.000001558	-0.000000659	-0.000000205
263	6	0.000001148	-0.000001388	0.000000247
264	6	-0.000000430	0.000000171	-0.000000416
265	1	0.000000590	-0.000000190	0.000000801
266	1	0.000000066	0.000000079	-0.000000220
267	1	0.000001015	-0.000000554	-0.000001089
268	1	-0.000000088	0.000000082	0.000000221
269	1	0.000000046	0.000000032	0.000000263
270	1	-0.000000124	-0.000000029	0.000000211
271	8	0.000002181	-0.000000449	0.000000183
272	8	-0.000006101	0.000000561	0.000001800
273	6	-0.000003361	-0.000001661	0.000002979
274	6	0.000001217	-0.000000047	-0.000001851
275	6	-0.000002228	0.000003613	0.000001974
276	6	0.000001884	-0.000002960	-0.000000391
277	6	-0.000000866	0.000000713	-0.000000427
278	6	-0.000001246	-0.000000409	0.000001966
279	6	-0.000000904	-0.000000347	-0.000000141
280	6	0.000000269	-0.000000155	-0.000000348
281	1	0.000000326	-0.000000301	0.000000738
282	1	-0.000000203	-0.000000168	0.000000026
283	1	-0.000000202	-0.000000143	-0.000000333
284	1	0.000000010	-0.000000172	-0.000000011
285	1	0.000000166	-0.000000061	-0.000000325
286	1	0.000000081	0.000000009	-0.000000012
287	1	-0.000001871	0.000005145	0.000001332
288	1	0.000003489	-0.000005878	-0.000003776
289	1	0.000002560	0.000004743	-0.000001153
290	1	-0.000000355	0.000000763	-0.000000151
291	1	0.000000780	-0.000014567	-0.000005036
292	1	0.000000460	0.000000070	0.000000455
293	1	-0.000000526	0.000001740	0.000002327
294	1	0.000000522	0.000003901	0.000000113
295	1	-0.000005147	-0.000000869	-0.000004276
296	1	-0.002885497	-0.000794487	0.003006641
297	8	-0.000000192	-0.000002952	-0.000003412
298	8	-0.000003291	0.000008760	0.000001467
299	6	0.000003108	-0.000002677	0.000002326
300	6	-0.000002542	0.000002699	0.000000792
301	6	0.000002874	-0.000006030	0.000001198
302	6	0.000001128	-0.000003224	0.000000024
303	6	-0.000001985	0.000000915	0.000000355
304	6	0.000000781	-0.000000678	-0.000001530
305	6	0.000000869	0.000001535	0.000000520
306	6	0.000000305	0.000000145	0.000000448
307	1	0.000000281	0.000000516	-0.000000017
308	1	0.000001138	0.000000977	0.000000578
309	1	0.000000764	-0.000000241	0.000000090

310	1	-0.000000033	0.000000055	-0.000000436
311	1	-0.000000048	-0.000000265	-0.000000193
312	1	-0.000000267	-0.000000287	-0.000000387
313	8	-0.000003066	-0.000008276	0.000000194
314	8	-0.000000361	-0.000004916	-0.000002891
315	6	0.000001471	0.000002160	-0.000001816
316	6	0.000001417	-0.000001041	0.000002163
317	6	0.000000906	0.000004217	-0.000003847
318	6	0.000001052	0.000001839	-0.000003047
319	6	-0.000002857	-0.000000123	-0.000002013
320	6	0.000001326	0.000000125	0.000002922
321	6	0.000000488	-0.000001057	0.000001049
322	6	0.000001043	0.000000124	0.000000027
323	1	0.000000418	-0.000000199	-0.000000559
324	1	0.000000548	-0.000000720	-0.000000437
325	1	0.000000666	-0.000000708	0.000000469
326	1	0.000000092	-0.000000177	-0.000000072
327	1	-0.000000101	-0.000000156	0.000000125
328	1	-0.000000054	-0.000000030	-0.000000029
329	8	-0.000000138	0.000001180	0.000000902
330	8	0.000002705	-0.000000028	-0.000004036
331	6	-0.000004082	0.000000375	0.000002822
332	6	0.000001543	-0.000000522	-0.000001111
333	6	-0.000002300	0.000002755	0.000003321
334	6	-0.000001604	-0.000000161	0.000000514
335	6	0.000001818	0.000001267	-0.000000135
336	6	0.000000544	-0.000000272	-0.000000601
337	6	-0.000000368	0.000000030	-0.000000048
338	6	-0.000000403	0.000000613	0.000000291
339	1	0.000000219	-0.000000108	0.000000126
340	1	-0.000000543	0.000000063	-0.000000473
341	1	-0.000000318	-0.000000056	0.000000325
342	1	0.000000257	0.000000002	0.000000085
343	1	0.000000256	-0.000000378	0.000000278
344	1	0.000000168	-0.000000419	-0.000000058
345	8	-0.000000317	-0.000003736	-0.000001219
346	8	0.000005991	0.000004760	0.000009131
347	6	-0.000001652	-0.000003901	-0.000002140
348	6	-0.000000283	0.000003642	0.000000463
349	6	-0.000005056	-0.000006428	-0.000005932
350	6	-0.000001213	-0.000000368	-0.000001031
351	6	0.000004444	-0.000003482	0.000001972
352	6	-0.000002574	0.000003127	-0.000001058
353	6	-0.000000569	0.000000445	0.000001431
354	6	-0.000001110	-0.000000234	0.000000337
355	1	0.000001124	-0.000002510	-0.000000612
356	1	-0.000001816	0.000000006	0.000000436
357	1	-0.000001087	-0.000000026	-0.000000638
358	1	0.000000054	0.000000206	-0.000000118
359	1	0.000000111	0.000000004	-0.000000054
360	1	0.000000271	0.000000311	0.000000001
361	1	-0.000004697	0.000006021	0.000004422
362	1	-0.000010640	-0.000028058	-0.000024068
363	1	-0.000010547	0.000004983	-0.000013095
364	1	0.000016452	0.000014135	-0.000004407
365	1	0.000001458	-0.000000085	0.000003835
366	1	0.000001220	-0.000002700	-0.000003842
367	1	-0.000002252	0.000000585	-0.000000110

368	1	0.000000637	-0.000000416	0.000000519
369	1	-0.000000463	0.000003049	-0.000003750
370	1	-0.000003559	-0.000006529	0.000007964
371	8	0.000005965	-0.000023358	-0.000007843
372	8	0.000012756	0.000017615	0.000017106
373	6	0.000002443	0.000009887	-0.000005408
374	6	-0.000002729	-0.000002277	0.000001241
375	6	-0.000000815	0.000001302	-0.000001543
376	6	-0.000003327	-0.000000451	0.000000560
377	6	-0.000000801	0.000001566	0.000001432
378	6	0.000002082	-0.000001074	0.000001183
379	6	0.000002777	-0.000000693	0.000001746
380	6	-0.000001341	0.000000873	-0.000000826
381	1	-0.000000146	-0.000000655	-0.000001652
382	1	0.000000812	-0.000000330	0.000000614
383	1	0.000001210	0.000000070	-0.000000253
384	1	0.000000424	-0.000000545	0.000000253
385	1	0.000000469	0.000000092	0.000000499
386	1	0.000000421	0.000000050	-0.000000153
387	8	0.000004079	0.000001343	-0.000013178
388	8	-0.000010805	-0.000006040	-0.000007957
389	6	-0.000004526	-0.000005381	-0.000003129
390	6	0.000000308	0.000000209	-0.000000715
391	6	0.000002169	-0.000004055	0.000006059
392	6	-0.000002112	0.000000947	-0.000000147
393	6	0.000000698	-0.000001429	0.000000471
394	6	0.000002034	0.000003040	0.000003749
395	6	0.000004103	0.000000161	-0.000000546
396	6	-0.000001174	0.000000687	-0.000000320
397	1	-0.000001534	0.000000376	-0.000000382
398	1	0.000000724	0.000000374	-0.000000184
399	1	0.000001334	0.000000406	0.000000027
400	1	-0.000000024	-0.000000049	-0.000000089
401	1	0.000000177	-0.000000174	-0.000000042
402	1	0.000000292	-0.000000201	0.000000291
403	8	-0.000000493	-0.000007951	-0.000009288
404	8	-0.000004611	0.000002743	-0.000002887
405	6	-0.000008988	-0.000001454	0.000012032
406	6	0.000000088	-0.000001807	-0.000002988
407	6	0.000003470	0.000002688	0.000007961
408	6	-0.000005049	-0.000004043	-0.000004487
409	6	0.000000743	0.000002100	0.000006986
410	6	0.000001516	0.000002721	-0.000007248
411	6	0.000001414	-0.000001950	-0.000000971
412	6	0.000000836	-0.000000104	-0.000000442
413	1	0.000000233	0.000000897	-0.000001621
414	1	0.000000699	-0.000000574	-0.000000805
415	1	0.000001220	0.000000483	0.000000356
416	1	-0.000000044	-0.000000549	-0.000000090
417	1	-0.000000133	0.000000019	-0.000000154
418	1	-0.000000051	-0.000000262	0.000000035
419	8	-0.000000977	0.000004154	-0.000001161
420	8	-0.000002963	-0.000003474	0.000003421
421	6	0.000000818	-0.000000853	0.000000708
422	6	-0.000000974	0.000000214	0.000002991
423	6	0.000001366	-0.000000653	-0.000003638
424	6	0.000000516	0.000001569	0.000001653
425	6	0.000000364	0.000000648	0.000002176

426	6	-0.000004820	0.000001429	-0.000001338
427	6	-0.000002919	0.000002164	0.000000094
428	6	0.000000637	-0.000000929	-0.000000821
429	1	0.000000098	-0.000000157	-0.000000435
430	1	0.000000205	-0.000000253	0.000000054
431	1	0.000000131	-0.000001970	-0.000000646
432	1	-0.000000014	0.000000295	-0.000000014
433	1	0.000000131	0.000000251	0.000000129
434	1	0.000000092	0.000000224	0.000000172
435	1	-0.000000006	0.000000676	0.000001797
436	1	0.000019801	-0.000007524	0.000001647
437	1	0.000010564	0.000008253	0.000001639
438	1	-0.000005858	0.000000447	-0.000005719
439	1	0.000013869	-0.000006746	-0.000001224
440	1	-0.000007646	-0.000008085	-0.000003733
441	1	0.000000938	-0.000000463	-0.000001736
442	1	0.000001339	-0.000002190	0.000001863
443	1	-0.000003877	-0.000004792	-0.000001602
444	1	-0.000000923	-0.000000136	-0.000001061
445	6	-0.000450686	0.000430779	-0.002098376
446	6	0.000142486	0.000680993	-0.001546027
447	6	-0.000092949	-0.000154145	0.000961539
448	6	0.000640144	-0.000603864	0.004606309
449	6	-0.000116709	-0.000210552	0.000048637
450	6	0.000206583	0.000472790	-0.000013025
451	1	-0.002005882	-0.004215664	-0.004219210
452	1	-0.001482856	-0.005530777	0.002288986
453	1	0.002111570	0.004500918	0.003862629
454	1	0.001593286	0.005727007	-0.001868932
455	17	0.000737095	0.003363626	-0.000923034
456	1	0.000000965	0.000001049	0.000004255
457	1	-0.000003546	-0.000001074	-0.000000498
458	1	-0.000001738	-0.000003038	0.000002698
459	8	0.000003501	-0.000004608	-0.000004672
460	1	0.000001668	0.000001905	-0.000001440
461	1	-0.000004566	-0.000003358	0.000005783
462	8	0.000000964	-0.000013201	-0.000004056
463	1	-0.000005559	0.000021192	0.000006222
464	1	0.000003122	0.000006279	0.000005768
465	8	0.000016802	-0.000007042	-0.000001377
466	1	-0.000006930	-0.000003624	-0.000002566
467	1	-0.000008004	0.000009779	0.000006476
468	6	0.000973651	0.003358995	0.001915235
469	8	-0.001966013	-0.006512119	-0.004607977
470	6	-0.002731866	-0.002522195	-0.003810051
471	1	-0.000716758	-0.003964687	0.005151118
472	1	-0.003136188	0.005147500	0.001060199
473	1	0.005801582	0.001665317	-0.002060555
474	8	-0.000941986	-0.002427205	0.002384462

Sum of electronic and thermal Free Energies= -1149.510470
0 imaginary frequency

3f@C_R

Center Number	Atomic Number	Integrated Forces (Hartrees/Bohr) X	Y	Z

1	8	-0.000012223	0.000556327	-0.000609398
2	8	-0.003836927	-0.006697376	-0.000917551
3	6	0.000014878	-0.000324738	0.000258469
4	6	-0.000000179	0.000298601	-0.000644207
5	6	0.001627306	0.004260222	0.001728657
6	6	-0.000071071	-0.000081192	0.000435526
7	6	-0.000178078	-0.000000563	0.000198999
8	6	0.000059419	0.000093378	0.000268237
9	6	-0.000116210	0.000333354	-0.000298500
10	6	-0.000119020	-0.000350727	0.001271223
11	1	-0.000125474	-0.000063574	0.000349080
12	1	0.000297968	0.000005939	-0.000775972
13	1	0.000172672	0.000024260	0.000037339
14	1	0.000122790	0.000201302	-0.000380072
15	1	0.000103061	-0.000096124	-0.000476830
16	1	-0.000071907	0.000149773	-0.000344498
17	8	0.000665257	0.000388423	0.000015493
18	8	-0.000970484	0.002483946	-0.001232555
19	6	-0.001004344	-0.000567279	-0.000373219
20	6	-0.000189029	0.001137024	-0.000250878
21	6	0.000133599	-0.002019514	0.000319344
22	6	-0.000055121	0.000618296	0.000244110
23	6	0.000006888	-0.000471653	-0.000071377
24	6	0.000630164	-0.000162777	0.000511781
25	6	-0.000194667	-0.000037382	-0.000476913
26	6	0.000665478	-0.000425282	0.000990657
27	1	0.000220615	-0.000442215	0.000008403
28	1	-0.000519072	0.000379441	-0.000385349
29	1	-0.000091058	-0.000130294	0.000010546
30	1	-0.000290586	0.000298178	-0.000273250
31	1	-0.000096539	0.000123321	-0.000286386
32	1	-0.000240182	0.000032428	-0.000307288
33	8	0.000970092	-0.001041856	-0.000301287
34	8	0.000058710	-0.000030448	0.000408663
35	6	-0.000540913	0.000706833	0.000192073
36	6	0.000112473	-0.000523277	-0.000222863
37	6	-0.000605884	0.000368527	-0.000497364
38	6	0.000411216	-0.000214448	0.000816762
39	6	-0.000043756	0.000119789	0.000208873
40	6	0.000167437	-0.000340968	0.000459312
41	6	0.000112577	0.000190621	-0.000407684
42	6	0.000668598	-0.000441699	0.001059906
43	1	0.000216870	0.000227789	0.000170946
44	1	-0.000362418	-0.000031185	-0.000831998
45	1	-0.000119719	0.000036331	0.000058198
46	1	-0.000183616	0.000247957	-0.000264256
47	1	-0.000127125	0.000107662	-0.000362832
48	1	-0.000355857	0.000053689	-0.000318575
49	8	-0.000752914	0.001046827	0.001054495
50	8	0.000265226	0.000094616	-0.000263361
51	6	0.000275799	-0.000920917	-0.000524175
52	6	0.000301683	0.000856111	0.000019622
53	6	-0.000495573	-0.000293946	-0.000252436
54	6	0.000026915	-0.000160807	0.000641701
55	6	0.000140503	-0.000202594	-0.000003614
56	6	0.000071688	0.000172863	0.000503366
57	6	-0.000177989	-0.000138226	-0.000462979

58	6	-0.000178876	-0.000268392	0.001270777
59	1	-0.000243049	-0.000429587	0.000228583
60	1	-0.000069054	0.000623642	-0.000592509
61	1	0.000118592	-0.000076548	-0.000018595
62	1	-0.000021889	0.000133792	-0.000323148
63	1	0.000261034	0.000177205	-0.000407144
64	1	0.000009756	-0.000000669	-0.000428400
65	8	0.000234511	-0.000254659	-0.000044074
66	8	-0.000307662	-0.002829219	0.001450323
67	6	-0.000680080	0.000365751	0.000554599
68	6	-0.000067616	-0.001168574	0.000338684
69	6	-0.000098641	0.002197287	-0.000274097
70	6	-0.000252624	-0.000638867	-0.000328323
71	6	-0.000045165	0.000477721	0.000034053
72	6	0.000342166	0.000225374	-0.000736045
73	6	0.000077499	-0.000038232	0.000530048
74	6	0.000087353	0.000548572	-0.001147743
75	1	0.000147378	0.000469663	-0.000184878
76	1	-0.000353152	-0.000410491	0.000629625
77	1	-0.000092334	0.000128422	-0.000000362
78	1	-0.000051255	-0.000071627	0.000396065
79	1	-0.000103220	-0.000386857	0.000308780
80	1	0.000065693	-0.000123056	0.000304345
81	8	0.000348346	-0.000770972	0.000271815
82	8	0.000410054	0.000358655	0.001652919
83	6	-0.000022930	0.000481014	0.000130326
84	6	0.000237622	0.000314424	-0.000130831
85	6	0.000237839	-0.000475629	-0.000521913
86	6	-0.000278380	0.000187371	-0.000375634
87	6	-0.000353802	0.000072060	0.000000327
88	6	-0.000110799	0.000002438	-0.000345695
89	6	0.000088928	-0.000454902	0.000281885
90	6	-0.000610899	0.000568396	-0.001117749
91	1	-0.000264037	-0.000227570	-0.000080826
92	1	0.000625620	-0.000079955	0.000637853
93	1	0.000125087	-0.000073155	-0.000119395
94	1	0.000063788	-0.000185150	0.000331667
95	1	0.000236416	-0.000274489	0.000282608
96	1	0.000299027	0.000035686	0.000458479
97	8	0.000527611	0.001345226	-0.000141558
98	8	-0.000380521	0.000169884	-0.000273317
99	6	-0.000235039	-0.000825192	0.000139382
100	6	-0.000053606	0.000707994	0.000243143
101	6	-0.000090648	-0.000428386	0.000424448
102	6	-0.000019821	0.000277094	-0.000672055
103	6	0.000017672	-0.000042329	-0.000241433
104	6	-0.000134698	0.000383186	-0.000525873
105	6	0.000270389	-0.000215963	0.000366268
106	6	0.000164644	0.000568945	-0.001252204
107	1	0.000180557	-0.000345478	-0.000193452
108	1	0.000029744	-0.000068037	0.000840811
109	1	-0.000103850	-0.000059295	-0.000052792
110	1	-0.000261109	-0.000118630	0.000448098
111	1	-0.000026918	-0.000301015	0.000328490
112	1	0.000040494	-0.000138046	0.000399245
113	8	-0.001448676	-0.001262083	-0.000430496
114	8	0.000165757	0.000251387	-0.000106331
115	6	0.000837260	0.001194644	0.000241377

116	6	-0.000188733	-0.000623662	-0.000202403
117	6	0.000341832	-0.000203805	0.000330820
118	6	-0.000432994	0.000257613	-0.000814214
119	6	0.000198936	0.000317046	0.000000918
120	6	-0.000157485	-0.000121801	-0.000296671
121	6	0.000034770	0.000077016	0.000593296
122	6	-0.000651623	0.000374763	-0.001205123
123	1	-0.000059857	0.000254675	0.000033656
124	1	0.000287368	-0.000697307	0.000458612
125	1	0.000036239	0.000089282	0.000018375
126	1	0.000167024	-0.000032054	0.000428516
127	1	0.000090684	-0.000151318	0.000315107
128	1	0.000382362	-0.000127898	0.000262417
129	8	0.000198808	0.000033289	-0.001237920
130	1	0.000047102	0.000069100	0.000551953
131	1	-0.000087343	-0.000121122	0.000435475
132	8	0.000357398	-0.000483147	0.000238451
133	1	-0.000022162	-0.000943852	-0.000482059
134	1	0.000889504	-0.000298418	0.000407934
135	8	0.006469137	-0.001223547	0.006050412
136	1	-0.004059664	-0.002306831	-0.004913805
137	1	-0.003004577	0.002554768	-0.001117540
138	8	0.000294988	0.000507146	-0.000143292
139	1	-0.000152622	0.000900081	0.000269454
140	1	0.000704453	0.000286259	-0.000736795
141	8	0.002310482	-0.004185513	-0.008283153
142	1	-0.000951806	0.004041188	0.004393718
143	1	-0.001888874	-0.000244738	0.005476361
144	1	-0.000163517	-0.000236153	-0.000327975
145	1	-0.000559869	0.000120297	-0.000068206
146	1	-0.000107436	-0.000379510	0.000148086
147	1	-0.000170163	0.000542266	-0.000450968
148	1	-0.000048145	-0.000306887	-0.000405202
149	8	0.000075552	0.000466280	0.000215710
150	8	-0.002448978	0.001359931	0.000733380
151	6	0.000201855	0.000105975	-0.000308650
152	6	-0.000877386	0.000762852	0.000341607
153	6	0.002272114	-0.001262110	-0.001016585
154	6	-0.000676199	0.000210261	-0.000103740
155	6	0.000464931	-0.000074920	-0.000141577
156	6	-0.000152141	-0.000637180	-0.000076485
157	6	0.000121050	0.000295746	0.000487013
158	6	0.000222773	-0.001058931	-0.000706455
159	1	0.000383778	-0.000338916	-0.000091823
160	1	-0.000223873	0.000769554	0.000004884
161	1	0.000012054	-0.000102730	0.000107084
162	1	0.000029232	0.000345527	0.000093030
163	1	-0.000265782	0.000367321	0.000135566
164	1	-0.000026725	0.000290247	0.000320346
165	8	-0.000159885	0.000362497	0.000427723
166	8	0.001282309	0.000928237	-0.000072274
167	6	0.000026580	0.000015579	-0.000077423
168	6	0.000301084	0.000593158	0.000346720
169	6	-0.000931933	-0.000501343	0.000155291
170	6	0.000138560	-0.000421373	-0.000121077
171	6	-0.000129399	0.000005561	-0.000177307
172	6	-0.000056740	-0.000537375	-0.000132713
173	6	-0.000131346	0.000543027	0.000051676

174	6	-0.000370343	-0.001208044	-0.000465751
175	1	-0.000248218	-0.000258368	-0.000214140
176	1	0.000346461	0.000539402	0.000648824
177	1	-0.000006044	0.000022719	-0.000101753
178	1	0.000082998	0.000422409	0.000037183
179	1	0.000002412	0.000288426	0.000202786
180	1	0.000395393	0.000281557	0.000221209
181	8	-0.000231790	0.001190600	-0.000732577
182	8	0.000413127	-0.000435110	0.000514801
183	6	0.000348853	-0.000436557	0.000392005
184	6	-0.000214562	0.000207308	0.000019899
185	6	-0.000051735	0.000402914	-0.000077898
186	6	0.000014923	-0.000538811	-0.000406732
187	6	0.000222572	-0.000094775	-0.000073890
188	6	-0.000054458	-0.000332034	-0.000430395
189	6	0.000066482	0.000529974	0.000029069
190	6	-0.000011763	-0.001071460	-0.000724629
191	1	0.000105671	-0.000108082	-0.000124562
192	1	-0.000431026	0.000549997	0.000563673
193	1	0.000077650	0.000003106	-0.000040130
194	1	-0.000064249	0.000233725	0.000447773
195	1	0.000097064	0.000310864	0.000172810
196	1	-0.000066268	0.000346794	0.000102344
197	8	0.000275097	0.000325153	0.001004447
198	8	-0.001080336	0.000648957	-0.000538173
199	6	-0.000148358	0.000176352	-0.000498653
200	6	0.000021534	0.000002555	0.000249565
201	6	0.000485192	-0.000016059	0.000103261
202	6	-0.000335273	-0.000487207	-0.000245095
203	6	-0.000167435	-0.000196851	0.000214893
204	6	-0.000197184	-0.000614234	-0.000171124
205	6	0.000292747	0.000259954	0.000389664
206	6	-0.000362561	-0.001231771	-0.000327843
207	1	-0.000087853	-0.000019521	-0.000071437
208	1	0.000485198	0.000811014	0.000005213
209	1	-0.000053852	-0.000007756	-0.000020169
210	1	0.000173815	0.000469861	-0.000104177
211	1	-0.000026470	0.000352212	0.000134444
212	1	0.000160711	0.000331631	0.000207382
213	1	0.000707482	-0.000276909	0.000617913
214	1	0.000033905	0.000383348	-0.000259393
215	1	-0.000157531	0.000571051	-0.000888465
216	1	0.000016490	-0.000033590	0.000245851
217	1	0.000460176	-0.000570223	-0.000682847
218	1	-0.000225975	0.000187168	0.000170501
219	1	0.000229593	0.000236808	-0.000739621
220	1	-0.000207703	0.000291034	-0.000081204
221	1	-0.000035658	-0.000478515	0.000352505
222	1	-0.000273890	0.000066510	0.000642683
223	8	0.000053785	-0.000549975	-0.000000039
224	8	-0.001945657	-0.001431810	0.000372095
225	6	0.000245944	-0.000027439	0.000069703
226	6	-0.000831758	-0.000765291	0.000069930
227	6	0.002125068	0.001279787	0.000095514
228	6	-0.000438212	-0.000276622	0.000426535
229	6	0.000372585	0.000102357	-0.000155564
230	6	-0.000180154	0.000533895	0.000213655
231	6	-0.000147072	-0.000193200	-0.000574385

232	6	0.000493030	0.000997891	0.000676944
233	1	0.000332580	0.000389783	-0.000059164
234	1	-0.000055194	-0.000630855	0.000046498
235	1	-0.000086257	0.000113454	-0.000096154
236	1	-0.000314562	-0.000440141	-0.000129807
237	1	-0.000142173	-0.000288160	-0.000332116
238	1	-0.000015945	-0.000318897	-0.000132452
239	8	-0.000072787	-0.000180946	-0.000008790
240	8	0.001637450	0.000086621	-0.000635948
241	6	-0.000199910	-0.000147889	-0.000380129
242	6	0.000428229	-0.000023952	-0.000317765
243	6	-0.001151650	-0.000016919	0.000215958
244	6	0.000224039	0.000411307	0.000078235
245	6	-0.000258848	0.000005724	0.000207491
246	6	0.000095881	0.000577775	0.000484759
247	6	-0.000205696	-0.000516837	-0.000089156
248	6	-0.000214538	0.001156636	0.000649176
249	1	-0.000167813	-0.000082623	0.000189117
250	1	0.000125956	-0.000490308	-0.000708982
251	1	0.000043283	-0.000037724	0.000100739
252	1	0.000076485	-0.000391132	-0.000081819
253	1	-0.000055521	-0.000309432	-0.000199107
254	1	0.000272202	-0.000326207	-0.000308143
255	8	-0.000153050	-0.000168710	-0.000297535
256	8	-0.000441352	-0.000844820	0.000922802
257	6	0.000489190	0.000022588	-0.000211212
258	6	-0.000043474	-0.000526705	-0.000007938
259	6	0.000126053	0.000414989	-0.000545565
260	6	0.000019864	0.000137696	0.000529431
261	6	-0.000100528	0.000316364	-0.000288548
262	6	-0.000332436	0.000592138	0.000402831
263	6	0.000157451	-0.000240612	-0.000451233
264	6	-0.000281757	0.001143284	0.000452183
265	1	-0.000117090	0.000247691	0.000030339
266	1	0.000414781	-0.000600429	-0.000280112
267	1	-0.000061583	-0.000004413	0.000025526
268	1	0.000091195	-0.000305342	-0.000265894
269	1	0.000217970	-0.000451785	-0.000000185
270	1	-0.000051737	-0.000358232	-0.000118205
271	8	0.000264516	-0.001147705	0.000753467
272	8	0.000413210	0.000281658	-0.000184820
273	6	0.000026461	0.000527641	-0.000505556
274	6	-0.000122610	-0.000286771	0.000239231
275	6	-0.000210021	-0.000420304	-0.000364999
276	6	0.000265227	0.000626716	0.000481597
277	6	0.000316017	0.000107227	-0.000185853
278	6	0.000104328	0.000185889	0.000429145
279	6	0.000096620	-0.000523036	-0.000015212
280	6	0.000222461	0.001082522	0.000717953
281	1	0.000115497	0.000113550	0.000022375
282	1	-0.000576729	-0.000586455	-0.000345860
283	1	0.000071911	0.000009062	-0.000038207
284	1	0.000041412	-0.000314721	-0.000212872
285	1	-0.000099065	-0.000340141	-0.000085926
286	1	-0.000252055	-0.000246972	-0.000367859
287	1	0.000164939	-0.000511202	-0.000516124
288	1	-0.002800769	-0.001218892	-0.000835447
289	1	0.000298990	0.000525225	0.000382962

290	1	-0.002395879	0.000934222	0.001587032
291	1	-0.001037426	-0.000136273	-0.001491791
292	1	0.000681546	0.000851430	0.000925951
293	1	-0.001130725	0.000958944	-0.000600777
294	1	0.000240198	-0.000860141	-0.001079155
295	1	-0.000812745	-0.000821050	0.000824207
296	1	0.002265424	0.002251637	-0.001160675
297	8	-0.000330121	0.000494224	0.000493098
298	8	0.002865696	0.000172534	0.001833787
299	6	0.000358869	0.000043011	-0.000499142
300	6	0.001163855	0.000143514	0.000949342
301	6	-0.001458968	0.000434465	-0.001537543
302	6	-0.000031745	0.000031988	0.000530780
303	6	-0.000103996	0.000065209	-0.000330437
304	6	-0.000627377	-0.000169074	0.000255104
305	6	0.000512123	-0.000188258	-0.000312995
306	6	-0.001298577	0.000072268	0.000123830
307	1	-0.000671418	-0.000182387	-0.000454518
308	1	0.000775777	0.000315907	0.000155501
309	1	-0.000065432	0.000086820	-0.000093081
310	1	0.000354895	0.000093684	-0.000083838
311	1	0.000429869	-0.000022076	0.000197172
312	1	0.000360829	-0.000096588	-0.000127100
313	8	-0.000484826	-0.000518585	-0.000313073
314	8	0.001911979	-0.000062560	-0.002426235
315	6	0.000504796	0.000046410	0.000388830
316	6	0.000770388	-0.000093746	-0.001238510
317	6	-0.000732450	-0.000462846	0.001747462
318	6	-0.000335861	-0.000157281	-0.000483983
319	6	0.000040423	-0.000060499	0.000391732
320	6	-0.000711978	0.000130077	-0.000062133
321	6	0.000598850	0.000238260	0.000092213
322	6	-0.001243085	-0.000188140	0.000343686
323	1	-0.000446934	0.000067449	0.000658445
324	1	0.000639805	-0.000246450	-0.000365071
325	1	-0.000004198	-0.000099406	0.000135628
326	1	0.000351856	0.000114177	0.000001416
327	1	0.000330488	-0.000052027	-0.000061557
328	1	0.000356532	0.000071158	-0.000358128
329	8	-0.000721374	-0.001127203	-0.001868182
330	8	0.000571470	-0.001139128	0.000666720
331	6	0.000303906	0.000649612	0.001464813
332	6	-0.000060174	-0.000200656	-0.000567510
333	6	0.000405069	0.001124976	0.000069039
334	6	-0.000940841	-0.000599303	0.000374072
335	6	0.000050770	0.000157200	0.000548877
336	6	-0.000397597	-0.000136018	-0.000325686
337	6	0.000434254	-0.000183291	-0.000364455
338	6	-0.001214420	-0.000380626	0.000511041
339	1	-0.000002718	-0.000133375	0.000076320
340	1	0.000690535	0.000387153	-0.000491202
341	1	-0.000002590	0.000033797	-0.000168569
342	1	0.000339469	0.000021191	-0.000069641
343	1	0.000370862	0.000069911	-0.000241580
344	1	0.000395382	0.000357424	-0.000120593
345	8	0.000145851	0.001219907	0.001888231
346	8	0.000302693	0.001047418	-0.000782805
347	6	-0.000326125	-0.000672398	-0.001343978

348	6	0.000335056	0.000210824	0.000510138
349	6	0.000311609	-0.001073572	-0.000190483
350	6	-0.000908851	0.000426577	-0.000075260
351	6	-0.000202892	-0.000156717	-0.000374733
352	6	-0.000352409	0.000226405	0.000467343
353	6	0.000515775	0.000235754	0.000191659
354	6	-0.001340106	0.000255555	-0.000035848
355	1	-0.000236367	0.000115233	-0.000080430
356	1	0.000775578	-0.000332584	0.000201292
357	1	0.000058384	-0.000044607	0.000194006
358	1	0.000405919	-0.000321961	-0.000029793
359	1	0.000326343	0.000015074	-0.000075686
360	1	0.000435644	-0.000032394	0.000084065
361	1	-0.000292625	0.000245105	0.000245778
362	1	0.001206051	0.000786856	-0.000234205
363	1	-0.000106350	-0.000107733	-0.000016383
364	1	0.000869829	-0.000916731	-0.000149750
365	1	-0.000434249	0.000276896	-0.000497480
366	1	-0.000311065	-0.000846903	0.000102207
367	1	0.000574208	-0.000047526	0.000290966
368	1	-0.000478672	-0.000690948	0.000207697
369	1	-0.000005199	0.000063474	0.000313634
370	1	0.001550689	-0.001605323	0.001637924
371	8	0.000497256	0.000121587	0.000087393
372	8	-0.000868827	-0.000377076	0.000873426
373	6	-0.000486063	-0.000389945	-0.000445467
374	6	-0.000484936	-0.000337043	0.000391561
375	6	0.000282963	0.000011483	-0.000499270
376	6	0.000220941	0.000113883	0.000159605
377	6	0.000083468	0.000107488	-0.000189816
378	6	0.000604110	0.000310729	0.000070198
379	6	-0.000491608	0.000014078	-0.000143868
380	6	0.001155030	0.000408189	-0.000248883
381	1	0.000289975	0.000288314	-0.000208605
382	1	-0.000596198	-0.000494715	0.000099201
383	1	0.000017259	-0.000091609	-0.000134705
384	1	-0.000316639	0.000007906	0.000087472
385	1	-0.000382553	-0.000145581	-0.000029016
386	1	-0.000302816	-0.000275426	0.000280914
387	8	0.000346663	-0.000077516	0.000608630
388	8	-0.000475118	0.000332673	0.000102431
389	6	-0.000140064	-0.000000845	-0.000094207
390	6	-0.000431485	-0.000134106	0.000042395
391	6	0.000006963	0.000483099	-0.000214754
392	6	0.000570223	-0.000221666	0.000012449
393	6	0.000061918	-0.000111292	-0.000289825
394	6	0.000367899	0.000040762	0.000108555
395	6	-0.000437154	-0.000259100	0.000139376
396	6	0.001301259	-0.000182299	-0.000065216
397	1	0.000166007	-0.000193569	0.000148205
398	1	-0.000662236	0.000412380	0.000084796
399	1	0.000045126	0.000084717	0.000092591
400	1	-0.000392040	-0.000075472	0.000031243
401	1	-0.000365565	0.000069285	0.000140848
402	1	-0.000418771	0.000216677	-0.000139378
403	8	0.000114508	-0.001172102	0.000393055
404	8	-0.000570264	-0.000346824	-0.001189146
405	6	0.000479781	0.001163256	-0.000395954

406	6	-0.000690974	-0.000522393	-0.000261854
407	6	0.000020657	0.000965357	0.000987695
408	6	0.000601370	-0.000202062	-0.000556616
409	6	0.000079907	0.000443119	-0.000155324
410	6	0.000256890	-0.000330048	0.000253272
411	6	-0.000468956	-0.000148671	-0.000072797
412	6	0.001152026	-0.000100559	-0.000476528
413	1	0.000361338	0.000028466	0.000055686
414	1	-0.000617821	-0.000058727	0.000585991
415	1	0.000032227	-0.000100079	0.000112941
416	1	-0.000338133	-0.000067206	0.000161264
417	1	-0.000307603	0.000237455	0.000319144
418	1	-0.000351481	0.000017387	0.000006104
419	8	0.000407829	0.000396637	-0.000211746
420	8	0.000263865	0.000201584	0.000526740
421	6	-0.000559345	-0.000037483	-0.000329542
422	6	-0.000201507	0.000347236	0.000453187
423	6	-0.000571438	-0.000749890	-0.000885465
424	6	0.000877899	0.000187815	0.000305460
425	6	0.000235284	-0.000301942	0.000039112
426	6	0.000488269	0.000201553	0.000045225
427	6	-0.000413771	0.000198761	0.000195596
428	6	0.001301615	0.000223666	0.000006493
429	1	0.000079020	-0.000029056	0.000054926
430	1	-0.000952518	-0.000033649	-0.000343174
431	1	-0.000027625	0.000065349	-0.000115181
432	1	-0.000415881	-0.000249741	-0.000127138
433	1	-0.000329891	-0.000084702	0.000145744
434	1	-0.000437295	0.000049739	-0.000017653
435	1	-0.000593211	-0.000447318	0.000280991
436	1	-0.000477152	0.000648441	0.000060149
437	1	0.000356174	-0.000066487	-0.000402132
438	1	-0.000124284	-0.000155366	-0.000132391
439	1	0.001132688	0.000063331	0.000948864
440	1	0.000709429	0.001855820	-0.002050240
441	1	0.000811420	0.000515737	0.000368500
442	1	-0.000426296	0.000179404	-0.000290990
443	1	-0.000366992	-0.000263344	-0.000313533
444	1	-0.000305117	-0.000021703	0.000250555
445	6	0.000342727	-0.003128472	0.001798224
446	6	-0.000083880	-0.000110196	0.000442428
447	6	-0.000075202	-0.000379690	-0.000407224
448	6	-0.001005649	0.005560520	-0.003263179
449	6	-0.000108962	-0.000038228	0.000513816
450	6	-0.000096072	0.000037205	-0.000165530
451	1	0.000476325	-0.005993858	-0.002896274
452	1	-0.000230750	0.001024523	-0.006767322
453	1	-0.000157597	0.006420580	0.002594333
454	1	0.000783988	-0.000397058	0.006518230
455	17	0.000239434	-0.000011596	0.003252398
456	1	0.000024868	-0.000208966	0.000047309
457	1	0.000455756	-0.000441704	0.000614990
458	1	0.000331727	0.000242280	-0.000072356
459	8	0.001892811	-0.001675658	0.000849101
460	1	-0.000862452	0.000485564	-0.000683581
461	1	-0.000732827	0.000946390	-0.000020783
462	8	-0.002002077	-0.000419748	-0.001395249
463	1	0.000914850	0.000644988	0.000572699

464	1	0.000765288	0.000003044	0.000576225
465	8	0.001175949	0.001793942	-0.001408508
466	1	-0.000406266	-0.000476643	0.000923933
467	1	-0.000579248	-0.001089694	0.000247645
468	6	0.001046444	0.003169542	0.003648072
469	8	-0.000697500	-0.006340475	-0.005358916
470	7	0.000540404	0.002239224	0.000405221
471	8	0.000176693	0.001196947	0.002653358
472	8	0.000120667	-0.001909900	-0.003429647

Sum of electronic and thermal Free Energies= -1239.595755
0 imaginary frequency

[2+3a]@C_R

Center Number	Atomic Number	Integrated Forces (Hartrees/Bohr) X	Y	Z
1	6	-0.000566147	0.000153917	0.001613080
2	6	0.000615441	0.001334442	0.000447932
3	7	0.002576951	0.003088330	-0.002509503
4	6	0.001295032	0.000230707	-0.000961994
5	6	-0.000143977	-0.001950148	-0.000283851
6	6	-0.002142909	-0.003276385	0.003108740
7	1	-0.001899690	-0.006141647	0.000142310
8	1	0.002454953	-0.002460331	-0.005893060
9	1	0.000170655	0.006051109	0.002622750
10	1	-0.003375680	-0.000704148	0.005422263
11	1	0.005488216	0.002962653	0.003100635
12	1	-0.003831726	0.004227291	-0.002003488
13	1	0.002851771	-0.002553381	-0.004839169
14	8	-0.000425119	0.000191906	-0.000076273
15	8	-0.003335588	0.006849184	0.002159416
16	6	0.000625541	-0.000500241	0.000193907
17	6	-0.000136443	0.000168689	0.000122387
18	6	0.000631483	-0.004154873	-0.002116252
19	6	-0.000252004	0.000234911	-0.000269010
20	6	-0.000158575	-0.000034449	-0.000275649
21	6	-0.000261559	0.000246279	-0.000593405
22	6	-0.000065124	-0.000453140	0.000286377
23	6	-0.000117372	0.000606909	-0.001232364
24	1	-0.000209804	-0.000131035	0.000000032
25	1	0.000326280	-0.000292724	0.000702972
26	1	0.000096399	-0.000098971	-0.000070894
27	1	0.000057137	-0.000297217	0.000337367
28	1	0.000181838	-0.000014426	0.000520461
29	1	-0.000093676	-0.000165838	0.000321156
30	8	0.000453302	-0.000417711	-0.000155456
31	8	-0.001482866	-0.002149313	0.001227417
32	6	-0.000722257	0.000733672	0.000515004
33	6	-0.000473941	-0.000988223	0.000224132
34	6	0.000598883	0.001757755	-0.000200771
35	6	-0.000227728	-0.000418425	-0.000328663
36	6	0.000157503	0.000506352	0.000077389
37	6	0.000500528	0.000059810	-0.000571303
38	6	0.000000301	0.000012576	0.000575481
39	6	0.000492983	0.000377199	-0.001141061

40	1	0.000352562	0.000376778	-0.000111048
41	1	-0.000600037	-0.000249985	0.000520487
42	1	-0.000042416	0.000151974	-0.000017355
43	1	-0.000283809	-0.000327767	0.000252217
44	1	-0.000052983	-0.000099890	0.000325554
45	1	-0.000153189	-0.000004848	0.000357844
46	8	0.000743630	0.000923928	-0.000155354
47	8	0.000250589	0.000305879	-0.000563962
48	6	-0.000547954	-0.000383461	-0.000085680
49	6	0.000334914	0.000745462	0.000105438
50	6	-0.000794379	-0.000480505	0.000594664
51	6	0.000398545	0.000167546	-0.000827424
52	6	-0.000099366	-0.000096593	-0.000252040
53	6	0.000197815	0.000342546	-0.000453719
54	6	0.000045270	-0.000345785	0.000363834
55	6	0.000688698	0.000419281	-0.001131679
56	1	-0.000019487	-0.000368368	-0.000112936
57	1	-0.000241406	0.000010818	0.000795258
58	1	-0.000123888	-0.000039367	-0.000075452
59	1	-0.000207122	-0.000233104	0.000290229
60	1	-0.000110182	-0.000114300	0.000370654
61	1	-0.000367049	0.000032227	0.000360357
62	8	-0.001409286	-0.000800540	-0.001047567
63	8	-0.000047109	0.000039214	-0.000659514
64	6	0.000954984	0.000751035	0.000686702
65	6	-0.000336103	-0.000716332	-0.000597279
66	6	0.000325440	-0.000247260	0.000889905
67	6	-0.000125892	0.000454682	-0.000837105
68	6	0.000247143	0.000229252	0.000007709
69	6	-0.000054505	-0.000100510	-0.000556297
70	6	-0.000173210	0.000075994	0.000543998
71	6	-0.000219110	0.000526604	-0.001323731
72	1	-0.000068404	0.000474183	0.000225470
73	1	-0.000045675	-0.000571890	0.000628125
74	1	0.000103254	0.000061167	0.000044640
75	1	-0.000020838	-0.000152905	0.000346172
76	1	0.000264042	-0.000303014	0.000397536
77	1	0.000060538	-0.000073799	0.000443317
78	8	0.000486259	0.000094631	0.000018835
79	8	0.000211965	0.002673384	-0.001361921
80	6	-0.000784551	-0.000186507	-0.000483402
81	6	0.000219688	0.001110010	-0.000373437
82	6	-0.000670944	-0.001925393	0.000306371
83	6	-0.000002224	0.000566074	0.000297728
84	6	-0.000103479	-0.000460931	-0.000110711
85	6	0.000319380	-0.000347448	0.000688148
86	6	-0.000008248	0.000017068	-0.000551897
87	6	0.000074016	-0.000581309	0.001148077
88	1	0.000042844	-0.000504060	0.000177700
89	1	-0.000288743	0.000537700	-0.000606817
90	1	-0.000112942	-0.000102149	-0.000002005
91	1	-0.000100451	0.000106083	-0.000380582
92	1	-0.000017830	0.000416422	-0.000287379
93	1	0.000040923	0.000101735	-0.000307729
94	8	0.000007914	0.000055880	-0.000281141
95	8	0.000590194	-0.001048849	-0.002915890
96	6	0.000396204	0.000204318	0.000078647
97	6	0.000186615	-0.000539030	-0.000942043

98	6	0.000286776	0.001140713	0.001621410
99	6	-0.000070916	-0.000577625	0.000018438
100	6	-0.000023777	0.000300002	0.000200007
101	6	-0.000226003	-0.000192789	0.000549470
102	6	0.000009909	0.000392145	-0.000382612
103	6	-0.000409998	-0.000387666	0.001179632
104	1	-0.000200819	0.000291887	0.000482497
105	1	0.000423037	-0.000005925	-0.000687580
106	1	0.000086186	0.000077415	0.000107722
107	1	0.000029160	0.000159673	-0.000335842
108	1	0.000229778	0.000176534	-0.000282508
109	1	0.000164915	-0.000117052	-0.000453916
110	8	0.000991447	-0.001904465	-0.000562426
111	8	-0.000120639	-0.000316553	0.000394514
112	6	-0.000549265	0.001331782	0.000450937
113	6	-0.000021567	-0.000794395	-0.000459374
114	6	-0.000338391	0.000692110	-0.000643813
115	6	0.000161783	-0.000405961	0.000985243
116	6	-0.000201638	0.000267544	0.000293307
117	6	0.000078156	-0.000506393	0.000262079
118	6	0.000277115	0.000213308	-0.000365902
119	6	0.000158116	-0.000667941	0.001196743
120	1	0.000164833	0.000291937	0.000286624
121	1	-0.000109020	0.000026422	-0.000891028
122	1	-0.000056153	0.000089815	0.000028054
123	1	-0.000279208	0.000192638	-0.000376837
124	1	0.000020273	0.000293317	-0.000315749
125	1	0.000017364	0.000143704	-0.000380110
126	8	-0.000868310	0.001400371	0.000725791
127	8	0.000409708	-0.000402954	0.000348944
128	6	0.000379741	-0.001168018	-0.000541372
129	6	0.000129560	0.000652511	0.000417120
130	6	0.000107148	0.000133411	-0.000464963
131	6	-0.000355445	-0.000267120	0.000664834
132	6	-0.000002755	-0.000353950	-0.000105870
133	6	0.000003787	0.000077079	0.000642603
134	6	-0.000079160	0.000126135	-0.000511167
135	6	-0.000542176	-0.000324954	0.001189045
136	1	-0.000164082	-0.000242981	-0.000153435
137	1	0.000414690	0.000516485	-0.000522664
138	1	0.000004961	-0.000099601	-0.000051604
139	1	0.000125036	0.000000638	-0.000395951
140	1	0.000084591	0.000143665	-0.000313400
141	1	0.000392735	0.000152605	-0.000352501
142	8	-0.000021063	0.000175231	0.001335939
143	1	0.000053519	0.000027820	-0.000476193
144	1	-0.000264257	0.000001481	-0.000495047
145	8	0.000570961	0.000272060	-0.000169869
146	1	0.000231734	0.000914867	0.000283683
147	1	0.000838857	0.000074088	-0.000536814
148	8	0.003301187	-0.003868635	-0.008553710
149	1	-0.001675029	0.004400431	0.003962373
150	1	-0.003054667	-0.000524256	0.004612137
151	8	0.000026001	-0.000670042	0.000158202
152	1	-0.000068990	-0.000780267	-0.000347861
153	1	0.000666129	-0.000446617	0.000634754
154	8	0.004627126	0.002896380	0.008502273
155	1	-0.002348123	-0.003628056	-0.004584574

156	1	-0.003588319	-0.000039607	-0.005485189
157	1	-0.000054381	0.000244358	0.000599740
158	1	-0.000724488	-0.000381538	0.000197771
159	1	-0.000158678	0.000334772	-0.000206075
160	1	-0.000221169	-0.000514644	0.000460622
161	1	0.000029716	-0.000073628	0.000064968
162	8	-0.000052058	-0.000551768	-0.000043868
163	8	-0.002686785	-0.000627867	-0.000358836
164	6	0.000185034	-0.000057927	0.000165837
165	6	-0.001023044	-0.000529613	-0.000225768
166	6	0.002570559	0.000630434	0.000784980
167	6	-0.000708545	-0.000067354	0.000137491
168	6	0.000455594	0.000002120	0.000158623
169	6	0.000031359	0.000634492	0.000008513
170	6	-0.000071202	-0.000259207	-0.000539312
171	6	0.000589443	0.000917461	0.000715650
172	1	0.000521148	0.000233230	-0.000005302
173	1	-0.000371324	-0.000677576	0.000033887
174	1	0.000020884	0.000117567	-0.000114262
175	1	-0.000071792	-0.000324757	-0.000113583
176	1	-0.000409445	-0.000281512	-0.000152590
177	1	-0.000155373	-0.000256489	-0.000324049
178	8	-0.000496217	-0.000284596	-0.000686842
179	8	0.001421347	0.000331309	-0.000096088
180	6	0.000371273	-0.000073355	0.000239780
181	6	0.000100488	0.000020487	-0.000369426
182	6	-0.000426340	-0.000031020	-0.000188273
183	6	0.000122663	0.000502119	0.000381366
184	6	0.000060117	0.000132348	0.000226018
185	6	-0.000072892	0.000459377	0.000234689
186	6	-0.000345807	-0.000441713	-0.000071861
187	6	0.000065651	0.001291679	0.000589621
188	1	-0.000136851	-0.000052293	0.000235352
189	1	0.000101266	-0.000574749	-0.000717021
190	1	0.000039440	-0.000026022	0.000111825
191	1	-0.000061311	-0.000441919	-0.000079418
192	1	-0.000127140	-0.000308716	-0.000223170
193	1	0.000181344	-0.000390380	-0.000280171
194	8	-0.000284046	-0.001130430	0.000857133
195	8	0.000448012	0.000313267	-0.000445710
196	6	0.000438800	0.000358140	-0.000487507
197	6	-0.000249954	-0.000127469	0.000138380
198	6	-0.000161986	-0.000420291	-0.000017174
199	6	0.000207473	0.000563716	0.000470587
200	6	0.000400594	-0.000023175	-0.000018790
201	6	0.000018076	0.000208168	0.000464861
202	6	0.000022302	-0.000560939	-0.000063345
203	6	0.000317376	0.001079872	0.000756021
204	1	0.000109565	0.000123714	0.000269149
205	1	-0.000553879	-0.000394584	-0.000490207
206	1	0.000090979	-0.000036353	0.000034591
207	1	-0.000176553	-0.000213432	-0.000422928
208	1	0.000002973	-0.000360484	-0.000198259
209	1	-0.000151081	-0.000304552	-0.000095456
210	8	-0.000470369	0.000337783	-0.000007774
211	8	-0.001317936	-0.000669749	0.000857235
212	6	0.000754908	-0.000523465	-0.000480038
213	6	-0.000335885	-0.000181868	0.000061887

214	6	0.000624928	0.000261326	-0.000414847
215	6	-0.000270986	0.000317389	0.000435272
216	6	0.000034358	0.000259814	-0.000382443
217	6	-0.000361969	0.000864192	0.000334908
218	6	0.000241091	-0.000349639	-0.000448452
219	6	-0.000036724	0.001296365	0.000431680
220	1	-0.000008205	0.000100084	0.000038603
221	1	0.000300877	-0.000871973	-0.000111307
222	1	-0.000023656	0.000026907	0.000004127
223	1	0.000041205	-0.000535347	0.000064941
224	1	-0.000138175	-0.000323664	-0.000144338
225	1	0.000041265	-0.000379615	-0.000245651
226	1	0.000748536	0.000177987	-0.000742466
227	1	0.000003305	-0.000079420	0.000193561
228	1	0.000014998	-0.000454196	0.000692827
229	1	-0.000071721	-0.000021638	0.000014963
230	1	0.000799824	0.000275083	0.000699392
231	1	0.000027233	-0.000119786	-0.000333625
232	1	0.000250398	-0.000156813	0.000733677
233	1	-0.000196732	-0.000092649	0.000207473
234	1	-0.000253553	0.000541726	-0.000493510
235	1	0.000019304	-0.000054114	0.000069309
236	8	-0.000057736	0.000198194	0.000154800
237	8	-0.001660288	0.001954699	0.000148311
238	6	0.000509287	0.000209781	-0.000300808
239	6	-0.000818785	0.000813446	0.000009647
240	6	0.001772434	-0.001722462	-0.000543281
241	6	-0.000446593	0.000371345	-0.000209805
242	6	0.000440425	-0.000289810	-0.000099024
243	6	-0.000353268	-0.000665750	-0.000020168
244	6	0.000090751	0.000233708	0.000448560
245	6	0.000088004	-0.001154734	-0.000598755
246	1	0.000251017	-0.000331915	0.000102468
247	1	0.000030421	0.000850073	-0.000144655
248	1	-0.000091610	-0.000096835	0.000087647
249	1	-0.000175772	0.000474514	0.000084783
250	1	-0.000008725	0.000327790	0.000326171
251	1	0.000109276	0.000339052	0.000107240
252	8	0.000310883	0.000148831	-0.000230593
253	8	0.002424369	0.000097582	0.001017018
254	6	-0.000599886	0.000183531	0.000571356
255	6	0.001183023	0.000299768	0.000528730
256	6	-0.002646072	-0.000449174	-0.000798868
257	6	0.000620416	-0.000203634	0.000462389
258	6	-0.000576145	0.000121768	-0.000028282
259	6	0.000141239	-0.000490754	-0.000568677
260	6	0.000190962	0.000580347	-0.000178160
261	6	-0.000655073	-0.001062937	-0.000306756
262	1	-0.000385415	-0.000094062	-0.000223446
263	1	0.000250466	0.000414799	0.000589231
264	1	0.000006828	0.000031377	-0.000180092
265	1	0.000207635	0.000379908	0.000011149
266	1	0.000058711	0.000269745	0.000135607
267	1	0.000394454	0.000218612	0.000269554
268	8	0.000162925	0.000134789	0.001434272
269	8	-0.000416068	0.000088167	-0.000501708
270	6	-0.000251173	0.000206957	-0.000858907
271	6	0.000140126	-0.000076898	0.000325008

272	6	0.000387573	0.000480498	0.000007327
273	6	-0.000566655	-0.000587056	-0.000100332
274	6	-0.000244080	-0.000020794	-0.000137284
275	6	-0.000163851	-0.000475421	0.000107843
276	6	0.000172184	0.000215148	0.000425734
277	6	-0.000610063	-0.001095390	-0.000363740
278	1	-0.000055332	-0.000073482	0.000055424
279	1	0.000643942	0.000511788	0.000139795
280	1	-0.000060374	0.000018241	-0.000020385
281	1	0.000211830	0.000258182	0.000225522
282	1	0.000304348	0.000384439	-0.000057580
283	1	0.000057621	0.000376871	0.000108318
284	8	0.000457433	0.000826706	-0.000636792
285	8	0.000591522	-0.000464625	0.000525629
286	6	-0.000207359	-0.000327419	0.000411837
287	6	-0.000039291	0.000148292	-0.000102713
288	6	-0.000092877	0.000397111	0.000016022
289	6	0.000059395	-0.000690612	-0.000165349
290	6	0.000078565	-0.000169834	-0.000166011
291	6	0.000193179	-0.000384304	-0.000335423
292	6	-0.000039347	0.000538818	-0.000074623
293	6	0.000043762	-0.001115897	-0.000659704
294	1	0.000075159	-0.000069749	0.000042246
295	1	-0.000252481	0.000660464	0.000445691
296	1	0.000047512	-0.000024899	0.000028395
297	1	0.000109583	0.000262276	0.000192066
298	1	-0.000057830	0.000390019	0.000089375
299	1	-0.000125687	0.000282896	0.000398432
300	1	0.000485918	0.000471322	0.000435919
301	1	-0.002249097	0.001796026	0.001111281
302	1	0.000094497	-0.000575329	-0.000453696
303	1	-0.002792228	-0.000164651	-0.001381511
304	1	-0.001271539	0.000358187	0.002548269
305	1	0.000189611	-0.000831332	-0.000941165
306	1	-0.001372041	-0.000478159	0.000659256
307	1	0.000688843	0.000749343	0.001039567
308	1	-0.000943325	0.000919737	-0.000746733
309	1	0.001566001	-0.003577705	0.000906344
310	8	-0.000665501	-0.000684845	-0.000287626
311	8	0.002235754	-0.000846354	-0.001939398
312	6	0.000418655	0.000282682	0.000330800
313	6	0.000833621	-0.000545763	-0.001016345
314	6	-0.001086444	-0.000047640	0.001505889
315	6	-0.000277917	0.000017927	-0.000406468
316	6	-0.000015665	0.000029071	0.000257882
317	6	-0.000507983	0.000123196	-0.000202412
318	6	0.000616255	0.000110902	0.000254864
319	6	-0.001318922	0.000199519	0.000002621
320	1	-0.000612447	0.000277622	0.000551199
321	1	0.000549676	-0.000418586	-0.000222852
322	1	-0.000066742	-0.000051496	0.000122723
323	1	0.000326563	-0.000165465	0.000056779
324	1	0.000458033	-0.000083420	-0.000336035
325	1	0.000405709	0.000015517	0.000082881
326	8	-0.000301927	0.000565346	0.000399512
327	8	0.002130446	-0.000620675	0.002357617
328	6	0.000588025	-0.000027642	-0.000388007
329	6	0.000913781	-0.000236461	0.001138021

330	6	-0.000862006	0.000794367	-0.001791626
331	6	-0.000151721	-0.000021811	0.000532260
332	6	0.000069202	0.000161419	-0.000408217
333	6	-0.000722948	0.000013754	0.000207253
334	6	0.000499982	-0.000288679	-0.000175174
335	6	-0.001218485	0.000453294	-0.000147430
336	1	-0.000621705	0.000070176	-0.000561763
337	1	0.000731705	0.000012428	0.000331770
338	1	-0.000000990	0.000096147	-0.000117403
339	1	0.000317901	-0.000199791	-0.000056220
340	1	0.000395188	-0.000013511	-0.000001372
341	1	0.000368998	-0.000150106	0.000287733
342	8	-0.000034502	0.001082351	0.001854237
343	8	0.000905594	0.000648791	-0.000852888
344	6	-0.000094858	-0.000451838	-0.001458825
345	6	0.000252355	-0.000045789	0.000542187
346	6	-0.000006704	-0.000819090	-0.000046634
347	6	-0.000731764	0.000557815	-0.000227434
348	6	-0.000056072	-0.000007611	-0.000379994
349	6	-0.000421612	0.000241469	0.000383464
350	6	0.000536534	0.000024444	0.000265706
351	6	-0.001144417	0.000661027	-0.000335760
352	1	-0.000134127	0.000161781	-0.000155213
353	1	0.000588079	-0.000485089	0.000358465
354	1	0.000005860	-0.000016348	0.000177321
355	1	0.000325759	-0.000097814	0.000019870
356	1	0.000357232	-0.000164523	0.000185602
357	1	0.000312838	-0.000431198	0.000053034
358	8	-0.000619659	-0.000995557	-0.001924129
359	8	0.000148219	-0.001059151	0.000765399
360	6	0.000160814	0.000554061	0.001356091
361	6	0.000083577	-0.000176938	-0.000585721
362	6	0.000632851	0.000964624	-0.000002975
363	6	-0.001063925	-0.000334478	0.000154922
364	6	-0.000106577	0.000216579	0.000385502
365	6	-0.000556061	-0.000109989	-0.000394738
366	6	0.000395233	-0.000336694	-0.000278512
367	6	-0.001398878	0.000085598	0.000231010
368	1	-0.000185790	-0.000148078	0.000163967
369	1	0.000793556	0.000062459	-0.000230506
370	1	0.000025486	0.000057734	-0.000196369
371	1	0.000525499	0.000225509	-0.000024510
372	1	0.000351306	-0.000088016	0.000030792
373	1	0.000466359	-0.000087608	-0.000133749
374	1	-0.000284511	0.000069920	-0.000608413
375	1	0.000512559	-0.001094458	0.000285075
376	1	0.000074130	0.000096243	0.000120029
377	1	0.002823603	0.001032260	0.000144984
378	1	-0.000606675	-0.000480354	0.000183715
379	1	-0.000590174	0.001138157	-0.000022726
380	1	0.000513306	-0.000051506	-0.000265896
381	1	-0.000052547	0.001246748	-0.000103055
382	1	0.000691957	-0.000299196	-0.000802036
383	1	0.001727732	0.001271583	-0.001779993
384	8	0.000266772	-0.000411327	0.000742796
385	8	-0.000475930	0.000607172	-0.000687873
386	6	0.000097842	0.000647223	-0.000050211
387	6	0.000009418	0.000176354	-0.000186007

388	6	0.000004541	0.000108308	0.000360963
389	6	0.000708040	-0.000265190	0.000069168
390	6	-0.000023243	-0.000164064	-0.000005888
391	6	0.000570165	-0.000451836	0.000213020
392	6	-0.000602546	0.000028346	0.000141349
393	6	0.001223482	-0.000634993	0.000193536
394	1	-0.000085189	-0.000246405	0.000120768
395	1	-0.000477219	0.000781434	-0.000084299
396	1	0.000012498	0.000018769	0.000120064
397	1	-0.000375221	0.000066488	-0.000067650
398	1	-0.000344076	0.000214897	0.000051614
399	1	-0.000312447	0.000357848	-0.000257493
400	8	0.000147496	-0.000616272	0.000273180
401	8	-0.002700854	-0.000905066	0.001016440
402	6	-0.000274598	0.000322277	-0.000488135
403	6	-0.001200871	-0.000519147	0.000524525
404	6	0.001394631	0.000304715	-0.001208978
405	6	-0.000100792	-0.000506748	0.000376639
406	6	0.000175091	0.000163842	-0.000320383
407	6	0.000472565	-0.000192471	0.000365992
408	6	-0.000404132	0.000435587	0.000011944
409	6	0.001237466	-0.000370387	-0.000041605
410	1	0.000673781	0.000299931	-0.000176473
411	1	-0.000723916	-0.000119033	-0.000089630
412	1	0.000091025	-0.000028383	-0.000145676
413	1	-0.000326379	0.000227178	-0.000004732
414	1	-0.000380422	0.000075176	-0.000118839
415	1	-0.000473913	-0.000052605	0.000178047
416	8	0.000725430	0.000897694	-0.000199683
417	8	-0.000693450	-0.000069095	0.001039405
418	6	-0.000649057	-0.000619480	0.000149847
419	6	-0.000137977	0.000237667	-0.000025136
420	6	-0.000137766	-0.000167040	-0.000905699
421	6	0.000489431	-0.000268602	0.000482049
422	6	0.000077498	-0.000054510	0.000120806
423	6	0.000707957	-0.000340149	-0.000068846
424	6	-0.000241903	0.000575184	-0.000201262
425	6	0.001126962	-0.000535078	0.000626590
426	1	0.000055758	0.000017558	0.000184946
427	1	-0.000615472	0.000205210	-0.000519815
428	1	0.000038987	0.000099246	-0.000192054
429	1	-0.000351410	0.000269781	-0.000194135
430	1	-0.000384086	-0.000005677	-0.000320362
431	1	-0.000330388	0.000110227	-0.000030330
432	8	-0.000612077	-0.002120109	0.000569823
433	8	-0.000136232	-0.000257445	0.000067539
434	6	0.000999727	0.001535709	-0.000736328
435	6	-0.000293429	-0.000764214	0.000436052
436	6	-0.000464180	0.000956173	0.000426796
437	6	0.000856223	-0.000742511	-0.000388341
438	6	0.000350218	0.000601606	-0.000110904
439	6	-0.000046367	-0.000598926	0.000320453
440	6	-0.000474776	0.000099520	-0.000342095
441	6	0.001226207	-0.000698808	0.000028099
442	1	0.000025013	0.000045953	-0.000275480
443	1	-0.000768707	0.000271415	0.000385157
444	1	-0.000023801	-0.000054930	0.000068107
445	1	-0.000332812	0.000374921	0.000155097

446	1	-0.000326909	0.000187998	-0.000124951
447	1	-0.000397400	0.000094639	0.000028740
448	1	-0.000080232	0.000516990	-0.000894591
449	1	-0.000305687	-0.000758655	0.000175379
450	1	0.000279772	-0.000311917	0.000395715
451	1	-0.000337737	0.000117112	0.000430614
452	1	0.001029974	-0.000376689	-0.000828934
453	1	0.000476492	-0.001919893	0.001946357
454	1	0.000565493	-0.000710505	-0.000526455
455	1	-0.000287161	0.000217725	0.000167928
456	1	0.000082907	0.000784616	-0.000177901
457	1	-0.000429488	0.000347716	-0.000352459
458	6	0.000493238	0.001353582	-0.001537899
459	6	0.000007793	0.000410613	-0.000590661
460	6	-0.000086701	-0.001158567	-0.000132767
461	6	-0.000126059	0.000104434	-0.000005730
462	6	-0.000508067	0.000463594	0.000438482
463	6	-0.000327500	-0.000224677	-0.000107032
464	1	-0.000189412	0.006289072	0.001316288
465	1	-0.002327447	0.001480255	0.005719101
466	1	0.000676514	-0.005961210	-0.002016513
467	1	0.002706904	-0.001117559	-0.005692816
468	17	0.001735028	-0.000617983	-0.003632484
469	1	0.000117543	0.000270438	-0.000049003
470	1	0.000347283	0.000387610	-0.000747071
471	1	0.000038121	-0.000128829	0.000063732
472	8	0.002131835	0.001103383	-0.001129970
473	1	-0.000925426	-0.000155166	0.000797101
474	1	-0.000901654	-0.000756823	0.000164686
475	8	-0.001565801	0.000757932	0.001722296
476	1	0.000486805	-0.000650311	-0.000619534
477	1	0.000746720	-0.000010693	-0.000901327
478	8	0.001178887	-0.002040140	0.000985475
479	1	-0.000403170	0.000596273	-0.000835921
480	1	-0.000261853	0.001137691	-0.000013658
481	6	-0.000432702	-0.003227649	-0.000715456
482	8	-0.001102130	0.007243490	0.002527755
483	1	-0.001850726	-0.004611706	0.003841440

Sum of electronic and thermal Free Energies= -1284.445411
0 imaginary frequency

[2+3e]@C$_R$

Center Number	Atomic Number	Integrated Forces (Hartrees/Bohr) X	Y	Z
1	6	0.001036719	-0.000971256	0.001766954
2	6	0.000519805	0.001826493	0.000382874
3	7	-0.000314289	0.003431444	-0.001802857
4	6	0.000106529	0.002000321	0.000548891
5	6	0.000826715	-0.001748660	-0.001415829
6	6	0.001119901	-0.005615031	0.002816285
7	1	-0.000850040	-0.006272720	-0.001841473
8	1	-0.002549504	0.000337955	-0.006323562
9	1	0.003368554	0.005087558	0.003962097
10	1	0.001478086	-0.003640589	0.005272709

11	1	-0.005833514	0.001966792	0.001859239
12	1	0.000608430	0.000571981	-0.006681638
13	1	0.004147752	0.003652197	0.002083672
14	8	-0.000951438	0.000229171	-0.000356458
15	8	-0.005306756	0.000208252	0.006203281
16	6	0.000941099	-0.000383418	-0.000227367
17	6	-0.000269915	0.000145036	-0.000025119
18	6	0.001775098	0.000557019	-0.004110539
19	6	-0.000430650	0.000717744	-0.000085437
20	6	-0.000153736	0.000283326	0.000017303
21	6	-0.000355997	0.000737749	-0.000050930
22	6	0.000133046	-0.000386560	-0.000431543
23	6	-0.000364784	0.001325301	0.000221198
24	1	-0.000034564	-0.000067200	-0.000117524
25	1	0.000453199	-0.000793620	0.000112111
26	1	0.000133320	0.000021973	-0.000091270
27	1	-0.000017715	-0.000341379	-0.000104262
28	1	0.000160453	-0.000379644	-0.000163010
29	1	0.000169088	-0.000508740	0.000187307
30	8	0.000662604	0.000124300	-0.000356943
31	8	-0.000705260	-0.001853834	-0.002362119
32	6	-0.000997613	-0.000466347	0.000586691
33	6	-0.000031610	-0.000450878	-0.001054510
34	6	-0.000040988	0.000722685	0.001933193
35	6	-0.000105575	0.000123117	-0.000565690
36	6	-0.000018412	-0.000033756	0.000488478
37	6	0.000538475	0.000643362	-0.000003317
38	6	0.000033738	-0.000526823	0.000085172
39	6	0.000312948	0.001145230	0.000263424
40	1	0.000148347	0.000164863	0.000434535
41	1	-0.000544628	-0.000514592	-0.000203196
42	1	-0.000081973	0.000030148	0.000161111
43	1	-0.000154547	-0.000329603	0.000012536
44	1	-0.000184634	-0.000340937	-0.000339606
45	1	-0.000004581	-0.000316950	-0.000038587
46	8	0.000497448	0.000885669	0.000593403
47	8	0.000067235	0.000523845	-0.000155968
48	6	-0.000151668	-0.000597451	-0.000055478
49	6	0.000003838	0.000222352	0.000192312
50	6	-0.000595418	-0.000572859	-0.000003936
51	6	0.000228253	0.000697894	-0.000096535
52	6	0.000117802	0.000208284	0.000009729
53	6	-0.000036121	0.000756725	-0.000035703
54	6	0.000196166	-0.000438902	-0.000056305
55	6	0.000465635	0.001216674	0.000179820
56	1	0.000165000	-0.000164976	-0.000189200
57	1	-0.000116689	-0.000842671	0.000194787
58	1	-0.000100186	0.000032577	-0.000068772
59	1	-0.000357888	-0.000372795	0.000013374
60	1	-0.000121606	-0.000321803	-0.000171216
61	1	-0.000051591	-0.000379604	-0.000026562
62	8	-0.001428421	0.000762433	-0.001546130
63	8	-0.000237189	0.000421166	0.000075538
64	6	0.001013001	-0.000138311	0.001192298
65	6	-0.000534922	0.000266234	-0.000384131
66	6	0.000772606	-0.000817303	-0.000247858
67	6	-0.000525505	0.001038580	0.000240277
68	6	0.000410475	0.000016113	0.000377128

69	6	-0.000229621	0.000342615	-0.000325396
70	6	-0.000133156	-0.000556141	0.000318778
71	6	-0.000359857	0.001375577	0.000073545
72	1	-0.000030206	-0.000090784	0.000187186
73	1	0.000183046	-0.000720627	-0.000623921
74	1	0.000049915	-0.000043402	0.000084069
75	1	0.000062109	-0.000432576	0.000052479
76	1	0.000014490	-0.000341236	-0.000058159
77	1	0.000353496	-0.000419400	-0.000111326
78	8	0.000259033	0.000156035	0.000084369
79	8	-0.000703754	0.001777538	0.001979555
80	6	-0.000526537	0.000294158	-0.000409096
81	6	-0.000238956	0.000552180	0.000876793
82	6	0.000029564	-0.000585417	-0.001573034
83	6	-0.000148709	-0.000295087	0.000384719
84	6	-0.000003533	0.000016698	-0.000412141
85	6	0.000298821	-0.000504067	-0.000088444
86	6	0.000126251	0.000525605	-0.000085989
87	6	0.000200362	-0.001213913	-0.000301379
88	1	0.000276279	-0.000253842	-0.000371726
89	1	-0.000372950	0.000598032	0.000318498
90	1	-0.000096382	-0.000021515	-0.000124754
91	1	-0.000177281	0.000392597	0.000318598
92	1	0.000018924	0.000339692	0.000056396
93	1	-0.000109325	0.000369629	-0.000013433
94	8	-0.000341355	0.000161494	-0.000183675
95	8	0.000302031	0.001931760	-0.000670797
96	6	0.000690737	-0.000024240	0.000472997
97	6	0.000018246	0.000832981	-0.000364858
98	6	0.000076940	-0.001074999	0.000714176
99	6	-0.000054280	-0.000048273	-0.000356103
100	6	-0.000124520	-0.000195524	0.000282632
101	6	-0.000376176	-0.000500596	-0.000214855
102	6	0.000070640	0.000379963	0.000372789
103	6	-0.000434663	-0.001178621	-0.000326164
104	1	-0.000159156	-0.000342015	0.000213989
105	1	0.000559435	0.000638456	0.000118480
106	1	0.000149310	-0.000106246	0.000109186
107	1	0.000215405	0.000407757	-0.000125642
108	1	0.000012471	0.000338357	0.000129156
109	1	0.000180296	0.000332232	0.000179561
110	8	0.001302399	0.000215718	-0.001607472
111	8	-0.000123078	-0.000474286	-0.000119048
112	6	-0.000977238	-0.000277185	0.001178559
113	6	0.000171063	0.000042512	-0.000889846
114	6	-0.000402867	0.000684003	0.000384278
115	6	0.000256711	-0.000846810	-0.000114177
116	6	-0.000198731	-0.000189473	0.000173504
117	6	0.000102552	-0.000327417	-0.000426849
118	6	0.000151589	0.000400587	0.000269146
119	6	0.000387281	-0.001244974	-0.000337057
120	1	0.000141537	-0.000059565	0.000393700
121	1	-0.000194176	0.000862917	-0.000206064
122	1	-0.000103374	-0.000029717	0.000062079
123	1	-0.000093585	0.000345871	0.000203994
124	1	-0.000027416	0.000387028	0.000058489
125	1	-0.000274486	0.000395395	0.000013798
126	8	-0.001341331	-0.000574875	0.001076383

127	8	-0.000081057	-0.000647236	-0.000129156
128	6	0.000766867	0.000384899	-0.000897264
129	6	-0.000256561	-0.000252895	0.000679004
130	6	0.000589922	0.000897654	0.000007988
131	6	-0.000348500	-0.000933208	-0.000269176
132	6	0.000155288	0.000016522	-0.000277808
133	6	-0.000098621	-0.000688054	0.000234198
134	6	-0.000157277	0.000539399	-0.000020779
135	6	-0.000472432	-0.001268315	-0.000275013
136	1	-0.000135041	0.000080555	-0.000383833
137	1	0.000192442	0.000683225	0.000468021
138	1	0.000080059	0.000018289	-0.000080058
139	1	0.000119096	0.000393727	-0.000009853
140	1	0.000058222	0.000348188	0.000099838
141	1	0.000342334	0.000364594	0.000144587
142	8	-0.001441189	0.001909034	-0.000875814
143	1	0.000528146	-0.000883400	0.000163399
144	1	0.000632568	-0.000584518	0.000814674
145	8	0.000170431	0.000319427	0.000270271
146	1	0.000866136	0.000585989	0.000376321
147	1	0.000075998	-0.000235086	0.001070103
148	8	0.004553637	0.007376867	0.001274408
149	1	-0.003815276	-0.004415449	0.001957628
150	1	-0.002602112	-0.002235285	-0.003348863
151	8	0.000322933	-0.000178763	-0.000523273
152	1	-0.000026666	0.000176791	-0.000985272
153	1	0.000766113	-0.000665357	-0.000186187
154	8	0.001759080	-0.007314708	0.001070957
155	1	-0.002007695	0.004138801	0.001891288
156	1	-0.001633799	0.005133610	-0.003180171
157	1	-0.000056933	-0.000639813	0.000478648
158	1	-0.000478730	0.000220499	0.000019156
159	1	-0.000009195	0.000135781	0.000492900
160	1	-0.000047084	-0.000541501	-0.000282652
161	1	-0.000064380	-0.000135937	0.000243995
162	8	-0.000081149	0.000026766	-0.000397731
163	8	-0.002493925	0.000172990	-0.001722783
164	6	0.000403904	-0.000300177	-0.000234619
165	6	-0.000983908	0.000029307	-0.000870783
166	6	0.002406842	-0.000459656	0.001613329
167	6	-0.000687073	-0.000255880	-0.000286839
168	6	0.000626856	-0.000165955	0.000064267
169	6	-0.000286167	0.000086121	0.000802645
170	6	0.000063748	0.000445688	-0.000396692
171	6	0.000227246	-0.000443286	0.001182077
172	1	0.000345909	0.000090293	0.000438496
173	1	-0.000184795	-0.000269328	-0.000849002
174	1	-0.000008441	0.000131883	0.000074802
175	1	0.000047155	0.000053736	-0.000336476
176	1	-0.000301038	0.000045132	-0.000388185
177	1	-0.000025545	0.000239444	-0.000356293
178	8	-0.000563678	0.000735135	-0.000606906
179	8	0.000748702	-0.000587955	-0.000508301
180	6	0.000420245	-0.000345288	-0.000175990
181	6	0.000119006	0.000214873	-0.000039110
182	6	-0.000278271	0.000339049	-0.000117141
183	6	-0.000075990	-0.000246221	0.000620624
184	6	-0.000078817	-0.000308440	0.000024993

185	6	-0.000142792	-0.000115212	0.000617275
186	6	-0.000194362	0.000008001	-0.000521455
187	6	-0.000333995	-0.000320277	0.001315652
188	1	-0.000042301	-0.000226761	-0.000018722
189	1	0.000328747	0.000624626	-0.000674900
190	1	0.000041514	-0.000086854	-0.000009993
191	1	-0.000018727	0.000133144	-0.000358760
192	1	0.000329231	0.000202955	-0.000357274
193	1	0.000082207	-0.000014778	-0.000411515
194	8	-0.000284744	-0.001091549	-0.001237498
195	8	0.000396539	0.000532047	0.000074420
196	6	0.000517629	0.000666147	0.000501541
197	6	-0.000283414	-0.000155644	-0.000331955
198	6	-0.000096120	-0.000006393	-0.000346102
199	6	0.000103161	-0.000320299	0.000735202
200	6	0.000230599	0.000212483	0.000239781
201	6	-0.000117067	-0.000397572	0.000159004
202	6	0.000210142	-0.000053070	-0.000560741
203	6	-0.000024534	-0.000490692	0.001268997
204	1	0.000108404	-0.000071709	0.000159297
205	1	-0.000417666	0.000400581	-0.000617026
206	1	0.000082860	-0.000015077	-0.000004400
207	1	-0.000050334	0.000016829	-0.000365248
208	1	-0.000122984	0.000366095	-0.000326125
209	1	0.000087571	0.000106253	-0.000374960
210	8	-0.000076225	0.000452948	-0.000437436
211	8	-0.000644103	-0.000896678	-0.000335170
212	6	0.000234722	-0.000153653	-0.000182700
213	6	0.000172271	-0.000082203	-0.000163017
214	6	0.000148304	0.000467467	-0.000072753
215	6	-0.000213512	-0.000316262	0.000400780
216	6	-0.000183499	0.000330118	0.000137939
217	6	-0.000223259	-0.000114611	0.000780740
218	6	0.000277824	0.000395037	-0.000391041
219	6	-0.000411431	-0.000131516	0.001267535
220	1	-0.000130074	0.000014767	0.000082939
221	1	0.000511211	-0.000026917	-0.000752166
222	1	-0.000053229	-0.000015466	0.000007253
223	1	-0.000013211	0.000055095	-0.000371574
224	1	0.000145687	0.000154589	-0.000348638
225	1	0.000223677	-0.000191197	-0.000418160
226	1	0.000501713	0.000782843	-0.000023299
227	1	0.000197559	-0.000437621	-0.000086412
228	1	0.000271331	-0.000737450	-0.000155063
229	1	-0.000053747	0.000180950	0.000111980
230	1	0.000751754	-0.000606280	0.000724555
231	1	0.001019522	0.000341835	0.000308916
232	1	0.000299375	-0.000651947	0.000215867
233	1	-0.000452162	-0.000357054	-0.000129379
234	1	-0.000222686	0.000453491	0.000392899
235	1	0.000445431	-0.000059855	0.000253600
236	8	-0.000274544	-0.000117827	0.000091589
237	8	-0.002308214	0.000050092	0.001296959
238	6	0.000533999	0.000548269	0.000406465
239	6	-0.001065966	0.000011969	0.000684695
240	6	0.002326386	0.000177067	-0.001215892
241	6	-0.000632459	0.000314303	0.000121757
242	6	0.000521476	0.000045359	-0.000067062

243	6	-0.000104129	-0.000239781	-0.000720610
244	6	0.000019112	-0.000405513	0.000414283
245	6	0.000380320	0.000300246	-0.001123506
246	1	0.000409570	-0.000115571	-0.000345259
247	1	-0.000157685	0.000355909	0.000838318
248	1	0.000000943	-0.000132101	-0.000070210
249	1	0.000010757	-0.000013124	0.000308520
250	1	-0.000306207	0.000026202	0.000366438
251	1	-0.000109182	-0.000188720	0.000327129
252	8	0.000101886	0.000197680	0.000251632
253	8	0.000749942	0.000613438	0.000493995
254	6	-0.000258536	-0.000688965	-0.000182801
255	6	-0.000142768	0.000282270	0.000789498
256	6	-0.000129677	-0.000389865	-0.000489458
257	6	0.000254260	0.000132570	-0.000188842
258	6	-0.000249064	0.000120061	-0.000091068
259	6	0.000109480	0.000332424	-0.000492598
260	6	-0.000229942	0.000095292	0.000477259
261	6	-0.000016795	0.000355619	-0.001171820
262	1	0.000098612	0.000007639	-0.000288913
263	1	0.000099080	-0.000620385	0.000588402
264	1	-0.000057043	0.000081084	-0.000013615
265	1	0.000001261	-0.000000884	0.000377586
266	1	-0.000118081	-0.000145868	0.000255573
267	1	0.000266563	-0.000232945	0.000350442
268	8	-0.000680348	-0.000705755	-0.000190602
269	8	-0.000368752	0.000390162	-0.000435494
270	6	0.000319581	0.000458831	0.000080649
271	6	-0.000390911	-0.000450302	-0.000100305
272	6	0.000345862	0.000248408	0.000771768
273	6	-0.000259576	-0.000021499	-0.000856418
274	6	-0.000177251	0.000060628	-0.000203236
275	6	-0.000128368	-0.000051256	-0.000529798
276	6	0.000024586	-0.000330879	0.000382214
277	6	-0.000067232	-0.000075087	-0.001283569
278	1	0.000059199	-0.000006568	0.000219249
279	1	0.000367314	-0.000014432	0.000803241
280	1	-0.000050127	0.000063859	0.000005554
281	1	-0.000116714	0.000010787	0.000366013
282	1	0.000048525	-0.000077676	0.000374345
283	1	0.000118593	0.000224127	0.000409133
284	8	0.000172979	0.001006978	0.000705277
285	8	0.000455329	-0.000234197	-0.000357828
286	6	0.000060659	-0.000516952	-0.000123632
287	6	-0.000103786	0.000223874	0.000041866
288	6	-0.000152891	-0.000223506	0.000521745
289	6	0.000229640	0.000265230	-0.000644647
290	6	0.000219355	0.000042295	-0.000119318
291	6	0.000166861	0.000311824	-0.000471997
292	6	-0.000027378	0.000155273	0.000466021
293	6	0.000242044	0.000527955	-0.001092235
294	1	0.000028309	-0.000001445	0.000009351
295	1	-0.000663631	-0.000375291	0.000615744
296	1	0.000064711	-0.000048874	-0.000009505
297	1	-0.000121419	-0.000332087	0.000280891
298	1	0.000037314	-0.000145856	0.000307047
299	1	-0.000102227	-0.000049663	0.000336838
300	1	0.000157628	-0.000499752	0.000598174

301	1	-0.002733261	-0.000959724	0.001322326
302	1	0.000343308	0.000218931	-0.000516532
303	1	-0.002395099	0.001069027	-0.001351880
304	1	-0.000815982	-0.001595224	-0.000075737
305	1	0.000549085	0.000822497	-0.000893169
306	1	-0.001064135	-0.000738856	-0.000739051
307	1	0.000376688	-0.000857068	0.001082613
308	1	-0.000901433	0.000926301	0.000762516
309	1	0.002469643	-0.002608035	-0.002294524
310	8	-0.000068409	0.000294261	-0.000610239
311	8	0.002550349	0.002018397	-0.000649179
312	6	0.000245942	-0.000334928	0.000125607
313	6	0.001085459	0.000980202	-0.000395799
314	6	-0.001204113	-0.001665638	-0.000072238
315	6	-0.000086262	0.000504001	-0.000146111
316	6	0.000009713	-0.000394611	-0.000033521
317	6	-0.000620896	0.000230141	0.000036421
318	6	0.000535035	-0.000183296	0.000326483
319	6	-0.001266330	-0.000050898	-0.000156427
320	1	-0.000662246	-0.000524535	0.000263724
321	1	0.000776059	0.000230401	-0.000251701
322	1	-0.000065899	-0.000114865	-0.000070040
323	1	0.000343374	0.000317080	-0.000048645
324	1	0.000354867	-0.000060683	0.000129698
325	1	0.000331931	-0.000048421	-0.000063765
326	8	-0.000531287	-0.000172185	0.000377986
327	8	0.002085859	-0.002005436	0.000644920
328	6	0.000547948	0.000345973	-0.000004109
329	6	0.000769679	-0.001043307	0.000339848
330	6	-0.000857815	0.001611636	0.000030662
331	6	-0.000282646	-0.000419530	0.000060078
332	6	-0.000040290	0.000190486	-0.000018386
333	6	-0.000685999	-0.000081321	-0.000141842
334	6	0.000574412	0.000148512	-0.000156675
335	6	-0.001273666	0.000207700	-0.000062841
336	1	-0.000535732	0.000537709	-0.000207644
337	1	0.000696699	-0.000257112	0.000383459
338	1	-0.000029639	0.000113142	0.000087276
339	1	0.000349626	0.000014509	0.000109087
340	1	0.000396909	-0.000331391	0.000061206
341	1	0.000347968	0.000008091	-0.000067239
342	8	-0.000553496	-0.001565769	0.001310191
343	8	0.000529115	0.000921153	0.000808350
344	6	0.000229417	0.001181110	-0.000775603
345	6	-0.000021565	-0.000477555	0.000211885
346	6	0.000329707	-0.000151494	-0.000969416
347	6	-0.000877409	0.000383633	0.000326240
348	6	-0.000025563	0.000406543	-0.000146749
349	6	-0.000536305	-0.000233133	0.000201891
350	6	0.000451376	-0.000307234	0.000283207
351	6	-0.001274912	0.000477518	0.000184845
352	1	0.000001154	0.000130765	0.000098510
353	1	0.000744947	-0.000462849	-0.000222786
354	1	0.000029167	-0.000203163	0.000027238
355	1	0.000333605	-0.000019429	-0.000001977
356	1	0.000417499	-0.000188566	0.000016835
357	1	0.000377586	-0.000178249	-0.000304470
358	8	-0.000077275	0.001891630	-0.001593222

359	8	0.000362575	-0.000812211	-0.001125574
360	6	-0.000238297	-0.001537533	0.001024698
361	6	0.000278857	0.000576919	-0.000461916
362	6	0.000364146	0.000079770	0.001434310
363	6	-0.000972915	-0.000281587	-0.000652386
364	6	-0.000150623	-0.000379084	0.000387331
365	6	-0.000339309	0.000543118	-0.000356338
366	6	0.000498380	0.000135215	-0.000224580
367	6	-0.001295073	-0.000245006	-0.000363131
368	1	-0.000168971	-0.000191880	-0.000111814
369	1	0.000833200	0.000300324	0.000353882
370	1	0.000018322	0.000208578	0.000034995
371	1	0.000353425	0.000078066	0.000329794
372	1	0.000342694	-0.000043664	0.000031923
373	1	0.000417513	0.000135472	0.000049488
374	1	-0.000204383	0.000198783	-0.000423048
375	1	0.000715273	-0.000244784	-0.000483822
376	1	-0.000033791	-0.000124908	0.000110281
377	1	0.001827201	0.000146972	0.001047061
378	1	-0.000321283	-0.000259747	-0.000370759
379	1	-0.000362493	0.000139765	0.000770977
380	1	0.000401962	0.000263421	0.000090944
381	1	-0.000453455	0.000331897	0.000977120
382	1	0.000910186	-0.001057765	0.000381400
383	1	0.001215921	0.002138970	0.001395072
384	8	0.000367933	-0.000482468	0.000509539
385	8	-0.000348331	0.000281125	0.000053521
386	6	-0.000298709	0.000198889	-0.000154221
387	6	-0.000316874	0.000041150	0.000369079
388	6	-0.000254410	0.000231830	0.000211582
389	6	0.000722654	-0.000118501	-0.000195393
390	6	0.000101617	0.000127184	-0.000212740
391	6	0.000555821	-0.000127783	0.000080116
392	6	-0.000542410	-0.000179804	-0.000109246
393	6	0.001355975	-0.000247841	-0.000185547
394	1	0.000213065	-0.000139099	-0.000261314
395	1	-0.000728553	0.000102213	0.000453319
396	1	-0.000012248	-0.000064754	0.000094607
397	1	-0.000365185	0.000056937	-0.000070508
398	1	-0.000408011	-0.000033998	0.000089651
399	1	-0.000397100	0.000248107	0.000168740
400	8	-0.000292136	-0.000079506	0.000213513
401	8	-0.001726066	-0.001064865	-0.000857216
402	6	-0.000113056	0.000267379	-0.000323724
403	6	-0.000961963	-0.000393760	-0.000241883
404	6	0.000782186	0.000867798	0.000186720
405	6	0.000267715	-0.000183280	-0.000157545
406	6	0.000051485	0.000207312	0.000049862
407	6	0.000529357	-0.000104216	0.000143785
408	6	-0.000542880	0.000079436	0.000273929
409	6	0.001298763	0.000101198	0.000061126
410	1	0.000383919	0.000182395	0.000219610
411	1	-0.000644737	-0.000061302	-0.000497124
412	1	0.000045521	0.000115566	-0.000052077
413	1	-0.000385388	0.000005382	0.000111285
414	1	-0.000364840	0.000073042	-0.000074350
415	1	-0.000426620	-0.000223038	-0.000170827
416	8	-0.000085508	-0.000182882	0.000404953

417	8	-0.000799461	-0.000006792	-0.000482116
418	6	-0.000131823	0.000340899	-0.000077544
419	6	-0.000341827	0.000069155	-0.000010004
420	6	0.000000782	0.000368116	0.000045587
421	6	0.000443018	-0.000318358	0.000033581
422	6	0.000086297	-0.000162543	0.000088351
423	6	0.000562346	-0.000184393	-0.000032471
424	6	-0.000462566	0.000054229	0.000261226
425	6	0.001281755	-0.000434886	0.000143285
426	1	0.000183885	-0.000198557	0.000085831
427	1	-0.000736779	0.000616426	-0.000002954
428	1	-0.000006456	0.000153311	-0.000006533
429	1	-0.000415875	0.000136980	0.000062521
430	1	-0.000355363	0.000238641	-0.000207005
431	1	-0.000374347	0.000001355	-0.000017537
432	8	0.000209973	-0.000712563	-0.001853317
433	8	-0.000337632	0.001210415	-0.000806404
434	6	0.000180406	0.000662794	0.001294331
435	6	-0.000408527	0.000046069	-0.000707645
436	6	-0.000358497	-0.000874163	0.001311944
437	6	0.000937062	0.000507332	-0.000472914
438	6	0.000185909	0.000168393	0.000572755
439	6	0.000256123	-0.000251929	-0.000508528
440	6	-0.000431747	0.000140329	-0.000215095
441	6	0.001353176	0.000254764	-0.000105624
442	1	0.000189546	0.000056726	0.000041258
443	1	-0.000706612	-0.000471982	-0.000025256
444	1	-0.000015296	-0.000254131	-0.000050309
445	1	-0.000460800	-0.000191480	0.000269664
446	1	-0.000386473	0.000040007	-0.000023345
447	1	-0.000430435	-0.000113298	-0.000077602
448	1	-0.000625209	-0.000176503	0.000292743
449	1	-0.000042095	-0.000380175	-0.000788095
450	1	0.000395933	-0.000420058	0.000209403
451	1	-0.000229080	-0.000261164	0.000257591
452	1	0.001150444	-0.000798816	0.000438892
453	1	0.001099181	-0.002220817	-0.001231099
454	1	0.000874894	0.000438802	-0.000527769
455	1	-0.000065372	-0.000096012	0.000101847
456	1	0.000015077	-0.000008648	0.000119105
457	1	-0.000416807	0.000434274	0.000108215
458	6	-0.000277694	0.000892657	0.001584186
459	6	0.000296067	0.001441428	0.000586848
460	6	-0.000218041	-0.000522791	-0.001081299
461	6	-0.000502555	-0.001458036	-0.002523933
462	6	-0.000053973	-0.001329015	0.000955750
463	6	0.000201558	0.000437234	-0.000184558
464	1	0.001307724	-0.000931382	0.006039276
465	1	0.000234802	-0.005914037	0.001139126
466	1	-0.001383921	0.002177798	-0.006379784
467	1	-0.000225272	0.006164133	-0.001600672
468	17	-0.000405444	0.003458229	-0.001145803
469	1	0.000100974	0.000228013	0.000341664
470	1	0.000331191	0.000720169	0.000344697
471	1	0.000213512	-0.000085485	-0.000171541
472	8	0.001657613	0.001383220	0.001362251
473	1	-0.000830076	-0.000962298	-0.000248774
474	1	-0.000546227	-0.000184489	-0.000878980

475	8	0.000025577	0.000815597	-0.000773130
476	1	-0.000217580	-0.000226562	0.000251598
477	1	-0.000363489	-0.000315491	0.000405158
478	8	0.001625454	-0.001518955	-0.001639580
479	1	-0.000600773	0.000838654	0.000603372
480	1	-0.000699559	0.000320735	0.001023562
481	6	-0.000618168	0.002235493	-0.001806879
482	8	0.001559085	-0.002820688	0.006727966
483	6	0.003714343	-0.001790072	0.005832991
484	1	-0.000448239	-0.006065059	-0.002322700
485	1	0.003882105	0.003361450	-0.003801977
486	1	-0.005668383	0.003888000	0.000623233
487	8	-0.000421096	-0.004495260	-0.002492084

Sum of electronic and thermal Free Energies= -1398.952147
0 imaginary frequency

[**2**+**3f**]@C_R

Center Number	Atomic Number	Integrated Forces (Hartrees/Bohr) X	Y	Z
1	6	-0.000557177	0.001172903	-0.001872881
2	6	-0.001201137	0.000952846	0.001496579
3	7	-0.000829789	-0.000522084	0.003857777
4	6	-0.001008336	0.000954703	0.001574753
5	6	-0.000009120	-0.002019245	-0.001496797
6	6	0.000067030	0.000677384	-0.007228004
7	1	0.002643246	-0.003798756	-0.004701464
8	1	0.002570397	-0.005297439	0.003333214
9	1	-0.004675137	0.005080793	0.002371115
10	1	-0.000626084	0.003253952	-0.005441803
11	1	0.002334211	-0.012925274	-0.005334495
12	1	0.005451302	0.002881461	0.002459834
13	1	-0.001236461	-0.006042751	0.003039537
14	8	0.000070862	-0.000395490	-0.000079734
15	8	0.005004898	0.006394757	-0.001555139
16	6	-0.000215276	0.000087876	-0.000057192
17	6	-0.000074228	-0.000110941	-0.000156769
18	6	-0.001702205	-0.003808439	0.002249497
19	6	-0.008821470	0.006233174	-0.000668131
20	6	0.001930873	-0.008417404	0.000948855
21	6	-0.000021644	0.000072923	0.000465500
22	6	0.000044490	-0.000514914	-0.000256504
23	6	-0.000080167	0.000779957	0.001161825
24	1	0.000235663	-0.000022411	0.000067364
25	1	-0.000184866	-0.000232343	-0.000822871
26	1	-0.000121941	-0.000111791	0.000088725
27	1	-0.000039034	-0.000312282	-0.000316790
28	1	-0.000000619	-0.000072702	-0.000515703
29	1	0.000122707	-0.000232745	-0.000247221
30	8	-0.000618819	-0.000306214	0.000059729
31	8	0.001190755	-0.002704545	-0.000379427
32	6	0.000924274	0.000479854	-0.000346304
33	6	0.000207351	-0.001121089	0.000073853
34	6	-0.000260412	0.002135391	-0.000114847
35	6	0.000152449	-0.000565592	0.000420530

36	6	-0.000128543	0.000556876	-0.000315320
37	6	-0.000546101	0.000288685	0.000394821
38	6	0.000189138	-0.000098549	-0.000502792
39	6	-0.000723983	0.000641301	0.000918222
40	1	-0.000303349	0.000410687	-0.000094690
41	1	0.000607059	-0.000483391	-0.000254828
42	1	0.000051045	0.000177637	-0.000035337
43	1	0.000313274	-0.000364845	-0.000176477
44	1	0.000119102	-0.000189465	-0.000283308
45	1	0.000265785	-0.000086716	-0.000298763
46	8	-0.000959415	0.000979668	-0.000102425
47	8	-0.000077557	-0.000039442	0.000368516
48	6	0.000404862	-0.000451177	0.000186047
49	6	0.000481051	0.000768909	0.000325963
50	6	0.000469055	-0.000235589	-0.000289993
51	6	-0.000256913	0.000525192	0.000755413
52	6	-0.000136441	0.002884017	0.000042696
53	6	0.000016297	0.015556747	0.008530303
54	6	-0.000031342	-0.000308536	-0.000370074
55	6	-0.000773822	0.000692233	0.000901557
56	1	-0.000081574	-0.000463897	-0.000106617
57	1	0.000404616	-0.000125452	-0.000746557
58	1	0.000094857	-0.000046217	0.000064491
59	1	0.000201579	-0.000312681	-0.000187488
60	1	0.000159038	-0.000188894	-0.000316629
61	1	0.000406280	-0.000136853	-0.000305589
62	8	0.000861305	-0.000687767	0.001309145
63	8	-0.000145460	0.000029768	0.000407573
64	6	-0.000433443	0.000760845	-0.000649719
65	6	0.000037412	-0.000668673	0.000308246
66	6	0.000247724	0.000162424	-0.000707231
67	6	-0.000246208	0.000258134	0.000859573
68	6	-0.000130869	0.000292546	-0.000060923
69	6	0.000049392	-0.000110864	0.000426990
70	6	0.000287863	0.000025888	-0.000522653
71	6	-0.000064931	0.000605102	0.001314556
72	1	0.000221831	0.000352093	0.000090148
73	1	0.000059482	-0.000718574	-0.000509076
74	1	-0.000061886	0.000106342	-0.000052091
75	1	0.000087667	-0.000207185	-0.000305257
76	1	-0.000184051	-0.000267120	-0.000425042
77	1	0.000050242	-0.000106887	-0.000451683
78	8	-0.000056016	0.000265535	-0.000025213
79	8	0.000199186	0.003138494	0.000814764
80	6	0.000438472	-0.000251463	0.000693570
81	6	0.000096077	0.001276118	0.000003721
82	6	0.000166708	-0.002302403	0.000183551
83	6	0.000222347	0.000632373	-0.000334436
84	6	-0.000089464	-0.000519057	0.000313586
85	6	-0.000155266	-0.000351045	-0.000682602
86	6	-0.000139589	0.000163488	0.000476923
87	6	0.000091674	-0.000791415	-0.001056897
88	1	-0.000264317	-0.000525148	-0.000095544
89	1	0.000197828	0.000516740	0.000485885
90	1	0.000096647	-0.000120332	0.000062810
91	1	0.000018307	0.000156417	0.000404525
92	1	0.000062084	0.000464976	0.000219024
93	1	-0.000128018	0.000187469	0.000280829

94	8	-0.000318532	0.000607241	0.000037234
95	8	-0.000580615	-0.000246345	0.001478437
96	6	-0.000028251	-0.000343456	0.000167959
97	6	-0.000134843	-0.000545777	-0.000174332
98	6	-0.000198545	0.000447788	-0.000650865
99	6	0.000324833	-0.000367495	-0.000305918
100	6	0.000258992	-0.000057965	-0.000070738
101	6	0.000241776	-0.000134230	-0.000395191
102	6	-0.000151876	0.000536501	0.000184444
103	6	0.000738806	-0.000843214	-0.000915055
104	1	0.000279681	0.000324380	0.000090852
105	1	-0.000641363	0.000195833	0.000480555
106	1	-0.000082739	0.000082133	-0.000133243
107	1	-0.000103884	0.000270232	0.000274370
108	1	-0.000255035	0.000340960	0.000191370
109	1	-0.000331819	0.000087691	0.000389376
110	8	-0.000347978	-0.001394898	0.000131444
111	8	0.000490409	-0.000198590	-0.000270760
112	6	0.000124974	0.000793655	0.000094694
113	6	0.000081588	-0.000613535	0.000291877
114	6	-0.000072065	0.000582751	0.000322602
115	6	0.000188145	-0.000451279	-0.000693450
116	6	0.000016393	0.000055303	-0.000310342
117	6	0.000261989	-0.000513723	-0.000401880
118	6	-0.000331305	0.000280357	0.000296590
119	6	0.000066782	-0.000878268	-0.001125464
120	1	0.000049623	0.000421255	-0.000433386
121	1	-0.000182111	0.000209337	0.000857633
122	1	0.000078967	0.000061182	-0.000028631
123	1	0.000168748	0.000248842	0.000475901
124	1	-0.000046475	0.000374052	0.000245800
125	1	-0.000121480	0.000227079	0.000360405
126	8	0.001467344	0.001255301	-0.000534743
127	8	-0.000127931	-0.000342604	-0.000120471
128	6	-0.000823190	-0.001151516	0.000382710
129	6	0.000226055	0.000544789	-0.000331835
130	6	-0.000378566	0.000316870	0.000233419
131	6	0.000549355	-0.000425359	-0.000592729
132	6	-0.000175452	-0.000408268	0.000087492
133	6	0.000301152	0.000162416	-0.000471414
134	6	-0.000115646	0.000027275	0.000587151
135	6	0.000824301	-0.000586446	-0.001076800
136	1	0.000011390	-0.000281559	-0.000054561
137	1	-0.000330402	0.000725775	0.000359138
138	1	-0.000043048	-0.000105217	0.000032409
139	1	-0.000230788	0.000100710	0.000417293
140	1	-0.000139779	0.000220580	0.000285465
141	1	-0.000425250	0.000219325	0.000201053
142	8	-0.000010078	-0.000228089	-0.001350540
143	1	-0.000154958	-0.000243813	0.000611683
144	1	-0.000007657	0.000286925	0.000482543
145	8	-0.000289560	0.000548741	0.000066287
146	1	-0.000051925	0.000770634	-0.000596723
147	1	-0.000991151	0.000266970	0.000219975
148	8	-0.006523468	0.002922484	0.005078827
149	1	0.004804672	0.000786917	-0.003928764
150	1	0.003399541	-0.003263951	-0.000157850
151	8	-0.000128953	-0.000698840	0.000245924

152	1	-0.000049087	-0.000781237	0.000324067
153	1	-0.000627417	-0.000350518	-0.000853067
154	8	-0.001332729	0.001970946	-0.009086474
155	1	0.000390062	-0.002830402	0.005165448
156	1	0.001308176	0.001713399	0.005338834
157	1	0.000288676	0.000090244	-0.000728375
158	1	0.000503627	0.000116956	0.000169420
159	1	0.000076504	0.000395790	0.000077224
160	1	0.000189175	-0.000645720	-0.000388230
161	1	0.000041895	0.000093820	-0.000368526
162	8	-0.000044539	-0.000312065	0.000397426
163	8	0.002501086	-0.001052365	0.001327125
164	6	-0.000191988	-0.000169281	-0.000230378
165	6	0.000863750	-0.000667081	0.000619230
166	6	-0.002290965	0.000914217	-0.001632323
167	6	0.000794244	-0.000241858	-0.000133606
168	6	-0.000416789	0.000030574	-0.000202798
169	6	0.000235995	0.000577418	-0.000194301
170	6	-0.000183173	-0.000178386	0.000542490
171	6	-0.000056836	0.000885153	-0.000983575
172	1	-0.000396499	0.000278145	-0.000221970
173	1	0.000266769	-0.000815808	0.000141321
174	1	-0.000035937	0.000127404	0.000099583
175	1	-0.000057013	-0.000312912	0.000175262
176	1	0.000238215	-0.000337197	0.000340503
177	1	-0.000035758	-0.000239204	0.000397133
178	8	0.000167174	-0.000153362	0.000900822
179	8	-0.000942471	-0.000933432	-0.000394414
180	6	-0.000315319	-0.000261377	-0.000424502
181	6	-0.000330723	0.000013982	0.000245203
182	6	0.000552512	0.000475037	0.000277878
183	6	0.000080172	0.000398184	-0.000340525
184	6	0.000214469	-0.000088259	-0.000202794
185	6	0.000073929	0.000494026	-0.000206553
186	6	0.000151754	-0.000459118	0.000214839
187	6	0.000368230	0.001144804	-0.000735234
188	1	0.000172094	-0.000083867	-0.000189156
189	1	-0.000313045	-0.000344690	0.000625513
190	1	0.000005067	-0.000054704	-0.000091728
191	1	-0.000070143	-0.000418482	0.000139584
192	1	-0.000013237	-0.000267928	0.000286920
193	1	-0.000355004	-0.000318901	0.000285299
194	8	0.000455868	-0.001379560	-0.000395243
195	8	-0.000532027	0.000636602	0.000669565
196	6	-0.000571168	0.000585525	0.000281863
197	6	0.000314877	-0.000084200	0.000044927
198	6	0.000144871	-0.000451891	-0.000139029
199	6	0.000023197	0.000539385	-0.000499584
200	6	-0.000244460	0.000092458	-0.000103984
201	6	0.000130718	0.000255014	-0.000408228
202	6	-0.000115325	-0.000524309	0.000150033
203	6	0.000156374	0.000973200	-0.000958355
204	1	-0.000129165	-0.000042632	-0.000107744
205	1	0.000193779	-0.000335289	0.000633048
206	1	-0.000084384	-0.000038588	-0.000047087
207	1	-0.000030372	-0.000197801	0.000501189
208	1	-0.000119613	-0.000324491	0.000216293
209	1	0.000041922	-0.000343757	0.000193318

210	8	-0.000132641	-0.000084449	0.000755400
211	8	0.001157594	-0.000707696	-0.000410130
212	6	-0.000106475	-0.000244257	-0.000308427
213	6	0.000047567	0.000070371	0.000090323
214	6	-0.000516506	0.000222452	0.000234068
215	6	0.000268805	0.000285439	-0.000305944
216	6	0.000037437	0.000301055	0.000192742
217	6	0.000324891	0.000485765	-0.000299030
218	6	-0.000351401	-0.000213796	0.000395207
219	6	0.000363882	0.001163171	-0.000608049
220	1	0.000054939	-0.000065286	-0.000011564
221	1	-0.000509022	-0.000657804	0.000222936
222	1	0.000073014	0.000035183	0.000021576
223	1	-0.000157935	-0.000495031	0.000018057
224	1	0.000033517	-0.000320547	0.000199319
225	1	-0.000157857	-0.000270892	0.000271806
226	1	-0.000778640	0.000304055	0.000604384
227	1	-0.000047454	-0.000410783	-0.000187323
228	1	0.000243327	-0.000762731	-0.000907186
229	1	-0.000043683	0.000221044	0.000154636
230	1	-0.000477471	0.000345102	-0.000983703
231	1	0.000332313	0.000042242	0.000089845
232	1	-0.000064791	-0.000369947	-0.000841478
233	1	0.000161724	-0.000286473	-0.000064373
234	1	-0.000086449	0.000601392	0.000230495
235	1	0.000217064	0.000006862	0.000271439
236	8	0.000040651	0.000497018	-0.000053539
237	8	0.001884029	0.001617050	0.000478205
238	6	-0.000327722	0.000065534	-0.000047173
239	6	0.000811799	0.000835210	0.000152268
240	6	-0.002183024	-0.001404079	0.000010797
241	6	0.000403871	0.000445817	0.000338833
242	6	-0.000416977	-0.000131922	-0.000014318
243	6	0.000112384	-0.000482672	0.000361545
244	6	0.000243149	0.000074059	-0.000658312
245	6	-0.000570754	-0.000851387	0.000906615
246	1	-0.000274330	-0.000464162	-0.000043295
247	1	0.000071353	0.000712307	-0.000110860
248	1	0.000096396	-0.000140181	-0.000105869
249	1	0.000308058	0.000357503	-0.000098815
250	1	0.000186919	0.000210523	-0.000394468
251	1	0.000023023	0.000261087	-0.000210076
252	8	0.000028710	0.000057010	-0.000020821
253	8	-0.001590801	-0.000435545	-0.000754482
254	6	0.000240493	0.000180097	-0.000404898
255	6	-0.000279926	-0.000406680	-0.000322569
256	6	0.001136224	0.000253450	0.000279103
257	6	-0.000357032	-0.000440250	0.000150222
258	6	0.000234637	0.000017088	0.000294239
259	6	-0.000090107	-0.000613591	0.000634988
260	6	0.000194023	0.000506033	-0.000184022
261	6	0.000150851	-0.001039426	0.000932333
262	1	0.000136810	0.000202426	0.000171230
263	1	0.000065335	0.000332177	-0.000834696
264	1	-0.000043819	0.000065027	0.000104350
265	1	-0.000060415	0.000387074	-0.000168008
266	1	0.000073352	0.000282705	-0.000259651
267	1	-0.000245359	0.000248413	-0.000432956

268	8	0.000068489	-0.000109770	-0.000140895
269	8	0.000149191	0.000984163	0.000985228
270	6	-0.000426487	0.000032516	-0.000321912
271	6	-0.000086227	0.000472281	0.000046851
272	6	0.000038762	-0.000667676	-0.000512713
273	6	0.000080109	0.000073799	0.000443613
274	6	0.000159585	-0.000429542	-0.000250105
275	6	0.000337395	-0.000563272	0.000612008
276	6	-0.000141240	0.000142046	-0.000566850
277	6	0.000239385	-0.001038120	0.000785374
278	1	0.000131960	-0.000115302	-0.000001761
279	1	-0.000399375	0.000526909	-0.000390243
280	1	0.000038669	0.000011766	0.000049783
281	1	-0.000050852	0.000238346	-0.000352824
282	1	-0.000230408	0.000454578	-0.000106636
283	1	0.000065413	0.000331529	-0.000187793
284	8	-0.000298680	0.001284987	0.000602799
285	8	-0.000569071	-0.000361735	-0.000346232
286	6	0.000021033	-0.000708013	-0.000416163
287	6	0.000074888	0.000323970	0.000211548
288	6	0.000311080	0.000337982	-0.000476764
289	6	-0.000352078	-0.000485160	0.000633270
290	6	-0.000253116	-0.000287754	-0.000146727
291	6	-0.000183166	0.000002424	0.000473269
292	6	-0.000098179	0.000522800	-0.000119444
293	6	-0.000282150	-0.000981454	0.000917041
294	1	-0.000056167	-0.000002616	0.000030971
295	1	0.000573608	0.000523060	-0.000495491
296	1	-0.000056246	-0.000024827	-0.000030279
297	1	-0.000028397	0.000280602	-0.000289467
298	1	0.000100389	0.000347139	-0.000141917
299	1	0.000225817	0.000212228	-0.000435965
300	1	-0.000089274	0.000316384	-0.000612870
301	1	0.002700468	0.001163132	-0.000897566
302	1	-0.000368584	-0.000453188	0.000296048
303	1	0.002132679	-0.000421015	0.001998049
304	1	0.001191093	0.000034997	-0.001240964
305	1	-0.000845359	-0.000641357	0.001013889
306	1	0.001092859	-0.001093939	-0.000203019
307	1	-0.000142709	0.000633677	-0.001246777
308	1	0.000686758	0.000922626	0.000658224
309	1	-0.001733009	-0.003228463	-0.001733834
310	8	0.000286075	-0.000198855	0.000673078
311	8	-0.003011965	-0.000021148	0.001640874
312	6	-0.000379823	-0.000315899	-0.000690686
313	6	-0.001185921	0.000048008	0.000907953
314	6	0.001789997	-0.000787253	-0.001290719
315	6	-0.000150853	0.000147186	0.000595962
316	6	0.000162327	-0.000220729	-0.000367550
317	6	0.000654188	0.000245752	0.000422997
318	6	-0.000478557	0.000097082	-0.000404319
319	6	0.001298444	-0.000006383	0.000334945
320	1	0.000797725	0.000115204	-0.000416633
321	1	-0.000775279	-0.000295241	0.000138259
322	1	0.000102699	-0.000067717	-0.000098282
323	1	-0.000345155	-0.000125266	-0.000135046
324	1	-0.000473419	0.000069692	0.000119228
325	1	-0.000347343	0.000068153	-0.000189666

326	8	0.000583066	0.000311453	-0.000369022
327	8	-0.001378956	-0.000487659	-0.002564336
328	6	-0.000739591	0.000140371	0.000323475
329	6	-0.000544723	-0.000251959	-0.001324130
330	6	0.000325342	0.000744339	0.001757995
331	6	0.000431662	0.000031990	-0.000535468
332	6	-0.000132445	0.000179490	0.000281182
333	6	0.000722285	-0.000120245	0.000111708
334	6	-0.000641271	-0.000205511	0.000078352
335	6	0.001219357	0.000313173	0.000436126
336	1	0.000377739	0.000116711	0.000684264
337	1	-0.000600157	0.000079001	-0.000501037
338	1	-0.000017136	0.000115508	0.000139429
339	1	-0.000338555	-0.000122334	-0.000016233
340	1	-0.000343999	0.000013576	-0.000099951
341	1	-0.000296170	-0.000163123	-0.000396206
342	8	0.001046101	0.000761161	-0.001854161
343	8	-0.000696848	0.001238514	0.000441070
344	6	-0.000597160	-0.000213807	0.001457487
345	6	0.000195879	0.000115569	-0.000599716
346	6	-0.000332451	-0.001069484	0.000149969
347	6	0.000878793	0.000681207	0.000362557
348	6	-0.000105988	0.000017853	0.000500324
349	6	0.000613607	0.000049673	-0.000282454
350	6	-0.000392914	0.000075772	-0.000468750
351	6	0.001167682	0.000529212	0.000576640
352	1	-0.000012011	0.000056632	0.000104194
353	1	-0.000626687	-0.000422224	-0.000440851
354	1	0.000059211	-0.000100585	-0.000126710
355	1	-0.000341225	-0.000060430	-0.000090331
356	1	-0.000346891	-0.000128796	-0.000265254
357	1	-0.000370947	-0.000393768	-0.000087145
358	8	-0.000275434	-0.000613615	0.002101873
359	8	-0.000085457	-0.001145663	-0.000516261
360	6	0.000385663	0.000290653	-0.001442892
361	6	-0.000466031	-0.000093711	0.000538995
362	6	-0.000346189	0.001092629	-0.000326013
363	6	0.000953387	-0.000371273	0.000152053
364	6	0.000284676	0.000081914	-0.000383195
365	6	0.000296294	-0.000085323	0.000542527
366	6	-0.000519313	-0.000239039	0.000111071
367	6	0.001347296	-0.000154190	0.000267868
368	1	0.000283845	-0.000177477	-0.000013399
369	1	-0.000824818	0.000304059	0.000005990
370	1	-0.000079550	0.000084818	0.000159769
371	1	-0.000413746	0.000259989	-0.000192661
372	1	-0.000318692	-0.000057245	-0.000131170
373	1	-0.000445633	0.000004008	-0.000013493
374	1	0.000307719	-0.000163968	0.000489682
375	1	-0.000645609	-0.000615666	-0.000156117
376	1	0.000184868	0.000012046	-0.000115326
377	1	-0.000745636	0.001097052	-0.000462380
378	1	0.000548513	-0.000361436	-0.000405149
379	1	0.000239275	0.000986513	-0.000028555
380	1	-0.000669857	0.000066521	0.000210196
381	1	0.000547332	0.000901148	0.000122535
382	1	-0.000054058	-0.000008043	0.000465357
383	1	-0.001715038	0.001812653	0.000893669

384	8	-0.000478459	-0.000097621	-0.000217942
385	8	0.000506631	0.000391539	0.000915456
386	6	0.000587806	0.000237852	-0.000245299
387	6	0.000297904	0.000420164	0.000466084
388	6	0.000020535	-0.000141539	-0.000407834
389	6	-0.000385675	-0.000104620	0.000074231
390	6	0.000054651	-0.000214475	-0.000145602
391	6	-0.000720034	-0.000210677	-0.000061356
392	6	0.000600944	0.000016267	-0.000134267
393	6	-0.001230240	-0.000457581	-0.000302225
394	1	-0.000267091	-0.000219310	-0.000255581
395	1	0.000648195	0.000540533	0.000132757
396	1	0.000012889	0.000026583	-0.000155792
397	1	0.000314375	0.000007517	0.000129417
398	1	0.000408802	0.000135364	-0.000005759
399	1	0.000297563	0.000325202	0.000256398
400	8	-0.000675828	-0.000095547	0.000730767
401	8	0.000448779	-0.000474999	0.000203241
402	6	0.000135125	0.000093099	-0.000378701
403	6	0.000212064	-0.000060035	0.000167500
404	6	0.000032354	-0.000430392	-0.000077470
405	6	-0.000609874	0.000182400	-0.000099791
406	6	-0.000035502	0.000054276	-0.000334473
407	6	-0.000379650	-0.000151804	0.000154631
408	6	0.000480137	0.000345384	0.000112455
409	6	-0.001371340	-0.000005934	-0.000260282
410	1	-0.000074803	0.000301832	0.000090592
411	1	0.000641294	-0.000330292	0.000297930
412	1	-0.000067039	-0.000013594	0.000094130
413	1	0.000394679	0.000115505	0.000063972
414	1	0.000354389	-0.000006727	0.000204414
415	1	0.000485711	-0.000196402	-0.000021545
416	8	-0.000310246	0.001074166	0.000154814
417	8	0.000652206	-0.000033230	-0.000864905
418	6	-0.000226489	-0.000943770	-0.000182614
419	6	0.000537922	0.000387323	-0.000102950
420	6	-0.000072262	-0.000504067	0.000818439
421	6	-0.000477465	-0.000006205	-0.000494488
422	6	-0.000052359	-0.000281413	-0.000110784
423	6	-0.000479585	0.000215902	0.000149747
424	6	0.000512972	0.000195286	-0.000012276
425	6	-0.001146364	-0.000117454	-0.000625953
426	1	-0.000240764	-0.000076619	-0.000069633
427	1	0.000547644	0.000225150	0.000601665
428	1	-0.000050556	0.000137845	0.000053449
429	1	0.000317457	0.000134536	0.000193916
430	1	0.000345103	-0.000111070	0.000386550
431	1	0.000360117	0.000023113	0.000057593
432	8	-0.000646679	-0.000716075	-0.000462579
433	8	-0.000338688	-0.000129631	0.000424964
434	6	0.000383994	0.000130146	0.000058681
435	6	0.000053463	-0.000373937	0.000100160
436	6	0.000739766	0.000626994	-0.000824204
437	6	-0.000970283	-0.000213994	0.000212354
438	6	-0.000287365	0.000249015	-0.000092105
439	6	-0.000540620	-0.000262135	-0.000037254
440	6	0.000410493	-0.000106383	0.000332697
441	6	-0.001351250	-0.000305139	-0.000237692

442	1	-0.000107090	0.000090333	0.000098188
443	1	0.000880094	0.000017789	-0.000215021
444	1	0.000021838	-0.000082315	-0.000062262
445	1	0.000465046	0.000228900	-0.000175797
446	1	0.000328738	0.000105503	0.000194226
447	1	0.000455855	-0.000027740	0.000092256
448	1	0.000573620	0.000583218	0.000254451
449	1	0.000365449	-0.000903529	0.000027291
450	1	-0.000321891	-0.000070309	-0.000447392
451	1	0.000352935	0.000054098	-0.000285154
452	1	-0.001074820	0.000020945	0.000658755
453	1	-0.000472112	-0.002270870	-0.001642470
454	1	-0.000867953	-0.000458511	0.000351138
455	1	0.000379252	-0.000113290	-0.000051954
456	1	0.000430647	0.000290600	-0.000366381
457	1	0.000270567	0.000057914	0.000345152
458	6	-0.000348609	0.003406505	0.001359248
459	6	-0.000283955	0.000155058	0.000329486
460	6	0.000057345	0.000380214	-0.000373505
461	6	0.001953410	-0.006048839	-0.001957009
462	6	0.000076491	0.000114485	0.000521530
463	6	0.000165536	-0.000033541	-0.000265792
464	1	-0.000320911	0.005510442	-0.003791900
465	1	0.001799204	-0.001987423	-0.006269109
466	1	-0.000104285	-0.005866955	0.003657849
467	1	-0.001974778	0.001448956	0.005989487
468	17	-0.000588443	0.000644218	0.003734981
469	1	-0.000015999	0.000339040	-0.000092300
470	1	-0.000567776	0.000524368	0.000462599
471	1	-0.000385211	-0.000331938	-0.000171111
472	8	-0.001840237	0.001630847	0.000305946
473	1	0.000980553	-0.000706530	0.000000844
474	1	0.000594872	-0.000806530	0.000202627
475	8	0.001882606	0.000095795	-0.001019170
476	1	-0.000826678	-0.000475412	0.000494096
477	1	-0.000769110	0.000218780	0.000267581
478	8	-0.000962983	-0.001923968	-0.000950051
479	1	0.000275147	0.000593986	0.000629965
480	1	0.000495253	0.001193767	0.000029306
481	6	0.000118121	-0.002185970	0.003069664
482	8	-0.000266297	0.005252904	-0.006487497
483	7	-0.000370020	-0.001390551	0.000398454
484	8	-0.000516883	-0.001023260	0.002876959
485	8	0.000293944	0.001024329	-0.003263534

Sum of electronic and thermal Free Energies= -1488.942347
0 imaginary frequency

α-TS1

Center Number	Atomic Number	Integrated Forces (Hartrees/Bohr) X	Y	Z
1	6	0.001228555	-0.001593348	0.004948236
2	6	0.000303096	0.002290049	-0.003708338
3	7	0.001869080	-0.001647162	0.007257118
4	6	-0.001455342	-0.001220535	-0.003751821

5	6	-0.000372193	0.004077766	-0.000532572
6	6	-0.001792251	-0.002599559	-0.003349159
7	1	-0.000243583	-0.006745366	-0.001409172
8	1	0.001772646	-0.002478553	0.005588207
9	1	-0.000471664	0.006695042	-0.001965770
10	1	0.000229454	-0.000371773	-0.007296209
11	1	-0.006115483	0.003092903	0.000192943
12	1	0.003719474	0.004748504	-0.001437882
13	1	0.001804181	-0.002745335	0.005626949
14	8	0.001407190	0.001074701	0.000000800
15	8	0.004823122	0.006022680	-0.003750210
16	6	-0.001493163	-0.001559665	-0.000230245
17	6	0.000742938	0.000442831	-0.000803566
18	6	-0.001714746	-0.004418384	0.003542066
19	6	0.000367503	0.000553655	-0.000466485
20	6	-0.000033281	-0.000672857	0.000626965
21	6	0.000758327	0.000830666	0.001050672
22	6	-0.000554975	-0.000641963	-0.000192353
23	6	0.000577057	0.000945267	0.001046885
24	1	-0.000138928	0.000085405	0.000243187
25	1	-0.000290619	-0.000296094	-0.000753575
26	1	-0.000175583	-0.000107075	0.000096116
27	1	-0.000163778	-0.000407897	-0.000320181
28	1	-0.000235731	-0.000120914	-0.000437716
29	1	-0.000003616	-0.000225439	-0.000245898
30	8	-0.000163807	-0.000158063	-0.000408966
31	8	0.001527290	-0.002436260	-0.000598575
32	6	0.000291778	0.000343636	-0.000019334
33	6	0.000497967	-0.000992733	-0.000130294
34	6	-0.000435817	0.001704335	-0.000199812
35	6	0.000107112	-0.000017158	0.000429620
36	6	-0.000202632	0.000057426	-0.000049824
37	6	-0.000161230	0.000303846	0.000158457
38	6	-0.000241150	-0.000311700	-0.000576209
39	6	-0.000366647	0.000756313	0.001043264
40	1	-0.000327639	0.000466288	-0.000018124
41	1	0.000494622	-0.000371701	-0.000313227
42	1	0.000030002	0.000179152	-0.000064598
43	1	0.000217921	-0.000445133	-0.000145188
44	1	0.000036416	-0.000201029	-0.000355500
45	1	0.000180002	-0.000098723	-0.000314785
46	8	-0.000522559	0.000769740	-0.001144884
47	8	-0.000045526	0.000197459	0.000072795
48	6	0.000743136	-0.000216628	0.001437687
49	6	-0.000298285	-0.000107822	-0.000778681
50	6	0.000626861	-0.000403111	-0.000115744
51	6	-0.000329202	0.000370339	0.000463416
52	6	0.000184356	-0.000075481	0.000235635
53	6	0.000044723	0.000426443	-0.000219244
54	6	0.000075124	-0.000455645	-0.000144421
55	6	-0.000698035	0.000786339	0.000773631
56	1	0.000041713	0.000098215	0.000426801
57	1	0.000283346	-0.000148357	-0.000761459
58	1	0.000096946	-0.000044101	0.000094006
59	1	0.000203695	-0.000348450	-0.000195338
60	1	0.000117134	-0.000187041	-0.000293084
61	1	0.000361999	-0.000168021	-0.000256499
62	8	0.002050646	-0.001185894	0.001575417

63	8	0.001138125	0.000463253	0.001653328
64	6	-0.001601094	0.001278965	-0.000963146
65	6	0.000766818	-0.000836319	0.001028956
66	6	-0.001449379	-0.000995836	-0.002653338
67	6	0.000448183	0.001284494	0.001451670
68	6	-0.000850808	0.000377150	-0.000423502
69	6	0.000588060	-0.000360156	0.000556104
70	6	0.000295097	0.000016057	-0.000393284
71	6	0.000216499	0.000797789	0.001281001
72	1	0.000015713	0.000421314	-0.000137978
73	1	0.000011286	-0.000742991	-0.000454880
74	1	-0.000280340	-0.000074406	-0.000077629
75	1	0.000028480	-0.000270499	-0.000339111
76	1	-0.000275831	-0.000360637	-0.000363809
77	1	-0.000028780	-0.000148905	-0.000478583
78	8	-0.000388597	0.000189308	-0.000182462
79	8	0.000095333	0.003159443	0.000759720
80	6	0.000800446	-0.000277853	0.000684632
81	6	-0.000054913	0.001311949	-0.000062268
82	6	0.000264026	-0.002279395	0.000203394
83	6	0.000099963	0.000587124	-0.000350810
84	6	0.000033153	-0.000476368	0.000243614
85	6	-0.000326270	-0.000388220	-0.000664762
86	6	-0.000028900	0.000148911	0.000509737
87	6	-0.000134379	-0.000800667	-0.001002518
88	1	-0.000190685	-0.000584231	-0.000097399
89	1	0.000306772	0.000585535	0.000553046
90	1	0.000095064	-0.000141188	0.000073981
91	1	0.000114241	0.000171139	0.000357451
92	1	0.000106470	0.000424850	0.000200603
93	1	-0.000048299	0.000185236	0.000267969
94	8	0.000190304	0.000129257	0.000187409
95	8	-0.001011926	-0.000324667	0.002846162
96	6	-0.000513235	0.000149991	-0.000164019
97	6	-0.000206483	-0.000341666	0.000839110
98	6	-0.000001369	0.000758977	-0.001819386
99	6	-0.000041793	-0.000446960	0.000073691
100	6	0.000267701	0.000213131	-0.000125367
101	6	0.000260829	-0.000288175	-0.000454217
102	6	-0.000053510	0.000463671	0.000262615
103	6	0.000579687	-0.000663760	-0.001004765
104	1	0.000205725	0.000217077	-0.000322173
105	1	-0.000521917	0.000136048	0.000530455
106	1	-0.000074104	0.000072159	-0.000125129
107	1	-0.000069876	0.000219041	0.000292991
108	1	-0.000244172	0.000274932	0.000211685
109	1	-0.000270599	0.000026042	0.000459540
110	8	-0.000981980	-0.001602672	0.000457300
111	8	0.000188933	-0.000301911	-0.000359009
112	6	0.000615774	0.001033066	-0.000395708
113	6	-0.000057235	-0.000764577	0.000397896
114	6	0.000228215	0.000790782	0.000597751
115	6	-0.000093882	-0.000578285	-0.000856384
116	6	0.000172010	0.000153436	-0.000242320
117	6	0.000060365	-0.000530447	-0.000273471
118	6	-0.000293681	0.000269709	0.000274418
119	6	-0.000130082	-0.000855300	-0.001066380
120	1	-0.000186888	0.000251319	-0.000280371

121	1	0.000029909	0.000219438	0.000862867
122	1	0.000093348	0.000060423	-0.000052676
123	1	0.000209514	0.000226158	0.000354437
124	1	0.000009396	0.000370430	0.000237538
125	1	-0.000039436	0.000205605	0.000354924
126	8	0.001372288	0.001364645	-0.001002111
127	8	0.000146498	-0.000321114	-0.000127034
128	6	-0.000833855	-0.001217781	0.000560478
129	6	0.000295002	0.000496248	-0.000390040
130	6	-0.000654634	0.000277628	0.000341636
131	6	0.000570218	-0.000507031	-0.000598367
132	6	-0.000208903	-0.000377805	0.000078718
133	6	0.000195093	0.000135245	-0.000464699
134	6	0.000041048	0.000086515	0.000491331
135	6	0.000653180	-0.000545565	-0.001064524
136	1	-0.000001730	-0.000142155	0.000137814
137	1	-0.000426611	0.000721552	0.000391576
138	1	-0.000076664	-0.000062080	0.000055686
139	1	-0.000160344	0.000082950	0.000389260
140	1	-0.000109116	0.000209817	0.000277646
141	1	-0.000329002	0.000210451	0.000238351
142	8	0.000095364	-0.000077939	-0.000927793
143	1	0.000079289	-0.000117272	0.000457318
144	1	-0.000153323	0.000355320	0.000230672
145	8	-0.000401686	0.000467312	0.000018089
146	1	-0.000096928	0.000756210	-0.000601552
147	1	-0.000904301	0.000255328	0.000382965
148	8	-0.004073111	0.002038413	0.006429197
149	1	0.003952193	0.000570505	-0.004625446
150	1	0.001304515	-0.001875300	-0.000655214
151	8	0.000099646	-0.000649452	0.000046113
152	1	0.000011176	-0.000751160	0.000494423
153	1	-0.000773732	-0.000468491	-0.000643283
154	8	-0.004029825	0.000600373	-0.009345333
155	1	0.002046039	-0.002405441	0.005508959
156	1	0.002747681	0.001578830	0.005685094
157	1	0.000112692	0.000112262	-0.000312488
158	1	0.000324600	-0.000256522	-0.000147347
159	1	0.000134756	0.000249317	0.000180156
160	1	0.000278995	-0.000736466	-0.000368296
161	1	-0.001058692	-0.000357700	-0.000422585
162	8	0.000170674	-0.000371426	0.000139228
163	8	0.002516029	-0.000836433	0.000895810
164	6	-0.000261563	-0.000206333	-0.000207112
165	6	0.000978606	-0.000572205	0.000456200
166	6	-0.002368311	0.000734752	-0.001167857
167	6	0.000698942	-0.000194903	-0.000071198
168	6	-0.000527106	-0.000009091	-0.000126449
169	6	0.000210573	0.000665741	-0.000246124
170	6	-0.000081769	-0.000185365	0.000508468
171	6	-0.000318173	0.000838949	-0.000874486
172	1	-0.000401278	0.000300183	-0.000090695
173	1	0.000316996	-0.000788543	0.000154371
174	1	-0.000014032	0.000143717	0.000095176
175	1	-0.000002063	-0.000307804	0.000153869
176	1	0.000259190	-0.000294962	0.000211157
177	1	0.000069270	-0.000189477	0.000362387
178	8	0.000240932	-0.000268395	0.000807095

179	8	0.000007539	-0.000331769	-0.000213050
180	6	-0.000359245	0.000414081	-0.000510526
181	6	0.000693124	-0.000932121	0.000384923
182	6	-0.000520163	0.000276263	0.000164582
183	6	0.000231251	0.000399781	-0.000254327
184	6	-0.000072695	-0.000125235	-0.000278316
185	6	0.000128859	0.000319478	0.000006254
186	6	0.000196052	-0.000504229	0.000013410
187	6	0.000277041	0.001098641	-0.000679209
188	1	-0.000232571	0.000203838	-0.000134267
189	1	-0.000301171	-0.000301177	0.000656322
190	1	-0.000038146	-0.000068893	-0.000095426
191	1	-0.000036917	-0.000429013	0.000139640
192	1	0.000015819	-0.000215438	0.000264311
193	1	-0.000289044	-0.000303732	0.000281318
194	8	0.000164781	-0.001038642	-0.000493292
195	8	-0.000593217	0.000731241	0.000625031
196	6	-0.000109913	0.000374483	0.000240661
197	6	0.000120536	0.000016902	0.000060878
198	6	0.000173458	-0.000525612	-0.000309511
199	6	-0.000108124	0.000455890	-0.000254285
200	6	-0.000149393	-0.000025494	-0.000215592
201	6	-0.000122255	0.000282705	-0.000487922
202	6	0.000017325	-0.000449788	0.000146033
203	6	-0.000070320	0.000783399	-0.000968415
204	1	-0.000059938	-0.000061912	-0.000009275
205	1	0.000489515	-0.000424837	0.000655178
206	1	-0.000076732	-0.000023699	-0.000049975
207	1	0.000054614	-0.000073413	0.000499602
208	1	-0.000098257	-0.000251682	0.000267097
209	1	0.000088677	-0.000276556	0.000186644
210	8	0.001211115	0.000406902	0.000529525
211	8	0.001038976	-0.000304439	-0.000521844
212	6	-0.001098783	-0.000145597	0.000018864
213	6	0.000432802	-0.000108751	0.000394026
214	6	-0.000584741	-0.000167148	0.000033212
215	6	0.000309071	0.000362385	-0.000273320
216	6	0.000028651	0.000285980	0.000135288
217	6	0.000392604	0.000679163	-0.000359768
218	6	-0.000334727	-0.000215520	0.000443970
219	6	0.000255137	0.001163205	-0.000595369
220	1	-0.000026581	0.000023824	-0.000215383
221	1	-0.000396036	-0.000730684	0.000232901
222	1	0.000045008	0.000015679	0.000010084
223	1	-0.000101722	-0.000482709	0.000025231
224	1	0.000063311	-0.000300941	0.000187562
225	1	-0.000130011	-0.000289131	0.000273348
226	1	-0.000774521	0.000215846	0.000708211
227	1	-0.000121284	0.000187120	-0.000292780
228	1	-0.000084162	-0.000546399	-0.000633348
229	1	0.000049230	0.000073153	0.000257539
230	1	-0.001008232	0.000157965	-0.001080581
231	1	-0.000775680	-0.000272077	0.000447177
232	1	-0.000337534	-0.000172481	-0.000785595
233	1	0.000008122	-0.000243719	-0.000055496
234	1	0.000211334	0.000695190	0.000429154
235	1	-0.000404249	-0.000052937	0.000189067
236	8	0.000556877	-0.000278164	-0.000090182

237	8	0.002169906	0.001618647	-0.000132801
238	6	-0.000899310	0.000685752	0.000195307
239	6	0.001012062	0.000681308	-0.000180569
240	6	-0.002284752	-0.001466872	0.000517456
241	6	0.000614240	0.000395665	0.000192060
242	6	-0.000594099	-0.000185181	0.000119364
243	6	0.000362996	-0.000835533	0.000181818
244	6	-0.000015079	0.000172622	-0.000502206
245	6	-0.000312007	-0.000963685	0.000834195
246	1	-0.000380659	-0.000266825	0.000036973
247	1	0.000131953	0.000787521	-0.000107492
248	1	0.000081159	-0.000131943	-0.000080848
249	1	0.000277074	0.000443147	-0.000147424
250	1	0.000099383	0.000234783	-0.000392343
251	1	-0.000042747	0.000302255	-0.000189747
252	8	-0.000044877	0.000166821	-0.000235822
253	8	-0.002297052	0.000010543	-0.001555067
254	6	0.000354209	0.000090119	-0.000183652
255	6	-0.000866694	-0.000024041	-0.000714023
256	6	0.002359345	-0.000383220	0.001174814
257	6	-0.000527920	-0.000396701	-0.000419111
258	6	0.000405209	0.000049745	0.000152773
259	6	-0.000118898	-0.000429031	0.000436436
260	6	-0.000141642	0.000673196	0.000109684
261	6	0.000509308	-0.001108061	0.000569225
262	1	0.000361490	0.000048935	0.000386461
263	1	-0.000123670	0.000290225	-0.000807361
264	1	-0.000056555	0.000073843	0.000175428
265	1	-0.000165258	0.000440162	-0.000104039
266	1	-0.000006493	0.000254549	-0.000200836
267	1	-0.000326269	0.000237772	-0.000317265
268	8	0.000719733	-0.000024043	-0.001336840
269	8	0.000833692	-0.000339420	0.000293256
270	6	-0.000151927	0.000097574	0.000688086
271	6	0.000130359	-0.000148348	-0.000744709
272	6	-0.000612393	0.000918903	0.000183364
273	6	0.000554836	-0.000717964	0.000391647
274	6	0.000255231	-0.000023058	0.000177660
275	6	0.000258923	-0.000491180	0.000024581
276	6	-0.000173198	0.000105141	-0.000537496
277	6	0.000474272	-0.001072726	0.000693423
278	1	-0.000128881	0.000069268	0.000229074
279	1	-0.000609500	0.000616268	-0.000290931
280	1	0.000052313	0.000022231	0.000023203
281	1	-0.000155578	0.000235365	-0.000328056
282	1	-0.000276209	0.000434705	-0.000042952
283	1	-0.000002592	0.000331016	-0.000180404
284	8	-0.000217591	0.001137470	0.000347262
285	8	-0.000426134	-0.000622277	-0.000736237
286	6	0.000077615	-0.000484183	-0.000346094
287	6	0.000142189	0.000155425	-0.000007562
288	6	0.000091218	0.000395150	0.000157185
289	6	-0.000228328	-0.000496278	0.000221059
290	6	-0.000228664	-0.000013394	0.000160421
291	6	-0.000191449	-0.000350321	0.000443808
292	6	0.000110029	0.000536108	-0.000042893
293	6	-0.000283209	-0.000942394	0.000842822
294	1	-0.000213361	-0.000065810	0.000087815

295	1	0.000427491	0.000522728	-0.000490812
296	1	-0.000059530	-0.000022436	0.000009953
297	1	-0.000046048	0.000237905	-0.000259387
298	1	0.000102524	0.000371072	-0.000148095
299	1	0.000215507	0.000177578	-0.000406927
300	1	-0.000338863	0.000435581	-0.000469477
301	1	0.002342599	0.001129703	-0.001148367
302	1	-0.000228756	-0.000506467	0.000548934
303	1	0.002462782	-0.000275632	0.001818830
304	1	0.001640574	-0.000372301	-0.002285815
305	1	-0.000500143	-0.000719053	0.001155857
306	1	0.001114707	-0.000871623	-0.000344499
307	1	-0.000372284	0.000606171	-0.001124594
308	1	0.000892322	0.000797132	0.000547484
309	1	-0.002238682	-0.003167870	-0.000310158
310	8	0.000588208	-0.000400069	0.000522950
311	8	-0.002486940	-0.000046259	0.001801647
312	6	-0.000385664	-0.000105859	-0.000507971
313	6	-0.000852592	-0.000081716	0.001014622
314	6	0.001263926	-0.000656090	-0.001294492
315	6	0.000179651	0.000087383	0.000502154
316	6	0.000100264	-0.000097255	-0.000277488
317	6	0.000545996	0.000283010	0.000262449
318	6	-0.000529559	0.000059990	-0.000336187
319	6	0.001316098	0.000031915	0.000088578
320	1	0.000538713	0.000045555	-0.000468426
321	1	-0.000661707	-0.000402083	0.000207470
322	1	0.000068597	-0.000068919	-0.000059184
323	1	-0.000342882	-0.000129909	-0.000048523
324	1	-0.000463085	0.000026036	0.000217010
325	1	-0.000375645	0.000051255	-0.000134501
326	8	0.000417534	0.000602799	-0.000557867
327	8	-0.001908858	-0.000780529	-0.002545136
328	6	-0.000611024	-0.000103195	0.000468244
329	6	-0.000654490	-0.000205333	-0.001259934
330	6	0.000670361	0.001051887	0.001679815
331	6	0.000244125	-0.000065719	-0.000549085
332	6	-0.000019974	0.000202144	0.000304482
333	6	0.000764199	-0.000025110	-0.000103098
334	6	-0.000574935	-0.000268615	0.000177637
335	6	0.001205477	0.000426919	0.000203088
336	1	0.000493582	0.000123841	0.000636897
337	1	-0.000683780	0.000033988	-0.000450062
338	1	0.000004660	0.000109546	0.000113060
339	1	-0.000332158	-0.000158728	0.000056948
340	1	-0.000345000	-0.000011843	-0.000043504
341	1	-0.000335046	-0.000245326	-0.000270288
342	8	0.000570243	0.000755265	-0.002041070
343	8	-0.000615344	0.001089593	0.000381680
344	6	-0.000203558	-0.000238624	0.001643504
345	6	-0.000076149	0.000024002	-0.000613509
346	6	-0.000252089	-0.001028066	0.000278208
347	6	0.000850694	0.000597427	0.000152185
348	6	0.000033483	0.000024146	0.000450981
349	6	0.000435904	0.000137165	-0.000457637
350	6	-0.000478886	0.000067386	-0.000302572
351	6	0.001176223	0.000573178	0.000315234
352	1	0.000047055	0.000126554	0.000068175

353	1	-0.000611839	-0.000437098	-0.000328930
354	1	0.000067907	-0.000077025	-0.000144761
355	1	-0.000332874	-0.000067629	-0.000023217
356	1	-0.000346272	-0.000142676	-0.000175859
357	1	-0.000319690	-0.000392603	-0.000020377
358	8	0.000250969	-0.000574346	0.001585505
359	8	-0.000477467	-0.000928102	-0.000662756
360	6	-0.000083441	0.000146772	-0.000996386
361	6	-0.000334826	0.000062870	0.000357890
362	6	-0.000224171	0.000746295	-0.000120952
363	6	0.000836891	-0.000220075	-0.000050441
364	6	0.000152683	-0.000083620	-0.000278615
365	6	0.000538059	-0.000007539	0.000318995
366	6	-0.000494055	-0.000222587	0.000309731
367	6	0.001361173	-0.000136153	-0.000058156
368	1	0.000225924	-0.000072995	-0.000043190
369	1	-0.000899671	0.000369128	0.000151046
370	1	-0.000050315	0.000064640	0.000160664
371	1	-0.000446212	0.000236971	-0.000069434
372	1	-0.000333975	-0.000059188	-0.000055167
373	1	-0.000461387	-0.000009435	0.000106081
374	1	-0.000174936	0.000510386	0.000727980
375	1	-0.001163737	-0.000987839	0.000117649
376	1	-0.000062202	0.000164576	0.000114674
377	1	-0.002594679	0.001333460	-0.000787551
378	1	0.000363814	-0.000573974	0.000131165
379	1	0.000614248	0.001048122	-0.000111236
380	1	-0.000568987	0.000040604	0.000285641
381	1	0.000177690	0.001157117	0.000034326
382	1	-0.000508768	-0.000180361	0.000462134
383	1	-0.001838904	0.001852646	0.001176351
384	8	0.000804532	-0.000447534	-0.000597815
385	8	0.000637051	0.000378001	-0.000238456
386	6	-0.000304843	0.000033582	0.000139710
387	6	0.000847403	0.000213846	-0.000230628
388	6	0.000036422	0.000472850	0.000444382
389	6	-0.000456928	-0.000563887	-0.000243790
390	6	-0.000245518	-0.000065286	0.000484281
391	6	-0.000121648	-0.000068031	-0.000359220
392	6	0.000544895	-0.000212038	0.000121455
393	6	-0.001281658	-0.000522247	-0.000429166
394	1	-0.000298089	-0.000401824	-0.000044180
395	1	0.000549164	0.000561993	0.000052660
396	1	-0.000010620	0.000114972	-0.000038286
397	1	0.000436769	0.000052112	0.000137013
398	1	0.000385394	0.000146792	-0.000004359
399	1	0.000345299	0.000384534	0.000244302
400	8	0.000285824	-0.000111138	-0.000989131
401	8	0.002904040	-0.001693106	-0.000653763
402	6	0.000273427	-0.000098983	0.001551095
403	6	0.001636063	-0.000467831	-0.000543682
404	6	-0.002000209	0.001061590	0.001044671
405	6	0.000630552	-0.000747053	-0.000488072
406	6	-0.000272835	0.000390830	0.000886670
407	6	-0.000730909	0.000152084	-0.000948894
408	6	0.000449696	0.000222839	0.000185329
409	6	-0.001234605	-0.000037821	-0.000302558
410	1	-0.000822547	0.000297364	0.000131139

411	1	0.000700754	-0.000234922	0.000261448
412	1	-0.000122590	-0.000009697	0.000112653
413	1	0.000363862	0.000118547	0.000071757
414	1	0.000348829	0.000026147	0.000213287
415	1	0.000423561	-0.000196826	-0.000041206
416	8	-0.000114686	0.000561336	-0.000267877
417	8	0.000290563	-0.000774160	-0.001101248
418	6	0.000349913	-0.000476649	0.000272428
419	6	0.000204036	0.000137654	-0.000859989
420	6	0.000990134	-0.000117774	0.000241341
421	6	-0.000122029	-0.000380972	-0.000911443
422	6	0.000359667	-0.000078416	-0.000452203
423	6	-0.000580930	-0.000213156	-0.000456477
424	6	0.000372086	0.000732968	0.000184802
425	6	-0.001207230	-0.000486994	-0.000648571
426	1	-0.000079857	0.000021944	0.000126324
427	1	0.000651024	0.000270190	0.000550622
428	1	0.000022101	0.000116340	0.000093992
429	1	0.000404473	0.000212797	0.000163370
430	1	0.000349980	-0.000021668	0.000426799
431	1	0.000324207	0.000061449	0.000035664
432	8	-0.000000703	-0.001954177	-0.000393542
433	8	0.000176102	-0.000032673	-0.000061228
434	6	-0.000329440	0.001132743	0.000364665
435	6	0.000169132	-0.000558928	-0.000491359
436	6	0.000542945	0.000468266	-0.000350608
437	6	-0.000981639	-0.000591176	0.000009996
438	6	-0.000272577	0.000565234	-0.000137895
439	6	-0.000038069	-0.000478519	-0.000137518
440	6	0.000328207	0.000334556	0.000475213
441	6	-0.001188266	-0.000738387	-0.000321562
442	1	-0.000098546	0.000055696	0.000359195
443	1	0.000782517	0.000129022	-0.000206562
444	1	-0.000002007	-0.000029581	0.000011244
445	1	0.000404548	0.000349118	-0.000109931
446	1	0.000342292	0.000207597	0.000234390
447	1	0.000381281	0.000087868	0.000089204
448	1	-0.000056203	0.000743531	0.000939141
449	1	-0.000705896	-0.000872446	0.000323457
450	1	-0.000208868	-0.000262470	-0.000063320
451	1	0.000104537	-0.000077744	-0.000102374
452	1	-0.000860028	0.000470540	0.000995117
453	1	-0.000640637	-0.002249322	-0.001540441
454	1	-0.000682186	-0.000565027	0.000636270
455	1	0.000191686	0.000134921	-0.000006698
456	1	-0.000011633	0.000872117	0.000041475
457	1	0.000236880	0.000193380	0.000175195
458	6	0.001336331	0.003114953	0.000481262
459	6	-0.001143075	0.000379709	-0.000443715
460	6	-0.000975004	0.000023873	0.001295875
461	6	-0.000143056	-0.000208638	0.000439944
462	6	-0.000386668	0.000022150	-0.000405801
463	6	0.000842971	-0.000437354	0.000469516
464	1	-0.002650487	0.004676898	-0.003206732
465	1	-0.003665248	-0.001764937	-0.004668469
466	1	-0.000902111	-0.005934583	-0.001334848
467	1	0.002890408	-0.004275045	0.003667918
468	1	0.003004443	0.002397371	0.004977490

469	17	0.001406662	0.000467895	0.000747564
470	1	-0.000214682	0.000271298	0.000143263
471	1	-0.000513986	0.000444391	0.000588089
472	1	-0.000165083	-0.000153876	0.000016404
473	8	-0.001894378	0.001569403	0.000470059
474	1	0.000801775	-0.000421824	-0.000476071
475	1	0.000815535	-0.000851884	0.000181369
476	8	0.002018518	-0.000758191	-0.001273934
477	1	-0.000724276	-0.000096416	0.000518249
478	1	-0.001184099	0.000669944	0.000311426
479	8	-0.001104240	-0.001736069	-0.001033173
480	1	0.000440503	0.000424560	0.000778091
481	1	0.000390647	0.000981918	0.000039185
482	6	-0.004678538	0.000252537	0.002748583
483	8	0.000211062	0.001840343	-0.003507622

Sum of electronic and thermal Free Energies= -1284.405101
1 imaginary frequency: -169.72

α-I

Center Number	Atomic Number	Integrated Forces (Hartrees/Bohr) X	Y	Z
1	6	-0.005418502	-0.000151448	0.004895800
2	6	0.001595488	-0.001555158	0.002168051
3	7	-0.003843058	-0.004864845	-0.002082688
4	6	0.006234942	0.000856150	-0.003774308
5	6	-0.000233466	0.007008193	-0.001231885
6	6	-0.006560844	0.001601042	0.000282561
7	1	0.003388418	-0.002140234	-0.001374408
8	1	-0.011456916	-0.002371607	0.002876416
9	1	0.000535308	-0.002105414	0.000875622
10	1	-0.000071775	-0.000504882	0.000527983
11	1	0.000569553	-0.000743852	0.000062490
12	1	0.001810283	-0.001126171	0.000571343
13	1	0.000716130	-0.000340587	-0.000382209
14	8	0.002279315	-0.000335487	0.002419753
15	8	-0.003201821	-0.011138450	0.000450978
16	6	-0.001974725	0.000652690	-0.003337309
17	6	0.001042305	-0.001760641	0.002375367
18	6	-0.002120379	0.007958748	-0.000555715
19	6	-0.000050801	-0.003839397	-0.001154507
20	6	-0.001574391	0.002059086	-0.001252952
21	6	0.001418238	-0.000293765	0.002393140
22	6	0.000722773	0.000340529	-0.000267063
23	6	-0.000678648	-0.000529336	0.000035912
24	1	0.000006836	-0.000197019	-0.000989055
25	1	0.000077014	0.000153004	0.000080557
26	1	0.000334727	0.000081900	0.000016419
27	1	0.000061728	0.000136531	0.000001194
28	1	0.000208532	-0.000001972	0.000065242
29	1	0.000138906	0.000055434	-0.000109815
30	8	0.000636475	0.000239727	-0.000555871
31	8	0.001251122	-0.000119411	0.000004771
32	6	-0.000177409	-0.000119312	0.000333856
33	6	0.000595297	0.000210718	-0.000120006

34	6	-0.001267139	0.000070490	0.000171243
35	6	0.000897320	-0.000039946	-0.000167627
36	6	-0.000336051	0.000433927	-0.000232343
37	6	0.000148577	-0.000610373	-0.000124081
38	6	0.000061097	0.000087597	-0.000129622
39	6	0.000131249	-0.000187585	0.000014640
40	1	-0.000234318	-0.000197894	0.000069381
41	1	-0.000105348	0.000088919	0.000079784
42	1	-0.000034630	0.000022677	0.000012426
43	1	-0.000047209	0.000066114	0.000005433
44	1	-0.000041538	0.000043900	-0.000006460
45	1	-0.000033196	0.000055532	0.000019830
46	8	-0.003447454	-0.002805242	-0.006818477
47	8	0.001456676	0.000618845	-0.002724488
48	6	0.001591014	0.006962493	0.008012926
49	6	-0.001032505	0.000453778	-0.005251701
50	6	-0.001275188	-0.000217958	0.003747336
51	6	0.000574095	0.000102729	-0.001584576
52	6	-0.000723994	0.000308213	0.001760634
53	6	-0.000656557	-0.003270141	-0.001432738
54	6	-0.000637143	0.000825035	0.000546534
55	6	0.000180586	-0.000560451	-0.000551190
56	1	0.001020416	-0.001332854	0.001099899
57	1	0.000101050	0.000278903	0.000012349
58	1	0.000015802	-0.000040820	0.000132982
59	1	0.000086865	0.000091537	0.000173318
60	1	-0.000048682	0.000156253	0.000097363
61	1	-0.000079576	0.000051160	0.000098294
62	8	0.000837136	-0.000096327	-0.000151009
63	8	0.000666616	0.001460144	0.001383617
64	6	-0.000861924	0.000004219	-0.000012194
65	6	0.000328328	0.000051112	0.000552875
66	6	-0.000434842	-0.000435100	-0.001920934
67	6	0.000560372	-0.000230158	0.000257369
68	6	-0.000050166	0.000058734	0.000108214
69	6	0.000522823	-0.000025705	0.000109469
70	6	-0.000573322	0.000614986	0.000338215
71	6	0.000287121	-0.000562706	-0.000013639
72	1	-0.000225608	0.000013905	-0.000218665
73	1	-0.000108144	0.000078071	-0.000013461
74	1	-0.000023708	0.000031026	-0.000112637
75	1	-0.000084493	0.000070289	-0.000020470
76	1	-0.000059073	0.000144288	0.000021621
77	1	0.000017491	0.000162043	-0.000051526
78	8	-0.000102093	-0.000151348	0.000063257
79	8	0.000288421	-0.000348442	0.000100732
80	6	0.000142836	0.000180663	-0.000021996
81	6	-0.000152143	-0.000381034	0.000064782
82	6	-0.000175202	0.000363404	-0.000082885
83	6	0.000020583	-0.000141686	0.000039436
84	6	-0.000041249	0.000062708	0.000005255
85	6	-0.000068208	-0.000129529	-0.000044200
86	6	0.000015065	0.000075237	0.000054591
87	6	0.000016515	-0.000134128	-0.000027296
88	1	0.000078412	0.000144188	-0.000042184
89	1	0.000023122	0.000028992	0.000011663
90	1	-0.000033718	0.000044603	-0.000009408
91	1	-0.000007362	0.000037193	-0.000005865

92	1	0.000003530	0.000026180	0.000007389
93	1	-0.000021712	0.000033410	0.000007129
94	8	-0.000006750	-0.000063058	-0.000095965
95	8	-0.000073780	0.000017141	-0.000098013
96	6	0.000025015	0.000044153	0.000126526
97	6	-0.000103546	-0.000041242	-0.000136106
98	6	0.000093382	0.000025311	0.000082161
99	6	-0.000060949	-0.000059920	-0.000062892
100	6	-0.000016943	0.000021035	0.000035321
101	6	-0.000016490	-0.000033940	-0.000081369
102	6	0.000042263	0.000015574	0.000059814
103	6	-0.000078992	-0.000025028	-0.000063788
104	1	0.000043599	0.000007085	0.000049596
105	1	0.000016653	0.000020620	0.000014281
106	1	0.000016490	-0.000010947	0.000007901
107	1	0.000016750	0.000001287	0.000020517
108	1	0.000016026	0.000012799	0.000015461
109	1	0.000027250	-0.000000499	0.000008380
110	8	-0.000179972	0.000057103	-0.000077934
111	8	-0.000269978	-0.000039852	-0.000047619
112	6	0.000169068	0.000000666	0.000069798
113	6	-0.000299012	-0.000003619	-0.000058466
114	6	0.000240147	0.000055944	0.000138216
115	6	-0.000126899	-0.000072688	-0.000055309
116	6	0.000015440	-0.000029504	0.000014378
117	6	-0.000098874	-0.000031478	-0.000034853
118	6	0.000045420	0.000057218	-0.000022899
119	6	-0.000078267	-0.000076474	0.000022580
120	1	0.000156684	0.000022678	0.000021631
121	1	0.000029805	0.000032111	0.000012667
122	1	0.000019823	0.000004245	-0.000017112
123	1	0.000015488	0.000022184	-0.000014267
124	1	0.000027100	0.000008569	-0.000008240
125	1	0.000015013	0.000022018	-0.000000695
126	8	0.000586651	-0.000633059	-0.000009843
127	8	0.000044444	-0.000261050	-0.000053647
128	6	-0.000233720	0.000384203	-0.000137773
129	6	0.000172795	-0.000514409	-0.000149816
130	6	0.000004571	0.000286008	0.000048919
131	6	-0.000030239	-0.000133716	-0.000017701
132	6	-0.000015235	0.000046142	-0.000028002
133	6	0.000087496	-0.000145539	-0.000011431
134	6	0.000009904	0.000062409	0.000018762
135	6	-0.000010510	-0.000087153	-0.000050773
136	1	-0.000104558	0.000264087	0.000108674
137	1	0.000012289	0.000029904	0.000015515
138	1	-0.000006345	0.000015158	0.000017513
139	1	0.000005018	0.000016559	0.000020788
140	1	-0.000009419	0.000026434	0.000012928
141	1	0.000004814	0.000019841	0.000010532
142	8	0.000290188	-0.000498712	0.000229044
143	1	-0.000341980	0.000243654	-0.000320112
144	1	-0.000173928	0.000183713	0.000052159
145	8	0.000575244	-0.000808836	0.000210677
146	1	-0.000413471	0.000733807	-0.000203617
147	1	-0.000507120	0.000268408	0.000002734
148	8	0.010670283	0.010337515	0.026155326
149	1	0.000653407	-0.001950874	-0.005678907

150	1	-0.007208079	-0.006095459	-0.009072711
151	8	0.000412391	0.000454379	0.000062532
152	1	-0.000079968	-0.000109465	-0.000028457
153	1	-0.000119492	-0.000245611	-0.000125956
154	8	-0.000052465	0.000085930	-0.000074334
155	1	0.000056899	-0.000076241	0.000060170
156	1	0.000022607	-0.000009856	0.000016961
157	1	0.015370474	-0.047872924	0.012091237
158	1	-0.000832242	0.000478001	-0.000742062
159	1	-0.000197216	0.000292204	0.000591450
160	1	0.010488321	0.005087507	-0.015983095
161	1	-0.000945472	0.001240104	-0.000244680
162	8	0.001705899	0.000025479	0.001014417
163	8	0.001122072	0.000223265	-0.001226015
164	6	-0.001864126	-0.000681673	-0.001163460
165	6	0.001525047	0.000209467	0.000049630
166	6	-0.001374300	-0.000647133	0.001097033
167	6	0.000393784	0.001087101	-0.000607168
168	6	-0.000266398	-0.000157247	-0.000090683
169	6	0.000804151	0.001122152	0.000323676
170	6	0.000461152	-0.000078831	0.000383642
171	6	-0.000263518	0.000130452	-0.000300857
172	1	-0.000591163	0.000274940	0.000028476
173	1	0.000108921	-0.000328105	0.000074178
174	1	-0.000026137	-0.000058709	0.000059933
175	1	0.000006481	-0.000065353	0.000047585
176	1	0.000077239	-0.000026674	0.000078027
177	1	0.000043811	0.000031153	0.000074835
178	8	-0.009180439	0.014915119	-0.036028950
179	8	0.002046667	-0.001248171	0.001898961
180	6	-0.004237020	0.030096421	0.048291020
181	6	-0.004132335	0.009732884	-0.006945392
182	6	0.001265918	0.000138398	0.000061785
183	6	-0.001901220	0.001190457	-0.003878019
184	6	0.001399202	0.000677439	0.006757225
185	6	-0.000066688	-0.012412172	-0.007192410
186	6	0.000904095	0.000776454	0.000842844
187	6	-0.000418612	-0.000371717	-0.000209484
188	1	0.002011070	-0.006638900	-0.000572043
189	1	0.000345757	-0.000112197	-0.000318770
190	1	-0.000044047	-0.000026443	0.000026560
191	1	-0.000024607	0.000112005	0.000069628
192	1	0.000146339	0.000087449	-0.000009687
193	1	0.000085658	0.000005609	0.000007863
194	8	0.000029381	-0.000015437	-0.000991482
195	8	-0.007179508	-0.007330836	0.005204262
196	6	0.000710241	-0.000627269	0.001256385
197	6	-0.001507392	-0.001308597	-0.000708169
198	6	0.001812880	0.006371878	0.002454937
199	6	-0.000700021	-0.002107402	-0.001306267
200	6	0.001238725	0.001313079	-0.000020857
201	6	-0.001887458	0.000523079	-0.000518136
202	6	0.000843931	0.000469074	-0.001684586
203	6	-0.000226718	-0.000697058	0.000285247
204	1	0.000379036	-0.000064739	0.000359081
205	1	0.000187930	-0.000063481	0.000229229
206	1	-0.000155870	0.000528641	-0.000201222
207	1	0.000151833	0.000106067	0.000015774

208	1	-0.000124739	0.000131355	-0.000031861
209	1	0.000075097	0.000061315	-0.000087610
210	8	-0.000131423	-0.003205621	0.001511187
211	8	0.001237378	0.000345278	0.000628527
212	6	0.001247950	0.002808949	-0.003116156
213	6	-0.000243520	-0.000389458	0.001457807
214	6	-0.001056123	-0.000979610	-0.001586745
215	6	-0.000753609	0.000490814	0.001049572
216	6	0.000928116	0.000254537	-0.000838315
217	6	-0.000323935	-0.001272363	0.001300562
218	6	0.000336274	-0.000260068	-0.000296855
219	6	-0.000138459	0.000312323	0.000220958
220	1	-0.000215982	0.000370052	-0.000404045
221	1	0.000204453	-0.000214288	-0.000175588
222	1	-0.000045411	-0.000056782	-0.000042632
223	1	0.000051250	-0.000085200	-0.000045338
224	1	-0.000010925	-0.000085748	-0.000016666
225	1	0.000022295	-0.000026521	-0.000084839
226	1	-0.000176535	-0.000217965	-0.000202634
227	1	-0.000559243	0.000128066	-0.000266515
228	1	-0.000091614	-0.000154973	-0.000019200
229	1	0.000119190	-0.000098334	-0.000003738
230	1	-0.000156053	0.000050088	-0.000046581
231	1	-0.000025254	-0.000048274	0.000091670
232	1	-0.000088535	-0.000169239	-0.000216540
233	1	0.000064409	-0.000088644	-0.000050371
234	1	0.000073003	-0.000024021	-0.000034647
235	1	-0.000751151	-0.000866341	-0.000861122
236	8	0.000576791	0.000113344	0.000106987
237	8	0.000108820	0.000219020	0.000001789
238	6	-0.000395742	-0.000193226	0.000055247
239	6	0.000225602	0.000161156	0.000062032
240	6	-0.000076735	-0.000177729	0.000027257
241	6	0.000032589	0.000105666	0.000004403
242	6	-0.000021674	-0.000050631	0.000051197
243	6	0.000144195	0.000081034	0.000048308
244	6	-0.000042227	-0.000094695	-0.000004687
245	6	0.000024310	0.000094842	0.000032782
246	1	-0.000132362	-0.000039776	-0.000047302
247	1	-0.000009764	-0.000018377	-0.000015010
248	1	-0.000000419	-0.000010981	-0.000014442
249	1	0.000003639	-0.000021385	-0.000015222
250	1	-0.000013905	-0.000019966	-0.000010721
251	1	-0.000005409	-0.000022719	-0.000006596
252	8	-0.000102799	0.000129904	-0.000039410
253	8	-0.000086616	0.000074394	-0.000073882
254	6	0.000087234	-0.000117292	0.000057957
255	6	-0.000162828	0.000093598	-0.000078814
256	6	0.000105680	-0.000085142	0.000043820
257	6	-0.000057725	0.000065090	-0.000019261
258	6	0.000003887	0.000003824	0.000021957
259	6	-0.000057055	0.000074016	-0.000010122
260	6	0.000003530	-0.000059705	0.000033600
261	6	-0.000029198	0.000077772	-0.000034272
262	1	0.000072428	-0.000043570	0.000028233
263	1	0.000015674	-0.000030714	-0.000004786
264	1	0.000005797	-0.000006270	0.000013091
265	1	0.000000444	-0.000020087	0.000012222

266	1	0.000012897	-0.000013165	0.000012364
267	1	0.000006015	-0.000020844	0.000002695
268	8	0.000000023	0.000101891	-0.000092722
269	8	0.000124623	0.000367880	0.000150744
270	6	0.000101486	-0.000119285	0.000070294
271	6	-0.000116377	0.000283869	-0.000121909
272	6	-0.000072683	-0.000233659	0.000006446
273	6	0.000056186	0.000097738	0.000006713
274	6	-0.000061304	-0.000055336	-0.000037925
275	6	-0.000059122	0.000128143	0.000038632
276	6	0.000018393	-0.000050073	-0.000074878
277	6	0.000019998	0.000091955	0.000051672
278	1	0.000071825	-0.000121745	0.000076807
279	1	0.000015509	-0.000026465	-0.000005741
280	1	-0.000025521	-0.000023905	-0.000005925
281	1	-0.000008810	-0.000029250	-0.000002869
282	1	-0.000002686	-0.000019830	-0.000012854
283	1	-0.000015899	-0.000020088	-0.000014908
284	8	0.000006506	0.000122689	0.000119973
285	8	-0.000088795	0.000178324	0.000086661
286	6	0.000017755	-0.000089494	-0.000147417
287	6	-0.000144884	0.000168077	0.000101879
288	6	0.000085210	-0.000245756	-0.000061190
289	6	-0.000052456	0.000164772	0.000014208
290	6	-0.000041062	-0.000042701	-0.000072135
291	6	0.000013169	0.000043075	0.000076478
292	6	0.000049980	-0.000013030	-0.000022760
293	6	-0.000065188	0.000040766	0.000019741
294	1	0.000086350	-0.000073864	-0.000023901
295	1	0.000011905	-0.000027799	-0.000000149
296	1	0.000014148	0.000000151	-0.000001244
297	1	0.000015769	-0.000007375	-0.000010535
298	1	0.000012255	-0.000014924	-0.000005101
299	1	0.000019514	-0.000006357	0.000001466
300	1	0.000047538	-0.000108428	-0.000103659
301	1	-0.000036814	-0.000272206	0.000053871
302	1	-0.000102080	-0.000052470	0.000087295
303	1	-0.000103486	0.000280578	-0.000177600
304	1	0.000062010	-0.000041020	0.000048573
305	1	-0.000011470	0.000172798	-0.000148859
306	1	0.000113331	0.000005986	-0.000093342
307	1	-0.000207283	-0.000075828	-0.000099295
308	1	-0.000617886	-0.000237460	0.000369062
309	1	0.004301493	0.001998530	-0.007013579
310	8	0.000030229	0.000155542	0.000260116
311	8	0.000306456	0.000463444	0.000020978
312	6	-0.000047880	-0.000077336	-0.000376923
313	6	-0.000015965	0.000274618	0.000316188
314	6	-0.000253598	-0.000469651	-0.000106319
315	6	0.000111206	0.000210525	0.000030303
316	6	-0.000156418	-0.000102117	-0.000060049
317	6	0.000073611	0.000010990	0.000282797
318	6	-0.000128598	0.000055547	-0.000096023
319	6	0.000127020	0.000024548	0.000091473
320	1	0.000040365	-0.000089512	-0.000098998
321	1	-0.000004524	-0.000001220	-0.000045339
322	1	-0.000018419	-0.000032104	-0.000016299
323	1	-0.000022003	-0.000019251	-0.000025095

324	1	-0.000029254	-0.000002827	-0.000021069
325	1	-0.000039692	-0.000012155	-0.000008142
326	8	0.001129955	0.000107849	-0.000705892
327	8	0.000233486	-0.000170198	0.000386416
328	6	-0.001292381	0.000050153	0.000394656
329	6	0.000620098	-0.000414615	0.000354916
330	6	-0.000251664	0.000091245	-0.000521464
331	6	0.000153218	0.000057206	0.000369607
332	6	-0.000328689	-0.000102740	-0.000061406
333	6	0.000444196	-0.000118137	-0.000215940
334	6	-0.000080419	0.000095703	-0.000072473
335	6	0.000050392	-0.000084494	0.000098802
336	1	-0.000262144	0.000219168	-0.000116755
337	1	-0.000008424	0.000018283	-0.000042193
338	1	0.000004703	0.000022233	-0.000008471
339	1	-0.000015818	0.000014421	-0.000025656
340	1	0.000004657	0.000021909	-0.000023769
341	1	-0.000018778	0.000023747	-0.000011459
342	8	-0.000153404	-0.000139653	0.000260985
343	8	-0.000148723	0.000035235	0.000143556
344	6	0.000152274	0.000055589	-0.000323137
345	6	-0.000281377	-0.000106447	0.000290349
346	6	0.000187390	-0.000002823	-0.000181255
347	6	-0.000069382	-0.000007619	0.000134246
348	6	0.000049895	0.000001220	-0.000003671
349	6	-0.000023227	-0.000052119	0.000210509
350	6	0.000020307	-0.000072442	-0.000074357
351	6	-0.000026033	0.000049560	0.000109683
352	1	0.000108596	0.000040543	-0.000112252
353	1	-0.000006829	0.000005008	-0.000059936
354	1	0.000018710	-0.000009365	-0.000012546
355	1	0.000004518	-0.000020850	-0.000019781
356	1	0.000017278	-0.000004019	-0.000024177
357	1	-0.000002150	-0.000008428	-0.000029204
358	8	0.000436069	0.000444483	0.000088017
359	8	0.000696590	-0.000157962	-0.000373177
360	6	-0.000334216	-0.000400228	-0.000136456
361	6	0.000585839	0.000149254	0.000207594
362	6	-0.000656384	0.000257354	0.000078935
363	6	0.000284742	0.000018930	0.000023044
364	6	-0.000052904	0.000045589	-0.000040227
365	6	0.000222405	0.000078879	0.000098813
366	6	-0.000023765	-0.000059169	-0.000087540
367	6	0.000098637	0.000022747	0.000064944
368	1	-0.000313055	-0.000133500	0.000003706
369	1	-0.000040170	-0.000011385	-0.000026548
370	1	-0.000034105	0.000006952	0.000006080
371	1	-0.000024491	-0.000006714	-0.000017657
372	1	-0.000025095	0.000007097	-0.000013144
373	1	-0.000029493	-0.000011874	-0.000007799
374	1	0.000293664	0.000434518	0.000315539
375	1	-0.000902799	-0.006108372	0.008120837
376	1	-0.000621533	-0.000345170	0.000273919
377	1	-0.000018809	-0.000030723	0.000117050
378	1	0.001230592	-0.002010772	-0.000869088
379	1	0.000083198	-0.000014430	0.000100391
380	1	0.000123805	0.000076808	-0.000067835
381	1	0.000065238	-0.000086883	0.000075628

382	1	0.000104133	0.000511477	-0.000188057
383	1	-0.000663497	-0.000332174	0.000153744
384	8	-0.000106534	-0.001398530	-0.000450468
385	8	-0.004880808	0.004358164	-0.011501590
386	6	0.000473277	0.001379458	0.000773135
387	6	-0.000541230	0.000425686	-0.002438925
388	6	0.002112872	0.002004363	0.001234968
389	6	-0.000681999	-0.000013299	-0.001003575
390	6	0.000087146	0.000408381	-0.000037844
391	6	-0.000142003	-0.000249211	-0.000268367
392	6	0.000168500	-0.000678623	-0.000032318
393	6	-0.000025151	0.000471672	-0.000100990
394	1	0.000060553	-0.000785339	0.002647069
395	1	0.000033522	-0.000123752	0.000123560
396	1	0.000005125	-0.000120401	0.000036619
397	1	0.000015254	-0.000095714	0.000028147
398	1	0.000005440	-0.000092371	-0.000022429
399	1	-0.000051982	-0.000088484	0.000069499
400	8	0.000075175	0.000078802	-0.000088229
401	8	0.000088396	0.000153952	-0.000113721
402	6	0.000004727	-0.000038423	-0.000000047
403	6	0.000028175	0.000082578	-0.000094746
404	6	-0.000135100	-0.000137340	0.000114355
405	6	0.000108671	0.000110793	-0.000110850
406	6	-0.000054303	-0.000130227	-0.000041292
407	6	0.000016528	0.000106620	0.000095705
408	6	0.000036063	-0.000087185	0.000042150
409	6	-0.000044370	0.000064016	-0.000076503
410	1	-0.000002687	-0.000017115	0.000055956
411	1	0.000005676	-0.000024328	0.000006965
412	1	0.000010164	0.000005090	0.000012338
413	1	0.000013649	-0.000002225	0.000027558
414	1	0.000012178	-0.000015854	0.000012229
415	1	0.000011530	-0.000021493	0.000019290
416	8	0.000172295	-0.000215622	0.001551976
417	8	0.000223745	-0.000011421	-0.000211613
418	6	-0.000884836	0.000889299	-0.002148300
419	6	0.000229152	-0.000665434	0.000478320
420	6	-0.000317605	0.000215678	-0.000009414
421	6	0.000104910	-0.000011486	0.000081859
422	6	-0.000141036	0.000092225	-0.000035716
423	6	0.000087940	0.000340072	0.000592517
424	6	-0.000147591	-0.000039924	-0.000052266
425	6	0.000065500	0.000085208	0.000021972
426	1	0.000001450	-0.000054580	0.000194513
427	1	0.000019394	-0.000066801	-0.000028682
428	1	-0.000006561	-0.000006150	0.000003124
429	1	-0.000005382	-0.000030557	-0.000003032
430	1	-0.000010515	-0.000022163	0.000010003
431	1	-0.000011121	-0.000020472	-0.000011456
432	8	-0.000182243	0.000028321	-0.000108018
433	8	-0.000128198	-0.000499041	-0.000010541
434	6	0.000174959	0.000084747	0.000049058
435	6	-0.000055000	-0.000117616	-0.000171819
436	6	-0.000089115	0.000209449	0.000126638
437	6	-0.000104261	0.000027586	-0.000235849
438	6	0.000162256	-0.000074676	0.000048786
439	6	-0.000183682	-0.000038324	0.000004404

440	6	0.000211807	0.000144180	0.000221277
441	6	-0.000077250	-0.000122300	-0.000266719
442	1	0.000009133	0.000060550	0.000067532
443	1	0.000015974	-0.000022438	0.000036801
444	1	-0.000019375	0.000071632	0.000012177
445	1	0.000019316	0.000039437	0.000035078
446	1	0.000027367	0.000000012	0.000064581
447	1	-0.000025305	0.000039615	0.000071060
448	1	0.000017065	-0.000071906	0.000061791
449	1	0.003887066	0.001155304	0.001525364
450	1	-0.000593898	0.000567863	0.000770025
451	1	-0.001015092	-0.000189974	0.000200648
452	1	-0.000087117	-0.000131621	0.000174360
453	1	-0.000126613	0.000204607	-0.000108847
454	1	-0.000154535	0.000249301	0.000052994
455	1	0.000029726	0.000036061	0.000052433
456	1	-0.000117826	-0.000076851	0.000055458
457	1	-0.000012844	0.000103714	0.000046063
458	6	-0.004550249	0.004053374	-0.001153005
459	6	-0.004646212	-0.000260003	-0.001656939
460	6	0.002823294	-0.002229547	0.000703637
461	6	-0.001629196	-0.002310675	0.000873747
462	6	0.000220478	0.002219398	-0.001038859
463	6	0.004361766	-0.002035235	-0.000969298
464	1	-0.000175315	0.001161455	-0.000672708
465	1	0.000247237	0.000536004	-0.000231065
466	1	-0.000087959	0.000553035	-0.000082932
467	1	-0.000179252	-0.000066536	0.000019649
468	1	-0.000328404	-0.000386617	0.000490656
469	17	-0.017977097	0.002093011	-0.023048602
470	1	-0.001186583	0.000358561	0.000138832
471	1	-0.000030849	-0.000355585	0.000585784
472	1	0.000087299	0.000093597	-0.000074865
473	8	-0.000211453	0.000058097	0.000066970
474	1	0.000104227	-0.000049212	-0.000007967
475	1	0.000152447	-0.000037426	-0.000047876
476	8	0.000357552	0.000419835	-0.000025046
477	1	-0.000356893	-0.000271899	-0.000145539
478	1	-0.000162767	-0.000245769	0.000046549
479	8	0.000213630	0.001183793	-0.000087310
480	1	0.000307557	-0.000806520	-0.000646774
481	1	-0.000196030	-0.001138354	0.000409139
482	6	0.007964720	-0.000238341	0.009496331
483	8	0.018511811	0.012720587	0.011378087

Sum of electronic and thermal Free Energies= -1284.412436
0 imaginary frequency

α-TS2

Center Number	Atomic Number	Integrated Forces (Hartrees/Bohr) X	Y	Z
1	6	-0.005952622	0.000109395	-0.002438229
2	6	0.007312334	-0.001434350	0.000807288
3	7	0.000158210	0.007938035	0.001206073
4	6	0.002294185	-0.000492543	0.003404329

5	6	-0.001405760	-0.003756852	0.000577977
6	6	-0.003913490	-0.005256522	-0.001486834
7	1	-0.000964510	-0.006713444	-0.000685000
8	1	0.005271665	-0.001775180	-0.007631667
9	1	-0.000494930	0.008662557	-0.003028242
10	1	-0.004936627	-0.000240326	-0.005500561
11	1	-0.000032193	0.006204834	-0.003901048
12	1	0.004787326	-0.003250695	-0.000312767
13	1	-0.001840092	0.001228483	0.006859518
14	8	-0.001505286	-0.000759456	0.002206177
15	8	0.003265787	0.002055020	-0.004210663
16	6	0.001005119	0.000704901	-0.001982039
17	6	-0.001472455	-0.000829546	0.001557318
18	6	-0.001434648	0.000816436	0.001291185
19	6	0.000403498	-0.002335446	-0.001101167
20	6	0.000410355	0.001559624	-0.000720680
21	6	-0.000609248	-0.000868046	0.001431808
22	6	-0.000944168	0.000476797	-0.000757072
23	6	0.001267727	-0.001385510	-0.000447672
24	1	0.000651795	-0.000163209	-0.000637086
25	1	-0.000211243	0.000383445	0.000612940
26	1	-0.000161441	0.000247145	0.000075125
27	1	-0.000149579	0.000577043	0.000269421
28	1	-0.000498628	0.000323079	0.000154425
29	1	-0.000324650	0.000206820	0.000155758
30	8	-0.001072736	0.000258550	-0.000124141
31	8	-0.004375890	-0.000679561	0.000663145
32	6	0.000173389	0.000279221	-0.000318620
33	6	-0.001557765	-0.000249453	0.000281223
34	6	0.003088692	0.001263793	-0.000618122
35	6	-0.001268096	-0.000709328	-0.000033958
36	6	0.000524070	0.000832405	-0.000284708
37	6	0.000360995	-0.001192685	0.000041311
38	6	-0.000281968	0.000411549	0.000046147
39	6	0.000919861	-0.000786858	-0.000278894
40	1	0.000681988	-0.000083640	0.000032627
41	1	-0.000636641	0.000364496	-0.000146018
42	1	0.000127988	0.000075229	-0.000140545
43	1	-0.000424925	0.000107149	0.000035614
44	1	-0.000193198	0.000217132	0.000219623
45	1	-0.000292594	0.000353443	0.000044514
46	8	-0.003352791	0.000545612	-0.007243002
47	8	-0.002862189	-0.000068945	-0.002967737
48	6	0.002664096	0.006879905	0.009445172
49	6	-0.001228621	-0.000590089	-0.004318988
50	6	0.002824617	-0.000139682	0.002935450
51	6	-0.000964519	-0.000035935	-0.001517063
52	6	0.001331505	-0.000053179	0.001877507
53	6	0.000459726	-0.004510216	-0.002721709
54	6	0.000390096	0.001117367	0.000994012
55	6	0.000735349	-0.001402223	-0.000910258
56	1	0.000440196	-0.000507783	0.000712738
57	1	-0.000492550	0.001044395	0.000102495
58	1	-0.000001563	-0.000137783	0.000065968
59	1	-0.000283518	0.000344127	0.000415508
60	1	-0.000252048	0.000557123	0.000028523
61	1	-0.000273878	0.000203328	0.000177819
62	8	-0.001801601	-0.000969717	-0.001256834

63	8	-0.000118714	0.000436720	0.000571670
64	6	0.001710152	0.000609007	0.000433882
65	6	-0.000914382	-0.000366700	0.000457773
66	6	0.000091821	0.000104146	-0.000429890
67	6	0.000234178	-0.000245369	-0.000418576
68	6	0.000385432	0.000092389	0.000254145
69	6	-0.000386285	-0.000342092	-0.000086796
70	6	0.000077548	0.000727308	0.000409110
71	6	0.000564138	-0.000907951	-0.001025111
72	1	0.000664085	0.000203080	-0.000571434
73	1	-0.000579925	-0.000004026	0.000534365
74	1	0.000074925	0.000045126	-0.000005280
75	1	-0.000244937	0.000169068	0.000205624
76	1	-0.000144080	0.000201782	0.000467919
77	1	-0.000203134	0.000372813	0.000265604
78	8	0.000220194	0.000289461	0.000203612
79	8	0.002772776	0.000957499	-0.001724343
80	6	-0.000286873	-0.000496119	-0.000698993
81	6	0.000868951	0.000532682	-0.000469597
82	6	-0.001933363	-0.001143415	0.000472751
83	6	0.000255842	0.000551511	-0.000070356
84	6	-0.000187885	-0.000403822	0.000085462
85	6	-0.000376256	0.000297087	0.000558627
86	6	0.000252937	-0.000376157	-0.000186166
87	6	-0.000807519	0.000445971	0.000778877
88	1	-0.000264616	-0.000334758	0.000309146
89	1	0.000354552	-0.000082952	-0.000698069
90	1	-0.000172320	-0.000045922	-0.000058905
91	1	0.000151604	-0.000196109	-0.000312451
92	1	0.000403278	0.000124795	-0.000328063
93	1	0.000268739	-0.000172775	-0.000122792
94	8	-0.000376018	-0.000059978	-0.000638089
95	8	0.001076626	-0.002618875	-0.000328305
96	6	0.000791611	0.000239449	0.000694330
97	6	0.000162931	-0.001023181	-0.000256887
98	6	-0.000014070	0.001783675	0.000552419
99	6	-0.000292362	-0.000465417	0.000041805
100	6	0.000012756	0.000431703	0.000115294
101	6	-0.000591392	0.000312548	-0.000120828
102	6	0.000422084	-0.000004609	-0.000225047
103	6	-0.001036299	0.000682231	0.000247628
104	1	-0.000151364	0.000568678	0.000033214
105	1	0.000566039	-0.000484511	0.000073437
106	1	0.000022761	0.000070092	0.000141771
107	1	0.000407506	-0.000129772	-0.000011204
108	1	0.000260919	-0.000432832	-0.000022297
109	1	0.000257881	-0.000153370	-0.000170679
110	8	-0.000419131	-0.001151542	0.001046458
111	8	-0.000662171	0.000079916	0.000349900
112	6	0.000408226	0.000796101	-0.000551310
113	6	-0.000508437	-0.000757316	-0.000012221
114	6	0.000626903	0.000100821	-0.000759697
115	6	-0.000537683	0.000337222	0.000553138
116	6	-0.000080430	0.000181956	-0.000007036
117	6	-0.000403050	0.000168001	0.000307886
118	6	0.000473240	-0.000213236	-0.000024249
119	6	-0.000840492	0.000419116	0.000913294
120	1	0.000264945	0.000312924	0.000210560

121	1	0.000413837	-0.000597579	-0.000451166
122	1	0.000009660	0.000094223	-0.000034476
123	1	0.000285382	-0.000034594	-0.000251361
124	1	0.000258047	-0.000160289	-0.000198465
125	1	0.000095299	-0.000171919	-0.000442301
126	8	0.000244096	0.002152421	-0.001363193
127	8	-0.000460640	0.000387802	0.000139838
128	6	-0.000480985	-0.001379889	0.001024102
129	6	0.000253526	0.000797855	-0.000208498
130	6	0.000755317	-0.000689093	0.000199422
131	6	-0.000873173	0.000447199	0.000037493
132	6	-0.000170295	-0.000305880	0.000314984
133	6	-0.000107936	0.000587138	-0.000073783
134	6	0.000164359	-0.000288140	-0.000233222
135	6	-0.001123337	0.000743881	0.000027604
136	1	-0.000236969	-0.000272613	-0.000149584
137	1	0.000831323	-0.000068003	-0.000177169
138	1	-0.000012839	-0.000100523	0.000157768
139	1	0.000293696	-0.000328903	-0.000063201
140	1	0.000337798	-0.000155364	-0.000103023
141	1	0.000470830	-0.000225237	0.000160469
142	8	0.000041573	-0.001169275	-0.002169272
143	1	-0.000484247	0.000218505	0.001114357
144	1	0.000478427	0.000837316	0.000670577
145	8	0.000745092	0.000492178	0.000048802
146	1	0.000394333	0.000836198	0.000014667
147	1	0.000685583	-0.000630137	0.000435604
148	8	0.000535460	-0.014726768	0.005527556
149	1	-0.001028192	0.007084589	0.001732964
150	1	0.003260810	0.007739314	-0.008633233
151	8	-0.000917171	-0.000139936	0.000534538
152	1	-0.000042790	0.000000560	0.000981921
153	1	-0.000389722	-0.000888761	-0.000275901
154	8	0.000996192	0.007938383	0.006530924
155	1	0.000385568	-0.003774608	-0.005416425
156	1	-0.001736608	-0.005608919	-0.002527702
157	1	0.004313855	0.002878956	0.004665974
158	1	0.000486652	-0.000885129	-0.001610464
159	1	0.000151599	-0.000169577	-0.000482680
160	1	0.009304602	-0.002867970	0.005917909
161	1	-0.000223968	-0.000514565	0.000102706
162	8	0.000462009	0.000066654	-0.000988962
163	8	-0.001457516	-0.000570414	-0.001770211
164	6	-0.001144897	0.000015964	0.001484466
165	6	-0.000269183	-0.000789932	-0.001074706
166	6	0.001209410	0.001262098	0.002014051
167	6	-0.000647333	-0.000757550	-0.000488054
168	6	-0.000039277	0.000051796	0.000932659
169	6	0.001008680	0.000258847	-0.000733359
170	6	0.000274193	-0.000357156	-0.000410830
171	6	0.000313647	0.000837231	0.000558596
172	1	0.000258149	0.000046924	0.000329999
173	1	-0.000546731	-0.000137387	-0.000046084
174	1	0.000169298	-0.000019820	-0.000098730
175	1	-0.000144192	-0.000182633	0.000036378
176	1	-0.000263579	-0.000185680	-0.000315931
177	1	-0.000049560	-0.000409017	-0.000192905
178	8	-0.006131267	0.001992495	0.004984984

179	8	-0.000836464	0.000880763	0.001953370
180	6	0.001926748	-0.013137260	-0.017952265
181	6	-0.001114971	0.002939629	0.004987021
182	6	0.003213477	-0.001475541	-0.001579218
183	6	-0.001549908	0.001082480	0.001348919
184	6	0.001153474	0.001115613	-0.002103837
185	6	-0.001368705	0.005666287	0.003287427
186	6	0.000504457	-0.000688087	-0.001275506
187	6	-0.000225817	0.001689345	0.000486718
188	1	0.001149117	-0.000569568	-0.000601661
189	1	0.000488259	-0.001097749	0.000116438
190	1	-0.000076028	0.000035415	-0.000032986
191	1	-0.000068581	-0.000520554	-0.000266260
192	1	0.000234855	-0.000610104	0.000082076
193	1	-0.000061867	-0.000315580	0.000011250
194	8	-0.001444657	-0.000015071	0.000970062
195	8	-0.007387614	0.005670460	0.000276662
196	6	0.001443093	0.001212916	-0.001294527
197	6	-0.002224277	0.000099791	0.002178600
198	6	0.003252167	-0.005555590	-0.004095511
199	6	-0.001114465	0.002402438	0.002299453
200	6	0.001787248	-0.001005823	-0.000300787
201	6	-0.001837659	-0.000702890	0.000536558
202	6	0.001090734	-0.000337282	0.001453976
203	6	-0.000633290	0.002004706	-0.000202860
204	1	0.000380788	0.000285839	-0.000583767
205	1	-0.000136761	-0.000436013	-0.000602846
206	1	0.000363395	-0.000465152	-0.000061813
207	1	0.000331179	-0.000599514	-0.000146439
208	1	-0.000021647	-0.000693508	0.000157161
209	1	-0.000011603	-0.000406819	-0.000042929
210	8	-0.001175212	0.002219641	-0.000802303
211	8	-0.000537590	0.000662545	-0.000346108
212	6	0.001868432	-0.002328335	0.002220826
213	6	-0.000699671	0.000291660	-0.001148819
214	6	0.000052947	-0.000012994	0.000696668
215	6	-0.000735538	0.000092434	-0.000534605
216	6	0.001023087	-0.000407565	0.000168445
217	6	-0.000680754	0.001539736	-0.001143179
218	6	0.000316264	-0.000486910	0.000272167
219	6	0.000461636	0.000847649	-0.000419529
220	1	0.000230982	-0.000115125	0.000363393
221	1	-0.000179058	-0.000560794	0.000578108
222	1	-0.000055030	0.000066299	-0.000026759
223	1	-0.000294951	-0.000221799	0.000253162
224	1	-0.000203305	-0.000198815	-0.000017113
225	1	-0.000076690	-0.000331940	0.000103492
226	1	0.000435107	-0.000727151	0.000080729
227	1	0.000313570	0.000100078	-0.000137114
228	1	-0.000504341	0.000278999	0.000647089
229	1	0.000097054	-0.000316204	-0.000200585
230	1	0.000610731	0.000574055	0.000666071
231	1	0.000119215	-0.000292014	-0.000080749
232	1	-0.000266986	0.000468010	0.000413889
233	1	-0.000267272	-0.000016948	0.000000271
234	1	0.000659683	-0.000120589	-0.000516956
235	1	0.000601531	-0.000727871	-0.001409546
236	8	-0.000665253	0.000280194	0.000095103

237	8	-0.000147284	0.001629957	-0.002270252
238	6	0.001068598	-0.000207288	0.000315956
239	6	-0.000085747	0.000905955	-0.000898415
240	6	0.000237304	-0.001874984	0.002278063
241	6	0.000106395	0.000152671	-0.000769210
242	6	0.000255619	-0.000174741	0.000600778
243	6	-0.000807909	-0.000410030	-0.000042566
244	6	0.000026218	0.000532750	0.000162217
245	6	-0.000373001	-0.001161456	0.000214992
246	1	-0.000082948	-0.000322715	0.000319383
247	1	0.000584320	0.000436958	-0.000417260
248	1	-0.000149974	0.000039684	0.000074520
249	1	0.000049377	0.000406707	-0.000014930
250	1	0.000173116	0.000235613	-0.000003193
251	1	0.000138973	0.000370934	-0.000310436
252	8	0.000128648	0.000403152	0.000070171
253	8	0.001133661	0.000644486	0.002534991
254	6	-0.000355565	0.000359516	-0.000160450
255	6	0.000587988	0.000472833	0.000941789
256	6	-0.001515802	-0.000506050	-0.002233643
257	6	-0.000002185	0.000060005	0.000683981
258	6	-0.000237774	0.000114902	-0.000476813
259	6	-0.000042841	-0.000765337	-0.000027176
260	6	0.000480156	0.000232637	-0.000170265
261	6	-0.000901682	-0.000846135	-0.000282295
262	1	-0.000204403	-0.000270042	-0.000397737
263	1	0.000107609	0.000759058	0.000370540
264	1	0.000114971	-0.000135920	-0.000054543
265	1	0.000362393	0.000246721	0.000018811
266	1	0.000150675	0.000301211	0.000017704
267	1	0.000280630	0.000250610	0.000297501
268	8	-0.000506904	0.001057832	0.000481315
269	8	0.000237830	-0.000066211	-0.000660867
270	6	0.000326011	-0.000316847	-0.000247651
271	6	-0.000027569	0.000286676	0.000079070
272	6	0.000106602	0.000178380	0.000341744
273	6	-0.000405814	-0.000481497	-0.000283461
274	6	-0.000194907	-0.000085363	-0.000112124
275	6	-0.000511750	-0.000306547	-0.000073286
276	6	0.000072347	0.000447295	0.000241853
277	6	-0.000889576	-0.000858554	-0.000139121
278	1	-0.000103553	-0.000126395	-0.000020865
279	1	0.000678400	0.000452583	0.000429490
280	1	-0.000006580	0.000031342	-0.000078271
281	1	0.000206077	0.000273354	0.000127358
282	1	0.000477804	0.000150597	0.000050304
283	1	0.000201032	0.000253250	-0.000064516
284	8	0.001138779	0.000132685	-0.000466181
285	8	-0.000508655	0.000374664	0.000884818
286	6	-0.000445826	0.000065158	0.000457431
287	6	0.000037870	0.000061026	-0.000231055
288	6	0.000338411	0.000066962	-0.000295508
289	6	-0.000225947	-0.000589332	0.000187648
290	6	0.000016101	-0.000211865	0.000180848
291	6	-0.000102630	-0.000504806	-0.000123629
292	6	0.000369879	0.000268870	-0.000165966
293	6	-0.000389734	-0.001183751	0.000044662
294	1	0.000085168	-0.000083695	0.000065761

295	1	0.000070377	0.000812468	-0.000266253
296	1	-0.000013118	-0.000024605	0.000110900
297	1	0.000175426	0.000298285	-0.000117904
298	1	-0.000064873	0.000470677	-0.000022305
299	1	0.000163675	0.000321286	0.000102575
300	1	0.000289369	0.000408959	0.000198584
301	1	-0.000513657	0.002141745	-0.001838786
302	1	0.000529721	-0.001062094	-0.000457226
303	1	-0.001178769	-0.001230087	-0.002702111
304	1	-0.001730152	0.002051545	-0.000078042
305	1	-0.000027122	-0.001438084	0.000164234
306	1	-0.001061997	0.000244105	-0.000427682
307	1	0.000449276	0.000893633	0.000575006
308	1	0.002488216	0.000599614	-0.000701315
309	1	-0.002014674	-0.000424063	0.003013330
310	8	-0.000457529	-0.000412389	-0.000336318
311	8	0.001292959	-0.001942985	0.001112485
312	6	-0.000068089	0.000158143	0.000335441
313	6	0.000754644	-0.001014797	0.000606524
314	6	-0.001194788	0.000926219	-0.000285208
315	6	0.000115247	-0.000207469	-0.000303376
316	6	-0.000110077	0.000216015	0.000060081
317	6	-0.000050201	0.000134110	-0.000455434
318	6	0.000251971	0.000137689	0.000497137
319	6	-0.000647880	0.000440890	-0.001006085
320	1	-0.000305586	0.000600282	-0.000275381
321	1	0.000073961	-0.000528835	0.000450937
322	1	-0.000130201	0.000020080	-0.000015220
323	1	0.000043176	-0.000163253	0.000323242
324	1	0.000302501	-0.000344645	0.000245035
325	1	0.000216819	-0.000003420	0.000306118
326	8	0.000051014	0.000877746	0.000422078
327	8	-0.000157352	0.001365838	0.002878232
328	6	-0.000000192	-0.000463758	-0.000112340
329	6	0.000065793	0.000587142	0.001428033
330	6	0.000676081	-0.000737089	-0.001626798
331	6	-0.000231642	0.000469346	0.000161099
332	6	0.000195603	-0.000171281	-0.000144410
333	6	-0.000273281	0.000305937	-0.000222676
334	6	0.000028907	-0.000441062	0.000413549
335	6	-0.000231795	0.000338385	-0.001169713
336	1	-0.000062233	-0.000313827	-0.000791488
337	1	0.000257964	0.000157679	0.000653930
338	1	0.000129741	-0.000019685	-0.000107060
339	1	-0.000057330	0.000086846	0.000510155
340	1	0.000061450	-0.000222819	0.000324675
341	1	0.000211380	-0.000051269	0.000293027
342	8	-0.000218124	0.002391341	0.000315871
343	8	0.001126180	0.000091257	-0.000019383
344	6	0.000198795	-0.001522377	-0.000545652
345	6	-0.000090683	0.000534473	0.000153691
346	6	-0.000798939	-0.000805093	0.000432519
347	6	0.000239078	0.000443640	-0.000942995
348	6	-0.000007932	-0.000448622	-0.000376565
349	6	-0.000106951	0.000555888	-0.000106318
350	6	0.000194411	0.000104781	0.000386145
351	6	-0.000029028	0.000411898	-0.001252538
352	1	0.000132530	-0.000041126	-0.000011145

353	1	-0.000166185	-0.000135464	0.000751080
354	1	-0.000199205	0.000079762	0.000082120
355	1	0.000163691	-0.000147516	0.000311627
356	1	0.000010441	-0.000036069	0.000468243
357	1	-0.000130933	-0.000293902	0.000386328
358	8	-0.000577855	-0.001045732	-0.000756899
359	8	-0.002278839	-0.000415644	0.000704290
360	6	0.000143255	0.000570806	0.000478021
361	6	-0.000190294	-0.000378443	0.000253446
362	6	0.001183217	0.001020429	0.000104577
363	6	-0.000798738	0.000203909	-0.000423584
364	6	-0.000179492	0.000299263	-0.000046691
365	6	-0.000372281	-0.000108972	-0.000487480
366	6	0.000238308	-0.000590352	0.000154301
367	6	-0.000954422	0.000504531	-0.000811301
368	1	0.000076545	-0.000034164	-0.000138944
369	1	0.000775356	-0.000324286	0.000430640
370	1	0.000204121	-0.000109138	-0.000113504
371	1	0.000451791	0.000035176	0.000329909
372	1	0.000149690	-0.000090345	0.000260638
373	1	0.000289101	-0.000241198	0.000268784
374	1	-0.000000728	-0.000591370	-0.000390085
375	1	-0.000295060	0.001289358	-0.001087737
376	1	0.000993704	-0.000377529	0.000282827
377	1	0.002050882	0.000248012	0.001902875
378	1	-0.000403723	0.000670987	0.000086454
379	1	0.000192804	0.000611209	-0.000767201
380	1	0.000681677	-0.000346129	0.000209331
381	1	0.000821160	0.001013682	-0.000516310
382	1	0.000348187	0.000183468	0.001515268
383	1	0.003419123	-0.000792600	-0.000082025
384	8	-0.000640446	0.001561833	0.000759736
385	8	0.000462747	-0.001679074	0.000916560
386	6	0.000522916	-0.001236148	-0.000542681
387	6	-0.000038253	-0.000172266	0.001275332
388	6	-0.000081573	0.000543857	0.000290544
389	6	0.000053597	-0.000373540	0.000654731
390	6	0.000193304	-0.000308296	0.000122969
391	6	0.000020062	0.000033067	0.000555934
392	6	-0.000317708	0.000318899	-0.000249383
393	6	0.000380631	-0.000411154	0.001160332
394	1	-0.000026010	0.000699275	-0.001334247
395	1	0.000036282	0.000500370	-0.000787235
396	1	-0.000033846	0.000155209	0.000083593
397	1	0.000130117	0.000082613	-0.000493442
398	1	-0.000217216	0.000001703	-0.000285401
399	1	-0.000166213	0.000216889	-0.000350097
400	8	-0.000255617	-0.000422344	0.000377611
401	8	-0.002787206	0.000625243	-0.001218290
402	6	0.000310299	-0.000009330	-0.000447780
403	6	-0.001421569	0.000297858	-0.000403360
404	6	0.002080136	-0.000890269	0.000308432
405	6	-0.000544874	0.000001815	0.000152634
406	6	0.000423173	-0.000097531	-0.000232014
407	6	0.000062246	0.000116262	0.000626202
408	6	-0.000045622	0.000456279	-0.000424865
409	6	0.000537696	-0.000577264	0.000957919
410	1	0.000730649	-0.000121012	0.000336870

411	1	-0.000516960	-0.000061078	-0.000449887
412	1	0.000132312	-0.000048460	-0.000044588
413	1	-0.000057112	0.000255489	-0.000324886
414	1	-0.000115721	0.000044873	-0.000298625
415	1	-0.000367281	0.000160544	-0.000221439
416	8	0.000860586	0.000761822	-0.001370889
417	8	-0.001001644	0.001199780	-0.000051163
418	6	-0.001305036	-0.001344069	0.002314940
419	6	-0.000032339	0.000647985	-0.000802829
420	6	-0.000100726	-0.000884984	-0.000083936
421	6	-0.000003298	0.000238670	0.000567708
422	6	-0.000213262	-0.000283123	0.000374904
423	6	0.000417689	-0.000059515	-0.000029543
424	6	0.000188523	0.000170793	-0.000473897
425	6	0.000152161	0.000110211	0.001332445
426	1	0.000308388	-0.000177728	0.000463693
427	1	-0.000091798	-0.000186935	-0.000774365
428	1	0.000179176	-0.000084967	-0.000093659
429	1	-0.000137474	-0.000207655	-0.000430242
430	1	-0.000142200	0.000038784	-0.000294447
431	1	0.000045793	-0.000000680	-0.000509918
432	8	-0.002040841	-0.001306198	0.000428669
433	8	-0.000156223	-0.000699299	-0.001004326
434	6	0.001810947	0.000582876	0.000017430
435	6	-0.000838523	-0.000199405	-0.000411637
436	6	0.000237766	0.001267314	0.000006749
437	6	0.000447280	-0.000937830	0.000523547
438	6	0.000745957	0.000301415	-0.000194806
439	6	-0.000760197	-0.000273756	0.000407365
440	6	-0.000157421	-0.000101159	-0.000444374
441	6	0.000608213	-0.000614016	0.001046823
442	1	0.000230890	-0.000173293	0.000256491
443	1	-0.000499574	0.000549866	-0.000378109
444	1	-0.000138486	0.000035313	0.000124887
445	1	-0.000343150	0.000147375	-0.000292267
446	1	-0.000155871	0.000383723	-0.000255645
447	1	-0.000116525	0.000099509	-0.000390511
448	1	0.000661343	-0.000306739	-0.000763884
449	1	-0.002374093	0.001524063	-0.000149635
450	1	0.000842977	0.000879615	0.001576556
451	1	0.000294122	0.000271897	-0.000151012
452	1	0.000960445	-0.001292522	0.000433057
453	1	-0.002119763	-0.000010795	0.002228581
454	1	0.000112385	-0.001073027	0.000534523
455	1	0.000174794	0.000020552	0.000168614
456	1	0.000552802	0.000431934	-0.000330805
457	1	0.000061524	-0.000085921	-0.000344502
458	6	0.001353031	0.002724073	-0.002651990
459	6	0.000583773	-0.000022754	0.000173535
460	6	-0.001281142	0.001406223	-0.001277840
461	6	-0.000213875	0.000339641	-0.000257112
462	6	0.000145575	-0.000073656	-0.000142356
463	6	0.000187404	0.000003527	-0.000591802
464	1	0.006287752	0.002548851	0.001521648
465	1	0.005562907	-0.004250688	0.002937427
466	1	-0.001849892	-0.006310250	0.001969300
467	1	-0.006353351	-0.001999178	-0.000506062
468	1	-0.004902642	0.004201332	-0.002389287

469	17	-0.005668977	-0.002510186	0.004269776
470	1	0.000006476	-0.000311070	0.000055580
471	1	0.000784585	-0.000455352	-0.000279418
472	1	0.000068226	-0.000332540	0.000209704
473	8	0.002266145	-0.000222563	0.000727783
474	1	-0.001073322	0.000354425	-0.000220384
475	1	-0.000794335	-0.000283671	-0.000419958
476	8	-0.001640047	0.002146066	-0.000623950
477	1	0.000454411	-0.001171505	0.000136520
478	1	0.001034508	-0.001049553	0.000431272
479	8	-0.002954746	-0.000908963	0.001599917
480	1	0.001171005	0.000152284	-0.001141923
481	1	0.001336881	0.000036917	-0.000316306
482	6	-0.005278192	-0.003192516	0.008560656
483	8	0.005296349	0.002537095	0.000438189

Sum of electronic and thermal Free Energies= -1284.404437
1 imaginary frequency: -517.58

***α*-4a**

Center Number	Atomic Number	Integrated Forces (Hartrees/Bohr) X	Y	Z
1	6	-0.000571077	0.000768660	-0.000533392
2	6	0.000926239	-0.000269380	0.001851427
3	7	0.002244219	-0.001757661	0.002481567
4	6	-0.000132288	-0.000561433	-0.000010549
5	6	-0.000272976	0.000183464	-0.000459506
6	6	-0.003031461	-0.000376899	-0.005229264
7	1	-0.002747143	-0.003059822	-0.005056427
8	1	-0.005589359	0.003386786	0.002153444
9	1	0.001430146	0.005498115	0.004087788
10	1	-0.003784345	0.004667547	-0.002558006
11	1	-0.000379942	0.005610385	0.003894278
12	1	0.005442973	-0.000880485	-0.002248339
13	1	-0.001325977	-0.005183153	0.003204415
14	8	-0.000274621	-0.000025408	0.000012604
15	8	0.002498622	0.006776652	0.002773113
16	6	-0.000022078	-0.000280017	0.000078302
17	6	0.000194435	-0.000139075	0.000310620
18	6	-0.002697006	-0.003345551	-0.002831571
19	6	0.000380451	-0.000081552	-0.000252257
20	6	-0.000077140	0.000152289	-0.000315802
21	6	0.000172973	0.000041835	-0.000523892
22	6	-0.000495711	0.000150112	0.000205491
23	6	0.000790460	-0.000248945	-0.001087341
24	1	-0.000109852	0.000144528	-0.000144749
25	1	-0.000212913	-0.000276937	0.000729455
26	1	-0.000076006	0.000011300	-0.000089967
27	1	-0.000078466	0.000085448	0.000542220
28	1	-0.000295544	0.000175297	0.000244896
29	1	-0.000285393	-0.000028640	0.000252885
30	8	-0.000251224	-0.000178797	0.000342556
31	8	-0.003442115	0.000070689	0.000437223
32	6	0.000168013	0.000519673	0.000249004
33	6	-0.001309450	-0.000069460	-0.000026282

34	6	0.002439114	0.000509785	0.000310610
35	6	-0.000680531	-0.000094135	-0.000464717
36	6	0.000523303	0.000166697	0.000314518
37	6	0.000388317	-0.000362984	-0.000712215
38	6	-0.000220879	0.000048177	0.000485435
39	6	0.000903778	-0.000278926	-0.000857601
40	1	0.000521810	-0.000139127	0.000020644
41	1	-0.000584850	0.000356763	0.000271549
42	1	0.000151186	0.000095457	0.000050301
43	1	-0.000443258	0.000082451	0.000130850
44	1	-0.000209378	-0.000001311	0.000298982
45	1	-0.000197161	0.000187775	0.000285502
46	8	0.000135541	-0.001101430	0.000640054
47	8	-0.000935611	0.000525723	-0.000552607
48	6	-0.000065977	0.000386481	-0.000212606
49	6	-0.000442533	0.000034639	0.000302667
50	6	0.000181191	-0.000205741	0.000242807
51	6	0.000265042	-0.000017364	-0.000522189
52	6	-0.000018518	-0.000300565	-0.000096728
53	6	0.000438751	-0.000223645	-0.000652191
54	6	-0.000102190	-0.000142662	0.000565882
55	6	0.000719459	-0.000400329	-0.001075744
56	1	0.000284763	-0.000002265	-0.000293739
57	1	-0.000215266	0.000385925	0.000739650
58	1	-0.000038604	-0.000003870	-0.000040218
59	1	-0.000167162	0.000030889	0.000391381
60	1	-0.000217950	0.000389751	0.000346696
61	1	-0.000315717	0.000074388	0.000207310
62	8	-0.001477943	0.000886247	-0.001571880
63	8	0.000096976	-0.000145878	-0.000358754
64	6	0.001332691	-0.000391781	0.000884610
65	6	-0.000712732	-0.000191638	-0.000539445
66	6	-0.000078689	-0.000098234	0.000654672
67	6	0.000485855	0.000309794	-0.000696182
68	6	0.000397201	-0.000174557	0.000210843
69	6	-0.000191656	0.000077171	-0.000618568
70	6	-0.000136902	0.000239712	0.000515957
71	6	0.000701971	0.000208789	-0.001235053
72	1	0.000337716	0.000279825	0.000131087
73	1	-0.000737264	-0.000195445	0.000436069
74	1	0.000113083	-0.000024762	0.000086240
75	1	-0.000288299	-0.000007349	0.000260538
76	1	-0.000252623	-0.000294687	0.000387922
77	1	-0.000132085	-0.000020194	0.000437053
78	8	0.000339387	-0.000203333	0.000144105
79	8	0.002902192	0.001394929	-0.000715109
80	6	-0.000464909	0.000509797	-0.000609675
81	6	0.001101318	0.000433347	0.000036556
82	6	-0.002168599	-0.000655349	-0.000360544
83	6	0.000381808	0.000313447	0.000328425
84	6	-0.000319229	-0.000189177	-0.000123599
85	6	-0.000262969	-0.000282202	0.000617344
86	6	0.000184278	-0.000103173	-0.000466299
87	6	-0.000792756	-0.000250607	0.000998145
88	1	-0.000414190	-0.000370618	-0.000034309
89	1	0.000382484	0.000301728	-0.000522272
90	1	-0.000161122	0.000051567	-0.000024543
91	1	0.000142761	0.000093128	-0.000405492

92	1	0.000357161	0.000224391	-0.000214981
93	1	0.000242225	-0.000038588	-0.000262647
94	8	-0.000337950	0.000396321	-0.000322878
95	8	0.000540970	-0.001526101	-0.002775457
96	6	0.000709748	-0.000635223	0.000527610
97	6	-0.000060984	-0.000360638	-0.001068773
98	6	0.000452265	0.000664930	0.002040049
99	6	-0.000457979	-0.000263838	-0.000310089
100	6	0.000166249	0.000114757	0.000360761
101	6	-0.000527330	0.000461136	0.000298920
102	6	0.000488543	0.000010051	-0.000157861
103	6	-0.000989123	0.000425646	0.000768311
104	1	0.000083981	0.000324222	0.000351334
105	1	0.000450515	-0.000491239	-0.000450620
106	1	0.000027201	-0.000047050	0.000161797
107	1	0.000383452	-0.000142009	-0.000143487
108	1	0.000206869	-0.000299081	-0.000327173
109	1	0.000259156	-0.000013983	-0.000245655
110	8	-0.000932861	-0.001547435	-0.000326542
111	8	-0.000547979	-0.000023966	0.000239115
112	6	0.000630268	0.001009998	0.000371428
113	6	-0.000558312	-0.000388929	-0.000353765
114	6	0.000671393	0.000405135	-0.000489378
115	6	-0.000586602	-0.000234541	0.000559545
116	6	0.000015368	0.000236906	0.000222720
117	6	-0.000359296	-0.000113875	0.000194654
118	6	0.000438397	-0.000088380	-0.000167493
119	6	-0.000893440	-0.000432629	0.000960586
120	1	0.000222715	-0.000006229	0.000224806
121	1	0.000312129	-0.000000656	-0.000778760
122	1	0.000023385	0.000131520	0.000058094
123	1	0.000370326	0.000153726	-0.000217602
124	1	0.000248479	0.000035086	-0.000291554
125	1	0.000164079	0.000293557	-0.000383941
126	8	0.000565623	0.001772538	0.000761119
127	8	-0.000311245	0.000306294	0.000626914
128	6	-0.000606663	-0.001207127	-0.000504212
129	6	0.000456669	0.000554365	0.000496566
130	6	0.000603518	-0.000747850	-0.000607660
131	6	-0.000743468	0.000366868	0.000478498
132	6	-0.000234110	-0.000297097	-0.000001478
133	6	-0.000114330	0.000286320	0.000415732
134	6	0.000125809	0.000016296	-0.000471077
135	6	-0.000887954	0.000604184	0.000895068
136	1	-0.000330129	0.000009754	-0.000215417
137	1	0.000820301	0.000026882	-0.000293536
138	1	-0.000056237	-0.000136409	-0.000031559
139	1	0.000197751	-0.000189001	-0.000357846
140	1	0.000283085	-0.000081191	-0.000244944
141	1	0.000372778	-0.000320599	-0.000155190
142	8	0.000040287	0.001143586	-0.002120424
143	1	-0.000366321	-0.000635792	0.000856179
144	1	0.000398908	-0.000212281	0.000929943
145	8	0.000792766	0.000115112	0.000070427
146	1	0.000616915	0.000156479	0.000602609
147	1	0.000541371	-0.000816169	-0.000314685
148	8	-0.000536245	-0.005377192	-0.006742219
149	1	0.001882356	0.002611244	0.004512331

150	1	-0.001140788	0.002858970	0.001400004
151	8	-0.000606928	-0.000464074	0.000216355
152	1	-0.000139870	-0.000668976	0.000641376
153	1	-0.000660439	-0.000147827	-0.000639920
154	8	0.002340043	-0.000639032	0.010003184
155	1	-0.000063362	0.001985018	-0.006224603
156	1	-0.002769347	-0.001046107	-0.005736030
157	1	0.000533444	0.000083126	-0.000369077
158	1	-0.000334621	0.000471624	-0.000386077
159	1	0.000287530	0.000092776	-0.000143923
160	1	-0.000253398	-0.000328925	0.000148146
161	1	-0.000387855	-0.000407089	0.000032143
162	8	0.000067477	0.000231105	-0.000360114
163	8	-0.001676625	0.001903487	-0.001334649
164	6	-0.000570936	-0.000554823	0.000597283
165	6	-0.000618750	0.000636051	-0.000796919
166	6	0.001460120	-0.001631076	0.001783143
167	6	-0.000528602	0.000465264	-0.000263805
168	6	0.000155936	-0.000504058	0.000340817
169	6	0.000667375	0.000410781	0.000035032
170	6	-0.000014668	-0.000168666	-0.000523032
171	6	0.000739445	0.000248840	0.000979695
172	1	0.000354162	-0.000266914	0.000195427
173	1	-0.000648966	-0.000127628	-0.000125717
174	1	0.000162737	-0.000012736	-0.000065664
175	1	-0.000260175	-0.000173495	-0.000154383
176	1	-0.000415901	0.000110697	-0.000319628
177	1	-0.000167238	-0.000121653	-0.000403172
178	8	-0.000605090	0.000075062	0.001084413
179	8	0.000078999	-0.000693703	0.000553349
180	6	0.000401485	0.000243213	-0.001295220
181	6	-0.000206397	-0.000243993	0.000121993
182	6	0.000269532	-0.000050937	-0.000361233
183	6	0.000060152	0.000199998	0.000519701
184	6	0.000103926	0.000286718	0.000078324
185	6	-0.000052596	0.000342818	0.000751114
186	6	-0.000352921	0.000011554	-0.000327708
187	6	0.000685329	0.000785846	0.000882350
188	1	-0.000062264	0.000129815	0.000102880
189	1	0.000034636	-0.000521704	-0.000780597
190	1	-0.000084627	0.000006525	0.000074597
191	1	-0.000195948	-0.000094965	-0.000352113
192	1	-0.000086599	-0.000461615	-0.000235971
193	1	-0.000313100	-0.000231484	-0.000174649
194	8	-0.001495139	-0.000028826	0.000376295
195	8	0.000195218	0.000085011	0.000412312
196	6	0.001057463	-0.000040661	-0.000189083
197	6	-0.000496481	0.000010685	0.000330106
198	6	-0.000000011	-0.000084037	-0.001105690
199	6	0.000254394	0.000090628	0.001004182
200	6	0.000423408	-0.000276353	-0.000046132
201	6	-0.000266853	0.000023036	0.000384846
202	6	-0.000249636	-0.000356320	-0.000091257
203	6	0.000524256	0.000554766	0.001098934
204	1	0.000071121	-0.000109209	-0.000024601
205	1	-0.000359338	0.000228870	-0.000742635
206	1	0.000063198	-0.000080481	-0.000047861
207	1	-0.000079897	-0.000106429	-0.000507485

208	1	-0.000154473	-0.000297444	-0.000272218
209	1	-0.000257466	-0.000067397	-0.000266691
210	8	0.000102919	0.000901320	0.000158525
211	8	-0.000695612	0.000620962	0.000711590
212	6	0.000278691	-0.001475761	-0.000188101
213	6	-0.000277469	0.000462542	-0.000022895
214	6	0.000281116	-0.000447157	-0.000210791
215	6	-0.000039847	0.000462004	0.000279550
216	6	0.000506467	-0.000040810	-0.000059536
217	6	0.000317150	0.000980076	0.000370603
218	6	0.000030091	-0.000403896	-0.000382284
219	6	0.000836641	0.000743575	0.000608115
220	1	-0.000027470	-0.000080232	-0.000129809
221	1	-0.000469649	-0.000591930	-0.000113085
222	1	-0.000009958	0.000084585	-0.000004497
223	1	-0.000424232	-0.000342143	-0.000035930
224	1	-0.000290130	-0.000090032	-0.000204825
225	1	-0.000158401	-0.000265602	-0.000259305
226	1	0.000394575	-0.000463762	-0.000783540
227	1	0.000033639	-0.000030191	0.000093510
228	1	-0.000618796	-0.000115606	0.000627960
229	1	0.000085954	0.000064186	-0.000246650
230	1	0.000503494	-0.000515664	0.001013107
231	1	0.000165768	-0.000137806	-0.000225289
232	1	-0.000268912	-0.000249678	0.000731327
233	1	-0.000321901	0.000163239	0.000035625
234	1	0.000817983	0.000283173	-0.000355273
235	1	-0.000083224	0.000182058	-0.000279575
236	8	-0.000091655	0.000207908	0.000187562
237	8	0.000541417	0.002543687	-0.000094408
238	6	0.000568947	-0.000455494	-0.000146636
239	6	0.000349407	0.001165033	0.000180481
240	6	-0.000486120	-0.002756919	-0.000312872
241	6	0.000283702	0.000639486	-0.000263269
242	6	0.000056066	-0.000530292	0.000062227
243	6	-0.000620175	-0.000058779	-0.000200666
244	6	0.000042450	0.000182853	0.000519827
245	6	-0.000617960	-0.000772635	-0.000801593
246	1	-0.000148010	-0.000482727	0.000003760
247	1	0.000646661	0.000339889	0.000115570
248	1	-0.000154830	0.000029428	0.000085922
249	1	0.000139247	0.000254986	0.000351416
250	1	0.000259083	0.000094610	0.000190957
251	1	0.000287190	0.000442196	0.000129245
252	8	0.000230121	-0.000040490	0.000142124
253	8	0.001258772	-0.001735371	0.001832540
254	6	-0.000314822	0.000432956	0.000313682
255	6	0.000704270	-0.000550202	0.000755513
256	6	-0.001567028	0.001690281	-0.001531876
257	6	0.000026509	-0.000413085	0.000442809
258	6	-0.000199820	0.000461102	-0.000169341
259	6	-0.000226526	-0.000354613	-0.000523049
260	6	0.000492739	0.000164934	0.000024185
261	6	-0.001035221	-0.000128480	-0.000762467
262	1	-0.000240038	0.000229334	-0.000391993
263	1	0.000140734	0.000068690	0.000719831
264	1	0.000046552	-0.000054615	-0.000144742
265	1	0.000417500	0.000056748	0.000142900

266	1	0.000191247	0.000141830	0.000262162
267	1	0.000306427	-0.000108653	0.000386957
268	8	-0.000330873	0.000297085	0.001238972
269	8	0.000350980	0.000575243	-0.000753779
270	6	0.000239704	-0.000057688	-0.000659258
271	6	-0.000043674	0.000079207	0.000254919
272	6	0.000128552	-0.000175806	0.000310085
273	6	-0.000474155	0.000101245	-0.000420101
274	6	-0.000250166	-0.000032950	-0.000145576
275	6	-0.000473940	0.000018043	-0.000132850
276	6	0.000160211	-0.000044617	0.000452246
277	6	-0.001065496	-0.000145690	-0.000689150
278	1	-0.000029404	0.000033716	-0.000062607
279	1	0.000687810	-0.000154704	0.000422710
280	1	-0.000011114	0.000039697	-0.000048791
281	1	0.000263088	0.000009679	0.000334404
282	1	0.000518559	0.000001908	0.000116919
283	1	0.000276676	0.000183526	0.000161517
284	8	0.001224675	0.000023077	-0.000283415
285	8	-0.000600646	-0.000406352	0.000604144
286	6	-0.000570114	-0.000068580	0.000300904
287	6	0.000156391	0.000129650	-0.000058024
288	6	0.000322777	0.000219432	-0.000010765
289	6	-0.000316778	-0.000450723	-0.000358694
290	6	-0.000032294	-0.000252827	-0.000040703
291	6	-0.000141070	-0.000272993	-0.000410184
292	6	0.000443518	0.000233665	0.000052179
293	6	-0.000704770	-0.000673113	-0.000823261
294	1	0.000014696	-0.000123218	-0.000012536
295	1	0.000230431	0.000637785	0.000402406
296	1	0.000002472	-0.000086266	0.000011649
297	1	0.000281978	0.000248453	0.000127506
298	1	0.000070652	0.000297655	0.000353184
299	1	0.000233979	0.000080834	0.000283164
300	1	0.000324877	0.000054905	0.000494427
301	1	-0.000116608	0.002830930	0.000642917
302	1	-0.000126622	-0.000490882	-0.000614991
303	1	-0.001071356	0.001796885	-0.002070594
304	1	-0.001414197	0.001656027	0.001969916
305	1	-0.000476871	-0.000988264	-0.000960813
306	1	-0.001044259	0.000823313	0.000034291
307	1	0.000614369	0.000185105	0.001132828
308	1	0.001109990	0.001024134	-0.000416584
309	1	-0.002007695	-0.002362270	0.000979691
310	8	-0.000333240	0.000132436	-0.000619989
311	8	0.001245895	-0.002546641	-0.001351645
312	6	-0.000105669	-0.000403260	0.000502750
313	6	0.000439165	-0.001083447	-0.000714779
314	6	-0.001280863	0.001182155	0.000968118
315	6	0.000146990	0.000032057	-0.000518628
316	6	-0.000225017	0.000104186	0.000200133
317	6	0.000082914	0.000607100	-0.000260227
318	6	0.000236944	-0.000368475	0.000417837
319	6	-0.000521330	0.001149786	-0.000335087
320	1	-0.000191164	0.000701764	0.000325555
321	1	0.000011929	-0.000843746	-0.000195550
322	1	-0.000092907	0.000045882	0.000074696
323	1	0.000028001	-0.000351220	0.000109253

324	1	0.000258661	-0.000402803	-0.000099779
325	1	0.000211082	-0.000273872	0.000203682
326	8	0.000171563	0.000694469	0.000535155
327	8	-0.000102143	-0.001570980	0.002560614
328	6	0.000116166	-0.000635127	-0.000487695
329	6	0.000008261	-0.000528739	0.001318030
330	6	0.000640047	0.000757281	-0.001645844
331	6	-0.000116002	0.000309729	0.000524308
332	6	0.000150984	-0.000056045	-0.000278837
333	6	-0.000264505	0.000595857	0.000040724
334	6	-0.000078170	-0.000648288	-0.000075169
335	6	-0.000013492	0.001233023	-0.000408927
336	1	-0.000047164	0.000370601	-0.000617233
337	1	0.000258452	-0.000447158	0.000500305
338	1	0.000092522	0.000003641	-0.000137533
339	1	-0.000102483	-0.000376418	0.000370530
340	1	-0.000016744	-0.000411268	0.000005093
341	1	0.000117145	-0.000294019	0.000101809
342	8	0.000372991	0.001107887	0.001821659
343	8	0.001178217	-0.000413856	-0.000267855
344	6	-0.000023877	-0.000443776	-0.001321625
345	6	0.000044622	0.000083301	0.000526518
346	6	-0.000900074	-0.000493877	-0.000116035
347	6	0.000340580	0.000916863	-0.000359803
348	6	-0.000037375	0.000015182	-0.000469744
349	6	-0.000003781	0.000460430	0.000208960
350	6	0.000185060	-0.000277717	0.000409242
351	6	0.000123674	0.001255262	-0.000530867
352	1	0.000114144	0.000121538	-0.000070637
353	1	-0.000266233	-0.000672486	0.000436889
354	1	-0.000127643	0.000025270	0.000135677
355	1	0.000082081	-0.000349871	0.000090090
356	1	0.000004496	-0.000389584	0.000290310
357	1	-0.000177013	-0.000426272	0.000076712
358	8	-0.000569050	-0.000382802	-0.001461609
359	8	-0.001295296	-0.000602798	0.000468774
360	6	0.000053999	0.000192080	0.000905543
361	6	0.000280155	-0.000528498	-0.000248257
362	6	0.000848614	0.000171539	0.000509983
363	6	-0.000511840	0.000709287	-0.000109283
364	6	-0.000105493	0.000159428	0.000297809
365	6	-0.000273384	0.000336644	-0.000309429
366	6	0.000065209	-0.000582042	-0.000263081
367	6	-0.000690279	0.001144754	-0.000136410
368	1	-0.000203670	0.000220895	-0.000083977
369	1	0.000590960	-0.000607184	0.000013303
370	1	0.000113711	-0.000037318	-0.000148604
371	1	0.000392472	-0.000314421	0.000156394
372	1	0.000085460	-0.000290615	0.000086290
373	1	0.000205538	-0.000401076	-0.000032860
374	1	-0.000081263	0.000001928	-0.000559459
375	1	-0.000722188	-0.001223401	0.000122668
376	1	0.000171903	0.000022719	-0.000253760
377	1	0.001995238	-0.001921553	0.001170443
378	1	-0.000753318	0.000387417	0.000296965
379	1	0.000506004	0.000847075	0.000050523
380	1	0.000450718	-0.000342229	-0.000087715
381	1	0.001067895	0.000837147	0.000440275

382	1	0.000130216	-0.001035387	0.000867399
383	1	0.002643515	-0.000957382	-0.001074329
384	8	0.000267947	0.000044494	0.000968577
385	8	0.000579789	0.000643620	-0.000679533
386	6	-0.000238296	0.000224056	-0.000624055
387	6	0.000750149	0.000018771	0.000317399
388	6	-0.000221501	0.000067846	0.000239262
389	6	0.000101697	-0.000678842	0.000047160
390	6	-0.000111397	-0.000259899	-0.000118548
391	6	0.000111115	-0.000560664	0.000230898
392	6	-0.000315792	0.000480180	0.000040111
393	6	0.000221743	-0.001288471	0.000422895
394	1	-0.000497201	0.000022326	-0.000066601
395	1	0.000171135	0.000832988	-0.000031525
396	1	0.000001640	0.000016565	0.000138843
397	1	0.000175890	0.000451457	-0.000271661
398	1	-0.000172969	0.000288894	-0.000189424
399	1	-0.000078808	0.000434767	-0.000034663
400	8	-0.000461150	-0.000558148	-0.000310619
401	8	-0.002524575	0.001888571	0.000106937
402	6	0.000450625	0.000390932	0.000064316
403	6	-0.001206086	0.000757391	-0.000056090
404	6	0.001521083	-0.000998929	-0.000720485
405	6	-0.000506598	-0.000262377	0.000210504
406	6	0.000230185	-0.000083882	-0.000177079
407	6	-0.000119529	-0.000467007	0.000226289
408	6	0.000137531	0.000645858	0.000083446
409	6	0.000297354	-0.001262913	0.000093610
410	1	0.000654691	-0.000433161	0.000078387
411	1	-0.000526938	0.000508160	-0.000301458
412	1	0.000095788	-0.000052613	-0.000085099
413	1	0.000035592	0.000432701	-0.000005704
414	1	-0.000091423	0.000297450	-0.000160725
415	1	-0.000273015	0.000364722	0.000104611
416	8	0.001160643	-0.000137914	-0.000154189
417	8	-0.000817359	0.000818772	0.000827764
418	6	-0.000796649	0.000190021	0.000059672
419	6	-0.000023280	0.000334154	-0.000022963
420	6	0.000012605	-0.000396849	-0.000609944
421	6	-0.000030064	-0.000500428	0.000317869
422	6	-0.000008572	0.000068411	0.000174431
423	6	0.000084165	-0.000460489	0.000278037
424	6	0.000279857	0.000549318	-0.000158906
425	6	0.000099798	-0.001109846	0.000759111
426	1	0.000057770	-0.000144615	0.000137638
427	1	0.000040912	0.000574298	-0.000628376
428	1	0.000157463	0.000006086	-0.000068742
429	1	-0.000187158	0.000236582	-0.000406244
430	1	-0.000090569	0.000291909	-0.000096810
431	1	0.000033547	0.000393870	-0.000229401
432	8	-0.002092212	-0.000836382	-0.000289067
433	8	-0.000106821	0.000393706	-0.001028625
434	6	0.001653282	0.000135054	-0.000005122
435	6	-0.000674356	0.000056622	0.000020659
436	6	0.000332414	0.000605537	0.000849873
437	6	0.000139817	-0.001038509	-0.000337634
438	6	0.000665847	0.000029198	0.000102871
439	6	-0.000547223	-0.000293634	0.000031071

440	6	-0.000175706	0.000353191	-0.000337315
441	6	0.000327162	-0.001388114	0.000092367
442	1	0.000103979	-0.000150617	-0.000206861
443	1	-0.000304635	0.000715509	0.000214917
444	1	-0.000113413	-0.000018338	0.000118854
445	1	-0.000249538	0.000414593	-0.000043051
446	1	-0.000012628	0.000492564	0.000133293
447	1	-0.000065278	0.000385088	-0.000151578
448	1	0.000448956	0.000194904	-0.000724332
449	1	-0.000667368	0.000757597	-0.000323754
450	1	0.000443232	-0.000502930	0.000633218
451	1	-0.000129969	0.000458121	0.000283832
452	1	0.000620104	-0.001196316	-0.000695060
453	1	-0.002078331	-0.001491651	0.001597919
454	1	-0.000119621	-0.000974879	-0.000410719
455	1	0.000122313	0.000122888	0.000007355
456	1	0.000592668	0.000484553	0.000091612
457	1	0.000106355	0.000189128	-0.000537843
458	6	0.001394316	0.002491410	0.000949891
459	6	0.001726031	0.000132446	0.000560647
460	6	-0.000313812	-0.000023483	0.000405140
461	6	0.000172661	0.000351357	-0.000053860
462	6	0.000198560	0.000096017	-0.000100818
463	6	-0.000412158	-0.000145108	-0.000079111
464	1	0.006692642	0.001131800	-0.000631207
465	1	0.003593601	-0.003128425	-0.003828198
466	1	-0.002369311	-0.004563216	-0.004030987
467	1	-0.006252694	-0.001251053	0.000187655
468	1	-0.003641976	0.003234042	0.004089321
469	17	0.006178589	-0.004721348	-0.001812380
470	1	0.000096604	-0.000059885	-0.000095737
471	1	0.000659116	-0.000425493	-0.000539902
472	1	-0.000124121	-0.000281186	0.000007718
473	8	0.002104896	-0.001164488	-0.000201512
474	1	-0.000772949	0.000643276	0.000366973
475	1	-0.000984438	0.000228257	-0.000261056
476	8	-0.000902255	0.002038825	0.001091978
477	1	-0.000062613	-0.000939990	-0.000512156
478	1	0.000767292	-0.000811072	-0.000278860
479	8	-0.001825619	-0.001984400	0.000651395
480	1	0.000523141	0.000808628	-0.000664936
481	1	0.000979909	0.000817785	-0.000000330
482	6	-0.001008000	-0.002844943	-0.000833002
483	8	0.004260842	0.000932524	0.003402760

Sum of electronic and thermal Free Energies= -1284.457956
0 imaginary frequency

β-TS1

Center Number	Atomic Number	Integrated Forces (Hartrees/Bohr) X	Y	Z
1	6	0.003371759	-0.004669286	-0.000084446
2	6	0.000530442	0.002992793	0.005743404
3	7	-0.005662945	0.004720239	-0.000289298
4	6	0.001998245	-0.004346188	-0.000551986

5	6	-0.002897614	0.002195667	0.000063167
6	6	0.002308309	-0.003799033	-0.002836742
7	1	-0.000957111	-0.002103028	-0.006437425
8	1	-0.004714146	0.004438820	-0.001985747
9	1	0.003924507	0.000244174	0.006269238
10	1	0.002448294	-0.005487295	-0.000516778
11	1	0.002486553	-0.001575392	0.005298673
12	1	-0.006721659	0.000623761	0.000797072
13	1	0.003007730	0.005281774	-0.002333973
14	8	-0.002680547	-0.000028572	0.001091534
15	8	-0.005492323	-0.004271150	0.003965000
16	6	0.002846867	0.000444032	-0.001676447
17	6	-0.001355684	-0.000852953	0.000168953
18	6	0.002424872	0.004213449	-0.003026114
19	6	-0.000614097	-0.000352965	0.000291129
20	6	0.000192767	0.000750730	-0.000338911
21	6	-0.001340588	0.000653201	0.000793037
22	6	0.000489324	0.000004505	-0.000572109
23	6	-0.000650172	0.000826777	0.000927253
24	1	0.000336589	0.000225074	0.000023959
25	1	0.000570169	-0.000576497	-0.000385761
26	1	0.000101781	0.000099008	-0.000077039
27	1	0.000027889	-0.000203491	-0.000211504
28	1	0.000203412	-0.000192987	-0.000388889
29	1	0.000284380	-0.000415506	-0.000148797
30	8	0.000499058	0.000128496	-0.000116433
31	8	-0.001003384	-0.000529957	-0.002189673
32	6	-0.000863223	-0.000342958	0.000436836
33	6	-0.000028549	-0.000085543	-0.001048290
34	6	-0.000059538	-0.000100365	0.001466928
35	6	0.000044072	0.000232855	-0.000002805
36	6	0.000097709	-0.000009914	0.000175469
37	6	0.000409827	0.000076851	0.000254975
38	6	0.000278963	-0.000405906	-0.000261950
39	6	0.000185262	0.000793820	0.000931865
40	1	0.000217738	-0.000087617	0.000392494
41	1	-0.000499054	-0.000284287	-0.000565684
42	1	-0.000056240	-0.000039282	0.000160762
43	1	-0.000146996	-0.000297005	-0.000168582
44	1	-0.000206595	-0.000095579	-0.000409164
45	1	0.000043424	-0.000259189	-0.000253759
46	8	0.000249316	0.000200375	0.000554217
47	8	0.000455812	0.000387764	0.000239060
48	6	-0.000231663	-0.000072016	-0.000094703
49	6	0.000268007	-0.000075972	-0.000232426
50	6	-0.000853495	-0.000384782	-0.000148187
51	6	0.000291157	0.000512245	0.000215503
52	6	-0.000020831	0.000189143	0.000039482
53	6	-0.000122476	0.000262319	0.000273884
54	6	0.000032023	-0.000097023	-0.000425733
55	6	0.000545839	0.000752800	0.000864475
56	1	0.000059438	-0.000052181	0.000031944
57	1	-0.000114923	-0.000730392	-0.000202670
58	1	-0.000097040	0.000061554	-0.000040619
59	1	-0.000347675	-0.000308925	-0.000174346
60	1	-0.000142162	-0.000154814	-0.000363436
61	1	-0.000069843	-0.000273120	-0.000194671
62	8	-0.001879931	0.001240283	-0.000210252

63	8	-0.000802513	0.000389190	0.000842237
64	6	0.001364656	-0.000677990	0.000381071
65	6	-0.001150938	0.000629299	0.000506804
66	6	0.001511030	-0.000675254	-0.001417637
67	6	-0.000738333	0.000471060	0.001173862
68	6	0.000518435	-0.000029220	0.000323651
69	6	-0.000474024	0.000357072	0.000176929
70	6	-0.000187735	-0.000379585	-0.000028991
71	6	-0.000514005	0.000990026	0.000928099
72	1	0.000256717	-0.000212533	-0.000475416
73	1	0.000157860	-0.000326053	-0.000856224
74	1	0.000238816	-0.000050218	0.000015948
75	1	0.000153261	-0.000433013	-0.000182996
76	1	0.000032691	-0.000257691	-0.000326451
77	1	0.000320268	-0.000235005	-0.000318029
78	8	0.000271766	-0.000068926	0.000204695
79	8	-0.000681037	0.000411523	0.003029763
80	6	-0.000737451	0.000458536	-0.000306723
81	6	-0.000199030	-0.000129557	0.001204004
82	6	-0.000074459	0.000422561	-0.002130758
83	6	-0.000089626	-0.000467944	0.000453806
84	6	-0.000079618	0.000218459	-0.000518479
85	6	0.000443419	-0.000419832	-0.000364892
86	6	0.000009870	0.000495718	0.000198976
87	6	0.000339282	-0.000906556	-0.000894107
88	1	0.000249711	0.000071938	-0.000478397
89	1	-0.000441087	0.000302375	0.000641260
90	1	-0.000089226	0.000052084	-0.000165340
91	1	-0.000188215	0.000142824	0.000456791
92	1	-0.000012337	0.000273725	0.000231482
93	1	-0.000171758	0.000336358	0.000180109
94	8	-0.000389775	0.000157657	-0.000186708
95	8	0.000544421	0.002865353	0.000196818
96	6	0.000808026	-0.000146794	0.000528805
97	6	0.000122943	0.001026094	-0.000333477
98	6	0.000121090	-0.001977079	0.000530804
99	6	0.000030186	0.000402232	-0.000510639
100	6	0.000059689	-0.000519102	0.000238921
101	6	-0.000505518	-0.000418598	-0.000424437
102	6	0.000068786	0.000200632	0.000488950
103	6	-0.000428157	-0.000948896	-0.000854762
104	1	-0.000237456	-0.000474142	0.000129792
105	1	0.000472474	0.000585365	0.000231516
106	1	0.000097163	-0.000141771	0.000045340
107	1	0.000225977	0.000497906	0.000125666
108	1	0.000030922	0.000246053	0.000253791
109	1	0.000193049	0.000199656	0.000343564
110	8	0.001080683	0.000830406	-0.001302427
111	8	-0.000025032	-0.000231594	-0.000494236
112	6	-0.000629579	-0.000786753	0.000950468
113	6	0.000091252	0.000633619	-0.000746189
114	6	-0.000406010	0.000274458	0.000935683
115	6	0.000311434	-0.000685203	-0.000598910
116	6	-0.000194319	-0.000288263	0.000095097
117	6	0.000034918	-0.000066051	-0.000467815
118	6	0.000178313	0.000242074	0.000407323
119	6	0.000417382	-0.000873683	-0.001001856
120	1	0.000103376	-0.000328066	0.000191041

121	1	-0.000185507	0.000802811	0.000290901
122	1	-0.000092116	-0.000076134	0.000065587
123	1	-0.000098910	0.000184144	0.000389954
124	1	-0.000053825	0.000297666	0.000245215
125	1	-0.000310516	0.000341363	0.000278246
126	8	-0.001253970	-0.001320976	0.001010691
127	8	-0.000128902	-0.000662773	-0.000223785
128	6	0.000746232	0.000927382	-0.000898544
129	6	-0.000135125	-0.000725736	0.000595698
130	6	0.000535988	0.000961563	0.000296647
131	6	-0.000351579	-0.000741490	-0.000696982
132	6	0.000189709	0.000167719	-0.000349986
133	6	-0.000117232	-0.000615984	0.000005284
134	6	-0.000157229	0.000452281	0.000188744
135	6	-0.000467472	-0.001073287	-0.000776852
136	1	-0.000157685	0.000245882	-0.000274062
137	1	0.000196389	0.000388298	0.000777923
138	1	0.000081207	0.000043009	-0.000067171
139	1	0.000108944	0.000413424	0.000145937
140	1	0.000064461	0.000259693	0.000268876
141	1	0.000349126	0.000302938	0.000314071
142	8	-0.001635288	0.001620835	0.000407572
143	1	0.000602519	-0.000497506	-0.000441412
144	1	0.000717119	-0.000849062	0.000243423
145	8	0.000038190	-0.000055981	0.000634643
146	1	0.000955042	0.000479456	0.000327059
147	1	0.000113238	-0.000585222	0.000693500
148	8	0.001747221	0.006520244	0.004055971
149	1	-0.002902069	-0.005459629	-0.000650639
150	1	0.000081946	-0.000429051	-0.002920154
151	8	-0.000107902	0.000094146	-0.000221179
152	1	0.000142890	0.000745087	-0.000844942
153	1	0.000886236	-0.000332906	-0.000680205
154	8	0.005634828	-0.008600818	0.000026640
155	1	-0.004697014	0.004067435	0.001954033
156	1	-0.001930497	0.006137459	-0.000753386
157	1	-0.000162128	-0.001195801	0.000201637
158	1	-0.000321847	-0.000056087	-0.000093664
159	1	-0.000003805	0.000008094	0.000339228
160	1	-0.000137952	-0.000459747	-0.000804597
161	1	0.000141328	-0.000136561	-0.000081149
162	8	-0.000178923	0.000047608	-0.000280017
163	8	-0.002658407	0.000536277	-0.001147717
164	6	0.000357904	-0.000039631	-0.000319659
165	6	-0.001061296	0.000255120	-0.000591337
166	6	0.002618975	-0.000879309	0.001006632
167	6	-0.000695273	-0.000150302	-0.000336391
168	6	0.000606041	-0.000052305	0.000013599
169	6	-0.000217276	-0.000366590	0.000626457
170	6	-0.000014029	0.000584268	-0.000080839
171	6	0.000367510	-0.001005706	0.000739427
172	1	0.000439011	-0.000103267	0.000293068
173	1	-0.000196993	0.000244911	-0.000675709
174	1	-0.000028634	0.000083296	0.000149900
175	1	0.000006797	0.000207051	-0.000278700
176	1	-0.000322627	0.000233582	-0.000316298
177	1	-0.000096102	0.000413970	-0.000164769
178	8	-0.000767771	0.001570054	-0.000024921

179	8	0.000329550	0.000035977	-0.000597384
180	6	0.000720532	-0.000790310	-0.000238700
181	6	-0.000289936	0.000658640	-0.000277399
182	6	0.000070206	0.000239722	-0.000024908
183	6	-0.000114770	-0.000554131	0.000557647
184	6	0.000220544	-0.000256695	-0.000131515
185	6	-0.000160221	-0.000054369	0.000436703
186	6	-0.000166849	0.000131448	-0.000474800
187	6	-0.000283992	-0.000862114	0.001047844
188	1	0.000011713	-0.000429051	0.000224490
189	1	0.000110894	0.000794566	-0.000267903
190	1	0.000078559	-0.000077890	-0.000038891
191	1	-0.000019344	0.000307740	-0.000240765
192	1	0.000280985	0.000335411	-0.000278051
193	1	0.000050995	0.000178851	-0.000409676
194	8	-0.000205727	-0.000336756	-0.001408768
195	8	0.000446933	0.001039937	0.000774465
196	6	0.000351917	0.000236245	0.000649664
197	6	-0.000228391	0.000088890	-0.000143407
198	6	-0.000103584	-0.000396938	-0.000532208
199	6	0.000047071	-0.000411195	0.000478557
200	6	0.000093549	-0.000139559	0.000062301
201	6	0.000045663	-0.000404834	0.000076451
202	6	0.000085731	0.000228783	-0.000495992
203	6	0.000163733	-0.001011799	0.000846481
204	1	0.000167494	-0.000116029	0.000002825
205	1	-0.000446065	0.000607661	-0.000348028
206	1	0.000107705	-0.000053944	-0.000050753
207	1	-0.000111949	0.000179485	-0.000326518
208	1	-0.000127014	0.000441923	-0.000133108
209	1	0.000058326	0.000295389	-0.000267376
210	8	-0.000431597	0.000478995	0.000022805
211	8	-0.000701634	-0.000877818	-0.000759888
212	6	0.000463520	0.000090763	-0.000214396
213	6	0.000006168	-0.000078411	-0.000189956
214	6	0.000234392	0.000490526	0.000376708
215	6	-0.000211028	-0.000498550	0.000093714
216	6	-0.000059654	0.000190271	0.000363620
217	6	-0.000363183	-0.000518937	0.000612118
218	6	0.000311743	0.000537006	-0.000143589
219	6	-0.000356445	-0.000817536	0.001012558
220	1	-0.000089462	0.000019036	0.000071947
221	1	0.000414493	0.000371189	-0.000624899
222	1	-0.000057371	0.000018657	0.000047777
223	1	-0.000036061	0.000211738	-0.000277669
224	1	0.000121705	0.000357349	-0.000225519
225	1	0.000218804	0.000097511	-0.000512512
226	1	0.000481627	0.000915635	0.000325758
227	1	0.000488144	-0.000375461	-0.000921175
228	1	0.000405717	-0.000378238	-0.000534369
229	1	-0.000071672	0.000384536	0.000016912
230	1	0.001117070	-0.000856919	0.000006671
231	1	0.000660664	0.000454299	-0.000300986
232	1	0.000929420	-0.000952267	-0.000172238
233	1	0.000022194	-0.000021769	-0.000437721
234	1	-0.000259024	0.000392793	0.000745956
235	1	0.001204787	-0.000334535	0.000131484
236	8	-0.000698825	0.000084586	0.000483506

237	8	-0.002666932	-0.001135087	0.001436821
238	6	0.000558748	0.000200268	0.000527822
239	6	-0.001143864	-0.000427878	0.000784694
240	6	0.002696513	0.001250701	-0.001432325
241	6	-0.000855611	0.000047737	0.000541736
242	6	0.000766416	0.000163643	0.000002663
243	6	-0.000365994	0.000229784	-0.000854061
244	6	0.000066103	-0.000504697	0.000169720
245	6	0.000173913	0.000895322	-0.000865983
246	1	0.000436243	0.000061916	-0.000504665
247	1	-0.000171755	-0.000156552	0.000727559
248	1	-0.000001668	-0.000068107	-0.000133489
249	1	0.000070424	-0.000185323	0.000316896
250	1	-0.000264547	-0.000246577	0.000390775
251	1	-0.000029030	-0.000387212	0.000202595
252	8	-0.000185891	-0.000602927	0.000133798
253	8	0.001953947	-0.001370207	-0.000107982
254	6	-0.000062603	0.000146030	-0.000033255
255	6	0.000765543	-0.000684294	0.000086387
256	6	-0.001852519	0.000802956	-0.000644081
257	6	0.000388741	-0.000228539	-0.000405207
258	6	-0.000088219	0.000177233	0.000017184
259	6	0.000029027	0.000224826	-0.000330166
260	6	0.000019780	0.000194501	0.000746797
261	6	-0.000299433	0.000532438	-0.001264472
262	1	-0.000357614	0.000253286	-0.000026616
263	1	0.000160967	-0.000689418	0.000322505
264	1	0.000069275	0.000202211	0.000098423
265	1	0.000045607	-0.000107398	0.000501775
266	1	-0.000035977	-0.000206216	0.000249744
267	1	0.000256572	-0.000297599	0.000318391
268	8	-0.000906034	-0.001271476	-0.000375810
269	8	-0.000782593	0.000358069	-0.000028071
270	6	0.000486995	0.000614273	0.000499951
271	6	-0.000178314	-0.000599863	-0.000611468
272	6	0.000603355	0.000056288	0.001032426
273	6	-0.000515139	0.000415686	-0.000768084
274	6	-0.000188169	0.000024819	-0.000233970
275	6	-0.000520939	0.000082372	-0.000736669
276	6	0.000444793	-0.000578643	0.000145676
277	6	-0.000459684	0.000749822	-0.001162059
278	1	0.000035810	0.000182880	0.000373936
279	1	0.000459547	-0.000279597	0.000698755
280	1	-0.000045374	-0.000011369	-0.000013736
281	1	-0.000023939	-0.000195461	0.000324275
282	1	0.000128659	-0.000361336	0.000297319
283	1	0.000261740	-0.000042188	0.000454517
284	8	-0.000322432	0.000314182	0.001513901
285	8	0.000537057	-0.000679827	-0.000639981
286	6	0.000326411	-0.000337448	-0.000797989
287	6	-0.000120091	-0.000012569	0.000227390
288	6	-0.000122172	0.000161767	0.000371898
289	6	0.000095324	0.000292618	-0.000410540
290	6	0.000070634	0.000257272	-0.000045247
291	6	0.000037677	0.000411097	-0.000211729
292	6	-0.000083878	-0.000174943	0.000476235
293	6	0.000208870	0.001004820	-0.000830966
294	1	0.000090503	0.000069524	0.000005763

295	1	-0.000411342	-0.000605570	0.000389453
296	1	0.000067611	0.000022893	0.000019953
297	1	-0.000119572	-0.000427238	0.000155751
298	1	0.000054273	-0.000286956	0.000255176
299	1	-0.000096953	-0.000211164	0.000318974
300	1	0.000342488	-0.000563829	0.000299774
301	1	-0.002384100	-0.001791904	0.000613309
302	1	0.000293633	0.000382010	-0.000441616
303	1	-0.002479993	0.001539219	-0.000458917
304	1	-0.001104310	-0.002072360	-0.000839745
305	1	0.000445165	0.001259491	-0.000540437
306	1	-0.000983021	-0.000405839	-0.000951389
307	1	0.000468548	-0.001210330	0.000366814
308	1	-0.001120184	0.000177037	0.000757049
309	1	0.002336838	-0.000071371	-0.002358825
310	8	-0.000619502	0.000611514	-0.000092324
311	8	0.002362069	0.002488518	0.000673053
312	6	0.000697616	-0.000466050	-0.000370730
313	6	0.000792534	0.001221818	0.000256253
314	6	-0.001145688	-0.001679297	-0.001110458
315	6	-0.000140077	0.000599134	0.000217828
316	6	-0.000014563	-0.000335487	-0.000260858
317	6	-0.000772651	0.000110545	0.000258897
318	6	0.000572407	-0.000232625	0.000048255
319	6	-0.001264297	-0.000049505	-0.000057076
320	1	-0.000529577	-0.000583798	-0.000029287
321	1	0.000627443	0.000385453	-0.000107904
322	1	-0.000044464	-0.000103260	-0.000113210
323	1	0.000448004	0.000243532	0.000056916
324	1	0.000357157	-0.000089291	0.000039035
325	1	0.000350276	0.000004818	-0.000102414
326	8	-0.000493353	-0.000461485	0.000316122
327	8	0.002244040	-0.001999046	-0.000653462
328	6	0.000518599	0.000404974	0.000133008
329	6	0.000787505	-0.001097004	-0.000209185
330	6	-0.000952764	0.001299623	0.000959686
331	6	-0.000244790	-0.000463104	-0.000078854
332	6	-0.000009338	0.000230498	0.000156765
333	6	-0.000670012	-0.000139363	-0.000069347
334	6	0.000545419	0.000297615	-0.000156247
335	6	-0.001280626	0.000011469	0.000238018
336	1	-0.000513177	0.000564366	0.000098985
337	1	0.000726768	-0.000328202	0.000137148
338	1	-0.000055514	0.000066268	0.000112832
339	1	0.000355597	-0.000000641	0.000048180
340	1	0.000399689	-0.000210756	-0.000151375
341	1	0.000345905	0.000101001	-0.000104472
342	8	-0.000413113	-0.002002117	0.000443660
343	8	0.000414745	0.000430670	0.001127209
344	6	0.000216763	0.001333645	-0.000056819
345	6	-0.000005605	-0.000547786	-0.000069075
346	6	0.000313879	0.000338207	-0.000975007
347	6	-0.000907196	0.000091302	0.000563095
348	6	-0.000115301	0.000376947	0.000007832
349	6	-0.000509721	-0.000348835	0.000100982
350	6	0.000509003	-0.000355665	0.000082132
351	6	-0.001276814	0.000124075	0.000414983
352	1	-0.000016856	0.000013470	0.000142609

353	1	0.000746961	-0.000202527	-0.000423974
354	1	0.000030552	-0.000147607	-0.000083315
355	1	0.000327484	0.000018308	-0.000007047
356	1	0.000425362	-0.000130600	-0.000099763
357	1	0.000468717	0.000106888	-0.000391808
358	8	-0.000429082	0.002230264	-0.000170512
359	8	0.000443074	-0.000236321	-0.000975353
360	6	0.000040914	-0.001669511	-0.000041900
361	6	0.000141294	0.000707362	0.000220938
362	6	0.000374292	-0.000368275	0.000907909
363	6	-0.000931293	-0.000047660	-0.000454030
364	6	-0.000049021	-0.000427402	-0.000023006
365	6	-0.000407329	0.000518667	0.000071217
366	6	0.000487066	0.000246453	-0.000165153
367	6	-0.001303669	-0.000091703	-0.000283260
368	1	-0.000154878	-0.000118863	-0.000233695
369	1	0.000701250	0.000092515	0.000416705
370	1	0.000008426	0.000139432	0.000090544
371	1	0.000342570	-0.000048543	0.000220055
372	1	0.000342751	-0.000005437	-0.000028527
373	1	0.000413112	0.000122147	0.000065355
374	1	-0.000352080	0.000587161	-0.000149753
375	1	0.001471948	0.000120517	-0.000731724
376	1	0.000035820	-0.000214390	-0.000115672
377	1	0.002418760	-0.000251006	0.001481148
378	1	-0.000122847	0.000634325	-0.000764951
379	1	-0.000554696	-0.000305060	0.000867068
380	1	0.000438373	0.000264866	0.000246652
381	1	-0.000294169	-0.000141986	0.001084026
382	1	0.001018057	-0.000845007	-0.000334641
383	1	0.001291929	0.001046120	0.001859220
384	8	0.000342176	-0.000835727	0.000154716
385	8	-0.000964627	-0.000045521	0.000700190
386	6	-0.000255037	0.000577436	-0.000313238
387	6	-0.000515264	-0.000354905	0.000498667
388	6	-0.000244052	-0.000046833	0.000207483
389	6	0.000812309	-0.000132637	-0.000415720
390	6	0.000155147	0.000490400	0.000121834
391	6	0.000277076	-0.000246170	-0.000070274
392	6	-0.000567025	0.000175703	-0.000301695
393	6	0.001408704	-0.000310167	-0.000204762
394	1	0.000326348	0.000041282	-0.000320001
395	1	-0.000677156	-0.000149734	0.000478390
396	1	-0.000035268	-0.000020022	0.000012344
397	1	-0.000425291	0.000086820	-0.000045502
398	1	-0.000403947	-0.000039222	0.000040769
399	1	-0.000439355	0.000193839	0.000305854
400	8	0.000150042	-0.001118680	0.000379955
401	8	-0.002427994	-0.001098037	-0.001923528
402	6	-0.000840029	0.001780628	-0.000266025
403	6	-0.000984862	-0.000928240	-0.000538946
404	6	0.001347762	0.001388212	0.001302560
405	6	-0.000225599	-0.000510680	-0.000559038
406	6	0.000117665	0.000645845	0.000419121
407	6	0.001152426	-0.000790695	0.000131331
408	6	-0.000532418	0.000131754	0.000195978
409	6	0.001310060	-0.000045596	-0.000006267
410	1	0.000458079	0.000198626	0.000279607

411	1	-0.000669960	0.000176194	-0.000343469
412	1	0.000059751	0.000139708	0.000078703
413	1	-0.000355599	-0.000028891	0.000123967
414	1	-0.000390715	0.000128937	0.000022286
415	1	-0.000430586	-0.000079378	-0.000231331
416	8	-0.000250678	-0.000706644	0.000135653
417	8	-0.000412822	-0.000109905	-0.000422735
418	6	-0.001179251	-0.000025933	0.000214627
419	6	-0.000409922	-0.000124887	0.000059947
420	6	-0.000253522	0.000594997	0.000318658
421	6	0.000720603	-0.000320163	-0.000260075
422	6	0.000084481	-0.000235405	-0.000074995
423	6	0.000860031	-0.000422946	-0.000232630
424	6	-0.000468193	-0.000083888	0.000422415
425	6	0.001403674	-0.000378532	-0.000212631
426	1	0.000286899	-0.000194092	-0.000062550
427	1	-0.000702945	0.000468252	0.000197279
428	1	-0.000041487	0.000090916	0.000050462
429	1	-0.000454684	0.000040594	0.000133905
430	1	-0.000425915	0.000325461	-0.000009695
431	1	-0.000396183	-0.000001009	0.000012495
432	8	0.000185731	0.000257511	-0.001917867
433	8	-0.000417494	0.001120380	0.000068511
434	6	0.000262290	-0.000026501	0.001294193
435	6	-0.000244561	0.000108918	-0.000501814
436	6	-0.000307963	-0.001216803	0.000608977
437	6	0.000843602	0.000610829	-0.000176720
438	6	0.000179328	-0.000138990	0.000528953
439	6	0.000159086	-0.000004802	-0.000435904
440	6	-0.000504996	0.000237492	-0.000002827
441	6	0.001328011	0.000190013	-0.000165081
442	1	0.000123710	0.000110651	0.000053443
443	1	-0.000701757	-0.000354209	-0.000163260
444	1	-0.000003918	-0.000077863	-0.000152248
445	1	-0.000405617	-0.000274499	0.000162394
446	1	-0.000363852	0.000056297	0.000076157
447	1	-0.000415919	-0.000049887	-0.000066562
448	1	0.000037010	0.001061209	0.001075327
449	1	0.000333950	0.000172482	-0.000760163
450	1	0.000222028	-0.000148128	-0.000356614
451	1	-0.000032156	-0.000013707	-0.000120366
452	1	0.000554071	-0.000443970	-0.000035286
453	1	0.001221070	-0.001165989	-0.002295604
454	1	0.000744836	0.000859067	-0.000367082
455	1	-0.000187804	-0.000114668	0.000137076
456	1	0.000043862	-0.000067299	0.000145228
457	1	-0.000391545	0.000341329	0.000224708
458	6	-0.001345028	-0.000126458	0.003067916
459	6	0.000050193	-0.000284631	0.000174133
460	6	-0.000323646	0.000366688	0.000223691
461	6	-0.000107166	0.000076768	-0.000761333
462	6	0.000539617	-0.001007581	0.000274334
463	6	-0.000860565	0.000555115	-0.000338651
464	1	0.003341109	-0.003402649	0.004847554
465	1	0.003852778	-0.004865739	-0.002000550
466	1	-0.003111273	0.003977108	-0.004498559
467	1	-0.003235513	0.004837372	0.002357985
468	17	-0.000851946	0.000902286	0.000709125

469	1	0.000159964	0.000146719	0.000255906
470	1	0.000318495	0.000622691	0.000662895
471	1	0.000259683	0.000081914	-0.000177106
472	8	0.001627283	0.000544628	0.001812192
473	1	-0.000673366	-0.000484780	-0.000630356
474	1	-0.000666054	0.000095808	-0.000875107
475	8	0.000239169	0.000589137	0.000462974
476	1	-0.000041708	-0.000081121	-0.000485169
477	1	-0.000414720	-0.000115449	-0.000458712
478	8	0.001761868	-0.000059646	-0.001815218
479	1	-0.000597834	0.000280467	0.000654582
480	1	-0.000769211	-0.000475419	0.000925960
481	6	0.003970369	0.004537403	-0.001410491
482	8	0.001348091	-0.002852218	0.002125511
483	1	0.000686266	-0.001362233	-0.006999854

Sum of electronic and thermal Free Energies= -1284.405132
1 imaginary frequency: -431.97

β-I1

Center Number	Atomic Number	Integrated Forces (Hartrees/Bohr) X	Y	Z
1	6	0.005871881	-0.000436568	0.000176465
2	6	-0.001527830	0.005634897	-0.000056728
3	7	-0.002782242	0.000980780	0.002322853
4	6	-0.000528374	-0.003262819	-0.005131922
5	6	-0.001335442	-0.000184002	-0.003305494
6	6	0.003989634	-0.004096470	-0.000643570
7	1	0.000301036	-0.006848527	-0.000216674
8	1	-0.006397436	-0.001993712	-0.000541708
9	1	0.002887587	0.005154073	0.003890681
10	1	0.001272581	0.000012729	0.005491833
11	1	-0.000096533	0.001282544	-0.006023197
12	1	0.001490232	0.005123626	0.003017799
13	1	-0.005785200	-0.001484339	0.003020076
14	8	-0.000592045	-0.000545482	0.000216130
15	8	-0.004092529	0.005394732	0.004144850
16	6	0.000353235	-0.000083438	-0.000116195
17	6	0.000202633	0.000020333	0.000069519
18	6	0.001245295	-0.003661474	-0.003011358
19	6	-0.000325972	0.000374560	-0.000417478
20	6	-0.000204565	0.000063712	-0.000197815
21	6	-0.000215608	0.000078892	-0.000490768
22	6	0.000187777	-0.000552807	0.000130598
23	6	-0.000555625	0.000831932	-0.001064502
24	1	-0.000187481	-0.000018084	-0.000192250
25	1	0.000541460	-0.000273009	0.000661871
26	1	0.000170275	-0.000003370	-0.000048601
27	1	0.000262651	-0.000118566	0.000451132
28	1	0.000039344	-0.000233724	0.000298766
29	1	0.000180302	-0.000350757	0.000252960
30	8	-0.000027141	-0.000202292	0.000110932
31	8	-0.000953813	-0.003009835	0.000831310
32	6	-0.000527027	0.000340517	0.000351472
33	6	-0.000299268	-0.001272917	0.000154775

34	6	0.000256839	0.002257828	0.000138120
35	6	-0.000202035	-0.000541319	-0.000365642
36	6	0.000048352	0.000476337	0.000232033
37	6	0.000386625	0.000249223	-0.000550665
38	6	0.000066966	-0.000169274	0.000496761
39	6	0.000248794	0.000705360	-0.001026638
40	1	0.000363061	0.000500197	-0.000121858
41	1	-0.000379916	-0.000485194	0.000447816
42	1	-0.000056145	0.000140585	0.000051925
43	1	-0.000194671	-0.000413277	0.000191318
44	1	0.000028500	-0.000187457	0.000293297
45	1	-0.000129072	-0.000109566	0.000377239
46	8	-0.000029815	0.001053762	0.000264682
47	8	-0.000680776	0.000237564	-0.000004698
48	6	-0.000137049	-0.000984604	0.000078216
49	6	-0.000447784	0.000686522	0.000643329
50	6	0.000401673	-0.000373824	-0.000085533
51	6	-0.000243779	0.000291122	-0.000401970
52	6	0.000286525	-0.000067313	-0.000192962
53	6	-0.000223762	0.000540415	-0.000539390
54	6	0.000181317	-0.000304417	0.000361820
55	6	0.000323673	0.000774507	-0.001066554
56	1	0.000145030	-0.000185161	-0.000481678
57	1	-0.000084060	-0.000173183	0.000790462
58	1	-0.000090922	-0.000062937	-0.000090979
59	1	0.000001377	-0.000204271	0.000355179
60	1	-0.000323100	-0.000144141	0.000438074
61	1	-0.000097959	-0.000357791	0.000239131
62	8	-0.001745217	-0.001216410	-0.000970791
63	8	-0.000283270	0.000004302	-0.000434106
64	6	0.001206650	0.001268349	0.000548666
65	6	-0.000373800	-0.000673782	-0.000554054
66	6	0.000722561	-0.000157356	0.001059543
67	6	-0.000548866	0.000653262	-0.000822185
68	6	0.000430849	0.000341326	0.000104659
69	6	-0.000349401	-0.000354960	-0.000447411
70	6	0.000025638	0.000105301	0.000623519
71	6	-0.000585791	0.000601254	-0.001240913
72	1	-0.000087435	0.000362515	0.000044907
73	1	0.000173898	-0.000658502	0.000396245
74	1	0.000121232	0.000095348	0.000029697
75	1	0.000076988	-0.000225866	0.000299161
76	1	0.000330696	-0.000281736	0.000281568
77	1	0.000153194	-0.000117637	0.000469025
78	8	0.000390879	0.000194250	0.000053270
79	8	-0.000720630	0.002973172	-0.000656460
80	6	-0.000692881	-0.000325983	-0.000496415
81	6	-0.000150710	0.001267568	0.000032803
82	6	-0.000064797	-0.002196339	-0.000200073
83	6	-0.000152657	0.000498654	0.000443518
84	6	0.000052699	-0.000487267	-0.000253509
85	6	0.000364630	-0.000315574	0.000534263
86	6	0.000024602	0.000159486	-0.000518670
87	6	0.000337905	-0.000794218	0.000953241
88	1	0.000272675	-0.000487478	0.000007164
89	1	-0.000451048	0.000588741	-0.000369345
90	1	-0.000078481	-0.000150839	-0.000062281
91	1	-0.000171421	0.000141911	-0.000319540

92	1	-0.000171899	0.000439485	-0.000215610
93	1	-0.000020717	0.000200155	-0.000284236
94	8	-0.000536265	-0.000249970	-0.000132871
95	8	0.000395594	-0.000156490	-0.003233252
96	6	0.000967949	0.000611965	0.000136715
97	6	0.000016150	-0.000430406	-0.001185777
98	6	0.000242334	0.000785218	0.002168660
99	6	0.000016894	-0.000475888	-0.000456277
100	6	0.000174403	0.000362555	0.000491527
101	6	-0.000470913	-0.000461159	0.000515171
102	6	0.000048542	0.000450031	-0.000261475
103	6	-0.000344475	-0.000701451	0.001065810
104	1	-0.000190379	0.000162456	0.000474995
105	1	0.000401555	0.000203666	-0.000682476
106	1	0.000100343	0.000052235	0.000110871
107	1	0.000180489	0.000293425	-0.000256223
108	1	0.000161543	0.000060553	-0.000498278
109	1	0.000003417	0.000226474	-0.000273796
110	8	0.001178683	-0.001461692	-0.000955318
111	8	-0.000060524	-0.000395354	0.000304747
112	6	-0.000702214	0.001061161	0.000888681
113	6	0.000090315	-0.000718346	-0.000554023
114	6	-0.000443012	0.000714894	-0.000376188
115	6	0.000338358	-0.000581932	0.000718786
116	6	-0.000142810	0.000197938	0.000343484
117	6	-0.000014772	-0.000586596	0.000025362
118	6	0.000147007	0.000363970	-0.000287825
119	6	0.000488069	-0.000864142	0.000939664
120	1	0.000200291	0.000264508	0.000274533
121	1	-0.000224022	0.000208683	-0.000790900
122	1	-0.000105346	0.000058319	0.000073113
123	1	-0.000111074	0.000361616	-0.000214962
124	1	-0.000070490	0.000216533	-0.000315142
125	1	-0.000332036	0.000229706	-0.000297518
126	8	-0.001297597	0.001277051	0.001303390
127	8	-0.000166216	-0.000269756	0.000648027
128	6	0.000890969	-0.001099249	-0.000847820
129	6	-0.000225573	0.000650850	0.000659472
130	6	0.000648433	0.000268308	-0.001107757
131	6	-0.000405077	-0.000639345	0.000903335
132	6	0.000213842	-0.000403112	-0.000235887
133	6	-0.000131784	0.000104726	0.000634392
134	6	-0.000203169	0.000167772	-0.000417029
135	6	-0.000389644	-0.000689548	0.001129812
136	1	-0.000147696	-0.000273986	-0.000130618
137	1	0.000199199	0.000692562	-0.000474802
138	1	0.000083932	-0.000077630	-0.000043106
139	1	0.000075136	0.000119454	-0.000430229
140	1	0.000038234	0.000247054	-0.000295805
141	1	0.000306834	0.000285895	-0.000308707
142	8	-0.001606776	0.000200630	-0.001614212
143	1	0.000654811	-0.000400555	0.000592256
144	1	0.000717381	0.000327646	0.000827378
145	8	0.000189377	0.000599078	-0.000028655
146	1	0.000121231	0.000760268	0.000500491
147	1	0.000842818	0.000255072	-0.000592929
148	8	0.002796377	-0.001986591	-0.008788930
149	1	-0.000844957	0.003154130	0.004822950

150	1	-0.003086722	-0.001078552	0.004519882
151	8	0.000054525	-0.000644909	0.000170350
152	1	0.000868853	-0.000398122	0.000374606
153	1	-0.000032072	-0.000727434	-0.000645158
154	8	0.004851582	0.002374007	0.008698368
155	1	-0.004262418	0.000231896	-0.004928397
156	1	-0.001889913	-0.003053351	-0.005324971
157	1	0.000170454	-0.000164940	-0.000103720
158	1	-0.000326598	-0.000637168	0.000271381
159	1	-0.000146598	0.000254415	-0.000082127
160	1	0.000158997	-0.000491573	0.000186083
161	1	0.000286663	-0.000416904	-0.000105639
162	8	-0.000345714	-0.000036445	-0.000139041
163	8	-0.002686044	-0.001063119	-0.000285319
164	6	0.000539271	-0.000482462	0.000174011
165	6	-0.001100926	-0.000636509	-0.000230543
166	6	0.002702207	0.001118725	0.000654299
167	6	-0.000781258	-0.000440278	0.000119532
168	6	0.000659292	0.000005597	-0.000005226
169	6	-0.000197064	0.000773748	0.000257308
170	6	-0.000027926	-0.000190219	-0.000479922
171	6	0.000419725	0.000840475	0.000784539
172	1	0.000461035	0.000301793	0.000016725
173	1	-0.000187919	-0.000745196	-0.000083042
174	1	-0.000028220	0.000151003	-0.000098048
175	1	0.000003002	-0.000299610	-0.000166663
176	1	-0.000315115	-0.000351548	-0.000112058
177	1	-0.000125169	-0.000189624	-0.000361560
178	8	-0.000646601	-0.000125519	0.000077615
179	8	0.000547456	0.000094272	-0.000196456
180	6	0.000351486	0.000277209	-0.000235977
181	6	-0.000217462	-0.000309944	0.000223720
182	6	0.000043077	0.000133053	-0.000444881
183	6	-0.000145387	0.000471637	0.000500889
184	6	0.000112618	-0.000134666	0.000361032
185	6	-0.000151818	0.000196262	0.000228914
186	6	-0.000188079	-0.000509671	-0.000014855
187	6	-0.000215425	0.001117431	0.000799295
188	1	-0.000019701	0.000020614	-0.000025681
189	1	0.000151274	-0.000308168	-0.000825115
190	1	0.000052641	-0.000072248	0.000075154
191	1	-0.000065733	-0.000257940	-0.000276452
192	1	0.000267127	-0.000308343	-0.000355054
193	1	0.000039123	-0.000427842	-0.000146480
194	8	0.000023324	-0.001134730	0.000399868
195	8	0.000390198	0.000234715	-0.000643924
196	6	0.000075992	0.000540082	-0.000108650
197	6	0.000081726	-0.000536133	-0.000319286
198	6	-0.000226329	-0.000021257	0.000551521
199	6	0.000002831	0.000366765	0.000188215
200	6	0.000247924	0.000038670	0.000143587
201	6	0.000051623	0.000210175	0.000428014
202	6	0.000184516	-0.000423481	-0.000197994
203	6	0.000132903	0.000833952	0.000941696
204	1	-0.000034059	0.000216844	0.000139795
205	1	-0.000546949	-0.000356655	-0.000559158
206	1	0.000107368	-0.000004478	0.000008981
207	1	-0.000141789	-0.000151171	-0.000439065

208	1	0.000061148	-0.000275281	-0.000292252
209	1	-0.000111217	-0.000279788	-0.000139791
210	8	-0.001091265	0.000590922	0.000484536
211	8	-0.000651148	-0.000565190	0.000918102
212	6	0.001405826	-0.000166169	-0.001023846
213	6	-0.000501339	-0.000457742	0.000072519
214	6	0.000348958	0.000329409	-0.000401626
215	6	-0.000325117	0.000067543	0.000431626
216	6	0.000022416	0.000410058	-0.000246202
217	6	-0.000586387	0.000725551	0.000561176
218	6	0.000318418	-0.000173521	-0.000565107
219	6	-0.000308199	0.001126220	0.000689877
220	1	0.000093163	0.000192062	0.000036044
221	1	0.000379863	-0.000675628	-0.000302793
222	1	-0.000042489	0.000015454	0.000034034
223	1	0.000182198	-0.000473573	-0.000060883
224	1	-0.000058389	-0.000308352	-0.000175546
225	1	0.000109213	-0.000273969	-0.000320083
226	1	0.000429672	0.000206571	-0.000871238
227	1	0.000114722	-0.000017345	0.000152213
228	1	0.000341260	-0.000541609	0.000471914
229	1	-0.000084556	0.000017314	-0.000104786
230	1	0.000833024	0.000363632	0.000667711
231	1	0.000021107	-0.000030273	-0.000272823
232	1	0.000333382	-0.000133921	0.000754717
233	1	-0.000173676	-0.000308613	0.000137911
234	1	-0.000375253	0.000654703	-0.000374912
235	1	0.000341816	0.000359878	-0.000288982
236	8	-0.000167434	-0.000012270	0.000224275
237	8	-0.002293550	0.001398688	0.000884462
238	6	0.000430500	0.000414326	-0.000356366
239	6	-0.000810411	0.000634656	0.000488297
240	6	0.002207052	-0.001191374	-0.001159437
241	6	-0.000560207	0.000288024	-0.000061195
242	6	0.000441362	-0.000096212	-0.000110590
243	6	-0.000227416	-0.000710699	-0.000224415
244	6	0.000124439	0.000181637	0.000463391
245	6	0.000176237	-0.000941258	-0.000817158
246	1	0.000361646	-0.000281956	-0.000155727
247	1	-0.000070019	0.000735638	0.000122343
248	1	-0.000040956	-0.000116125	0.000094483
249	1	-0.000029635	0.000229046	0.000382707
250	1	0.000050914	0.000318963	0.000143056
251	1	-0.000221665	0.000411299	0.000203374
252	8	0.000148058	0.000202921	0.000116253
253	8	0.002375568	0.000591343	0.000844519
254	6	-0.000324282	-0.000016442	0.000288227
255	6	0.000929024	0.000420200	0.000423796
256	6	-0.002379553	-0.000898867	-0.000580014
257	6	0.000528225	-0.000206516	0.000263293
258	6	-0.000439100	-0.000041917	-0.000049187
259	6	0.000105455	-0.000378687	-0.000415606
260	6	0.000018115	0.000600006	-0.000061498
261	6	-0.000430114	-0.001080442	-0.000510135
262	1	-0.000404939	-0.000091414	-0.000217501
263	1	0.000297484	0.000246366	0.000642242
264	1	0.000015804	0.000051525	-0.000156537
265	1	0.000124094	0.000423098	0.000089852

266	1	0.000013391	0.000237003	0.000211618
267	1	0.000379021	0.000283814	0.000291054
268	8	0.000200107	-0.000047454	0.001363185
269	8	-0.000467286	0.000092927	-0.000555692
270	6	-0.000204221	0.000194381	-0.000745735
271	6	0.000182614	-0.000070136	0.000263022
272	6	0.000199945	0.000357601	0.000175645
273	6	-0.000335406	-0.000549746	-0.000111531
274	6	-0.000201015	-0.000123965	-0.000103160
275	6	-0.000105701	-0.000460702	-0.000000357
276	6	0.000179123	0.000177674	0.000426503
277	6	-0.000376020	-0.001119279	-0.000523756
278	1	-0.000090822	-0.000030138	-0.000017915
279	1	0.000437089	0.000706445	0.000121628
280	1	-0.000077301	0.000014842	-0.000032351
281	1	0.000167088	0.000254632	0.000234195
282	1	0.000200712	0.000452652	0.000014616
283	1	-0.000016584	0.000337919	0.000171288
284	8	0.000061562	0.001219562	-0.000427775
285	8	0.000595623	-0.000534203	0.000552745
286	6	0.000064538	-0.000484916	0.000350040
287	6	-0.000097586	0.000189224	0.000037289
288	6	-0.000161304	0.000372800	-0.000152970
289	6	0.000139354	-0.000504301	-0.000265853
290	6	0.000139435	0.000014925	-0.000295548
291	6	0.000106212	-0.000363262	-0.000486096
292	6	-0.000140334	0.000484429	0.000088106
293	6	0.000166417	-0.000881044	-0.000892398
294	1	0.000053049	-0.000083700	-0.000071883
295	1	-0.000484312	0.000441926	0.000598921
296	1	0.000069026	-0.000001409	-0.000011127
297	1	-0.000096841	0.000321354	0.000179249
298	1	-0.000116281	0.000197495	0.000450741
299	1	0.000079513	0.000233373	0.000246764
300	1	0.000349128	0.000396645	0.000461775
301	1	-0.002307372	0.000884602	0.001602169
302	1	0.000144242	-0.000409802	-0.000585938
303	1	-0.002703166	-0.000601688	-0.001266843
304	1	-0.001045805	-0.000674318	0.002565280
305	1	0.000310564	-0.000709524	-0.001190346
306	1	-0.000977515	-0.000884768	0.000593475
307	1	0.000656328	0.000557999	0.001073385
308	1	-0.001093743	0.000961331	-0.000322739
309	1	0.001866061	-0.002834346	-0.000351305
310	8	-0.000457948	-0.000401975	-0.000466758
311	8	0.002014741	0.000079333	-0.002191983
312	6	0.000543304	0.000059049	0.000474883
313	6	0.000773072	-0.000049919	-0.001204124
314	6	-0.000841694	-0.000680996	0.001494957
315	6	-0.000315741	0.000038808	-0.000460785
316	6	0.000053733	-0.000105321	0.000323723
317	6	-0.000634284	0.000133818	-0.000063067
318	6	0.000597598	0.000119626	0.000192057
319	6	-0.001262449	-0.000084380	0.000216574
320	1	-0.000482913	0.000023253	0.000559080
321	1	0.000664110	-0.000189025	-0.000421421
322	1	-0.000030209	-0.000102063	0.000115461
323	1	0.000334984	-0.000089362	-0.000043386

324	1	0.000343964	0.000092104	-0.000300839
325	1	0.000355663	0.000063374	0.000042863
326	8	-0.000194303	0.000532193	0.000531206
327	8	0.002458865	-0.000376871	0.001943505
328	6	0.000321857	-0.000109357	-0.000450446
329	6	0.001008892	-0.000076926	0.001011969
330	6	-0.001110138	0.000874605	-0.001356043
331	6	-0.000133418	-0.000088022	0.000469639
332	6	-0.000033130	0.000090361	-0.000292737
333	6	-0.000604110	-0.000072749	0.000240581
334	6	0.000507622	-0.000219652	-0.000312714
335	6	-0.001261045	0.000286585	0.000052196
336	1	-0.000634148	0.000028286	-0.000462338
337	1	0.000809240	0.000188921	0.000207397
338	1	-0.000051693	0.000087744	-0.000098304
339	1	0.000413977	-0.000206912	0.000269159
340	1	0.000342291	-0.000123465	-0.000124924
341	1	0.000355605	0.000049118	-0.000031697
342	8	-0.000309261	0.000677640	0.001990352
343	8	0.000457062	0.001068595	-0.000579817
344	6	0.000000685	-0.000287000	-0.001404497
345	6	0.000212051	0.000018662	0.000490490
346	6	0.000206312	-0.000984980	-0.000260141
347	6	-0.000855967	0.000515471	-0.000052173
348	6	-0.000110439	0.000028781	-0.000418320
349	6	-0.000464795	0.000183093	0.000457845
350	6	0.000526201	0.000118326	0.000252607
351	6	-0.001265876	0.000430403	-0.000046283
352	1	-0.000073068	0.000167016	-0.000099879
353	1	0.000742251	-0.000444741	0.000169754
354	1	0.000020153	-0.000082652	0.000166038
355	1	0.000318767	-0.000011497	-0.000059415
356	1	0.000400448	-0.000099967	0.000090118
357	1	0.000363648	-0.000365186	-0.000067427
358	8	-0.000847380	-0.000518351	-0.001800594
359	8	0.000592527	-0.001085414	0.000546315
360	6	0.000312485	0.000139730	0.001298396
361	6	-0.000092739	0.000029761	-0.000508651
362	6	0.000357746	0.000858607	0.000139448
363	6	-0.000922480	-0.000385271	0.000191984
364	6	-0.000031975	-0.000024110	0.000440232
365	6	-0.000526695	-0.000011193	-0.000313278
366	6	0.000413385	-0.000152024	-0.000378993
367	6	-0.001270782	-0.000290991	0.000333571
368	1	-0.000004782	-0.000153679	0.000038211
369	1	0.000789851	0.000277907	-0.000342889
370	1	-0.000021192	0.000074271	-0.000156039
371	1	0.000365955	0.000328908	0.000024762
372	1	0.000343107	-0.000017829	-0.000015636
373	1	0.000401349	0.000054278	-0.000204285
374	1	-0.000248342	0.000141086	-0.000475368
375	1	0.000947838	-0.001134321	-0.000203078
376	1	0.000531513	0.000256510	-0.000095240
377	1	0.002634273	0.001524376	0.000100833
378	1	-0.000225252	-0.000500558	-0.000042593
379	1	-0.000608161	0.000870126	0.000325651
380	1	0.000398360	0.000156709	-0.000284840
381	1	-0.000263274	0.001390742	0.000525157

382	1	0.001066645	-0.000224540	0.000739531
383	1	0.001259027	0.002117664	-0.001572559
384	8	0.000528621	-0.000246536	0.000443778
385	8	-0.000671678	0.000485297	-0.000252303
386	6	-0.000550339	0.000215829	0.000161081
387	6	-0.000099185	0.000152115	-0.000024530
388	6	-0.000000400	0.000368089	0.000150535
389	6	0.000725624	-0.000307292	-0.000207647
390	6	-0.000006047	-0.000145761	-0.000199960
391	6	0.000463173	-0.000284091	-0.000058779
392	6	-0.000512551	-0.000154899	0.000218364
393	6	0.001332637	-0.000367269	-0.000005352
394	1	0.000030608	-0.000305249	0.000104765
395	1	-0.000648269	0.000534800	0.000152062
396	1	0.000007582	0.000056779	0.000061077
397	1	-0.000406122	0.000329644	-0.000123883
398	1	-0.000386885	-0.000036857	-0.000045508
399	1	-0.000387550	0.000104559	0.000128142
400	8	0.000105669	-0.000590473	-0.000431614
401	8	-0.002207560	-0.001820677	0.000987066
402	6	-0.000037989	0.000428206	0.000280464
403	6	-0.000886889	-0.001112723	0.000225520
404	6	0.000841030	0.001152426	-0.001074575
405	6	0.000178102	-0.000367800	0.000032787
406	6	0.000010045	0.000093656	-0.000054538
407	6	0.000335271	-0.000212146	0.000094155
408	6	-0.000486941	0.000428585	0.000296717
409	6	0.001213784	-0.000212504	-0.000455287
410	1	0.000625021	0.000544240	-0.000180146
411	1	-0.000675310	-0.000298485	-0.000036430
412	1	0.000050783	0.000051781	-0.000098469
413	1	-0.000323270	0.000184876	0.000136747
414	1	-0.000340192	0.000026187	0.000001784
415	1	-0.000396011	-0.000138127	0.000267435
416	8	0.000136358	0.000000143	0.000336720
417	8	-0.000681815	-0.000703312	0.001229922
418	6	-0.000623165	0.000515060	-0.000218206
419	6	-0.000389800	-0.000215860	0.000143899
420	6	0.000201251	0.000063524	-0.000819995
421	6	0.000154706	-0.000426039	0.000302740
422	6	0.000238906	0.000385354	0.000193878
423	6	0.000963156	-0.000512253	0.000149947
424	6	-0.000354470	0.000576732	-0.000101487
425	6	0.001263259	-0.000424650	0.000368167
426	1	0.000178018	-0.000025030	0.000024764
427	1	-0.000722049	0.000281802	-0.000414862
428	1	0.000015560	0.000154387	-0.000153753
429	1	-0.000422049	-0.000017267	-0.000262064
430	1	-0.000329179	0.000062747	0.000027630
431	1	-0.000425346	0.000223027	-0.000056352
432	8	0.000077792	-0.002238334	-0.000225212
433	8	-0.000566735	-0.000191355	-0.001130046
434	6	0.000208726	0.001464454	-0.000210578
435	6	-0.000316198	-0.000593284	0.000041163
436	6	-0.000235148	0.000716289	0.001108148
437	6	0.000820097	-0.000301926	-0.000779833
438	6	0.000253281	0.000634806	0.000053112
439	6	0.000220588	-0.000711671	-0.000014459

440	6	-0.000525281	0.000012510	-0.000218612
441	6	0.001307283	-0.000292352	-0.000377342
442	1	0.000127326	0.000028978	-0.000182186
443	1	-0.000702747	-0.000053103	0.000589553
444	1	0.000009912	-0.000081766	0.000112312
445	1	-0.000392741	-0.000030046	0.000119547
446	1	-0.000366326	0.000215370	0.000328588
447	1	-0.000396636	0.000092919	-0.000022792
448	1	-0.000266946	0.000519438	-0.000735164
449	1	0.000208755	-0.000705246	-0.000234398
450	1	0.000554983	-0.000301057	0.000260681
451	1	-0.000099019	0.000177016	0.000356930
452	1	0.000856473	0.000176709	-0.001056843
453	1	0.001218790	-0.002180932	0.001356790
454	1	0.000712899	-0.000479002	-0.000848018
455	1	-0.000012214	0.000116970	0.000075910
456	1	0.000043232	0.000810465	-0.000083875
457	1	-0.000478964	0.000285471	-0.000326860
458	6	-0.001366163	0.001618937	0.002774767
459	6	0.000063322	0.000532832	-0.000227813
460	6	-0.000346164	-0.000200712	-0.000012129
461	6	0.000073726	-0.000038280	-0.000172565
462	6	0.000338768	0.000169898	0.000400439
463	6	-0.000664068	-0.000404837	-0.000294453
464	1	0.004234813	0.002672095	0.004067544
465	1	0.005861095	-0.002565563	0.000318027
466	1	-0.004183756	-0.002215637	-0.004239998
467	1	-0.005506258	0.003285544	-0.000465560
468	17	0.001419375	0.001708204	-0.000969033
469	1	0.000163789	0.000141323	-0.000180238
470	1	0.000260700	0.000450770	-0.000615403
471	1	0.000146760	-0.000176992	-0.000044304
472	8	0.001613158	0.001713425	-0.000964709
473	1	-0.000695426	-0.000510433	0.000739668
474	1	-0.000719004	-0.000951927	0.000085047
475	8	-0.001273182	-0.000393019	0.001887868
476	1	0.000406370	-0.000193904	-0.000787699
477	1	0.000571514	0.000566709	-0.000670941
478	8	0.001797077	-0.001697220	0.000577138
479	1	-0.000910836	0.000695223	-0.000592303
480	1	-0.000493777	0.000765132	-0.000002032
481	6	-0.001164227	-0.002024248	-0.001488247
482	8	0.004169757	0.004637433	0.001642953
483	1	0.001639240	-0.005082202	-0.003664017

Sum of electronic and thermal Free Energies= -1284.426305
0 imaginary frequency

β-TS2

Center Number	Atomic Number	Integrated Forces (Hartrees/Bohr) X	Y	Z
1	6	-0.001972631	-0.004929200	-0.000519596
2	6	-0.004084817	0.001938131	-0.000254274
3	7	-0.002071400	0.006466006	0.004398676
4	6	0.000286714	-0.005265580	0.003185261

5	6	0.009516006	0.005559155	-0.016170050
6	6	0.004737769	-0.006025849	-0.000839245
7	1	0.006488450	-0.001663918	0.001270008
8	1	0.001859097	0.005545821	0.004356066
9	1	-0.004327770	-0.000413075	-0.005668536
10	1	-0.005448559	-0.002245958	0.007218858
11	1	-0.001360510	0.001513715	0.006177246
12	1	-0.005396792	-0.001410285	-0.003230476
13	1	0.002394769	0.006157503	-0.002306567
14	8	-0.000402303	-0.000414434	0.000262972
15	8	0.003683196	0.005704596	0.002134726
16	6	0.000311246	0.000268199	0.000154079
17	6	0.000169720	-0.000492519	0.000238377
18	6	-0.003050687	-0.002668132	-0.001431659
19	6	0.000020139	0.000145579	-0.001194302
20	6	0.000178883	0.000574306	0.000008167
21	6	-0.000258350	-0.000286753	-0.000581391
22	6	-0.000343697	0.000111217	0.000307910
23	6	0.000483575	0.000179039	-0.001468878
24	1	-0.000121538	0.000230117	-0.000287945
25	1	-0.000064893	-0.000382048	0.000754795
26	1	0.000108137	-0.000017287	0.000181612
27	1	0.000094941	-0.000088283	0.000535394
28	1	-0.000192613	0.000039841	0.000438055
29	1	-0.000216489	-0.000123568	0.000402651
30	8	-0.000517834	0.000407628	0.000256594
31	8	-0.003520053	-0.000023386	0.000825018
32	6	0.000217749	0.000235146	0.000183006
33	6	-0.001502517	-0.000058045	0.000277419
34	6	0.002551772	0.000481989	-0.000065986
35	6	-0.000888475	0.000115383	-0.000453403
36	6	0.000547455	0.000232786	0.000333538
37	6	0.000314629	-0.000399317	-0.000839310
38	6	-0.000217123	0.000034721	0.000409645
39	6	0.000676854	-0.000098103	-0.001050360
40	1	0.000558015	-0.000130944	-0.000158228
41	1	-0.000514633	0.000259938	0.000465807
42	1	0.000145984	0.000105755	0.000070595
43	1	-0.000403714	0.000019067	0.000222759
44	1	-0.000130070	-0.000050830	0.000308236
45	1	-0.000148622	0.000121884	0.000336182
46	8	0.001358229	0.001827333	-0.001021238
47	8	-0.001375624	0.001796937	-0.000099648
48	6	-0.000194675	-0.000647840	0.001716215
49	6	-0.000160804	0.000598476	-0.000075241
50	6	0.001156007	-0.001055916	0.000080071
51	6	-0.000457169	0.000535526	-0.000428059
52	6	0.000443195	-0.000717368	0.000023533
53	6	-0.000263716	0.000046853	-0.001490028
54	6	0.000291396	-0.000348053	0.000723369
55	6	0.000401177	-0.000036377	-0.001360034
56	1	0.000151597	-0.000262160	-0.000257479
57	1	-0.000093284	0.000276056	0.000925986
58	1	-0.000057288	-0.000036849	-0.000053247
59	1	-0.000097238	-0.000086534	0.000453811
60	1	-0.000139399	0.000246933	0.000438283
61	1	-0.000244529	-0.000014483	0.000274710
62	8	-0.001890159	0.000832059	-0.001290896

63	8	-0.000382356	0.000284925	-0.000557410
64	6	0.001729797	-0.000346637	0.000484679
65	6	-0.000865242	-0.000172326	-0.000275397
66	6	0.000168318	-0.000321978	0.000832403
67	6	0.000176792	0.000436545	-0.000888515
68	6	0.000600121	-0.000316162	0.000192679
69	6	-0.000575528	-0.000014313	-0.000243397
70	6	0.000247593	0.000155643	0.000717697
71	6	0.000182834	0.000467387	-0.001434861
72	1	0.000327785	0.000265409	-0.000051052
73	1	-0.000513951	-0.000341978	0.000517726
74	1	0.000151991	-0.000068023	-0.000013015
75	1	-0.000143654	-0.000069122	0.000314153
76	1	-0.000093205	-0.000342921	0.000430504
77	1	-0.000044836	-0.000036247	0.000515477
78	8	0.000431254	0.000176239	-0.000194825
79	8	0.003380804	0.001723992	-0.000630314
80	6	-0.000543282	0.000536436	-0.000534964
81	6	0.001250165	0.000768812	-0.000243591
82	6	-0.002506768	-0.001263402	-0.000015021
83	6	0.000464043	0.000390957	0.000238075
84	6	-0.000402820	-0.000072520	-0.000270915
85	6	-0.000180879	-0.000565521	0.000736516
86	6	0.000212916	0.000046719	-0.000601873
87	6	-0.000578642	-0.000393457	0.001055414
88	1	-0.000429120	-0.000358635	0.000087076
89	1	0.000361219	0.000574996	-0.000454639
90	1	-0.000192513	0.000016610	-0.000099786
91	1	0.000053283	0.000157013	-0.000358496
92	1	0.000370547	0.000297259	-0.000197264
93	1	0.000184836	0.000028723	-0.000282340
94	8	-0.000643641	0.000487151	-0.000151268
95	8	0.000086849	-0.000903973	-0.002989586
96	6	0.001050802	-0.000689103	0.000207859
97	6	-0.000447639	-0.000251745	-0.000995758
98	6	0.000766154	0.000296569	0.001994205
99	6	-0.000429715	-0.000107690	-0.000326219
100	6	0.000275394	-0.000025500	0.000508637
101	6	-0.000620238	0.000430233	0.000460757
102	6	0.000493871	-0.000005747	-0.000218684
103	6	-0.000873350	0.000328755	0.000989103
104	1	0.000162213	0.000263618	0.000388114
105	1	0.000352003	-0.000490922	-0.000589751
106	1	0.000065589	-0.000113039	0.000125584
107	1	0.000382474	-0.000102711	-0.000236448
108	1	0.000118430	-0.000223735	-0.000430660
109	1	0.000216694	0.000018927	-0.000290722
110	8	-0.000781104	-0.001261318	-0.000288231
111	8	-0.000462960	-0.000070119	0.000346078
112	6	0.000616719	0.000791693	0.000273930
113	6	-0.000782815	-0.000207681	-0.000285561
114	6	0.000517154	0.000285239	-0.000299388
115	6	-0.000356287	-0.000130577	0.000459409
116	6	0.000056817	0.000000822	0.000321960
117	6	-0.000300838	-0.000148871	0.000239602
118	6	0.000308048	-0.000096147	-0.000256826
119	6	-0.000684036	-0.000592890	0.000977976
120	1	0.000323365	-0.000028390	0.000200198

121	1	0.000166677	0.000143521	-0.000764570
122	1	0.000014268	0.000129932	0.000088774
123	1	0.000297819	0.000201321	-0.000227907
124	1	0.000183935	0.000094858	-0.000319375
125	1	0.000048969	0.000305196	-0.000419608
126	8	0.000591648	0.002711572	0.000990899
127	8	0.000034152	0.000607172	0.000995052
128	6	-0.000982829	-0.001805797	-0.000580901
129	6	0.000404922	0.000813320	0.000708569
130	6	0.000956134	-0.001227539	-0.001254751
131	6	-0.000922274	0.000440609	0.000979875
132	6	-0.000224640	-0.000570519	-0.000153776
133	6	0.000173845	0.000457467	0.000365259
134	6	0.000010068	0.000100426	-0.000539794
135	6	-0.000760236	0.000442056	0.001125323
136	1	-0.000206444	-0.000116455	-0.000256559
137	1	0.000788993	0.000068848	-0.000443863
138	1	-0.000038280	-0.000164967	-0.000039001
139	1	0.000145716	-0.000109786	-0.000416878
140	1	0.000250966	-0.000036459	-0.000280684
141	1	0.000348592	-0.000288227	-0.000298365
142	8	-0.001120763	0.001252051	-0.001369971
143	1	-0.000121800	-0.000541386	0.000568338
144	1	0.000759750	-0.000442413	0.000746262
145	8	0.000977784	0.000144365	-0.000202699
146	1	0.000335478	0.000282988	0.001063418
147	1	0.000612037	-0.000945621	-0.000378765
148	8	-0.001408123	-0.008618549	-0.008715843
149	1	0.001970333	0.004217637	0.004530776
150	1	-0.001415278	0.005189041	0.001870965
151	8	-0.000514889	-0.000222597	0.000237616
152	1	-0.000053679	-0.000845796	0.000471549
153	1	-0.000766237	-0.000145801	-0.000609412
154	8	0.003748570	-0.002603174	0.009121881
155	1	-0.000853873	0.003198364	-0.005683606
156	1	-0.003677374	0.000182212	-0.005355724
157	1	0.000193132	-0.001643200	-0.000354834
158	1	-0.000126967	0.000463377	-0.000322725
159	1	0.000089966	0.000473230	-0.000245093
160	1	0.000673112	-0.001250302	-0.000501771
161	1	-0.001196631	-0.000735795	0.002157629
162	8	0.000170588	0.000949227	-0.000709480
163	8	-0.002306086	0.002175150	-0.001372471
164	6	-0.000746285	-0.001223664	0.001166791
165	6	-0.000873556	0.000741651	-0.001514519
166	6	0.002712344	-0.002141268	0.001985870
167	6	-0.001611231	0.000879269	-0.000557429
168	6	0.000382774	-0.001086020	0.000707594
169	6	0.000798973	0.000636842	-0.000319004
170	6	-0.000236553	-0.000028331	-0.000459381
171	6	0.000816842	-0.000122077	0.000797932
172	1	0.000356703	-0.000224341	0.000289506
173	1	-0.000615826	-0.000118926	0.000022193
174	1	0.000258053	0.000091634	0.000024567
175	1	-0.000255216	-0.000141527	-0.000137528
176	1	-0.000337382	0.000157461	-0.000226659
177	1	-0.000170555	-0.000029204	-0.000401676
178	8	-0.000358107	0.000154343	0.001938869

179	8	-0.000399980	-0.000606118	0.001194203
180	6	-0.000423170	0.000812054	-0.002628167
181	6	0.000313377	-0.000091195	0.000927519
182	6	0.000449693	0.000329473	-0.001075853
183	6	-0.000222278	0.000198545	0.000842537
184	6	0.000359027	0.000286438	0.000177666
185	6	-0.000774800	0.000394364	0.001020251
186	6	-0.000693943	0.000430775	-0.000620786
187	6	0.000637076	0.000636227	0.001151170
188	1	0.001110801	0.000112566	0.000129583
189	1	0.000092519	-0.000370625	-0.000750429
190	1	-0.000048813	0.000110457	-0.000030891
191	1	-0.000208777	-0.000018362	-0.000365502
192	1	-0.000048852	-0.000401164	-0.000389474
193	1	-0.000307287	-0.000221874	-0.000193452
194	8	-0.002153140	-0.000472860	-0.000081329
195	8	-0.000932850	0.000493703	0.002142493
196	6	0.002067854	0.000379000	0.000041549
197	6	-0.001782240	-0.001008161	0.000369754
198	6	0.000602529	0.000122274	-0.002693766
199	6	0.000159186	-0.000244833	0.001763031
200	6	0.001021899	-0.000176324	-0.000762205
201	6	-0.000906816	0.000160627	0.000296981
202	6	0.000362031	-0.000588301	-0.000261437
203	6	0.000295431	0.000731151	0.001239624
204	1	0.000561381	0.000436874	0.000049531
205	1	-0.000362543	0.000185947	-0.000515993
206	1	0.000196743	0.000003256	-0.000142337
207	1	-0.000046912	-0.000096716	-0.000564335
208	1	-0.000144654	-0.000337420	-0.000350780
209	1	-0.000196169	-0.000088096	-0.000267150
210	8	0.001022764	0.000792545	-0.002083964
211	8	-0.001308120	0.000900519	0.000956132
212	6	0.000256734	-0.001707498	-0.001089257
213	6	-0.000562244	0.000583413	0.000359220
214	6	0.000779481	-0.000760654	-0.000592681
215	6	-0.000300935	0.001233320	0.000087502
216	6	0.000496705	-0.000092075	-0.000382659
217	6	0.000206454	0.001202751	0.000529852
218	6	0.000448480	-0.001021533	-0.000216896
219	6	0.000666493	0.000922532	0.000793658
220	1	-0.000014502	-0.000140849	-0.000320212
221	1	-0.000397452	-0.000786092	-0.000305841
222	1	-0.000039771	0.000063177	-0.000002036
223	1	-0.000298056	-0.000383054	-0.000063433
224	1	-0.000253324	-0.000122147	-0.000150259
225	1	-0.000159993	-0.000291026	-0.000314410
226	1	0.000614874	-0.000865446	-0.001057515
227	1	0.000025398	0.000011564	0.000222420
228	1	-0.000467973	-0.000338489	0.000555287
229	1	-0.000029917	0.000086583	-0.000364323
230	1	0.000689789	-0.000538514	0.000852392
231	1	0.000350943	-0.000254104	-0.000267666
232	1	-0.000168439	-0.000442317	0.000818936
233	1	-0.000293335	0.000012257	0.000030346
234	1	0.000693157	0.000314926	-0.000427193
235	1	-0.000157688	-0.000082679	-0.000612032
236	8	-0.000056002	0.000234394	0.000203915

237	8	0.000344389	0.002718171	0.000472524
238	6	0.000665230	-0.000367929	-0.000209204
239	6	0.000271631	0.001226549	0.000313177
240	6	-0.000395135	-0.002894216	-0.000797420
241	6	0.000151973	0.000874421	-0.000110320
242	6	0.000105977	-0.000560928	-0.000079178
243	6	-0.000754072	-0.000013763	-0.000198815
244	6	0.000167317	0.000094237	0.000506375
245	6	-0.000724323	-0.000645597	-0.000793522
246	1	-0.000197356	-0.000445110	-0.000048032
247	1	0.000639434	0.000378857	0.000108236
248	1	-0.000129162	-0.000018048	0.000103297
249	1	0.000169214	0.000180323	0.000350946
250	1	0.000284942	0.000091586	0.000155612
251	1	0.000258332	0.000366455	0.000141707
252	8	0.000171593	0.000078602	0.000325310
253	8	0.001300851	-0.001991819	0.001309709
254	6	-0.000123068	0.000227586	0.000200399
255	6	0.000585986	-0.000606233	0.000569080
256	6	-0.001517889	0.001781729	-0.000815407
257	6	0.000004250	-0.000448012	0.000257474
258	6	-0.000089350	0.000349567	-0.000158705
259	6	-0.000365789	-0.000285728	-0.000385993
260	6	0.000537453	0.000222823	-0.000068707
261	6	-0.001191087	-0.000081932	-0.000588282
262	1	-0.000202326	0.000305216	-0.000343422
263	1	0.000475142	-0.000210816	0.000792082
264	1	0.000066514	-0.000028290	-0.000169287
265	1	0.000454305	0.000054393	0.000093507
266	1	0.000215431	0.000110578	0.000244181
267	1	0.000359538	-0.000145664	0.000272441
268	8	-0.000236008	0.000085373	0.001254332
269	8	0.000140454	0.000777666	-0.000699720
270	6	0.000249653	0.000090334	-0.000535962
271	6	-0.000088051	-0.000012698	0.000198988
272	6	0.000276279	-0.000276183	0.000305019
273	6	-0.000591676	0.000002081	-0.000472271
274	6	-0.000318983	0.000161969	0.000065327
275	6	-0.000666467	0.000017139	-0.000176851
276	6	0.000222597	-0.000188667	0.000450954
277	6	-0.001177262	-0.000029047	-0.000596764
278	1	0.000057643	0.000005453	-0.000026468
279	1	0.000788120	-0.000149906	0.000269866
280	1	-0.000005943	0.000015074	-0.000036884
281	1	0.000313253	-0.000032189	0.000285511
282	1	0.000464790	-0.000028867	0.000035406
283	1	0.000284025	0.000148273	0.000165528
284	8	0.001134065	0.000436830	-0.000479598
285	8	-0.000495108	-0.000433998	0.000675745
286	6	-0.000438768	-0.000375041	0.000398027
287	6	-0.000028556	0.000285376	-0.000055286
288	6	0.000468586	0.000120613	-0.000108957
289	6	-0.000430373	-0.000262539	-0.000357757
290	6	0.000090447	-0.000300732	-0.000081085
291	6	-0.000200270	-0.000178081	-0.000477230
292	6	0.000433440	0.000204108	0.000085872
293	6	-0.000786721	-0.000500927	-0.000839229
294	1	0.000028756	-0.000138744	-0.000017460

295	1	0.000261453	0.000632527	0.000376463
296	1	0.000041609	-0.000079105	-0.000009769
297	1	0.000272044	0.000188720	0.000138233
298	1	0.000061836	0.000313682	0.000477670
299	1	0.000273054	0.000035346	0.000264955
300	1	0.000554146	-0.000006051	0.000387582
301	1	-0.000147645	0.002547855	0.001191410
302	1	-0.000060865	0.000099452	-0.001050910
303	1	-0.001605992	0.002292218	-0.001796258
304	1	-0.001018782	0.001149425	0.002396600
305	1	-0.000761332	-0.000849186	-0.001212059
306	1	-0.000825539	0.000834720	0.000440109
307	1	0.000689292	-0.000019643	0.001142647
308	1	0.001422779	0.000548021	-0.000306787
309	1	-0.001135998	-0.003147682	0.001350627
310	8	-0.000500629	0.000156477	-0.000427715
311	8	0.001064713	-0.002062840	-0.001787504
312	6	0.000000173	-0.000490125	0.000428907
313	6	0.000340668	-0.000841068	-0.000955378
314	6	-0.001052927	0.000728285	0.001225622
315	6	-0.000062442	0.000305219	-0.000374573
316	6	-0.000165149	-0.000005223	0.000213440
317	6	0.000058301	0.000748840	-0.000038842
318	6	0.000119617	-0.000566945	0.000246795
319	6	-0.000570762	0.001117945	0.000071479
320	1	-0.000177047	0.000579540	0.000487300
321	1	0.000036260	-0.000779588	-0.000292304
322	1	-0.000103190	0.000017701	0.000068366
323	1	0.000046918	-0.000370336	0.000004082
324	1	0.000267565	-0.000393395	-0.000268735
325	1	0.000223539	-0.000254289	0.000086190
326	8	0.000824268	-0.000387004	0.001415881
327	8	0.000598475	-0.001987981	0.002427402
328	6	-0.000225979	-0.000112971	-0.000458718
329	6	0.000570743	-0.001137315	0.001233154
330	6	0.000185799	0.000923113	-0.001635973
331	6	0.000051386	0.000311216	0.000536725
332	6	-0.000050705	0.000169469	-0.000118528
333	6	-0.000120076	0.000385135	0.000135875
334	6	-0.000235892	-0.000822783	-0.000305574
335	6	-0.000046268	0.001211884	-0.000261104
336	1	-0.000305275	0.000776128	-0.000490574
337	1	0.000382080	-0.000544103	0.000376605
338	1	0.000096203	0.000092172	-0.000105244
339	1	-0.000035969	-0.000422401	0.000334947
340	1	-0.000020392	-0.000393905	-0.000057970
341	1	0.000129309	-0.000278185	0.000042953
342	8	0.000522272	0.000636206	0.002163931
343	8	0.001158720	-0.000101761	-0.000479882
344	6	-0.000226978	-0.000258210	-0.001275239
345	6	0.000065850	0.000034841	0.000572150
346	6	-0.001046033	-0.000683073	-0.000392882
347	6	0.000351093	0.001015017	-0.000206688
348	6	-0.000114789	-0.000106206	-0.000609191
349	6	-0.000122894	0.000558001	0.000536691
350	6	0.000374948	-0.000349634	0.000357608
351	6	-0.000015301	0.001313805	-0.000246038
352	1	0.000120078	0.000110018	-0.000119358

353	1	-0.000199669	-0.000846361	0.000309924
354	1	-0.000082464	-0.000010944	0.000165779
355	1	0.000092981	-0.000336489	-0.000006698
356	1	0.000052133	-0.000417713	0.000193205
357	1	-0.000164366	-0.000476352	-0.000018199
358	8	-0.000857346	0.000104900	-0.001366206
359	8	-0.000894137	-0.000286328	0.000215633
360	6	0.000147279	-0.000071161	0.000514430
361	6	0.000320825	-0.000462402	-0.000098037
362	6	0.000732684	-0.000072439	0.000144932
363	6	-0.000308002	0.000521049	0.000258549
364	6	-0.000376864	0.000000494	0.000215016
365	6	-0.000242815	0.000534975	-0.000217192
366	6	0.000019149	-0.000156385	-0.000432876
367	6	-0.000828103	0.000994966	0.000223261
368	1	-0.000163140	0.000254395	-0.000079464
369	1	0.000548551	-0.000536790	-0.000226830
370	1	0.000089779	0.000166198	-0.000086207
371	1	0.000429445	-0.000256392	-0.000005648
372	1	0.000116098	-0.000254844	-0.000012446
373	1	0.000224560	-0.000336421	-0.000165625
374	1	0.000362357	0.000237803	-0.000350044
375	1	-0.000031443	0.000062477	0.001729377
376	1	0.000474484	-0.000330497	-0.000074916
377	1	0.002217194	-0.001968816	0.000591310
378	1	-0.000638706	0.000175201	0.000382742
379	1	0.000423778	0.000776474	0.000166365
380	1	0.000480642	-0.000458851	-0.000224543
381	1	0.001198064	0.000949444	0.000332712
382	1	0.000338133	-0.000786674	0.000042298
383	1	0.002791782	-0.000908986	-0.001486165
384	8	-0.000493579	-0.000275420	-0.000276386
385	8	-0.001218749	0.001090819	-0.001155063
386	6	-0.000291355	0.000931324	0.000851198
387	6	-0.000295838	0.000863039	-0.000139474
388	6	0.001996528	-0.000906714	0.000208575
389	6	0.000072008	-0.000349867	-0.000089621
390	6	-0.000222196	-0.000167105	-0.000259379
391	6	-0.000244018	-0.000605458	0.000069437
392	6	-0.000043349	0.000562319	0.000136136
393	6	0.000357148	-0.001354745	0.000112457
394	1	-0.000080036	-0.000054825	-0.000385263
395	1	0.000209972	0.000858759	0.000155582
396	1	-0.000022080	-0.000009922	0.000027118
397	1	0.000136243	0.000448470	-0.000155750
398	1	-0.000185914	0.000300697	-0.000085808
399	1	-0.000099896	0.000453546	0.000100843
400	8	-0.000678387	-0.000466649	-0.000007302
401	8	-0.002500305	0.001663494	0.000781329
402	6	0.000250998	0.000506360	-0.000272782
403	6	-0.001228030	0.000741922	0.000341901
404	6	0.001541320	-0.000815144	-0.001051093
405	6	-0.000593295	-0.000263024	0.000240907
406	6	0.000334310	0.000155184	-0.000131585
407	6	0.000019351	-0.000581156	0.000204639
408	6	0.000083640	0.000669564	0.000293740
409	6	0.000300319	-0.001297184	-0.000136545
410	1	0.000677349	-0.000454815	-0.000076677

411	1	-0.000559296	0.000415764	-0.000133288
412	1	0.000120959	0.000012014	-0.000095106
413	1	0.000010597	0.000417646	0.000031453
414	1	-0.000118255	0.000303800	-0.000075387
415	1	-0.000300374	0.000344484	0.000149206
416	8	0.000485944	0.000075797	-0.000433461
417	8	-0.000656438	0.000546155	0.001132438
418	6	0.000021744	0.000276239	0.000115612
419	6	-0.000517229	0.000800752	0.000516770
420	6	-0.000105967	-0.000371912	-0.000865818
421	6	-0.000088696	-0.000594280	0.000284870
422	6	0.000143246	-0.000233375	0.000046054
423	6	0.000119401	-0.000752725	0.000140003
424	6	0.000346400	0.000579002	-0.000193535
425	6	0.000260558	-0.001249867	0.000521347
426	1	0.000198929	-0.000236472	0.000039302
427	1	-0.000176256	0.000712791	-0.000369985
428	1	0.000146912	0.000059394	-0.000144156
429	1	-0.000229362	0.000317786	-0.000320359
430	1	-0.000122168	0.000313809	-0.000013379
431	1	-0.000011908	0.000447442	-0.000146990
432	8	-0.002338076	-0.001051356	-0.000203291
433	8	-0.000458420	-0.000033534	-0.000732089
434	6	0.001828324	0.000267275	-0.000154592
435	6	-0.000814716	0.000020778	0.000049152
436	6	0.000564163	0.000833114	0.001105979
437	6	0.000056329	-0.001177985	-0.000904600
438	6	0.000766580	-0.000036054	0.000208505
439	6	-0.000751443	-0.000315156	-0.000019392
440	6	-0.000084703	0.000386005	-0.000194584
441	6	0.000401899	-0.001380247	-0.000149125
442	1	0.000114373	-0.000164812	-0.000240092
443	1	-0.000310611	0.000642581	0.000386626
444	1	-0.000200138	-0.000000577	0.000181661
445	1	-0.000219107	0.000383435	0.000061623
446	1	0.000060793	0.000469307	0.000183588
447	1	-0.000079269	0.000424972	-0.000080648
448	1	0.000447130	0.000454850	-0.000807775
449	1	-0.001137617	0.000419997	0.000239226
450	1	0.000677263	-0.000994837	0.000741663
451	1	0.000498102	0.000183145	0.000569554
452	1	0.000537360	-0.001020665	-0.001021146
453	1	-0.001933469	-0.001789554	0.001470355
454	1	-0.000183775	-0.001209358	-0.000701971
455	1	0.000302849	0.000122685	-0.000112463
456	1	0.000571215	0.000575556	0.000082761
457	1	-0.000038869	0.000347853	-0.000499898
458	6	0.001337553	0.002114890	0.002518489
459	6	0.000905573	-0.000175982	-0.000014319
460	6	-0.000206946	0.000534525	-0.000375933
461	6	0.000039729	0.000130861	0.000471361
462	6	-0.000022991	0.000078380	0.000716882
463	6	-0.001282623	0.000591227	0.000246834
464	1	0.002489184	-0.002476022	0.005351759
465	1	-0.001259774	-0.006316882	0.001007130
466	1	-0.001941050	0.002827908	-0.005510420
467	1	0.002458568	0.005873462	-0.001492284
468	17	-0.000342084	-0.002772842	-0.000523904

469	1	0.000156679	-0.000276644	0.000111887
470	1	0.000572498	-0.000205832	-0.000418051
471	1	-0.000121480	-0.000392994	0.000030708
472	8	0.002319383	-0.001078606	-0.000540873
473	1	-0.000744050	0.000420837	0.000478787
474	1	-0.001278351	0.000397226	-0.000158772
475	8	-0.000799720	0.001769716	0.001319421
476	1	0.000013498	-0.000880026	-0.000545360
477	1	0.000725636	-0.000753448	-0.000431410
478	8	-0.002081627	-0.001802274	0.001003817
479	1	0.000513522	0.000887936	-0.000820989
480	1	0.000830601	0.000456360	-0.000477231
481	6	0.000484438	0.000039478	0.001576118
482	8	0.000760461	-0.004839275	0.003863716
483	1	-0.003224830	-0.003502739	-0.004489735

Sum of electronic and thermal Free Energies= -1284.418636
1 imaginary frequency: -604.80

β-TS2$_{shift}$

Center Number	Atomic Number	Integrated Forces (Hartrees/Bohr) X	Y	Z
1	6	-0.000380812	-0.001089778	0.000109969
2	6	-0.000748595	0.001603700	-0.002007726
3	7	0.004858606	0.000328538	-0.002882892
4	6	0.000043328	0.001912421	0.001983887
5	6	-0.002314221	0.002419222	0.000654209
6	6	-0.004865764	-0.000087417	0.003956712
7	1	-0.001122988	-0.001221853	0.006518937
8	1	0.006552922	0.000118943	0.002299699
9	1	-0.003812796	0.002655450	-0.005649025
10	1	-0.000369200	-0.003849380	-0.000574025
11	1	0.006471788	-0.000888411	0.002024614
12	1	-0.001498083	0.005571223	-0.001028820
13	1	-0.000173254	-0.003681816	-0.005877085
14	8	0.000093961	-0.001342844	0.000990408
15	8	0.004241348	0.002221779	-0.004933421
16	6	0.000440077	0.000657874	-0.000711550
17	6	-0.000762731	-0.000572204	0.000622118
18	6	-0.001167715	-0.001694275	0.003363378
19	6	0.000313889	0.001651828	0.000867660
20	6	0.000650290	0.000259704	-0.000750340
21	6	-0.000515279	-0.000108181	0.000738263
22	6	0.000036775	-0.000607166	0.000371120
23	6	0.000279339	0.001587488	0.000436671
24	1	0.000309708	0.000325124	-0.000032497
25	1	-0.000485079	-0.000798959	-0.000301693
26	1	-0.000177415	-0.000155788	-0.000351562
27	1	-0.000169150	-0.000433064	-0.000337781
28	1	-0.000005548	-0.000468073	-0.000062004
29	1	-0.000061573	-0.000501295	-0.000016412
30	8	0.000636382	-0.000543278	0.000096786
31	8	0.001095453	-0.002784346	0.002053248
32	6	0.000012314	-0.000050423	-0.000256707
33	6	0.000550957	-0.000950931	0.000722770

34	6	-0.000409087	0.001494666	-0.001840237
35	6	0.000345036	-0.000201914	0.000823121
36	6	-0.000133971	0.000016191	-0.000668574
37	6	-0.000125941	0.000821753	0.000442816
38	6	0.000028074	-0.000506009	-0.000217519
39	6	-0.000331016	0.001201326	0.000246917
40	1	-0.000352577	0.000443463	-0.000329096
41	1	0.000428275	-0.000622298	-0.000005720
42	1	0.000098571	0.000095776	-0.000146371
43	1	0.000162629	-0.000425492	0.000177243
44	1	0.000001931	-0.000366538	-0.000111901
45	1	0.000153025	-0.000348384	-0.000161248
46	8	0.001339054	-0.000362804	0.000444190
47	8	0.002661138	-0.000172871	0.000228257
48	6	-0.000943339	-0.001943572	-0.000932311
49	6	0.001358670	-0.000028245	-0.001050368
50	6	-0.002336500	0.000343986	0.000221473
51	6	0.001083127	0.000014976	0.000047039
52	6	-0.001252859	-0.000019442	-0.000308236
53	6	0.000340747	0.001979459	0.001046618
54	6	-0.000369536	-0.000630239	-0.000535228
55	6	-0.000085390	0.001365635	0.000486031
56	1	-0.000480416	0.000383640	0.000705207
57	1	0.000226012	-0.000760192	-0.000575485
58	1	-0.000037480	0.000016705	0.000112859
59	1	-0.000065009	-0.000450885	-0.000088050
60	1	0.000292397	-0.000379373	-0.000260637
61	1	0.000038262	-0.000358401	0.000003905
62	8	0.001799699	-0.000101610	0.002032018
63	8	0.000112004	-0.000189609	0.000477993
64	6	-0.001181441	0.000672426	-0.001612680
65	6	0.000197044	-0.000264646	0.000870205
66	6	-0.000077690	-0.000306423	-0.000336262
67	6	0.000548690	0.000926834	0.000322139
68	6	-0.000528584	0.000077553	-0.000438500
69	6	0.000312776	-0.000099370	0.000694551
70	6	-0.000130998	-0.000342556	-0.000866063
71	6	0.000462266	0.001375041	0.000721753
72	1	0.000219107	0.000230211	-0.000309673
73	1	-0.000130951	-0.000724324	0.000163630
74	1	-0.000125259	0.000023294	-0.000095252
75	1	-0.000043994	-0.000362992	-0.000109049
76	1	-0.000329478	-0.000450211	-0.000102475
77	1	-0.000028077	-0.000467202	-0.000271179
78	8	-0.000232625	0.000070814	-0.000248156
79	8	0.000655359	0.002538800	-0.001836317
80	6	0.000570421	0.000308812	0.000544987
81	6	0.000242750	0.000629827	-0.000925036
82	6	0.000063008	-0.001166393	0.001702408
83	6	0.000114019	-0.000052397	-0.000549828
84	6	0.000003776	-0.000135593	0.000413626
85	6	-0.000291705	-0.000654337	-0.000122232
86	6	-0.000107802	0.000474430	0.000245799
87	6	-0.000220698	-0.001218607	-0.000041832
88	1	-0.000238043	-0.000290075	0.000425145
89	1	0.000364006	0.000687918	-0.000161473
90	1	0.000133592	-0.000080993	0.000109181
91	1	0.000143472	0.000351596	0.000102237

92	1	0.000153252	0.000406835	-0.000200274
93	1	-0.000019642	0.000328765	0.000047569
94	8	0.000795536	-0.000103693	0.000297610
95	8	-0.000706754	0.001930620	0.001835143
96	6	-0.000897576	0.000390579	-0.000691311
97	6	-0.000135166	0.000474631	0.000856329
98	6	-0.000025784	-0.000810832	-0.001522338
99	6	-0.000016968	-0.000202218	0.000446840
100	6	0.000037377	-0.000061604	-0.000469368
101	6	0.000469811	-0.000633946	0.000004372
102	6	-0.000047057	0.000507222	-0.000233803
103	6	0.000455962	-0.001184610	-0.000099708
104	1	0.000198061	-0.000194641	-0.000412827
105	1	-0.000456877	0.000590770	0.000315648
106	1	-0.000103749	-0.000067022	-0.000112271
107	1	-0.000189975	0.000328243	-0.000059747
108	1	-0.000249578	0.000401525	0.000268871
109	1	-0.000046440	0.000324302	0.000006244
110	8	-0.001076719	-0.000457670	0.001375976
111	8	0.000028269	-0.000661237	0.000129305
112	6	0.000558510	0.000375874	-0.001062778
113	6	-0.000022150	-0.000240430	0.000960722
114	6	0.000413768	0.000984338	-0.000302982
115	6	-0.000208875	-0.000947567	-0.000061821
116	6	0.000097683	-0.000082759	-0.000246050
117	6	0.000021840	-0.000603190	0.000262118
118	6	-0.000232567	0.000462139	-0.000063563
119	6	-0.000343453	-0.001268696	-0.000060578
120	1	-0.000157825	-0.000081051	-0.000385878
121	1	0.000089278	0.000819242	0.000342666
122	1	0.000093324	-0.000020525	-0.000074275
123	1	0.000072083	0.000379595	-0.000085900
124	1	0.000033985	0.000359873	0.000031242
125	1	0.000320467	0.000402242	0.000102617
126	8	0.001547280	0.000008385	-0.001781826
127	8	0.000551071	-0.000730884	-0.000154596
128	6	-0.001051025	-0.000222952	0.001284068
129	6	0.000407691	-0.000063786	-0.000885328
130	6	-0.001074071	0.001035780	0.000397784
131	6	0.000534678	-0.001121705	0.000040078
132	6	-0.000308740	-0.000209947	0.000261742
133	6	0.000220259	-0.000367971	-0.000355067
134	6	0.000152818	0.000459001	0.000197843
135	6	0.000574581	-0.001212046	-0.000153804
136	1	0.000114098	-0.000025924	0.000347973
137	1	-0.000201679	0.000857640	-0.000317042
138	1	-0.000101611	-0.000008283	0.000064020
139	1	-0.000144648	0.000337431	0.000139412
140	1	-0.000097685	0.000354699	0.000017730
141	1	-0.000400505	0.000401301	-0.000033917
142	8	0.001409031	0.001294620	0.000971865
143	1	-0.000525486	-0.000876601	-0.000068924
144	1	-0.000618850	-0.000162667	-0.000925875
145	8	-0.000124353	0.000323610	-0.000661464
146	1	-0.000059164	-0.000007013	-0.000980979
147	1	-0.000945490	0.000651851	0.000130147
148	8	-0.006219910	0.003306278	0.004619463
149	1	0.003005891	-0.002733043	-0.004500924

150	1	0.004270131	-0.000205196	0.001052307
151	8	0.000111384	-0.000589340	0.000461907
152	1	-0.000871417	-0.000658960	-0.000021854
153	1	-0.000135138	0.000048547	0.000969087
154	8	-0.003470233	-0.005460804	-0.008042496
155	1	0.003504036	0.004280776	0.003535502
156	1	0.000979193	0.002234656	0.006004366
157	1	-0.001126696	0.002708214	0.002042083
158	1	0.000429086	0.000466327	0.000458203
159	1	-0.000074944	0.000204444	-0.000071910
160	1	-0.001097975	0.000304073	0.000297065
161	1	0.000112483	0.000015219	0.000559332
162	8	0.000627498	0.000250361	0.000004375
163	8	0.002759050	-0.000170586	0.001252831
164	6	-0.000754662	-0.000864388	0.000460523
165	6	0.001169273	0.000005201	0.000824684
166	6	-0.002773434	0.000004434	-0.001727905
167	6	0.001070858	-0.000321919	0.000550181
168	6	-0.000945646	-0.000374660	-0.000038910
169	6	0.000400224	0.000586991	-0.000960531
170	6	-0.000024059	0.000202485	0.000437382
171	6	-0.000280998	-0.000090392	-0.001044008
172	1	-0.000400474	0.000130924	-0.000304873
173	1	0.000116368	-0.000357037	0.000632736
174	1	-0.000024123	0.000165298	-0.000032069
175	1	-0.000010654	-0.000042529	0.000277724
176	1	0.000302085	-0.000055865	0.000352195
177	1	0.000064981	0.000123394	0.000343443
178	8	0.001233272	-0.002926054	-0.001781943
179	8	0.001223213	-0.000619679	-0.000877992
180	6	-0.000169778	0.002060099	0.000349578
181	6	0.001274706	-0.002136597	0.000022219
182	6	-0.002145396	0.000989879	0.001172514
183	6	0.000945416	-0.000654670	-0.001340075
184	6	-0.000553641	-0.000288061	-0.000362892
185	6	0.000821401	-0.000614493	-0.000281099
186	6	0.000300269	0.000044451	0.000471260
187	6	0.000385458	-0.000149524	-0.001409536
188	1	-0.000263914	0.000681564	0.000313107
189	1	-0.000224540	0.000410052	0.000813046
190	1	-0.000010584	-0.000032755	0.000022330
191	1	-0.000005765	0.000097914	0.000436589
192	1	-0.000374777	0.000127518	0.000429048
193	1	-0.000073994	-0.000081716	0.000390347
194	8	0.000201546	-0.001051504	0.000645347
195	8	-0.000000439	-0.001056488	0.000067168
196	6	-0.000051890	0.000470308	-0.000725285
197	6	-0.000390216	-0.000768500	0.001057288
198	6	0.000409463	0.001064530	-0.000140533
199	6	-0.000021515	-0.000607369	-0.000424917
200	6	-0.000283136	0.000432915	-0.000101363
201	6	0.000239157	-0.000401827	0.000111790
202	6	-0.000849628	0.000044176	0.000331173
203	6	0.000420312	-0.000304095	-0.001203502
204	1	0.000142314	0.000186679	-0.000373532
205	1	0.000311479	0.000125515	0.000520675
206	1	-0.000131793	0.000053263	0.000063824
207	1	0.000016996	0.000328764	0.000413131

208	1	-0.000200028	0.000020003	0.000455037
209	1	0.000014989	-0.000026621	0.000251618
210	8	0.000973936	-0.000624831	-0.001141945
211	8	0.000873024	-0.001188209	-0.000236166
212	6	-0.001805609	0.000749624	0.001152509
213	6	0.000448399	-0.000610690	-0.000008586
214	6	-0.000295723	0.000853272	0.000008903
215	6	0.000415706	-0.000625412	-0.000230030
216	6	-0.000258900	0.000841150	-0.000101262
217	6	0.001017087	-0.000121630	-0.001170816
218	6	-0.000592329	0.000355465	0.000507847
219	6	0.000542627	0.000139181	-0.001212204
220	1	0.000024526	0.000174075	0.000102607
221	1	-0.000517649	-0.000247102	0.000741631
222	1	0.000066018	0.000036469	0.000005455
223	1	-0.000209523	-0.000287922	0.000399623
224	1	-0.000009206	-0.000046961	0.000305044
225	1	-0.000153416	0.000066790	0.000373493
226	1	-0.000740876	0.000847180	0.000500407
227	1	-0.000007540	0.000046127	-0.000355580
228	1	-0.000332889	-0.000601143	-0.000085840
229	1	0.000052480	0.000303489	0.000263945
230	1	-0.000759369	-0.000294927	-0.000876832
231	1	-0.000007671	0.000162566	0.000171477
232	1	-0.000232370	-0.000434217	-0.000408377
233	1	0.000018162	-0.000219687	0.000192371
234	1	0.000334953	0.000727146	-0.000107685
235	1	0.000114099	0.001193007	0.000270387
236	8	0.000367991	-0.000582506	0.000205514
237	8	0.002466667	0.000360083	-0.001199475
238	6	-0.000773408	0.000924524	-0.000490946
239	6	0.001121072	-0.000014150	-0.000479363
240	6	-0.002450457	-0.000162413	0.001288357
241	6	0.000702716	0.000291127	-0.000057300
242	6	-0.000625432	0.000077386	0.000115682
243	6	0.000253290	-0.000521908	0.000849847
244	6	-0.000103682	-0.000180360	-0.000511770
245	6	-0.000275020	-0.000096101	0.001206641
246	1	-0.000416378	-0.000083322	0.000191228
247	1	0.000277487	0.000552092	-0.000780173
248	1	0.000022527	-0.000134135	0.000025719
249	1	0.000072258	-0.000083196	-0.000386181
250	1	-0.000031054	0.000071841	-0.000314916
251	1	0.000286116	0.000170016	-0.000319207
252	8	-0.000002307	-0.000069372	-0.000458251
253	8	-0.002101651	-0.000520020	-0.001293078
254	6	0.000364652	-0.000172276	-0.000183491
255	6	-0.000902049	-0.000258313	-0.000685673
256	6	0.002136681	0.000068605	0.001267813
257	6	-0.000511164	-0.000346825	-0.000101976
258	6	0.000491618	-0.000017332	0.000057576
259	6	-0.000196635	0.000128839	0.000640089
260	6	0.000000770	0.000461933	-0.000440263
261	6	0.000356680	-0.000285555	0.001183901
262	1	0.000376821	0.000207395	0.000306447
263	1	-0.000204035	-0.000331226	-0.000772856
264	1	-0.000041499	0.000144524	0.000057499
265	1	-0.000075484	0.000164372	-0.000368309

266	1	0.000012429	0.000014779	-0.000314522
267	1	-0.000317982	-0.000026717	-0.000400663
268	8	-0.000097282	-0.001119584	-0.000794292
269	8	0.000350084	-0.000209620	0.000540408
270	6	0.000285889	0.000736321	0.000242375
271	6	-0.000293843	-0.000394089	-0.000027922
272	6	-0.000161837	0.000708399	-0.000774952
273	6	0.000303921	-0.000350823	0.000826042
274	6	0.000289397	0.000177859	0.000153571
275	6	-0.000030242	-0.000351823	0.000379706
276	6	-0.000062260	-0.000242439	-0.000475525
277	6	0.000257528	-0.000339093	0.001221369
278	1	0.000158705	0.000092677	0.000027589
279	1	-0.000418935	0.000260959	-0.000639159
280	1	0.000052669	0.000057598	-0.000020503
281	1	-0.000095154	-0.000018451	-0.000356905
282	1	-0.000229471	0.000279164	-0.000334397
283	1	0.000030303	0.000087175	-0.000344326
284	8	0.000061792	0.001047103	-0.000614864
285	8	-0.000529941	-0.000728031	-0.000105845
286	6	-0.000138893	-0.000567628	0.000080266
287	6	0.000212588	0.000140461	-0.000092690
288	6	0.000109342	0.000202875	-0.000321205
289	6	-0.000217343	-0.000092276	0.000644077
290	6	-0.000175438	0.000047602	0.000152025
291	6	-0.000155399	0.000089717	0.000443217
292	6	0.000116691	0.000300289	-0.000423141
293	6	-0.000281510	0.000062168	0.001226971
294	1	-0.000111026	0.000018175	0.000004153
295	1	0.000432372	-0.000143809	-0.000691264
296	1	-0.000059018	-0.000042771	0.000016321
297	1	0.000105665	0.000096384	-0.000347519
298	1	0.000164626	-0.000241529	-0.000422615
299	1	-0.000039365	-0.000021830	-0.000348690
300	1	-0.000331566	-0.000045196	-0.000644537
301	1	0.002233485	-0.000295793	-0.001498459
302	1	-0.000163909	0.000607406	0.000907305
303	1	0.002503583	0.000809284	0.001713425
304	1	0.001242084	-0.002170886	-0.000924878
305	1	-0.000626509	0.000451515	0.001267590
306	1	0.000996078	-0.000946801	0.000432281
307	1	-0.000525508	-0.000417881	-0.001054286
308	1	0.000684887	0.001562211	-0.000735699
309	1	-0.002492138	-0.003162534	0.001868708
310	8	0.000502239	-0.000103964	0.000696651
311	8	-0.001967914	0.001158768	0.001043474
312	6	-0.000429143	-0.000202258	-0.000302285
313	6	-0.000780950	0.000624866	0.000610388
314	6	0.000818903	-0.001042221	-0.000191902
315	6	0.000307515	0.000281736	0.000117830
316	6	0.000049057	-0.000161214	-0.000187386
317	6	0.000568608	0.000146546	0.000068147
318	6	-0.000511799	-0.000138129	-0.000283328
319	6	0.001270128	-0.000144997	-0.000062143
320	1	0.000451477	-0.000365771	-0.000334170
321	1	-0.000759917	0.000196570	0.000519361
322	1	0.000051244	-0.000093960	0.000068980
323	1	-0.000346877	-0.000059363	0.000077331

324	1	-0.000437173	0.000303444	0.000147634
325	1	-0.000365162	0.000002023	-0.000098178
326	8	0.000084109	0.000027147	-0.001068878
327	8	-0.002049100	-0.001809318	-0.001316036
328	6	-0.000101573	-0.000024207	0.000655351
329	6	-0.000923142	-0.000790690	-0.000757116
330	6	0.000878521	0.001458681	0.000506825
331	6	0.000133030	-0.000313383	-0.000328728
332	6	-0.000003546	0.000257218	0.000081463
333	6	0.000442460	-0.000192111	-0.000229844
334	6	-0.000539127	-0.000005462	0.000388096
335	6	0.001206566	0.000270921	-0.000081676
336	1	0.000529393	0.000452329	0.000397648
337	1	-0.000651194	-0.000143038	-0.000284021
338	1	0.000042216	0.000162455	-0.000032754
339	1	-0.000341979	-0.000344734	-0.000146078
340	1	-0.000343108	-0.000020770	0.000110361
341	1	-0.000334196	0.000027197	-0.000051977
342	8	0.000680838	-0.001202254	-0.001959809
343	8	-0.000349944	0.001014501	-0.000753850
344	6	-0.000192062	0.001025904	0.001215345
345	6	0.000075459	-0.000367805	-0.000533209
346	6	-0.000483760	-0.000498402	0.001240440
347	6	0.000991038	0.000515509	-0.000485104
348	6	0.000052244	0.000405843	0.000488130
349	6	0.000518579	-0.000420591	-0.000470810
350	6	-0.000474891	-0.000058937	-0.000261948
351	6	0.001230398	0.000452538	-0.000202441
352	1	-0.000063188	0.000150520	-0.000077760
353	1	-0.000725946	-0.000545319	0.000192708
354	1	0.000022837	-0.000220180	-0.000028131
355	1	-0.000339252	-0.000004531	0.000018781
356	1	-0.000377024	-0.000192841	-0.000008212
357	1	-0.000350928	-0.000264410	0.000245750
358	8	0.000806987	0.000560613	0.001340634
359	8	-0.000422267	-0.001549677	0.000464401
360	6	-0.000449298	-0.000416158	-0.000715311
361	6	-0.000002687	-0.000077480	0.000248219
362	6	-0.000213958	0.000502487	-0.000764181
363	6	0.000693755	-0.000463353	0.000163781
364	6	0.000163035	-0.000216134	-0.000021595
365	6	0.000676271	-0.000016239	0.000204435
366	6	-0.000441914	0.000315540	0.000405736
367	6	0.001250136	-0.000540434	0.000037208
368	1	-0.000098253	0.000070154	0.000078197
369	1	-0.000734584	0.000401817	-0.000230333
370	1	-0.000008684	0.000254218	0.000071889
371	1	-0.000439869	0.000224690	-0.000241249
372	1	-0.000302238	0.000018912	0.000011687
373	1	-0.000429002	0.000178871	0.000059947
374	1	0.000420668	0.000494903	0.000714330
375	1	0.000306403	0.000541592	-0.000590375
376	1	-0.000784842	0.000603420	-0.000227959
377	1	-0.002653990	0.000679442	-0.001518237
378	1	0.000367597	-0.000208324	0.000429185
379	1	0.000599544	0.000413815	-0.000802959
380	1	-0.000469933	0.000431225	0.000057479
381	1	0.000405085	0.000596147	-0.001309919

382	1	-0.001098311	-0.000597165	-0.000291104
383	1	-0.001660867	0.002679994	-0.000824135
384	8	-0.000639319	-0.000657962	-0.001172289
385	8	-0.002826605	0.001866459	0.000320955
386	6	0.000387127	0.000488044	0.000439789
387	6	0.000101046	-0.000623731	0.000037780
388	6	0.002860051	0.001550272	0.001036015
389	6	-0.001201380	-0.000492371	0.000112302
390	6	-0.000207189	0.000324052	0.000272298
391	6	-0.000492071	-0.000344113	-0.000093050
392	6	0.000565357	-0.000230871	0.000083307
393	6	-0.001394797	-0.000359618	0.000007635
394	1	-0.000054662	-0.000091823	-0.000074939
395	1	0.000826651	0.000263552	-0.000391422
396	1	0.000042741	-0.000056823	-0.000041614
397	1	0.000384502	0.000366257	-0.000090689
398	1	0.000375476	0.000028665	0.000108460
399	1	0.000405144	0.000011611	-0.000026185
400	8	-0.000238727	-0.000179868	0.000919250
401	8	0.002409426	-0.001810067	0.001003134
402	6	0.000209963	0.000306777	-0.000429984
403	6	0.000922298	-0.000897711	0.000585114
404	6	-0.001122692	0.001565025	-0.000184814
405	6	-0.000103609	-0.000398813	0.000279961
406	6	-0.000071471	0.000247645	0.000022178
407	6	-0.000406685	-0.000345160	0.000015505
408	6	0.000555572	0.000082952	-0.000461052
409	6	-0.001281397	0.000027213	0.000294189
410	1	-0.000568295	0.000384068	-0.000298392
411	1	0.000659305	-0.000099702	0.000246398
412	1	-0.000044899	0.000130486	0.000038904
413	1	0.000359172	0.000066285	-0.000179829
414	1	0.000340340	0.000068104	0.000000621
415	1	0.000400748	-0.000229406	-0.000018321
416	8	-0.000318607	-0.001178539	-0.000192475
417	8	0.000819127	-0.001171047	-0.000087212
418	6	-0.000682028	-0.000350802	-0.001166392
419	6	0.000611118	-0.000779984	0.000138231
420	6	-0.000380510	0.000486725	0.000302167
421	6	-0.000083018	-0.000487766	0.000297415
422	6	-0.000085152	0.000057389	-0.000639805
423	6	-0.000916104	-0.000939228	-0.000340562
424	6	0.000337087	0.000611568	-0.000390452
425	6	-0.001171091	-0.000862142	0.000034845
426	1	-0.000334827	0.000276929	-0.000036557
427	1	0.000622581	0.000564242	0.000239718
428	1	-0.000048695	0.000201338	-0.000063055
429	1	0.000414033	0.000307327	0.000198042
430	1	0.000312806	0.000103857	-0.000030082
431	1	0.000396769	0.000270024	-0.000108052
432	8	0.000117742	-0.001550726	0.002117356
433	8	0.000290490	0.000489248	0.001031638
434	6	-0.000489714	0.001402124	-0.001349690
435	6	0.000446943	-0.000527383	0.000570143
436	6	0.000521851	-0.000209078	-0.001539727
437	6	-0.001079670	0.000246000	0.000742827
438	6	-0.000124071	0.000494447	-0.000695140
439	6	-0.000106325	-0.000645736	0.000592156

440	6	0.000511278	0.000278474	0.000189068
441	6	-0.001394195	0.000074468	0.000219969
442	1	-0.000302654	0.000245489	0.000078176
443	1	0.000726540	-0.000382873	-0.000229552
444	1	0.000005297	-0.000202923	0.000059585
445	1	0.000415382	-0.000094749	0.000013767
446	1	0.000437166	-0.000070348	-0.000287460
447	1	0.000404578	0.000060355	0.000008255
448	1	0.000096938	0.000925069	0.000108949
449	1	-0.000124251	0.000068921	0.000411384
450	1	-0.001023509	-0.000560977	-0.000521856
451	1	-0.000326710	0.000240486	-0.000970052
452	1	-0.000678077	0.000836333	0.000408513
453	1	-0.001140372	-0.002600614	0.000855360
454	1	-0.000848483	0.000227925	0.000931548
455	1	-0.000267249	0.000127964	-0.000073354
456	1	0.000007314	0.000606836	-0.000786731
457	1	0.000337738	0.000420389	0.000023175
458	6	0.002154520	-0.001079384	-0.002047155
459	6	-0.000504645	0.000084885	-0.000348488
460	6	0.001832484	-0.000583282	0.000159837
461	6	-0.000093672	-0.000216279	-0.000034501
462	6	0.000117101	-0.000377466	-0.000288999
463	6	0.001008477	0.000290774	0.000611378
464	1	-0.002650734	-0.003803475	-0.004519409
465	1	-0.005520823	-0.003250429	0.001824666
466	1	0.002608400	0.004218709	0.004027912
467	1	0.004737499	0.004012522	-0.002612272
468	17	-0.000621428	-0.003934175	-0.002366100
469	1	-0.000213064	0.000281990	0.000017116
470	1	-0.000380010	0.000664527	0.000121831
471	1	-0.000320683	-0.000032776	0.000227340
472	8	-0.001759865	0.001610895	-0.000893237
473	1	0.000733461	-0.000919729	0.000189592
474	1	0.000717471	-0.000309729	0.000724118
475	8	0.001022301	-0.001798270	-0.000659169
476	1	-0.000346658	0.000458028	0.000553986
477	1	-0.000578951	0.001118167	0.000018170
478	8	-0.001357842	-0.002361166	0.000509241
479	1	0.000934104	0.001305078	0.000114877
480	1	0.000033527	0.000850469	-0.000050337
481	6	-0.002634833	0.003112886	0.002000504
482	8	-0.001575742	-0.001447912	-0.004814724
483	1	-0.002663106	0.000537751	0.005986592

Sum of electronic and thermal Free Energies= -1284.398006
1 imaginary frequency: -1010.46

β-I2

Center Number	Atomic Number	Integrated Forces (Hartrees/Bohr) X	Y	Z
1	6	-0.010617902	-0.001161973	-0.003606290
2	6	0.005723124	-0.000836702	-0.007343147
3	7	-0.000388402	0.000679589	0.010744315
4	6	-0.002547607	0.000837943	-0.002861391

5	6	0.006808656	0.005309801	0.006362287
6	6	0.005709850	0.000110570	-0.004738458
7	1	0.001405652	0.000264047	-0.006579006
8	1	-0.006612585	0.000943120	-0.002634420
9	1	0.002645777	0.004295835	0.003431220
10	1	0.001181023	-0.006118705	0.002359248
11	1	-0.006603280	0.000279541	-0.002388106
12	1	0.000865882	0.005079564	0.003261892
13	1	-0.000437662	-0.004825711	0.004980493
14	8	-0.000299240	-0.000983075	-0.000994934
15	8	-0.004851884	0.001529582	0.004910558
16	6	-0.000312421	0.000418227	0.000626828
17	6	0.000428692	-0.000504278	-0.000395064
18	6	0.001602797	-0.001054520	-0.003384732
19	6	-0.000477264	0.001466563	-0.000817596
20	6	-0.000648710	0.000162691	0.000678089
21	6	0.000350078	0.000137137	-0.000674839
22	6	0.000033331	-0.000536857	-0.000465679
23	6	-0.000302488	0.001614985	-0.000252762
24	1	-0.000255610	0.000103181	0.000017940
25	1	0.000434337	-0.000850111	0.000189279
26	1	0.000165873	-0.000164680	0.000337687
27	1	0.000162822	-0.000445098	0.000277481
28	1	-0.000017199	-0.000473964	0.000018411
29	1	0.000105772	-0.000524859	-0.000031036
30	8	-0.000742185	-0.000495224	-0.000124102
31	8	-0.001213064	-0.002508117	-0.002264784
32	6	0.000062205	-0.000065444	0.000224766
33	6	-0.000663366	-0.000822799	-0.000857762
34	6	0.000396202	0.001363851	0.001835918
35	6	-0.000262091	-0.000107947	-0.000663050
36	6	0.000036538	-0.000097325	0.000637517
37	6	0.000091238	0.000698472	-0.000337853
38	6	0.000021600	-0.000554623	0.000153187
39	6	0.000329296	0.001208272	-0.000098828
40	1	0.000452600	0.000380352	0.000372921
41	1	-0.000415524	-0.000611098	-0.000081830
42	1	-0.000072987	0.000097342	0.000167455
43	1	-0.000212088	-0.000377163	-0.000208115
44	1	-0.000011797	-0.000370799	0.000075710
45	1	-0.000166042	-0.000325467	0.000103178
46	8	-0.001352786	-0.000470494	-0.000438436
47	8	-0.002636629	-0.000129190	-0.000188942
48	6	0.001227109	-0.002162600	0.000726720
49	6	-0.001650748	-0.000178017	0.000894270
50	6	0.002452661	0.000380449	-0.000267219
51	6	-0.001074738	0.000017521	0.000029789
52	6	0.001194377	-0.000084961	0.000340640
53	6	-0.000321248	0.002054767	-0.000842647
54	6	0.000297442	-0.000678440	0.000423367
55	6	0.000166801	0.001369716	-0.000301792
56	1	0.000581396	0.000515368	-0.000622388
57	1	-0.000277828	-0.000834090	0.000498121
58	1	0.000041856	0.000036783	-0.000114684
59	1	0.000030917	-0.000438802	0.000055539
60	1	-0.000275927	-0.000337946	0.000197770
61	1	-0.000060069	-0.000341967	-0.000047632
62	8	-0.001908232	0.000311337	-0.002103956

63	8	-0.000435418	0.000016237	-0.000543660
64	6	0.001270858	0.000465421	0.001623837
65	6	-0.000231522	-0.000147927	-0.000790218
66	6	0.000272858	-0.000514100	0.000213948
67	6	-0.000443727	0.001145078	-0.000147766
68	6	0.000673465	-0.000044927	0.000496741
69	6	-0.000404285	-0.000013175	-0.000694211
70	6	0.000066592	-0.000428148	0.000817629
71	6	-0.000410822	0.001473669	-0.000587534
72	1	-0.000136861	0.000186880	0.000306477
73	1	0.000094825	-0.000790637	-0.000274534
74	1	0.000157062	-0.000013517	0.000101582
75	1	0.000031277	-0.000376396	0.000067168
76	1	0.000336064	-0.000471165	0.000057469
77	1	0.000043182	-0.000517798	0.000240465
78	8	0.000091804	0.000047957	0.000271308
79	8	-0.000635895	0.002424463	0.002123173
80	6	-0.000486630	0.000397429	-0.000525448
81	6	-0.000252111	0.000580563	0.001011181
82	6	-0.000021430	-0.001025767	-0.001901864
83	6	-0.000173275	-0.000081138	0.000572834
84	6	0.000071423	-0.000047276	-0.000421786
85	6	0.000201770	-0.000722239	0.000078545
86	6	0.000182447	0.000492898	-0.000175418
87	6	0.000107985	-0.001231699	-0.000062768
88	1	0.000297388	-0.000316956	-0.000484660
89	1	-0.000317294	0.000721282	0.000171205
90	1	-0.000129637	-0.000062336	-0.000124942
91	1	-0.000096894	0.000365218	-0.000069183
92	1	-0.000113381	0.000405898	0.000266138
93	1	0.000053184	0.000337877	-0.000016001
94	8	-0.000890252	-0.000037664	-0.000404788
95	8	0.000804938	0.002166677	-0.001627624
96	6	0.001006748	0.000276278	0.000804976
97	6	0.000133147	0.000598117	-0.000796900
98	6	-0.000029294	-0.000967014	0.001438061
99	6	0.000003608	-0.000126679	-0.000499908
100	6	-0.000096724	-0.000042728	0.000462433
101	6	-0.000431336	-0.000708407	-0.000096000
102	6	0.000087797	0.000464968	0.000289320
103	6	-0.000530677	-0.001175662	-0.000048099
104	1	-0.000212223	-0.000303414	0.000363828
105	1	0.000532424	0.000570325	-0.000124335
106	1	0.000106701	-0.000084912	0.000112013
107	1	0.000199883	0.000328152	0.000104604
108	1	0.000237912	0.000419102	-0.000266217
109	1	0.000061303	0.000344175	0.000034691
110	8	0.001026001	-0.000407207	-0.001395830
111	8	-0.000128099	-0.000588373	-0.000206755
112	6	-0.000558604	0.000258619	0.001048886
113	6	0.000015545	-0.000106876	-0.000980390
114	6	-0.000295158	0.000934219	0.000405310
115	6	0.000109943	-0.000900694	0.000009212
116	6	-0.000057983	-0.000137006	0.000194621
117	6	-0.000081442	-0.000600367	-0.000292182
118	6	0.000240253	0.000436466	0.000098645
119	6	0.000269694	-0.001290953	-0.000067612
120	1	0.000166471	-0.000076547	0.000468383

121	1	-0.000079321	0.000904593	-0.000288951
122	1	-0.000104133	-0.000022653	0.000053825
123	1	-0.000046550	0.000378597	0.000119215
124	1	-0.000007797	0.000369516	-0.000003206
125	1	-0.000264203	0.000407168	-0.000048857
126	8	-0.001745076	-0.000039411	0.001892638
127	8	-0.000674592	-0.000805185	0.000096885
128	6	0.001150635	-0.000166561	-0.001501775
129	6	-0.000522821	-0.000198128	0.000929409
130	6	0.001247356	0.001097270	-0.000302367
131	6	-0.000639826	-0.001094453	-0.000105205
132	6	0.000318194	-0.000150297	-0.000351404
133	6	-0.000324912	-0.000384328	0.000374403
134	6	-0.000125772	0.000499656	-0.000154780
135	6	-0.000626594	-0.001213370	0.000018608
136	1	-0.000066683	0.000063794	-0.000385953
137	1	0.000218263	0.000794282	0.000369579
138	1	0.000109420	-0.000013778	-0.000060058
139	1	0.000156274	0.000348859	-0.000091205
140	1	0.000121095	0.000345290	0.000023374
141	1	0.000413496	0.000307515	0.000071605
142	8	-0.001322323	0.001515962	-0.000837614
143	1	0.000474070	-0.000896903	0.000004039
144	1	0.000564069	-0.000380478	0.000869518
145	8	0.000122422	0.000311991	0.000694432
146	1	0.000034175	-0.000174226	0.000989458
147	1	0.000963521	0.000576852	-0.000046345
148	8	0.005773046	0.003803541	-0.003799865
149	1	-0.003350902	-0.003276917	0.003758025
150	1	-0.003453465	-0.000129372	-0.001008593
151	8	-0.000179861	-0.000490160	-0.000437523
152	1	0.000842149	-0.000711788	-0.000033624
153	1	0.000150649	0.000088226	-0.001016489
154	8	0.002946034	-0.006538228	0.007419267
155	1	-0.003246325	0.004788480	-0.003144600
156	1	-0.000636842	0.002939639	-0.005731634
157	1	0.001322268	0.002725165	-0.001482565
158	1	-0.000414737	0.000442434	-0.000464648
159	1	0.000069787	0.000152058	0.000101422
160	1	0.001162948	0.000157093	-0.000280439
161	1	0.000057010	0.000013950	-0.000664832
162	8	-0.000701805	0.000539891	0.000082733
163	8	-0.002868149	0.000348758	-0.001373188
164	6	0.000812004	-0.001149666	-0.000639111
165	6	-0.001203005	0.000302406	-0.000892110
166	6	0.002840789	-0.000426039	0.001882829
167	6	-0.001153625	-0.000111480	-0.000736502
168	6	0.001029153	-0.000491520	0.000047086
169	6	-0.000446102	0.000717248	0.001110506
170	6	0.000041454	0.000136184	-0.000389619
171	6	0.000264942	-0.000140682	0.001012150
172	1	0.000431756	0.000059152	0.000315173
173	1	-0.000113305	-0.000279228	-0.000670530
174	1	0.000040395	0.000145642	0.000062533
175	1	0.000011739	-0.000028806	-0.000280797
176	1	-0.000292927	-0.000032037	-0.000356263
177	1	-0.000063996	0.000155727	-0.000315313
178	8	-0.001692741	-0.002388864	0.001016495

179	8	-0.001448065	-0.000512450	0.000851525
180	6	0.000745680	0.001360129	0.000154857
181	6	-0.001647322	-0.001547173	-0.000233539
182	6	0.002684263	0.000994374	-0.001310777
183	6	-0.001206343	-0.000658407	0.001463649
184	6	0.000694334	-0.000483800	0.000310080
185	6	-0.000928792	-0.000361103	0.000050751
186	6	-0.000280811	0.000007254	-0.000487730
187	6	-0.000435458	-0.000184303	0.001389208
188	1	0.000380497	0.000708580	-0.000210560
189	1	0.000272561	0.000447826	-0.000789729
190	1	0.000049141	-0.000040605	-0.000012849
191	1	0.000024457	0.000117264	-0.000403503
192	1	0.000361777	0.000111540	-0.000410968
193	1	0.000076915	-0.000065586	-0.000405446
194	8	-0.000289432	-0.000889239	-0.000822013
195	8	-0.000007488	-0.000760966	-0.000332331
196	6	0.000136073	0.000429293	0.000921506
197	6	0.000391321	-0.000676316	-0.001221800
198	6	-0.000306218	0.000748115	0.000412961
199	6	-0.000092710	-0.000513840	0.000353227
200	6	0.000309160	0.000402447	0.000177116
201	6	-0.000287102	-0.000346324	-0.000262006
202	6	0.000952053	0.000048688	-0.000351360
203	6	-0.000519393	-0.000356040	0.001139070
204	1	-0.000170008	0.000149816	0.000408773
205	1	-0.000277329	0.000253218	-0.000507483
206	1	0.000133215	0.000031472	-0.000027955
207	1	-0.000020011	0.000320095	-0.000374801
208	1	0.000216820	0.000056811	-0.000425387
209	1	-0.000010551	-0.000013465	-0.000219408
210	8	-0.001280362	-0.000753300	0.001043220
211	8	-0.001002694	-0.001147975	0.000127270
212	6	0.001951796	0.000713029	-0.001147562
213	6	-0.000504627	-0.000572380	0.000087529
214	6	0.000436868	0.000740653	0.000013975
215	6	-0.000535495	-0.000576025	0.000187852
216	6	0.000324506	0.000804224	0.000197835
217	6	-0.001086473	-0.000100695	0.001248666
218	6	0.000697396	0.000392718	-0.000444297
219	6	-0.000623866	0.000073736	0.001214749
220	1	0.000000558	0.000165996	-0.000092550
221	1	0.000536566	-0.000218370	-0.000812887
222	1	-0.000056644	-0.000013908	0.000024395
223	1	0.000265521	-0.000289254	-0.000352942
224	1	0.000026928	-0.000031446	-0.000300552
225	1	0.000186103	0.000079905	-0.000363978
226	1	0.000881983	0.000850332	-0.000400869
227	1	0.000035588	-0.000088026	0.000392771
228	1	0.000317425	-0.000632167	0.000026019
229	1	-0.000024772	0.000301393	-0.000164061
230	1	0.000858656	-0.000517620	0.000858047
231	1	0.000001652	0.000187138	-0.000079495
232	1	0.000257486	-0.000528530	0.000362545
233	1	0.000016212	-0.000193673	-0.000245591
234	1	-0.000264261	0.000680770	0.000189359
235	1	0.000064413	0.001122682	-0.000198597
236	8	-0.000492266	-0.000463956	-0.000357838

237	8	-0.002500740	0.000430615	0.001246658
238	6	0.000879624	0.000810601	0.000656682
239	6	-0.001155652	0.000067411	0.000483587
240	6	0.002509610	-0.000162795	-0.001300230
241	6	-0.000621308	0.000341238	0.000175889
242	6	0.000699019	0.000064638	-0.000093961
243	6	-0.000340779	-0.000394154	-0.000960256
244	6	0.000091331	-0.000221289	0.000498155
245	6	0.000292162	0.000004312	-0.001211746
246	1	0.000438737	-0.000142386	-0.000186900
247	1	-0.000142712	0.000384394	0.000722462
248	1	-0.000037471	-0.000130072	-0.000057119
249	1	-0.000076571	-0.000093744	0.000366622
250	1	0.000038304	0.000039270	0.000339584
251	1	-0.000244589	0.000187107	0.000341795
252	8	-0.000033448	-0.000122776	0.000323781
253	8	0.001942560	-0.000773843	0.001201311
254	6	-0.000314503	-0.000202172	0.000165509
255	6	0.000833351	-0.000339770	0.000627073
256	6	-0.002007407	0.000291184	-0.001165897
257	6	0.000381970	-0.000390257	0.000029344
258	6	-0.000385992	0.000097639	-0.000054818
259	6	0.000171085	0.000094442	-0.000683283
260	6	0.000006259	0.000435643	0.000492899
261	6	-0.000366414	-0.000157150	-0.001232242
262	1	-0.000339762	0.000235257	-0.000273956
263	1	0.000122412	-0.000510505	0.000706740
264	1	0.000052461	0.000130976	-0.000038132
265	1	0.000083735	0.000133354	0.000400496
266	1	-0.000009032	-0.000008017	0.000317139
267	1	0.000272299	-0.000146178	0.000365280
268	8	-0.000027177	-0.001161982	0.000626447
269	8	-0.000397320	-0.000130020	-0.000531795
270	6	-0.000203788	0.000775105	-0.000105316
271	6	0.000266628	-0.000441604	-0.000117928
272	6	0.000203499	0.000651132	0.000910241
273	6	-0.000326206	-0.000275107	-0.000881458
274	6	-0.000306846	0.000159801	-0.000115677
275	6	0.000042830	-0.000284325	-0.000524287
276	6	0.000069683	-0.000321740	0.000454544
277	6	-0.000284206	-0.000212065	-0.001265409
278	1	-0.000183364	0.000138849	0.000000253
279	1	0.000521044	0.000320660	0.000828636
280	1	-0.000048461	0.000067973	0.000021027
281	1	0.000095633	-0.000050178	0.000369550
282	1	0.000220881	0.000266240	0.000387758
283	1	-0.000037074	0.000056855	0.000357231
284	8	-0.000074700	0.000966827	0.000696682
285	8	0.000442237	-0.000660861	-0.000038898
286	6	0.000181191	-0.000590105	-0.000100842
287	6	-0.000230565	0.000169504	0.000043113
288	6	-0.000124101	0.000114317	0.000374355
289	6	0.000172742	-0.000013939	-0.000625496
290	6	0.000246652	0.000045742	-0.000057152
291	6	-0.000019872	0.000126134	-0.000609093
292	6	-0.000074809	0.000270825	0.000471157
293	6	0.000284487	0.000185238	-0.001214299
294	1	0.000124924	0.000013610	-0.000041162

295	1	-0.000520564	-0.000214491	0.000766466
296	1	0.000060506	-0.000030611	0.000003918
297	1	-0.000089936	0.000051047	0.000348916
298	1	-0.000242410	-0.000215296	0.000335059
299	1	0.000033797	-0.000053803	0.000347255
300	1	0.000334329	-0.000161689	0.000694284
301	1	-0.002291500	-0.000361167	0.001442517
302	1	0.000237136	0.000655283	-0.000902746
303	1	-0.002380658	0.001036479	-0.001644189
304	1	-0.001355191	-0.002189022	0.000739017
305	1	0.000652885	0.000507182	-0.001232693
306	1	-0.000924064	-0.000886522	-0.000562603
307	1	0.000481110	-0.000604897	0.000947506
308	1	-0.000661045	0.001399139	0.000799818
309	1	0.002607295	-0.003182572	-0.002048379
310	8	-0.000517687	0.000071885	-0.000638419
311	8	0.002121268	0.001184415	-0.000909190
312	6	0.000434013	-0.000335228	0.000204138
313	6	0.000805944	0.000656055	-0.000517598
314	6	-0.000963967	-0.001055907	0.000092456
315	6	-0.000215519	0.000265524	-0.000062456
316	6	-0.000136850	-0.000191970	-0.000020377
317	6	-0.000560340	0.000186405	0.000046526
318	6	0.000494894	-0.000206853	0.000213877
319	6	-0.001270781	-0.000061649	0.000083876
320	1	-0.000468797	-0.000410412	0.000298056
321	1	0.000672517	0.000069016	-0.000377220
322	1	-0.000053674	-0.000124320	-0.000086198
323	1	0.000348444	-0.000072191	-0.000106828
324	1	0.000407280	0.000247723	-0.000098204
325	1	0.000346341	-0.000019219	0.000085183
326	8	0.000111824	0.000077009	0.001213602
327	8	0.001859992	-0.001841767	0.001133256
328	6	-0.000128245	-0.000087108	-0.000813992
329	6	0.000938197	-0.000828339	0.000735369
330	6	-0.000775887	0.001396423	-0.000372941
331	6	-0.000195832	-0.000333960	0.000275279
332	6	-0.000003703	0.000141550	-0.000140333
333	6	-0.000335419	-0.000147685	0.000277647
334	6	0.000539140	-0.000024061	-0.000435040
335	6	-0.001183526	0.000332264	0.000136366
336	1	-0.000540184	0.000483182	-0.000349248
337	1	0.000747177	-0.000155908	0.000329208
338	1	-0.000007143	0.000163954	0.000041855
339	1	0.000334864	-0.000323318	0.000094623
340	1	0.000340156	-0.000029728	-0.000124894
341	1	0.000313699	-0.000012093	0.000047795
342	8	-0.000787085	-0.001362478	0.001902439
343	8	0.000312960	0.000976299	0.000926808
344	6	0.000206933	0.001151869	-0.001220404
345	6	-0.000136187	-0.000446020	0.000562156
346	6	0.000554039	-0.000391060	-0.001351402
347	6	-0.000996557	0.000533540	0.000567741
348	6	-0.000005349	0.000475529	-0.000401184
349	6	-0.000400502	-0.000320824	0.000499444
350	6	0.000461617	-0.000124378	0.000277734
351	6	-0.001209646	0.000499618	0.000227091
352	1	0.000036020	0.000152647	0.000030647

353	1	0.000714204	-0.000646404	-0.000248875
354	1	0.000004755	-0.000207886	0.000008666
355	1	0.000329183	-0.000029100	-0.000020707
356	1	0.000358604	-0.000219881	-0.000002846
357	1	0.000355082	-0.000214179	-0.000292539
358	8	-0.000747372	0.000828670	-0.001167208
359	8	0.000421012	-0.001415187	-0.000461692
360	6	0.000407568	-0.000513950	0.000554406
361	6	0.000044396	-0.000049413	-0.000187415
362	6	0.000152836	0.000367823	0.000652430
363	6	-0.000717412	-0.000362637	-0.000117347
364	6	-0.000165652	-0.000186194	0.000061320
365	6	-0.000683397	0.000077131	-0.000180002
366	6	0.000468629	0.000320481	-0.000351628
367	6	-0.001255109	-0.000440822	-0.000093666
368	1	0.000103553	0.000092708	-0.000097884
369	1	0.000744237	0.000459400	0.000148521
370	1	0.000012523	0.000215031	-0.000060750
371	1	0.000396554	0.000151371	0.000210696
372	1	0.000294197	-0.000001454	-0.000008427
373	1	0.000432460	0.000170134	-0.000025719
374	1	-0.000319314	0.000649660	-0.000581396
375	1	-0.000164181	0.000300884	0.000295476
376	1	0.000854450	0.000540760	0.000313769
377	1	0.002638027	0.000413918	0.001643918
378	1	-0.000371542	-0.000201298	-0.000440193
379	1	-0.000575842	0.000342592	0.000804330
380	1	0.000536546	0.000369533	0.000003642
381	1	-0.000373870	0.000394195	0.001422155
382	1	0.001007033	-0.000587167	0.000255152
383	1	0.001671245	0.002541217	0.001034890
384	8	0.000631471	-0.000976343	0.001135352
385	8	0.000783421	-0.000781970	-0.000231773
386	6	-0.000471611	0.000583443	-0.000429304
387	6	-0.000002733	-0.000594085	0.000170656
388	6	-0.000746345	0.001715188	-0.000011941
389	6	0.001000432	-0.000549236	-0.000041128
390	6	0.000222047	0.000337252	-0.000223286
391	6	0.000457721	-0.000382772	0.000008631
392	6	-0.000603334	-0.000204831	-0.000095503
393	6	0.001405418	-0.000432895	-0.000037445
394	1	-0.000076118	-0.000154625	-0.000021712
395	1	-0.000780319	0.000217662	0.000333589
396	1	-0.000032161	-0.000039184	0.000054866
397	1	-0.000392944	0.000393737	0.000148631
398	1	-0.000362869	0.000084731	-0.000119617
399	1	-0.000411076	0.000036231	0.000032223
400	8	0.000310839	-0.000217305	-0.001043113
401	8	-0.002500798	-0.001570028	-0.001239200
402	6	-0.000229899	0.000187687	0.000629593
403	6	-0.000883280	-0.000743187	-0.000705287
404	6	0.001212551	0.001461183	0.000367779
405	6	0.000080702	-0.000329846	-0.000278171
406	6	0.000100032	0.000157908	-0.000017485
407	6	0.000330377	-0.000366794	-0.000042561
408	6	-0.000560575	0.000033150	0.000506447
409	6	0.001296482	0.000011410	-0.000314902
410	1	0.000577299	0.000378499	0.000374998

411	1	-0.000714096	-0.000053713	-0.000286485
412	1	0.000057659	0.000136768	-0.000016182
413	1	-0.000381551	0.000068074	0.000197353
414	1	-0.000335405	0.000081949	0.000005938
415	1	-0.000434913	-0.000226465	-0.000015585
416	8	0.000249587	-0.001150475	0.000152867
417	8	-0.000937534	-0.001179666	-0.000076571
418	6	-0.000449969	0.001593204	0.000573162
419	6	-0.000641782	-0.000691520	-0.000229043
420	6	0.000410733	0.000497420	-0.000141551
421	6	0.000035113	-0.000438789	-0.000387748
422	6	0.000170155	0.000001557	0.000713288
423	6	0.000942225	-0.001058788	-0.000140915
424	6	-0.000311227	0.000599243	0.000475168
425	6	0.001127528	-0.000958232	-0.000128960
426	1	0.000291938	0.000219419	0.000015702
427	1	-0.000602183	0.000650835	-0.000107742
428	1	0.000061109	0.000208835	0.000064450
429	1	-0.000356628	0.000357543	-0.000166128
430	1	-0.000305549	0.000128397	0.000043852
431	1	-0.000379295	0.000302347	0.000135674
432	8	-0.000180956	-0.001217389	-0.002286144
433	8	-0.000298041	0.000546396	-0.000968495
434	6	0.000557233	0.001172554	0.001440750
435	6	-0.000470324	-0.000416126	-0.000667036
436	6	-0.000534515	-0.000351154	0.001509398
437	6	0.001051170	0.000274275	-0.000774368
438	6	0.000145526	0.000408406	0.000705009
439	6	0.000060397	-0.000608090	-0.000708547
440	6	-0.000462368	0.000339515	-0.000145385
441	6	0.001416167	0.000014020	-0.000209495
442	1	0.000348357	0.000209310	-0.000049859
443	1	-0.000725410	-0.000338462	0.000211813
444	1	0.000013684	-0.000184429	-0.000051236
445	1	-0.000439598	-0.000086509	-0.000018458
446	1	-0.000430384	-0.000072359	0.000282538
447	1	-0.000415989	0.000091279	-0.000016545
448	1	-0.000021454	0.000955774	0.000004592
449	1	0.000290647	0.000114794	-0.000246509
450	1	0.000947927	-0.000652044	0.000388409
451	1	0.000336155	0.000084627	0.001000971
452	1	0.000789066	0.000824984	-0.000313970
453	1	0.001060310	-0.002572145	-0.001086988
454	1	0.000950541	0.000236754	-0.000822529
455	1	0.000341477	0.000127204	0.000080882
456	1	0.000107876	0.000623945	0.000767015
457	1	-0.000315378	0.000476534	0.000019964
458	6	-0.002351001	-0.000743689	0.002257857
459	6	0.000124676	0.000015881	0.000531773
460	6	-0.000292706	0.000328734	-0.000425752
461	6	0.000055608	-0.000199904	-0.000031911
462	6	-0.000090295	-0.000440357	0.000083684
463	6	-0.000650700	0.000371497	-0.000590713
464	1	0.002067110	-0.004282832	0.004315428
465	1	0.004995274	-0.003742097	-0.001854013
466	1	-0.001988642	0.004779885	-0.003896359
467	1	-0.004278952	0.004607545	0.002469443
468	17	0.000530529	-0.004189564	0.001417123

469	1	0.000200104	0.000188114	-0.000063628
470	1	0.000489167	0.000450278	-0.000026941
471	1	0.000435526	-0.000110188	-0.000269102
472	8	0.001768898	0.001411114	0.001149601
473	1	-0.000747698	-0.000793951	-0.000246931
474	1	-0.000681592	-0.000253130	-0.000774415
475	8	-0.001228709	-0.001687830	0.000515947
476	1	0.000461174	0.000404214	-0.000432123
477	1	0.000683741	0.000951934	0.000080585
478	8	0.001270757	-0.002315637	-0.000582421
479	1	-0.000938411	0.001365519	-0.000148583
480	1	-0.000014629	0.000826013	0.000124831
481	6	0.002569563	0.002416567	-0.001342297
482	8	0.001516662	-0.001661105	0.004970233
483	1	0.002859745	0.000594360	-0.005849578

Sum of electronic and thermal Free Energies= -1284.432236
0 imaginary frequency

β-TS3

Center Number	Atomic Number	Integrated Forces (Hartrees/Bohr) X	Y	Z
1	6	-0.003944283	-0.000632107	-0.001632513
2	6	0.000897260	-0.002881828	0.001569430
3	7	0.002614072	0.006989350	-0.002044940
4	6	-0.005468772	-0.003255097	0.000814874
5	6	0.005118768	0.002153579	0.000800453
6	6	0.002239662	-0.005677780	0.004478014
7	1	-0.001428650	-0.005734045	0.003123829
8	1	-0.006136926	-0.001381086	-0.002644305
9	1	0.001527083	-0.002029759	-0.004791962
10	1	0.004277767	0.006477810	0.001016309
11	1	-0.006736612	-0.000231216	-0.000740824
12	1	0.003151889	-0.002050694	-0.004743351
13	1	0.001227569	0.006891689	0.001714571
14	8	0.000119783	-0.000988172	0.001733214
15	8	-0.002189555	0.004200447	-0.004879672
16	6	-0.000173236	0.000968533	-0.000927502
17	6	0.000743919	-0.000360040	0.000690583
18	6	0.000752956	-0.002105291	0.001961863
19	6	-0.000000296	-0.001136118	-0.001025882
20	6	-0.000433525	0.000862429	-0.000616940
21	6	0.000154979	-0.000827021	-0.000066959
22	6	-0.000186377	-0.000162030	0.000535179
23	6	0.000228727	-0.000653382	-0.001543519
24	1	-0.000353313	-0.000109572	-0.000262852
25	1	0.000259648	0.000321616	0.000862357
26	1	0.000104344	0.000333070	0.000173804
27	1	0.000074645	0.000423371	0.000351697
28	1	-0.000140459	0.000162013	0.000422949
29	1	-0.000043053	0.000081314	0.000470949
30	8	-0.000605078	0.000034694	0.000314364
31	8	-0.002095213	-0.001516129	0.002680829
32	6	-0.000109494	0.000163868	-0.000110654
33	6	-0.000686243	-0.000551181	0.000862889

34	6	0.000921280	0.001605476	-0.001726801
35	6	-0.000267265	-0.000582225	0.000206750
36	6	0.000002917	0.000448452	-0.000214813
37	6	0.000458010	-0.000451887	-0.000457064
38	6	-0.000169994	0.000383587	0.000445396
39	6	0.000664946	-0.000473734	-0.001015708
40	1	0.000519211	0.000273197	-0.000317252
41	1	-0.000553433	0.000085372	0.000355513
42	1	-0.000037854	0.000150816	-0.000147578
43	1	-0.000313809	-0.000032259	0.000326962
44	1	-0.000101420	0.000159496	0.000339191
45	1	-0.000261999	0.000242521	0.000250643
46	8	-0.002708563	-0.000299647	-0.001796836
47	8	-0.001997017	-0.000129231	-0.000564574
48	6	0.001157115	0.002685325	0.002768004
49	6	-0.001734492	0.000505741	-0.000612954
50	6	0.001808019	-0.000235400	0.000338838
51	6	-0.000929390	0.000089241	-0.000380653
52	6	0.001115234	0.000051545	0.000283096
53	6	0.000130765	-0.001471817	-0.001626703
54	6	0.000150788	0.000434923	0.000634411
55	6	0.000528326	-0.000569664	-0.001195527
56	1	0.000473697	-0.000799232	-0.000267145
57	1	-0.000402852	0.000696920	0.000568016
58	1	0.000033619	-0.000136706	0.000017967
59	1	-0.000077005	0.000134370	0.000402045
60	1	-0.000359798	0.000297621	0.000296310
61	1	-0.000168958	0.000030934	0.000349098
62	8	-0.001827403	-0.001768718	-0.000228990
63	8	-0.000473223	-0.000518792	-0.000310164
64	6	0.001492372	0.001283651	-0.000383046
65	6	-0.000285919	-0.000750734	0.000124630
66	6	0.000273970	0.000451021	0.000509065
67	6	-0.000191761	-0.000417348	-0.000610201
68	6	0.000620974	0.000306132	0.000025410
69	6	-0.000496114	-0.000491338	0.000056872
70	6	-0.000064944	0.000652049	0.000276699
71	6	-0.000052423	-0.000798956	-0.001372622
72	1	0.000008695	0.000385857	-0.000370355
73	1	-0.000174474	-0.000151798	0.000830779
74	1	0.000119678	0.000044008	-0.000060999
75	1	-0.000090938	0.000133884	0.000372710
76	1	0.000165135	0.000123453	0.000485858
77	1	-0.000077406	0.000331303	0.000416500
78	8	0.000118583	0.000257189	0.000000811
79	8	0.000327410	0.001503227	-0.002816237
80	6	-0.000489153	-0.000535688	-0.000395071
81	6	0.000031846	0.000865552	-0.000818952
82	6	-0.000600016	-0.001546352	0.001406955
83	6	-0.000074349	0.000538484	-0.000144530
84	6	-0.000142570	-0.000385818	0.000263895
85	6	0.000148578	0.000179124	0.000662231
86	6	0.000224046	-0.000286477	-0.000398489
87	6	-0.000207175	0.000219830	0.001267091
88	1	0.000183223	-0.000351095	0.000436672
89	1	-0.000115556	0.000118638	-0.000804775
90	1	-0.000165807	-0.000120922	0.000067042
91	1	-0.000029068	-0.000158254	-0.000396044

92	1	0.000033135	0.000135930	-0.000481635
93	1	0.000145508	-0.000116546	-0.000307943
94	8	-0.000392541	-0.000406678	-0.000004108
95	8	0.001136679	-0.002458258	-0.001474843
96	6	0.000877994	0.000490833	-0.000241023
97	6	0.000302341	-0.001045390	-0.000259080
98	6	-0.000260558	0.002006595	0.000579200
99	6	0.000084204	-0.000559925	0.000279403
100	6	0.000001748	0.000523644	0.000000943
101	6	-0.000584991	0.000023758	0.000476535
102	6	0.000177365	0.000075069	-0.000529192
103	6	-0.000728723	0.000283888	0.001032532
104	1	-0.000235235	0.000424777	0.000022570
105	1	0.000622482	-0.000329910	-0.000398455
106	1	0.000045232	0.000148049	0.000045667
107	1	0.000268446	0.000014306	-0.000260534
108	1	0.000308744	-0.000326835	-0.000228971
109	1	0.000129014	-0.000060284	-0.000327738
110	8	0.000868318	-0.001526678	0.001159801
111	8	-0.000317132	-0.000137040	0.000687788
112	6	-0.000493240	0.001118823	-0.000578647
113	6	-0.000061422	-0.000928376	0.000209991
114	6	-0.000096274	0.000181753	-0.001061419
115	6	-0.000047709	0.000287528	0.000964015
116	6	-0.000159480	0.000235171	0.000038559
117	6	-0.000187678	-0.000177399	0.000470782
118	6	0.000387526	0.000003622	-0.000364394
119	6	-0.000119914	0.000226708	0.001322932
120	1	0.000134322	0.000575353	0.000134569
121	1	0.000066882	-0.000469185	-0.000746944
122	1	-0.000076919	0.000093343	-0.000027375
123	1	0.000067816	0.000031865	-0.000411319
124	1	0.000111960	-0.000113567	-0.000348276
125	1	-0.000133636	-0.000126409	-0.000466229
126	8	-0.001537978	0.001966851	-0.000618075
127	8	-0.000575989	0.000005779	0.000581045
128	6	0.000843870	-0.001443237	0.000652028
129	6	-0.000452169	0.000812698	0.000105390
130	6	0.001077183	-0.000399091	-0.000593533
131	6	-0.000762252	0.000144661	0.000860693
132	6	0.000086057	-0.000280398	0.000289945
133	6	-0.000308114	0.000466602	0.000266908
134	6	-0.000014297	-0.000134551	-0.000433976
135	6	-0.000874157	0.000317314	0.001007315
136	1	-0.000024301	-0.000313894	-0.000294445
137	1	0.000517042	0.000088115	-0.000726005
138	1	0.000075317	-0.000068385	0.000039801
139	1	0.000224822	-0.000203985	-0.000261259
140	1	0.000190227	-0.000060858	-0.000338913
141	1	0.000412484	-0.000035100	-0.000261691
142	8	-0.000862145	-0.000840960	-0.001594995
143	1	0.000356315	-0.000295823	0.001069547
144	1	0.000561763	0.000975620	0.000337145
145	8	0.000120804	0.000572449	-0.000554940
146	1	0.000267522	0.000898040	-0.000039461
147	1	0.001075342	-0.000251826	-0.000107500
148	8	0.014112230	-0.004892150	0.000581839
149	1	-0.003188889	0.005238085	0.000256478

150	1	-0.005969030	-0.000488387	-0.001855532
151	8	-0.000336492	-0.000407507	0.000642883
152	1	0.000649997	0.000114402	0.000836194
153	1	-0.000073753	-0.000856723	0.000178246
154	8	0.002390174	0.008347456	0.005297670
155	1	-0.002050188	-0.003899784	-0.004869965
156	1	-0.001153587	-0.006058721	-0.001580755
157	1	0.002216439	-0.003116718	-0.001556232
158	1	-0.000334515	-0.000318482	0.000048135
159	1	-0.000117442	-0.000166785	-0.000335760
160	1	0.000694198	-0.000017265	-0.000174766
161	1	0.001568822	-0.001857632	0.002016419
162	8	-0.000486757	0.000051324	-0.000485605
163	8	-0.002903213	-0.001053649	-0.000994787
164	6	0.000324453	-0.000476731	0.001162751
165	6	-0.001259887	-0.000499600	-0.000315218
166	6	0.002832148	0.001393241	0.000916757
167	6	-0.001087372	-0.000238613	-0.000090801
168	6	0.000692668	0.000029352	0.000527313
169	6	0.000011670	0.000927267	-0.000771466
170	6	0.000032957	-0.000460603	0.000042367
171	6	0.000350783	0.001062850	-0.000095102
172	1	0.000530440	0.000170545	-0.000060850
173	1	-0.000325712	-0.000477761	0.000356441
174	1	0.000098972	-0.000020266	-0.000150436
175	1	-0.000019279	-0.000267116	0.000125174
176	1	-0.000361989	-0.000351279	0.000060411
177	1	-0.000075867	-0.000358536	-0.000077328
178	8	-0.001981336	0.001682720	0.002460718
179	8	-0.000507245	0.000143618	0.000619535
180	6	0.001877532	0.000327914	-0.002707265
181	6	-0.001659256	0.001223379	0.001881914
182	6	0.001638423	-0.000628869	-0.000987480
183	6	-0.001100530	0.000795871	0.000386979
184	6	0.000343933	0.000368512	0.000341400
185	6	-0.000795333	0.000459473	0.000524445
186	6	-0.000264874	-0.000266665	0.000169717
187	6	-0.000099941	0.001312368	-0.000140132
188	1	0.001812003	0.000959857	-0.001644995
189	1	0.000407413	-0.000676585	-0.000234648
190	1	0.000010388	0.000007719	0.000107138
191	1	-0.000048558	-0.000367683	-0.000112311
192	1	0.000339871	-0.000510358	0.000060890
193	1	-0.000011198	-0.000365407	0.000136505
194	8	-0.000202030	-0.000254151	0.000849587
195	8	0.000221232	-0.000206274	0.000590434
196	6	-0.000181338	0.000378927	-0.000366160
197	6	0.000236286	-0.000659048	0.000677740
198	6	-0.000737064	0.000735962	-0.000948537
199	6	0.000113888	0.000189815	0.000278685
200	6	0.000044240	0.000207648	-0.000029189
201	6	0.000122000	0.000262050	0.000178163
202	6	0.000540304	-0.000134120	0.000110593
203	6	-0.000385282	0.001188286	0.000147711
204	1	-0.000177597	0.000140293	-0.000187483
205	1	-0.000381623	-0.000512810	-0.000180940
206	1	0.000168723	-0.000029520	-0.000076738
207	1	0.000062403	-0.000412440	-0.000246907

208	1	0.000214906	-0.000446437	0.000056978
209	1	-0.000076839	-0.000289673	0.000013869
210	8	-0.002881161	0.003405735	-0.002370127
211	8	-0.000553946	0.000964109	0.000588013
212	6	0.002218147	-0.001219324	-0.000139363
213	6	-0.000485458	-0.000466755	0.000463595
214	6	0.000132465	-0.000641608	-0.000303975
215	6	-0.000258286	0.000641583	-0.000030361
216	6	0.000274588	0.000103132	-0.000409104
217	6	-0.000750928	0.000956506	-0.000343687
218	6	0.000372292	-0.000623299	-0.000115629
219	6	-0.000011620	0.001190111	-0.000525670
220	1	0.000133605	0.000463545	-0.000363554
221	1	0.000172022	-0.000798399	0.000464246
222	1	-0.000064681	-0.000010628	-0.000044006
223	1	-0.000055358	-0.000363749	0.000348704
224	1	-0.000095924	-0.000296050	0.000087669
225	1	0.000043516	-0.000391843	0.000086809
226	1	0.000510642	-0.000814801	-0.000549558
227	1	0.000105809	0.000241235	-0.000052677
228	1	-0.000049523	0.000156015	0.000840202
229	1	0.000008423	-0.000063123	-0.000257892
230	1	0.000773332	0.000849408	0.000425092
231	1	0.000082731	-0.000208485	-0.000068235
232	1	0.000039388	0.000498018	0.000501738
233	1	-0.000113825	-0.000072629	0.000145668
234	1	-0.000152307	-0.000036154	-0.000739285
235	1	-0.000069756	-0.000360153	-0.000335260
236	8	-0.000652814	-0.000082243	0.000373726
237	8	-0.001933014	0.001140360	-0.001350282
238	6	0.001094328	0.000304891	-0.000472019
239	6	-0.000881988	0.000600767	-0.000489915
240	6	0.002112679	-0.001377808	0.001133071
241	6	-0.000539714	0.000076566	-0.000524263
242	6	0.000527980	-0.000202643	0.000117877
243	6	-0.000468681	-0.000666813	0.000366293
244	6	0.000038874	0.000528040	0.000192347
245	6	0.000203105	-0.001230628	0.000238248
246	1	0.000264810	-0.000196641	0.000276257
247	1	0.000104353	0.000612562	-0.000583915
248	1	-0.000085595	-0.000001586	0.000146630
249	1	-0.000067064	0.000409872	0.000025969
250	1	0.000063602	0.000301416	-0.000095223
251	1	-0.000199523	0.000375869	-0.000260778
252	8	0.000372756	0.000089654	-0.000026177
253	8	0.001988378	0.001157513	0.001134568
254	6	-0.000560638	0.000365206	0.000147172
255	6	0.000902622	0.000619495	0.000500503
256	6	-0.002196699	-0.001171629	-0.000682822
257	6	0.000476068	0.000220263	0.000505635
258	6	-0.000509147	-0.000102072	-0.000179347
259	6	0.000141287	-0.000737340	0.000023740
260	6	0.000199291	0.000322010	-0.000461100
261	6	-0.000623318	-0.001096452	0.000282227
262	1	-0.000334679	-0.000263389	-0.000285696
263	1	0.000118648	0.000753182	0.000302314
264	1	0.000113778	-0.000097195	-0.000104720
265	1	0.000214144	0.000325788	-0.000179657

266	1	0.000040567	0.000329151	-0.000050110
267	1	0.000315784	0.000334436	0.000078317
268	8	-0.000251615	0.000901449	0.000834462
269	8	-0.000329109	-0.000453211	-0.000206791
270	6	0.000096079	-0.000224112	-0.000540846
271	6	0.000100565	0.000084157	0.000351641
272	6	0.000386959	0.000592801	-0.000442141
273	6	-0.000415581	-0.000733126	0.000201501
274	6	-0.000193151	-0.000103196	-0.000072955
275	6	-0.000341205	-0.000383810	0.000240824
276	6	0.000043254	0.000495875	0.000209333
277	6	-0.000462107	-0.001132833	0.000336646
278	1	-0.000058422	-0.000016554	-0.000089636
279	1	0.000574460	0.000602836	-0.000064335
280	1	-0.000047743	0.000007244	-0.000072209
281	1	0.000134731	0.000338701	0.000009690
282	1	0.000310843	0.000325124	-0.000237691
283	1	0.000023020	0.000315151	-0.000139425
284	8	0.000404416	0.000396709	-0.001075735
285	8	0.000320442	0.000066642	0.000781663
286	6	-0.000105069	0.000027943	0.000630248
287	6	-0.000078012	0.000010074	-0.000221206
288	6	-0.000194832	0.000297071	-0.000231819
289	6	0.000231795	-0.000585466	0.000131563
290	6	0.000159620	-0.000139471	0.000097504
291	6	0.000164716	-0.000493095	0.000021370
292	6	-0.000030921	0.000336239	-0.000375219
293	6	0.000194666	-0.001249140	0.000156343
294	1	0.000135031	0.000036577	0.000011817
295	1	-0.000380285	0.000687564	-0.000274447
296	1	0.000032108	0.000007920	0.000090609
297	1	-0.000043996	0.000352405	-0.000179824
298	1	-0.000253030	0.000475973	0.000154143
299	1	0.000076860	0.000374697	-0.000002866
300	1	0.000349116	0.000587281	0.000137130
301	1	-0.002154810	0.001760874	-0.000654017
302	1	0.000230281	-0.000731302	-0.000289458
303	1	-0.002112722	-0.001692468	-0.001309706
304	1	-0.001713908	0.001395100	0.001603194
305	1	0.000567597	-0.001313614	-0.000058799
306	1	-0.001176674	-0.000195332	0.000687384
307	1	0.000420216	0.001074635	0.000331163
308	1	-0.000331398	0.000454822	-0.001289020
309	1	0.000814753	-0.001244578	0.003594627
310	8	-0.000487389	-0.000623182	0.000027093
311	8	0.002244747	-0.001433611	-0.000409580
312	6	0.000257840	0.000259689	0.000290301
313	6	0.001003747	-0.000723586	-0.000268671
314	6	-0.001282205	0.000489825	0.000779470
315	6	-0.000069697	-0.000215866	-0.000319606
316	6	-0.000131946	0.000126537	0.000108905
317	6	-0.000468700	-0.000016708	-0.000326410
318	6	0.000444631	0.000245784	0.000231589
319	6	-0.001246836	0.000148624	-0.000317865
320	1	-0.000575777	0.000429351	0.000119884
321	1	0.000643566	-0.000548500	0.000201269
322	1	-0.000076721	-0.000045601	0.000077441
323	1	0.000312583	-0.000110785	0.000223791

324	1	0.000505130	-0.000201860	-0.000101733
325	1	0.000366081	0.000092868	0.000115476
326	8	-0.000140682	0.000981276	-0.000182472
327	8	0.001302101	0.001461268	0.002252468
328	6	0.000264758	-0.000454974	0.000032317
329	6	0.000578481	0.000860981	0.001030474
330	6	-0.000336083	-0.000637609	-0.001721439
331	6	-0.000283065	0.000403965	0.000268605
332	6	0.000043412	-0.000081894	-0.000284677
333	6	-0.000552523	0.000165741	-0.000020283
334	6	0.000494412	-0.000362105	0.000214669
335	6	-0.001086071	0.000115415	-0.000679031
336	1	-0.000346285	-0.000430440	-0.000534055
337	1	0.000567317	0.000287507	0.000308842
338	1	0.000034602	0.000002842	-0.000155948
339	1	0.000302451	0.000150624	0.000416958
340	1	0.000300334	-0.000140409	0.000164772
341	1	0.000339587	0.000033378	0.000088569
342	8	-0.000874127	0.001936974	0.000522844
343	8	0.000719323	0.000355993	-0.000922777
344	6	0.000469661	-0.001262567	-0.000687367
345	6	-0.000224601	0.000502004	0.000238843
346	6	0.000104955	-0.000925116	0.000747901
347	6	-0.000680697	0.000372203	-0.000763051
348	6	0.000045529	-0.000384549	-0.000378253
349	6	-0.000429514	0.000412337	0.000060720
350	6	0.000423886	0.000252584	0.000197605
351	6	-0.001018619	0.000186484	-0.000829595
352	1	0.000137772	0.000038673	-0.000176237
353	1	0.000434442	-0.000146934	0.000736355
354	1	-0.000067975	0.000076201	0.000160580
355	1	0.000322694	-0.000036178	0.000106256
356	1	0.000304951	0.000029140	0.000289822
357	1	0.000284259	-0.000203606	0.000347829
358	8	-0.000481229	-0.001373217	-0.000603623
359	8	-0.000030117	-0.000266814	0.001360800
360	6	0.000266023	0.000727036	0.000424884
361	6	-0.000001759	-0.000234008	-0.000122374
362	6	0.000471014	0.000622789	-0.000437657
363	6	-0.000836864	-0.000084031	0.000138010
364	6	-0.000229016	0.000114173	0.000197909
365	6	-0.000594338	-0.000186217	-0.000243962
366	6	0.000447350	-0.000412729	0.000003259
367	6	-0.001362038	0.000020975	-0.000040157
368	1	0.000011757	-0.000079894	0.000085937
369	1	0.000824045	0.000098682	-0.000121827
370	1	0.000063874	-0.000059034	-0.000224956
371	1	0.000495755	0.000198788	-0.000059653
372	1	0.000304262	-0.000007489	0.000151874
373	1	0.000476877	-0.000111356	-0.000003695
374	1	0.000150137	-0.000168989	-0.000848111
375	1	0.000512099	0.000056385	-0.000165086
376	1	0.000690566	0.000064327	-0.000207805
377	1	0.002845930	0.001325352	-0.000027477
378	1	-0.000504941	-0.000880245	0.001904904
379	1	-0.000515014	0.000775593	-0.000796919
380	1	0.000665550	-0.000100552	-0.000219511
381	1	-0.000082138	0.001185796	-0.001149293

382	1	0.000583696	0.000636537	0.001073520
383	1	0.002432601	0.000286550	-0.002291325
384	8	0.000377618	-0.000963889	0.000002686
385	8	-0.000613154	-0.000303357	-0.000114103
386	6	-0.000884298	0.001772359	0.000065408
387	6	0.000089424	-0.000492474	-0.001249596
388	6	0.000315038	0.000346905	0.000504963
389	6	0.000279545	-0.000136347	-0.000323386
390	6	-0.000046003	0.000050537	0.000398237
391	6	0.000963370	-0.000634390	0.000733818
392	6	-0.000589064	0.000417417	-0.000083917
393	6	0.001130614	-0.000392868	0.000843168
394	1	0.000343271	-0.000055914	0.000558291
395	1	-0.000431425	0.000468138	-0.000636507
396	1	-0.000040242	0.000170009	0.000115266
397	1	-0.000247154	0.000124614	-0.000474905
398	1	-0.000321557	-0.000007077	-0.000159979
399	1	-0.000383701	0.000134543	-0.000170150
400	8	0.000298688	-0.000745168	-0.000130839
401	8	-0.002996305	-0.000697169	0.000957908
402	6	-0.000440131	0.000717639	-0.000459518
403	6	-0.001118980	-0.000218676	0.000260294
404	6	0.001525747	0.000188990	-0.001371245
405	6	-0.000125347	0.000019204	-0.000013893
406	6	0.000092913	0.000067574	-0.000000762
407	6	0.000170508	0.000077647	0.000199601
408	6	-0.000425474	0.000503755	-0.000341132
409	6	0.001200251	-0.000295898	0.000425725
410	1	0.000682403	0.000275443	-0.000257743
411	1	-0.000645490	-0.000293450	-0.000050279
412	1	0.000120576	-0.000084329	-0.000137935
413	1	-0.000326399	0.000175032	-0.000227444
414	1	-0.000295918	-0.000054302	-0.000186904
415	1	-0.000459587	0.000097666	0.000103521
416	8	0.000191880	-0.000467101	-0.000977114
417	8	-0.001546530	0.000421631	0.001299347
418	6	-0.000271859	0.001536159	-0.001404156
419	6	-0.000708647	-0.000158607	-0.000311693
420	6	0.001292845	-0.000591755	-0.000211016
421	6	-0.000022380	-0.000313728	0.000333429
422	6	0.000035052	0.000491626	0.000008577
423	6	0.000928345	0.000028028	0.001119611
424	6	-0.000075772	0.000141208	-0.000750251
425	6	0.000951952	-0.000022609	0.000989498
426	1	0.000271259	-0.000001767	0.000021228
427	1	-0.000462345	-0.000208642	-0.000734708
428	1	0.000020492	0.000053745	-0.000142551
429	1	-0.000327817	-0.000266093	-0.000331256
430	1	-0.000289190	0.000033255	-0.000154388
431	1	-0.000283988	0.000061486	-0.000371390
432	8	-0.000599926	-0.001506730	0.002142698
433	8	-0.000220099	-0.001773681	-0.000443162
434	6	0.000782403	0.000972378	-0.001321733
435	6	-0.000443406	-0.000679186	0.000328491
436	6	-0.000858285	0.001918585	-0.000352854
437	6	0.001115527	-0.001296630	0.000206210
438	6	0.000281561	0.000689060	-0.000612249
439	6	-0.000131327	-0.000531046	0.000532161

440	6	-0.000167382	-0.000210393	-0.000504379
441	6	0.001207632	-0.000458308	0.000755842
442	1	0.000221451	-0.000131440	-0.000090922
443	1	-0.000829677	0.000335123	0.000102186
444	1	-0.000083883	0.000153803	0.000083485
445	1	-0.000427194	0.000072091	-0.000108319
446	1	-0.000366158	0.000364478	-0.000231283
447	1	-0.000364418	0.000024000	-0.000284727
448	1	0.000215377	-0.000272074	-0.000914013
449	1	0.001146172	-0.000475697	0.000220215
450	1	0.000755587	0.000350135	0.000826791
451	1	-0.000011149	0.000492488	-0.000228922
452	1	0.001410827	-0.000707223	-0.000514027
453	1	0.000189802	-0.000503684	0.002780491
454	1	0.000905886	-0.001009223	0.000219137
455	1	-0.000002985	0.000160423	-0.000227852
456	1	0.000084243	0.000550475	-0.000514637
457	1	-0.000200339	0.000179179	-0.000521723
458	6	-0.002157831	0.003602858	-0.000115371
459	6	0.000253560	0.000779487	0.000412919
460	6	0.000042430	-0.000847049	0.000277718
461	6	-0.000171000	-0.000198364	0.000824392
462	6	-0.000354299	0.000736442	0.000124378
463	6	-0.000718081	-0.000741954	-0.000746404
464	1	0.001371503	0.004795918	0.004019275
465	1	0.002984221	-0.001811411	0.005660362
466	1	-0.000920879	-0.004485465	-0.004511155
467	1	-0.002275599	0.002149858	-0.005907878
468	17	-0.006343735	-0.001367349	0.003942088
469	1	0.000453308	-0.000143459	-0.000305021
470	1	0.000728644	-0.000328500	-0.000213727
471	1	0.000417913	-0.000246767	0.000209493
472	8	0.002170341	0.000467241	-0.001019416
473	1	-0.001042415	0.000031458	0.000485204
474	1	-0.000812993	-0.000472686	0.000203766
475	8	-0.002142387	0.001434929	0.001001495
476	1	0.000727900	-0.000837732	-0.000169956
477	1	0.001027400	-0.000274038	-0.000609776
478	8	0.000628743	-0.000434090	0.002507752
479	1	-0.000550861	-0.000118289	-0.001552605
480	1	0.000129777	0.000130548	-0.000794066
481	6	-0.000040202	-0.003144764	-0.000694989
482	8	0.002559825	0.004528738	0.004017137
483	1	0.002006948	-0.006198834	0.001663934

Sum of electronic and thermal Free Energies= -1284.429818
1 imaginary frequency: -684.18

***β*-4a**

Center Number	Atomic Number	Integrated Forces (Hartrees/Bohr) X	Y	Z
1	6	-0.002156714	-0.000481916	-0.000807098
2	6	0.000448758	0.002889935	0.000280635
3	7	0.003404105	0.003423580	0.001387941
4	6	0.000489957	-0.000160193	-0.000349439

5	6	-0.001415860	-0.002162021	0.000584129
6	6	-0.003442421	-0.005729614	-0.000915744
7	1	0.000174691	-0.006597550	-0.000027704
8	1	0.005880343	-0.001016141	0.003157532
9	1	0.003691824	-0.002543590	-0.003928741
10	1	-0.003281062	0.005369827	-0.002182725
11	1	-0.000670957	0.001220035	0.006168508
12	1	-0.002229042	0.004919627	-0.003045352
13	1	0.006448091	-0.000066427	-0.001576275
14	8	0.000241382	-0.000076372	-0.000032717
15	8	0.004270783	0.007038516	0.000029846
16	6	-0.000302667	-0.000265072	-0.000342145
17	6	-0.000189873	0.000556920	-0.000233418
18	6	-0.001284412	-0.005107579	0.000528874
19	6	-0.000087686	0.000302079	0.000589647
20	6	0.000145310	-0.000102469	0.000241888
21	6	0.000027752	0.000071759	0.000654059
22	6	0.000063171	-0.000384491	-0.000450676
23	6	-0.000011443	0.000201337	0.001371857
24	1	0.000325473	-0.000274153	0.000110767
25	1	-0.000277690	-0.000000567	-0.000899288
26	1	-0.000119270	-0.000108290	0.000026042
27	1	-0.000069733	0.000209254	-0.000468186
28	1	0.000118495	-0.000085023	-0.000340766
29	1	-0.000038741	-0.000158078	-0.000422678
30	8	-0.000006692	-0.000156902	-0.000102231
31	8	0.001410815	-0.002028604	-0.001664820
32	6	0.000378515	0.000421214	-0.000201755
33	6	0.000522627	-0.000833504	-0.000395479
34	6	-0.000436211	0.001644009	0.000689023
35	6	0.000213034	-0.000454513	0.000355992
36	6	-0.000112335	0.000510052	-0.000072737
37	6	-0.000302393	-0.000005941	0.000535485
38	6	0.000011001	0.000113310	-0.000563666
39	6	-0.000554553	0.000133923	0.001127210
40	1	-0.000335364	0.000349366	0.000186410
41	1	0.000647630	-0.000265192	-0.000486319
42	1	0.000057111	0.000138206	0.000042909
43	1	0.000291336	-0.000285278	-0.000368333
44	1	0.000077461	-0.000023885	-0.000339394
45	1	0.000218241	0.000060521	-0.000301249
46	8	-0.000723359	0.001477899	-0.000057089
47	8	0.000508582	0.000066059	0.000443694
48	6	0.000596119	-0.001101233	-0.000136811
49	6	0.000293570	0.001086923	0.000083958
50	6	-0.000087044	-0.000363007	-0.000408910
51	6	-0.000175951	0.000071232	0.000629353
52	6	-0.000077592	-0.000090596	0.000088071
53	6	-0.000195586	0.000219804	0.000739613
54	6	-0.000043073	-0.000087661	-0.000560312
55	6	-0.000696829	0.000114132	0.001137176
56	1	-0.000284876	-0.000513314	0.000146401
57	1	0.000350207	0.000299991	-0.000741140
58	1	0.000050052	-0.000081335	0.000029520
59	1	0.000124414	-0.000035403	-0.000370023
60	1	0.000357736	0.000050988	-0.000256281
61	1	0.000187406	-0.000142795	-0.000302455
62	8	0.001409842	-0.001540667	0.000929293

63	8	0.000045768	-0.000224876	0.000571824
64	6	-0.000890648	0.001299351	-0.000431078
65	6	0.000187625	-0.000869707	0.000341254
66	6	-0.000353842	0.000125218	-0.001077216
67	6	0.000096464	0.000080734	0.001127155
68	6	-0.000326103	0.000238436	-0.000009286
69	6	-0.000004306	-0.000435233	0.000348771
70	6	0.000269429	0.000243090	-0.000461345
71	6	0.000052627	0.000090668	0.001439915
72	1	0.000227914	0.000486353	0.000005320
73	1	0.000001767	-0.000436097	-0.000749053
74	1	-0.000125497	0.000074265	-0.000009169
75	1	0.000074279	-0.000073422	-0.000373118
76	1	-0.000170187	-0.000105118	-0.000458167
77	1	0.000036977	0.000058293	-0.000460771
78	8	-0.000187202	0.000151221	0.000155100
79	8	0.000134235	0.002289810	0.002075969
80	6	0.000416325	-0.000329683	0.000358945
81	6	0.000069139	0.001010689	0.000599318
82	6	0.000291754	-0.001865737	-0.000714230
83	6	0.000248050	0.000511893	-0.000201795
84	6	0.000021961	-0.000443289	-0.000055013
85	6	-0.000154296	-0.000093565	-0.000584969
86	6	-0.000283388	-0.000083835	0.000469400
87	6	0.000148171	-0.000274479	-0.001248891
88	1	-0.000171024	-0.000398127	-0.000323819
89	1	0.000209872	0.000334311	0.000693765
90	1	0.000123167	-0.000119944	-0.000018695
91	1	0.000037126	-0.000011194	0.000389974
92	1	0.000006830	0.000306060	0.000397240
93	1	-0.000125418	0.000033620	0.000333476
94	8	0.000489073	-0.000416756	0.000202876
95	8	-0.001191531	-0.001538702	0.002637112
96	6	-0.000845093	0.000661096	0.000104036
97	6	-0.000238141	-0.000795150	0.000561856
98	6	0.000183461	0.001482781	-0.001429931
99	6	-0.000057217	-0.000496871	-0.000019830
100	6	0.000120363	0.000524267	-0.000019238
101	6	0.000480359	-0.000190534	-0.000713482
102	6	-0.000166100	0.000338832	0.000400230
103	6	0.000695782	-0.000236243	-0.001089151
104	1	0.000232105	0.000337187	-0.000229147
105	1	-0.000634227	-0.000143117	0.000521253
106	1	-0.000083462	0.000089463	-0.000122080
107	1	-0.000270235	0.000159284	0.000281364
108	1	-0.000282537	-0.000167108	0.000353236
109	1	-0.000094108	0.000088171	0.000345672
110	8	-0.001074257	-0.001667194	-0.000352685
111	8	0.000272159	-0.000124655	-0.000413242
112	6	0.000695499	0.001119838	0.000066810
113	6	-0.000010924	-0.000911391	0.000106078
114	6	0.000162967	0.000368362	0.000724408
115	6	-0.000069562	-0.000009586	-0.000860406
116	6	0.000118081	0.000168359	-0.000188696
117	6	0.000118643	-0.000391243	-0.000446887
118	6	-0.000303955	0.000155102	0.000363148
119	6	-0.000044853	-0.000295113	-0.001330549
120	1	-0.000131936	0.000381687	-0.000137223

121	1	-0.000087212	-0.000136566	0.000859922
122	1	0.000102789	0.000070004	-0.000025941
123	1	-0.000000903	0.000209353	0.000370556
124	1	-0.000073875	0.000042724	0.000395658
125	1	0.000199887	0.000026251	0.000484058
126	8	0.001818445	0.001785277	-0.000155089
127	8	0.000775700	-0.000253490	-0.000788804
128	6	-0.001188252	-0.001467741	0.000048921
129	6	0.000664068	0.000760675	-0.000585239
130	6	-0.001357951	-0.000183705	0.001072889
131	6	0.000788112	-0.000255633	-0.000961926
132	6	-0.000238042	-0.000348419	-0.000168101
133	6	0.000294921	0.000250951	-0.000375486
134	6	0.000068570	0.000011247	0.000534056
135	6	0.000750812	-0.000174928	-0.001150728
136	1	-0.000013889	-0.000238225	0.000443739
137	1	-0.000356061	0.000470668	0.000649842
138	1	-0.000068813	-0.000062580	-0.000005019
139	1	-0.000192087	-0.000047617	0.000349354
140	1	-0.000141891	0.000058721	0.000347839
141	1	-0.000437500	0.000142022	0.000304542
142	8	0.001120353	-0.000504508	0.001676015
143	1	-0.000468516	-0.000403548	-0.000869709
144	1	-0.000602116	0.000820846	-0.000646007
145	8	-0.000212086	0.000492308	0.000367349
146	1	-0.000196221	0.000884867	-0.000341016
147	1	-0.000955596	0.000086978	0.000329649
148	8	-0.006825669	-0.004941794	0.005804365
149	1	0.002411816	0.004797664	-0.002648194
150	1	0.005666086	0.000422005	-0.003168731
151	8	-0.000027794	-0.000598411	-0.000313926
152	1	-0.000654715	-0.000154514	-0.000867927
153	1	0.000040434	-0.000965777	0.000025082
154	8	-0.002536474	0.006331556	-0.007649319
155	1	0.002284959	-0.002073022	0.005765667
156	1	0.001029267	-0.005268397	0.003511722
157	1	-0.000132324	-0.000235943	-0.000044028
158	1	0.000471763	-0.000712121	-0.001104507
159	1	0.000255497	0.000088496	0.000398705
160	1	-0.000058705	-0.000272656	-0.000378884
161	1	-0.000928760	-0.000361161	-0.000771578
162	8	0.000608682	0.000336574	0.000622219
163	8	0.002822107	-0.001089829	0.001237085
164	6	-0.000485700	-0.000967203	-0.001294134
165	6	0.001217430	-0.000560811	0.000586713
166	6	-0.002734347	0.001382795	-0.001269750
167	6	0.001188779	-0.000360547	0.000176414
168	6	-0.000920815	-0.000083837	-0.000613248
169	6	0.000180258	0.001242806	0.000699631
170	6	-0.000124776	-0.000485733	-0.000012410
171	6	-0.000233181	0.001139263	-0.000102447
172	1	-0.000451064	0.000324292	-0.000041316
173	1	0.000237186	-0.000732286	-0.000236811
174	1	-0.000069972	0.000042310	0.000117960
175	1	-0.000032554	-0.000328642	-0.000032725
176	1	0.000322017	-0.000365424	-0.000008240
177	1	0.000049951	-0.000303163	0.000147642
178	8	0.000618540	-0.000224559	0.000022848

179	8	-0.000993045	0.000382907	0.000272858
180	6	-0.000269992	0.000051841	0.000223746
181	6	-0.000121697	0.000153579	-0.000004061
182	6	0.000261491	-0.000160023	0.000492106
183	6	0.000309089	0.000599110	-0.000024071
184	6	-0.000132063	0.000063863	-0.000309114
185	6	0.000283508	0.000163777	-0.000092528
186	6	0.000217077	-0.000525300	-0.000488906
187	6	0.000162856	0.001382817	0.000118047
188	1	0.000226273	-0.000219349	-0.000102612
189	1	-0.000347581	-0.000665952	0.000481524
190	1	-0.000002312	-0.000081071	-0.000207788
191	1	0.000057048	-0.000275306	0.000074458
192	1	-0.000264216	-0.000413735	-0.000010444
193	1	-0.000018218	-0.000455333	-0.000093528
194	8	-0.000011312	-0.000611103	-0.000610570
195	8	-0.000586427	0.000024256	0.000201755
196	6	0.000257991	0.000300438	0.000232538
197	6	-0.000270511	-0.000600347	-0.000250769
198	6	0.000319315	0.000170372	0.000040732
199	6	-0.000101427	0.000274608	-0.000102224
200	6	0.000010585	0.000092099	-0.000213834
201	6	-0.000262389	0.000429606	-0.000149277
202	6	-0.000216042	-0.000405971	-0.000052267
203	6	0.000170495	0.001087034	-0.000520777
204	1	0.000099345	0.000168456	0.000040978
205	1	0.000373407	-0.000601359	0.000357823
206	1	-0.000104330	0.000009328	-0.000010916
207	1	-0.000033862	-0.000319505	0.000376547
208	1	-0.000124683	-0.000342782	0.000098086
209	1	0.000046229	-0.000286625	0.000079933
210	8	0.001536133	0.001044360	0.001039522
211	8	0.000658753	0.000672534	-0.000676594
212	6	-0.001427028	-0.000920092	-0.000131885
213	6	0.000424810	0.000505361	0.000046028
214	6	-0.000496602	-0.001047416	0.000284891
215	6	0.000354325	0.001142825	0.000125547
216	6	0.000131848	0.000246371	0.000029242
217	6	0.000493177	0.000965965	0.000380208
218	6	-0.000425769	-0.000452560	0.000338990
219	6	0.000206635	0.001388017	0.000079227
220	1	0.000010779	-0.000361493	0.000082683
221	1	-0.000328231	-0.000821480	-0.000160712
222	1	0.000081703	0.000013419	-0.000019849
223	1	-0.000048271	-0.000445353	-0.000234547
224	1	0.000075755	-0.000358998	0.000013490
225	1	-0.000111004	-0.000445150	0.000073726
226	1	-0.000721129	-0.000480043	0.000810936
227	1	-0.000043559	0.000027855	-0.000213574
228	1	0.000088694	-0.000219556	-0.000815129
229	1	0.000025597	0.000040518	0.000107414
230	1	-0.000749896	0.000663848	-0.000727098
231	1	-0.000229121	-0.000112470	0.000173429
232	1	-0.000240673	0.000062210	-0.000685545
233	1	0.000154089	-0.000172053	-0.000142513
234	1	0.000157392	0.000324134	0.000557455
235	1	0.000192025	-0.000070618	0.000001001
236	8	0.000276153	0.000046024	-0.000107001

237	8	0.001982438	0.001519573	0.000780685
238	6	-0.000680640	0.000381882	0.000260999
239	6	0.000884942	0.000695460	0.000307339
240	6	-0.002094967	-0.001452804	-0.000344657
241	6	0.000508406	0.000201374	0.000389394
242	6	-0.000520805	-0.000131979	-0.000102755
243	6	0.000414122	-0.000753855	-0.000081628
244	6	0.000029537	0.000384355	-0.000406489
245	6	-0.000373732	-0.001194695	0.000255193
246	1	-0.000302632	-0.000201637	-0.000276441
247	1	-0.000016441	0.000726253	0.000302262
248	1	0.000111367	-0.000068390	-0.000117140
249	1	0.000134029	0.000365318	-0.000186548
250	1	-0.000026063	0.000339400	-0.000039609
251	1	0.000305753	0.000418992	0.000116985
252	8	-0.000265653	0.000092086	0.000148600
253	8	-0.002103474	0.000901204	-0.001211593
254	6	0.000638295	0.000318367	-0.000482171
255	6	-0.000958459	0.000444041	-0.000492620
256	6	0.002173224	-0.000942382	0.000860780
257	6	-0.000471754	-0.000035220	-0.000452907
258	6	0.000530621	-0.000037877	0.000093458
259	6	-0.000275281	-0.000765610	0.000302498
260	6	-0.000055910	0.000543701	0.000269399
261	6	0.000439425	-0.001197054	0.000142016
262	1	0.000332768	-0.000170360	0.000314264
263	1	-0.000212956	0.000600670	-0.000619290
264	1	-0.000075793	-0.000010395	0.000134828
265	1	-0.000140684	0.000394916	0.000048998
266	1	-0.000002589	0.000312712	-0.000062693
267	1	-0.000320589	0.000344875	-0.000204069
268	8	0.000290540	0.000449813	-0.001081387
269	8	0.000493355	-0.000098530	0.000464215
270	6	0.000072882	0.000108870	0.000576902
271	6	-0.000195724	-0.000115726	-0.000277872
272	6	-0.000313868	0.000621991	0.000103554
273	6	0.000299330	-0.000717072	0.000089359
274	6	0.000182442	-0.000191350	0.000134799
275	6	0.000151867	-0.000539718	-0.000120470
276	6	-0.000076253	0.000351698	-0.000392101
277	6	0.000389269	-0.001219422	0.000079126
278	1	0.000079513	0.000006553	0.000025049
279	1	-0.000521856	0.000698300	0.000027522
280	1	0.000038560	-0.000004255	0.000084665
281	1	-0.000119903	0.000342520	-0.000152080
282	1	-0.000232611	0.000402088	0.000158673
283	1	0.000016156	0.000374797	0.000010407
284	8	-0.000393971	0.000771255	0.000808287
285	8	-0.000479375	-0.000206342	-0.000432304
286	6	0.000146460	-0.000189707	-0.000477046
287	6	0.000091776	0.000073498	0.000226542
288	6	0.000188185	0.000498205	-0.000125326
289	6	-0.000184880	-0.000629599	0.000180676
290	6	-0.000273453	-0.000094229	-0.000025208
291	6	-0.000164490	-0.000470945	0.000238596
292	6	-0.000006264	0.000502196	0.000208142
293	6	-0.000245609	-0.001199589	0.000341468
294	1	-0.000134739	0.000095756	0.000024962

295	1	0.000585991	0.000766449	-0.000236514
296	1	-0.000056882	-0.000002014	-0.000055587
297	1	0.000068237	0.000372448	0.000019827
298	1	0.000222191	0.000320998	-0.000291468
299	1	-0.000044738	0.000328631	-0.000115353
300	1	-0.000287974	0.000638429	-0.000392476
301	1	0.002505408	0.001662955	-0.000159018
302	1	-0.000242895	-0.000671487	0.000388819
303	1	0.002197124	-0.001184288	0.001438149
304	1	0.001816984	0.000567742	-0.002325709
305	1	-0.000624364	-0.001077079	0.000564328
306	1	0.001161976	-0.000655254	-0.000727261
307	1	-0.000336895	0.000935450	-0.000944277
308	1	0.000768789	0.000661931	0.000979387
309	1	-0.001478061	-0.002633834	-0.001552681
310	8	0.000447250	-0.000613167	0.000308799
311	8	-0.002659156	-0.000937468	0.001178954
312	6	-0.000349059	0.000078479	-0.000480484
313	6	-0.001017175	-0.000498878	0.000667390
314	6	0.001428905	0.000074559	-0.001166711
315	6	0.000159908	-0.000129282	0.000477616
316	6	0.000176235	-0.000027022	-0.000245104
317	6	0.000546980	0.000123112	0.000266856
318	6	-0.000473866	0.000170855	-0.000310496
319	6	0.001264506	0.000115201	0.000244787
320	1	0.000577104	0.000271423	-0.000313754
321	1	-0.000674140	-0.000394019	0.000000242
322	1	0.000085719	-0.000065096	-0.000086984
323	1	-0.000334001	-0.000124626	-0.000156295
324	1	-0.000474644	-0.000094917	0.000182284
325	1	-0.000348487	0.000066907	-0.000128972
326	8	0.000134169	0.000991884	-0.000110225
327	8	-0.001565910	0.000564932	-0.002278450
328	6	-0.000271489	-0.000558904	0.000184202
329	6	-0.000711852	0.000495943	-0.001194797
330	6	0.000500171	-0.000088022	0.001677645
331	6	0.000312058	0.000287053	-0.000360726
332	6	-0.000053361	-0.000047273	0.000229919
333	6	0.000658236	0.000286952	-0.000019439
334	6	-0.000533560	-0.000352126	-0.000049727
335	6	0.001148936	0.000208799	0.000523020
336	1	0.000469398	-0.000238558	0.000562700
337	1	-0.000623922	0.000227493	-0.000350285
338	1	-0.000002484	0.000074554	0.000153722
339	1	-0.000327410	0.000015370	-0.000420408
340	1	-0.000326156	-0.000135901	-0.000079284
341	1	-0.000343508	0.000031186	-0.000077832
342	8	0.000773464	0.001588280	-0.001350336
343	8	-0.000689792	0.000785765	0.000838421
344	6	-0.000391791	-0.000935950	0.001228153
345	6	0.000131394	0.000409271	-0.000400724
346	6	-0.000296836	-0.001214638	-0.000225124
347	6	0.000772664	0.000603861	0.000579042
348	6	-0.000119062	-0.000291006	0.000529493
349	6	0.000562135	0.000310130	-0.000263828
350	6	-0.000434508	0.000214600	-0.000303347
351	6	0.001102078	0.000346946	0.000700721
352	1	0.000043115	0.000041846	0.000104681

353	1	-0.000569841	-0.000325535	-0.000597896
354	1	0.000043043	0.000001778	-0.000187889
355	1	-0.000316429	-0.000051535	-0.000079273
356	1	-0.000322403	-0.000045412	-0.000277880
357	1	-0.000282157	-0.000320902	-0.000245081
358	8	0.000162675	-0.001324767	0.001574900
359	8	-0.000246847	-0.000752180	-0.001081855
360	6	0.000075200	0.000767052	-0.001168616
361	6	-0.000132375	-0.000220453	0.000468694
362	6	-0.000364136	0.000909484	0.000063186
363	6	0.000782329	-0.000430951	-0.000081274
364	6	0.000192229	0.000258918	-0.000258608
365	6	0.000345132	-0.000175808	0.000350314
366	6	-0.000473967	-0.000299658	0.000135436
367	6	0.001356700	-0.000100189	-0.000029554
368	1	0.000002314	-0.000086343	-0.000122909
369	1	-0.000842110	0.000187095	0.000168778
370	1	-0.000061041	0.000004731	0.000256248
371	1	-0.000453406	0.000226181	0.000004330
372	1	-0.000320537	-0.000032234	-0.000117181
373	1	-0.000473603	-0.000040839	0.000043546
374	1	-0.000244429	0.000242443	-0.000085260
375	1	-0.001549115	-0.001535930	-0.001036574
376	1	-0.000267717	0.000178316	-0.000004530
377	1	-0.002571008	0.001469567	-0.000376489
378	1	0.000666721	-0.000449185	-0.000523468
379	1	0.000567504	0.000888323	0.000305446
380	1	-0.000548320	-0.000032985	0.000210059
381	1	0.000236285	0.001380946	0.000288230
382	1	-0.000394356	0.000311366	-0.000824273
383	1	-0.001812146	0.001129044	0.001947126
384	8	-0.000163887	-0.001098754	0.001247937
385	8	0.000997978	0.000977329	0.002090818
386	6	0.001256234	0.001201303	-0.001158093
387	6	0.000411184	0.000325504	0.001352873
388	6	-0.000148915	-0.000477723	-0.001664348
389	6	-0.000510251	0.000152163	0.000585057
390	6	-0.000154954	0.000081025	-0.000481720
391	6	-0.000815720	-0.000881541	-0.000003349
392	6	0.000669618	0.000182926	-0.000136592
393	6	-0.001317117	-0.000465280	-0.000388528
394	1	-0.000390514	-0.000293259	-0.000564708
395	1	0.000613099	0.000541663	0.000364291
396	1	0.000028796	0.000046225	-0.000145129
397	1	0.000336513	0.000297780	0.000340760
398	1	0.000349843	0.000004655	0.000105166
399	1	0.000444933	0.000150561	0.000009203
400	8	-0.000124973	-0.000689448	0.000170299
401	8	0.002573067	-0.001176503	-0.000668682
402	6	-0.000035514	0.000208470	-0.000027893
403	6	0.001090809	-0.000461639	-0.000210637
404	6	-0.001391658	0.000278982	0.000915101
405	6	-0.000030135	-0.000244782	-0.000214902
406	6	-0.000156250	0.000009509	0.000127146
407	6	-0.000332327	-0.000179954	-0.000201810
408	6	0.000491982	0.000560683	0.000026969
409	6	-0.001297849	-0.000256705	-0.000161135
410	1	-0.000538671	0.000258570	0.000266338

411	1	0.000691221	-0.000373740	0.000104693
412	1	-0.000086335	-0.000045448	0.000118474
413	1	0.000374709	0.000208149	0.000108076
414	1	0.000340728	-0.000015869	0.000156194
415	1	0.000463094	-0.000040843	-0.000138824
416	8	-0.000936147	0.000787085	0.000815436
417	8	0.000895636	-0.000002497	-0.000914048
418	6	0.000852141	-0.000393034	-0.000335624
419	6	0.000297138	0.000125874	0.000097175
420	6	-0.000281006	-0.000432227	0.000442786
421	6	-0.000165737	-0.000172525	-0.000410691
422	6	-0.000231507	0.000232642	-0.000120912
423	6	-0.000563267	-0.000305642	-0.000285906
424	6	0.000330237	0.000427419	0.000380550
425	6	-0.001081620	-0.000158883	-0.000801755
426	1	-0.000176475	0.000015535	-0.000125920
427	1	0.000515729	0.000054058	0.000622820
428	1	-0.000055032	0.000088266	0.000152142
429	1	0.000353034	-0.000177599	0.000342761
430	1	0.000327538	0.000048211	0.000092308
431	1	0.000329562	0.000123190	0.000268562
432	8	0.000232524	-0.002314933	-0.001014713
433	8	-0.000605128	-0.002216186	0.000949883
434	6	-0.000762653	0.001616913	0.000714292
435	6	0.000407505	-0.001143638	-0.000109323
436	6	0.001226667	0.002911802	-0.000724298
437	6	-0.001442496	-0.001162501	0.000475208
438	6	-0.000396108	0.001084811	0.000221746
439	6	0.000307536	-0.000941824	-0.000346774
440	6	0.000288472	-0.000160836	0.000366480
441	6	-0.001399111	-0.000568223	-0.000195110
442	1	-0.000169494	0.000021995	0.000054414
443	1	0.000785350	0.000198670	-0.000245142
444	1	0.000185333	0.000311239	-0.000264851
445	1	0.000499239	0.000049647	0.000049269
446	1	0.000443510	0.000343161	-0.000040549
447	1	0.000408500	0.000109329	0.000174208
448	1	-0.000023103	0.000145024	0.000861160
449	1	0.000302391	-0.000812336	-0.000133715
450	1	-0.000440305	-0.000029117	-0.000561099
451	1	0.000356707	0.000208753	-0.000274803
452	1	-0.001182183	-0.000279739	0.000637643
453	1	-0.000487112	-0.001454761	-0.002444155
454	1	-0.001071936	-0.000779061	0.000184002
455	1	0.000037313	0.000217324	0.000047512
456	1	0.000096983	0.000766719	0.000265071
457	1	0.000271260	0.000329385	0.000549520
458	6	0.002668961	0.002567073	-0.000833536
459	6	-0.000808199	0.000718810	-0.000449645
460	6	0.000574009	-0.000572646	0.000010921
461	6	-0.000377523	0.000022909	-0.000044911
462	6	0.000317454	-0.000061174	-0.000478207
463	6	0.000064644	-0.000306156	0.000432935
464	1	-0.000361083	0.003040365	-0.005292788
465	1	-0.002825868	-0.002847218	-0.004513928
466	1	0.000040014	-0.002422856	0.005941743
467	1	0.002304702	0.003469176	0.004631524
468	17	-0.005506701	0.003491298	0.004483410

469	1	-0.000355582	-0.000005700	0.000318673
470	1	-0.000709107	-0.000114565	0.000143333
471	1	-0.000327874	-0.000274708	-0.000229513
472	8	-0.002013698	0.001178412	0.001109895
473	1	0.001024582	-0.000218413	-0.000736643
474	1	0.000704347	-0.000771371	-0.000122449
475	8	0.002072740	0.000674788	-0.001241211
476	1	-0.000775002	-0.000627602	0.000278584
477	1	-0.000962405	-0.000019142	0.000688721
478	8	-0.001113512	-0.001252014	-0.001880075
479	1	0.000573716	0.000233530	0.001265845
480	1	0.000332714	0.000732644	0.000439083
481	6	-0.001635451	-0.001951611	-0.000044622
482	8	-0.001559595	0.003109375	-0.003434022
483	1	-0.002626168	-0.005708588	0.001112672

Sum of electronic and thermal Free Energies= -1284.461945
0 imaginary frequency

α-**4f**-TS1

Center Number	Atomic Number	Integrated Forces (Hartrees/Bohr) X	Y	Z
1	6	-0.000543079	-0.000701253	-0.005291424
2	6	-0.000446351	0.001355149	0.003908313
3	7	-0.000862178	-0.000239254	-0.007364639
4	6	0.001259984	-0.001701102	0.003594464
5	6	-0.000401743	0.003421782	0.001252662
6	6	0.001941218	-0.002728452	0.002808530
7	1	0.001047825	-0.006599495	0.000225040
8	1	-0.000852773	-0.001346720	-0.005961523
9	1	-0.001110345	0.006102849	0.003354404
10	1	-0.000803382	-0.001620529	0.007071778
11	1	0.006479807	0.002997975	-0.000250837
12	1	-0.004559569	0.003864164	0.002020972
13	1	-0.001390335	-0.001640753	-0.005895320
14	8	-0.001745647	0.000586277	-0.000460207
15	8	-0.006257722	0.004384526	0.005358530
16	6	0.001899707	-0.001479417	0.000261688
17	6	-0.000799846	0.000442393	0.000683952
18	6	0.002649598	-0.003347748	-0.004444205
19	6	-0.000305881	0.000581352	0.000540213
20	6	0.000337228	-0.000460305	-0.000796955
21	6	-0.000831670	0.001067491	-0.000904618
22	6	0.000643035	-0.000623016	0.000159290
23	6	-0.000547586	0.001056203	-0.000815067
24	1	0.000139053	-0.000017447	-0.000104732
25	1	0.000313471	-0.000416693	0.000690853
26	1	0.000125596	-0.000091144	-0.000154807
27	1	0.000195040	-0.000411192	0.000212007
28	1	0.000182170	-0.000188870	0.000445314
29	1	0.000012455	-0.000253092	0.000141576
30	8	-0.000030682	-0.000072533	0.000390472
31	8	-0.000727369	-0.002775224	0.000036809
32	6	-0.000254469	0.000192201	0.000083457
33	6	-0.000225407	-0.001063062	-0.000190767

34	6	-0.000251126	0.001721475	0.000404397
35	6	-0.000148469	0.000020528	-0.000311605
36	6	0.000181857	0.000041280	0.000060389
37	6	0.000017665	0.000415415	-0.000136495
38	6	0.000236933	-0.000338903	0.000459883
39	6	0.000137784	0.001068042	-0.000721551
40	1	0.000217402	0.000448457	0.000140734
41	1	-0.000485966	-0.000678602	0.000177300
42	1	-0.000064813	0.000119217	0.000074791
43	1	-0.000073411	-0.000486604	0.000089639
44	1	0.000034637	-0.000276020	0.000268653
45	1	-0.000144883	-0.000232474	0.000262203
46	8	0.000396388	0.001055742	0.001662675
47	8	-0.000147110	0.000216453	-0.000071900
48	6	-0.000626811	-0.000151270	-0.001926918
49	6	0.000244410	-0.000227314	0.000877013
50	6	-0.000336390	-0.000477366	-0.000091267
51	6	0.000256102	0.000600713	-0.000285191
52	6	-0.000158352	-0.000126748	-0.000285073
53	6	-0.000293515	0.000502563	0.000431350
54	6	0.000033684	-0.000516338	0.000020502
55	6	0.000460578	0.001063434	-0.000516180
56	1	0.000037128	0.000156947	-0.000415907
57	1	-0.000193403	-0.000285234	0.000595581
58	1	-0.000044541	-0.000044210	-0.000103305
59	1	-0.000091212	-0.000408085	0.000102114
60	1	-0.000056235	-0.000232311	0.000221118
61	1	-0.000322367	-0.000304523	0.000223559
62	8	-0.001519197	-0.001030530	-0.001847517
63	8	-0.000526702	0.000367757	-0.000623003
64	6	0.001095976	0.001151843	0.001308986
65	6	-0.000543752	-0.000545940	-0.000830205
66	6	0.001170201	-0.000882711	0.001292938
67	6	-0.000596256	0.001174795	-0.000843749
68	6	0.000503017	0.000431215	0.000391309
69	6	-0.000223222	-0.000195794	-0.000665235
70	6	-0.000295391	-0.000194393	0.000384921
71	6	-0.000268037	0.001046266	-0.001006443
72	1	-0.000072450	0.000265281	0.000148555
73	1	0.000258502	-0.000796167	0.000198613
74	1	0.000113797	0.000051930	0.000078084
75	1	-0.000007793	-0.000355071	0.000216922
76	1	0.000298109	-0.000402499	0.000311831
77	1	0.000027549	-0.000255081	0.000403287
78	8	0.000042820	0.000286281	0.000354463
79	8	-0.001176048	0.003472053	-0.000051916
80	6	-0.000558425	-0.000279725	-0.000804072
81	6	-0.000407574	0.001267231	0.000322946
82	6	0.000433611	-0.002550857	-0.000787306
83	6	-0.000272259	0.000623160	0.000449043
84	6	0.000042923	-0.000491989	-0.000385512
85	6	0.000373531	-0.000422563	0.000699622
86	6	0.000028259	0.000242228	-0.000455227
87	6	0.000264207	-0.000900252	0.000887318
88	1	0.000409473	-0.000499272	-0.000040470
89	1	-0.000434766	0.000619607	-0.000398775
90	1	-0.000065449	-0.000157664	-0.000095152
91	1	-0.000116996	0.000198982	-0.000373022

92	1	-0.000207182	0.000443376	-0.000136785
93	1	0.000032918	0.000244893	-0.000252613
94	8	-0.000707784	0.000648658	0.000045561
95	8	0.000222569	0.000434522	-0.001128019
96	6	0.000575872	0.000006091	-0.000036477
97	6	-0.000082147	-0.000034755	-0.000166506
98	6	0.000309281	0.000217800	0.000475001
99	6	-0.000324674	-0.000434019	0.000271623
100	6	-0.000269608	-0.000129689	-0.000001260
101	6	-0.000325603	-0.000234819	0.000431875
102	6	0.000073857	0.000579583	-0.000016806
103	6	-0.000583412	-0.001107555	0.000754944
104	1	-0.000152328	0.000056681	0.000162874
105	1	0.000599079	0.000457986	-0.000484567
106	1	0.000125889	0.000042181	0.000099344
107	1	0.000057002	0.000293044	-0.000222166
108	1	0.000197138	0.000422121	-0.000128680
109	1	0.000307630	0.000221014	-0.000386954
110	8	0.000270348	-0.001182901	-0.000089630
111	8	-0.000478094	-0.000508336	0.000145415
112	6	0.000027069	0.000901921	-0.000149555
113	6	-0.000106393	-0.000611259	-0.000403215
114	6	0.000005753	0.000639222	-0.000185078
115	6	-0.000004196	-0.000476957	0.000450570
116	6	0.000147025	-0.000049508	0.000204155
117	6	-0.000222635	-0.000709315	0.000509467
118	6	0.000276415	0.000395720	-0.000269686
119	6	0.000240771	-0.000995910	0.000916648
120	1	0.000114786	0.000225321	0.000320619
121	1	0.000000387	0.000403898	-0.000787840
122	1	-0.000090497	0.000023094	0.000076060
123	1	-0.000270215	0.000215286	-0.000399897
124	1	-0.000084489	0.000382227	-0.000164019
125	1	0.000007903	0.000285190	-0.000272488
126	8	-0.001918016	0.000905797	0.001119722
127	8	-0.000083375	-0.000153398	-0.000123914
128	6	0.001321233	-0.001049066	-0.000624225
129	6	-0.000502762	0.000352853	0.000220545
130	6	0.000496494	0.000456995	-0.000051671
131	6	-0.000560568	-0.000701861	0.000574319
132	6	0.000438352	-0.000294571	-0.000100913
133	6	-0.000411414	0.000189921	0.000426964
134	6	0.000164826	0.000012728	-0.000658348
135	6	-0.000717140	-0.000853401	0.001014311
136	1	0.000052997	-0.000117647	-0.000090427
137	1	0.000221380	0.000735804	-0.000197809
138	1	0.000120458	-0.000089808	-0.000046968
139	1	0.000196338	0.000205630	-0.000419189
140	1	0.000094069	0.000273530	-0.000236973
141	1	0.000369899	0.000347989	-0.000184254
142	8	-0.000483094	-0.000541436	0.000459333
143	1	-0.000006939	-0.000147421	-0.000426521
144	1	0.000035457	0.000452678	0.000202021
145	8	-0.000123868	0.000864740	0.000215295
146	1	-0.000073652	0.000479409	0.000781592
147	1	0.000858489	0.000433893	-0.000268792
148	8	0.002919501	0.001236786	-0.007383892
149	1	-0.003089900	0.000772777	0.004972061

150	1	-0.000940208	-0.001624072	0.001313833
151	8	0.000046302	-0.000532367	-0.000150694
152	1	0.000258204	-0.000606117	-0.000707103
153	1	0.000853811	-0.000392368	0.000578165
154	8	0.001916486	-0.000551057	0.009466596
155	1	-0.000356315	-0.001023003	-0.006020324
156	1	-0.002102206	0.002620139	-0.004122642
157	1	-0.000254672	-0.000402290	0.000252413
158	1	-0.000319950	-0.000407899	-0.000178251
159	1	-0.000393211	0.000238481	-0.000374091
160	1	-0.000296849	-0.000598576	0.000178304
161	1	0.000756791	-0.000789879	-0.000449616
162	8	-0.000410438	0.000178253	-0.000288268
163	8	-0.002245174	-0.001040962	-0.001366092
164	6	0.000516691	-0.000702369	0.000455914
165	6	-0.000765267	-0.000459167	-0.000768281
166	6	0.002026385	0.000995162	0.001580552
167	6	-0.000663990	-0.000376214	-0.000095781
168	6	0.000543312	0.000083651	0.000237276
169	6	-0.000307836	0.000718390	0.000169831
170	6	0.000222559	-0.000126857	-0.000409247
171	6	-0.000069734	0.000762434	0.000909992
172	1	0.000340833	0.000259224	0.000209934
173	1	-0.000107821	-0.000773568	-0.000284482
174	1	0.000020000	0.000143956	-0.000065647
175	1	0.000131938	-0.000268913	-0.000162655
176	1	-0.000187307	-0.000323680	-0.000277410
177	1	0.000030244	-0.000138591	-0.000374794
178	8	-0.000193193	0.000006594	-0.001000205
179	8	-0.000438671	-0.000239942	0.000312956
180	6	0.000427617	0.000385121	0.000918548
181	6	-0.000539955	-0.000472775	-0.000554462
182	6	0.001295782	0.000304478	-0.000181798
183	6	-0.000729710	0.000089802	0.000515681
184	6	0.000312491	-0.000049619	0.000407847
185	6	-0.000252266	-0.000020518	-0.000220163
186	6	-0.000024702	-0.000571507	-0.000077088
187	6	-0.000560348	0.000768996	0.000855100
188	1	0.000163061	0.000021549	0.000206474
189	1	0.000340068	0.000020769	-0.000651907
190	1	-0.000019424	-0.000097416	0.000034042
191	1	0.000144736	-0.000330060	-0.000207093
192	1	0.000024418	-0.000115892	-0.000303522
193	1	0.000386022	-0.000138713	-0.000295422
194	8	0.000198761	-0.000875551	0.000347391
195	8	0.000785020	0.000755744	-0.000450495
196	6	-0.000203784	0.000197996	-0.000179275
197	6	0.000242110	-0.000168988	-0.000131458
198	6	-0.000408681	-0.000392843	0.000317407
199	6	0.000170705	0.000186058	0.000190624
200	6	-0.000048615	-0.000113968	0.000331504
201	6	0.000144892	0.000409726	0.000548527
202	6	0.000079628	-0.000299512	-0.000178340
203	6	-0.000222718	0.000435511	0.001087091
204	1	-0.000055145	0.000048969	0.000027944
205	1	-0.000201981	-0.000316175	-0.000719401
206	1	0.000081339	0.000017268	0.000049919
207	1	0.000015664	-0.000026310	-0.000463810

208	1	0.000205867	-0.000143070	-0.000294809
209	1	-0.000007492	-0.000230260	-0.000284530
210	8	-0.001446943	0.000833312	0.000815718
211	8	-0.000825548	-0.000079526	0.000475131
212	6	0.001773672	0.000022968	-0.000900276
213	6	-0.000992874	0.000089230	-0.000076276
214	6	0.000805483	-0.000288127	-0.000129964
215	6	-0.000590103	0.000361798	0.000361144
216	6	-0.000048998	0.000321664	-0.000019072
217	6	-0.000827032	0.000583735	0.000427119
218	6	0.000339517	-0.000016453	-0.000443243
219	6	-0.000471107	0.000987665	0.000710452
220	1	0.000351340	-0.000267647	-0.000071602
221	1	0.000541142	-0.000499698	-0.000368524
222	1	-0.000066115	-0.000012690	0.000014639
223	1	0.000252069	-0.000530471	-0.000063928
224	1	-0.000003183	-0.000292873	-0.000215641
225	1	0.000187898	-0.000214045	-0.000270172
226	1	0.000578428	0.000258638	-0.000659491
227	1	0.000173818	-0.000073078	0.000125397
228	1	0.000203318	-0.000708938	0.000581454
229	1	0.000107030	0.000149135	-0.000478680
230	1	0.000911940	0.000178412	0.001142708
231	1	0.000085347	0.000000278	-0.000105073
232	1	0.000300155	-0.000251539	0.000801189
233	1	0.000119425	-0.000429088	-0.000103945
234	1	0.000039057	0.000717503	-0.000191427
235	1	0.000645125	0.000244812	0.000199775
236	8	-0.000326372	-0.000097789	0.000097628
237	8	-0.002788438	0.000955861	0.000272028
238	6	0.000510326	0.000494207	-0.000015762
239	6	-0.001190640	0.000497140	0.000235384
240	6	0.002911748	-0.000879915	-0.000539776
241	6	-0.000776758	0.000412058	-0.000216342
242	6	0.000625739	0.000022822	0.000067008
243	6	-0.000142927	-0.000582096	-0.000404128
244	6	-0.000174122	-0.000029019	0.000606640
245	6	0.000567803	-0.000578518	-0.001008135
246	1	0.000449919	-0.000287743	-0.000037561
247	1	-0.000176219	0.000642295	0.000269500
248	1	-0.000015231	-0.000172971	0.000073808
249	1	-0.000435401	0.000262862	0.000158605
250	1	-0.000174365	0.000122901	0.000415026
251	1	-0.000026144	0.000240144	0.000238011
252	8	-0.000516694	-0.000000756	0.000468725
253	8	0.001290028	-0.000217540	0.000541475
254	6	0.000224047	0.000055708	0.000067091
255	6	0.000207830	-0.000177270	0.000363001
256	6	-0.000756179	-0.000189978	-0.000173909
257	6	0.000197362	-0.000267205	-0.000385471
258	6	-0.000120741	0.000023794	-0.000225499
259	6	0.000024978	-0.000290079	-0.000467807
260	6	-0.000241807	0.000402738	0.000198475
261	6	-0.000006594	-0.000917011	-0.001051995
262	1	-0.000155432	0.000150004	-0.000217134
263	1	-0.000061599	0.000164841	0.000859308
264	1	0.000067355	0.000084410	-0.000071453
265	1	-0.000008890	0.000397869	0.000225010

266	1	-0.000130310	0.000220845	0.000337078
267	1	0.000226513	0.000234896	0.000451628
268	8	-0.000462127	-0.000089484	0.000252058
269	8	-0.000437712	0.000474324	-0.000836080
270	6	0.000587244	0.000088740	0.000234119
271	6	-0.000050069	0.000326928	0.000111792
272	6	0.000128513	-0.000317818	0.000348902
273	6	-0.000104447	0.000003632	-0.000525804
274	6	0.000016437	-0.000441146	0.000119623
275	6	-0.000347915	-0.000510505	-0.000615771
276	6	0.000200910	0.000090750	0.000572695
277	6	-0.000105000	-0.000952252	-0.000923155
278	1	-0.000048383	-0.000078577	-0.000099335
279	1	0.000296773	0.000602127	0.000507908
280	1	-0.000056886	-0.000016718	-0.000043382
281	1	0.000042127	0.000193255	0.000388069
282	1	0.000114951	0.000436789	0.000193774
283	1	-0.000107040	0.000273003	0.000222746
284	8	-0.000386735	0.001602196	-0.000242833
285	8	0.000209820	-0.000571347	0.000322714
286	6	0.000447896	-0.000914745	0.000316032
287	6	-0.000267246	0.000308750	-0.000058143
288	6	-0.000194411	0.000260224	0.000221517
289	6	0.000305817	-0.000351351	-0.000637638
290	6	0.000366892	-0.000157686	0.000044767
291	6	-0.000016528	0.000064780	-0.000438040
292	6	0.000039116	0.000459279	0.000258548
293	6	0.000397133	-0.000718902	-0.001091243
294	1	0.000134903	-0.000020278	-0.000096686
295	1	-0.000561860	0.000266168	0.000509051
296	1	0.000077340	0.000005518	0.000011845
297	1	-0.000010586	0.000245410	0.000338271
298	1	-0.000163648	0.000292481	0.000215160
299	1	-0.000230879	0.000061675	0.000422417
300	1	0.000101145	0.000166032	0.000512998
301	1	-0.002618539	0.000148431	0.001085605
302	1	0.000304229	-0.000178916	-0.000502282
303	1	-0.002030266	-0.000638078	-0.002193410
304	1	-0.000788384	-0.000644635	0.000947500
305	1	0.000895033	-0.000450221	-0.001217205
306	1	-0.000725505	-0.001205871	0.000077763
307	1	0.000095035	0.000289508	0.001154268
308	1	-0.001193073	0.000711981	-0.000333625
309	1	0.002342615	-0.002159641	-0.000368911
310	8	-0.000465471	0.000185006	-0.000572802
311	8	0.002596754	0.001120408	-0.001458285
312	6	0.000703533	-0.000599363	0.000466882
313	6	0.000936189	0.000567649	-0.000818651
314	6	-0.001301618	-0.001335858	0.000944004
315	6	0.000050070	0.000319164	-0.000485345
316	6	-0.000191345	-0.000396448	0.000219424
317	6	-0.000839993	0.000294156	-0.000172041
318	6	0.000453872	0.000013023	0.000356818
319	6	-0.001252555	-0.000208385	-0.000207567
320	1	-0.000526200	-0.000197428	0.000422467
321	1	0.000847859	-0.000068814	-0.000248606
322	1	-0.000042254	-0.000123479	0.000061798
323	1	0.000376062	-0.000053274	0.000075612

324	1	0.000385095	0.000160231	-0.000137755
325	1	0.000310412	0.000105855	0.000186323
326	8	-0.000288758	0.000340160	0.000471658
327	8	0.001666965	-0.000509974	0.002485171
328	6	0.000435414	-0.000068971	-0.000439703
329	6	0.000686456	-0.000100791	0.001324103
330	6	-0.000602413	0.000660349	-0.001541597
331	6	-0.000291345	-0.000121757	0.000392905
332	6	0.000036076	0.000061465	-0.000220703
333	6	-0.000601161	-0.000053337	0.000071886
334	6	0.000651579	-0.000191123	-0.000204788
335	6	-0.001265377	0.000217835	-0.000272764
336	1	-0.000469995	0.000087168	-0.000671821
337	1	0.000532744	0.000126410	0.000533428
338	1	-0.000026290	0.000116862	-0.000095112
339	1	0.000392452	-0.000079648	-0.000038824
340	1	0.000315765	0.000067833	0.000090267
341	1	0.000385794	-0.000195607	0.000315640
342	8	-0.001244139	0.000287929	0.002204621
343	8	0.000136914	0.001415902	-0.000054662
344	6	0.000532929	0.000321455	-0.001630746
345	6	-0.000246851	-0.000041117	0.000642134
346	6	0.000703619	-0.001145257	-0.000498355
347	6	-0.001042947	0.000620196	-0.000193478
348	6	0.000016548	0.000040228	-0.000552314
349	6	-0.000577715	0.000020556	0.000458165
350	6	0.000350629	0.000117546	0.000408666
351	6	-0.001289794	0.000365046	-0.000349946
352	1	0.000048585	0.000114621	0.000005583
353	1	0.000728119	-0.000414567	0.000261330
354	1	-0.000012135	-0.000167242	0.000127901
355	1	0.000369930	0.000019866	0.000044566
356	1	0.000399231	-0.000091578	0.000222128
357	1	0.000436908	-0.000270775	-0.000010596
358	8	-0.000135943	-0.000037787	-0.001636806
359	8	0.000681068	-0.000867124	0.000583475
360	6	0.000104792	-0.000227590	0.000994920
361	6	0.000307224	0.000340073	-0.000282183
362	6	0.000011323	0.000504711	0.000247723
363	6	-0.000664785	-0.000316999	-0.000090698
364	6	-0.000107259	-0.000094906	0.000193202
365	6	-0.000508763	-0.000059994	-0.000352206
366	6	0.000567715	-0.000008599	-0.000233552
367	6	-0.001222417	-0.000467581	-0.000183700
368	1	-0.000197938	-0.000281228	-0.000038714
369	1	0.000720731	0.000506457	0.000049088
370	1	0.000034454	0.000118446	-0.000131868
371	1	0.000337503	0.000327251	0.000209628
372	1	0.000300430	0.000018224	0.000091583
373	1	0.000412207	0.000131244	-0.000026337
374	1	-0.000046687	0.000153300	-0.000424138
375	1	0.001458021	-0.000771868	-0.000055365
376	1	0.000173504	0.000122276	0.000005418
377	1	0.001046003	0.000819575	0.000386538
378	1	0.000111227	-0.000574704	-0.000663431
379	1	-0.000273863	0.000727891	0.000219535
380	1	0.000639583	0.000479001	-0.000262889
381	1	-0.000254444	0.000519645	0.000360593

382	1	0.001298141	0.000283879	-0.000265487
383	1	0.001188805	0.002410884	-0.000588918
384	8	-0.000633891	-0.000320376	0.000548616
385	8	-0.000702581	0.000371817	-0.000085932
386	6	0.000456277	0.000332251	-0.000421692
387	6	-0.000805986	0.000111373	0.000305562
388	6	-0.000173356	0.000365882	-0.000107816
389	6	0.000799248	-0.000268003	0.000289374
390	6	0.000209106	0.000021971	-0.000354204
391	6	0.000078820	-0.000062401	0.000254795
392	6	-0.000426039	-0.000300154	-0.000268896
393	6	0.001278632	-0.000082516	0.000663569
394	1	0.000236889	-0.000192652	0.000033474
395	1	-0.000785064	0.000313176	-0.000035054
396	1	-0.000018468	0.000034009	0.000041671
397	1	-0.000351896	-0.000110477	-0.000227800
398	1	-0.000427550	0.000008563	-0.000086450
399	1	-0.000402507	0.000260102	-0.000272658
400	8	0.000334640	-0.000076598	-0.000442125
401	8	-0.001004323	-0.000513661	0.000223095
402	6	-0.000207892	0.000073788	0.000136519
403	6	-0.000609260	-0.000088944	0.000030054
404	6	0.000448800	-0.000062588	-0.000244695
405	6	0.000296565	0.000087404	0.000208856
406	6	0.000086737	0.000146647	-0.000047048
407	6	0.000452072	0.000006020	0.000262122
408	6	-0.000516342	0.000112161	-0.000151523
409	6	0.001308290	0.000361743	0.000199407
410	1	0.000139592	0.000162633	0.000011306
411	1	-0.000609447	-0.000361388	-0.000304467
412	1	0.000009643	-0.000003291	-0.000066046
413	1	-0.000411135	-0.000001285	-0.000008384
414	1	-0.000357926	-0.000082568	-0.000167975
415	1	-0.000399867	-0.000302222	0.000014628
416	8	-0.000271024	-0.000183492	0.000810677
417	8	-0.001084334	-0.000859474	0.000681025
418	6	-0.000177509	0.000755444	-0.000452883
419	6	-0.000764632	-0.000340714	0.000053260
420	6	0.000337158	0.000179928	-0.000546286
421	6	0.000386132	-0.000304086	0.000286482
422	6	0.000155977	0.000036108	0.000293279
423	6	0.000495957	-0.000282994	0.000603602
424	6	-0.000492401	0.000359548	-0.000029954
425	6	0.001210081	-0.000164247	0.000654131
426	1	0.000356230	0.000132979	-0.000042512
427	1	-0.000480631	0.000138406	-0.000627818
428	1	0.000015368	0.000142807	-0.000071624
429	1	-0.000401290	0.000133191	-0.000167452
430	1	-0.000358762	-0.000071937	-0.000425571
431	1	-0.000340030	-0.000019798	-0.000083253
432	8	0.000195786	-0.000218457	-0.000543741
433	8	-0.000898242	0.000100830	-0.000177267
434	6	0.000045243	-0.000354614	0.000153022
435	6	-0.000637709	0.000026191	-0.000455726
436	6	0.000089833	-0.000125762	0.000644064
437	6	0.000433258	0.000213323	-0.000051108
438	6	0.000158900	0.000095657	-0.000022550
439	6	0.000443569	0.000062141	-0.000066126

440	6	-0.000361237	-0.000060241	-0.000295421
441	6	0.001322488	0.000116737	0.000188772
442	1	0.000367529	-0.000023731	-0.000037771
443	1	-0.000701628	-0.000267046	0.000192141
444	1	0.000014283	-0.000107700	0.000060903
445	1	-0.000455211	0.000031584	0.000123525
446	1	-0.000385403	-0.000008694	-0.000174456
447	1	-0.000400355	-0.000157821	-0.000105976
448	1	-0.000469729	0.000397255	-0.000166790
449	1	0.000941176	-0.000999717	-0.000456041
450	1	0.000419039	-0.000339520	-0.000000007
451	1	-0.000300233	-0.000084668	0.000199305
452	1	0.001251821	0.000542729	-0.000725701
453	1	0.001392955	-0.002512187	0.001162626
454	1	0.000929617	-0.000221068	-0.000630954
455	1	0.000433548	-0.000300167	0.000014196
456	1	-0.000146288	0.000389601	0.000286837
457	1	-0.000036778	-0.000189630	-0.000348780
458	6	-0.001655562	0.003534923	0.000321286
459	6	-0.000160655	0.000736066	-0.000158894
460	6	0.000380048	-0.000099588	0.000147424
461	6	0.001208053	-0.005697880	0.000013295
462	6	-0.000420365	-0.000046268	-0.000566760
463	6	-0.000410974	-0.000371085	-0.000026540
464	1	0.001102520	0.004504853	0.005151335
465	1	0.002874682	-0.003359334	0.005109296
466	1	-0.000600047	-0.004504316	-0.004677260
467	1	-0.002297429	0.003147769	-0.005259427
468	17	-0.001159255	0.000804534	-0.001102830
469	1	0.000235744	0.000072894	-0.000274277
470	1	0.000645967	0.000433933	-0.000349339
471	1	0.000513763	-0.000159857	-0.000093499
472	8	0.001273927	0.002080106	0.000182411
473	1	-0.000565694	-0.000783950	0.000176021
474	1	-0.000361909	-0.000837709	-0.000413894
475	8	-0.001520376	-0.001059567	0.001521501
476	1	0.000728155	0.000109366	-0.000935681
477	1	0.000519284	0.000825507	-0.000244417
478	8	0.001860498	-0.001466403	0.000518643
479	1	-0.000785105	0.000590238	-0.000618926
480	1	-0.000681956	0.000619504	0.000189182
481	6	0.004577874	0.002554055	-0.001238165
482	8	-0.001475882	-0.000345596	0.003786552
483	7	0.001251112	-0.001940191	-0.000782370
484	8	-0.001377163	0.000410107	-0.002763700
485	8	0.000790276	-0.000074493	0.003257921

Sum of electronic and thermal Free Energies= -1488.930626
1 imaginary frequency: -81.02

β-**4f**-TS1

Center Number	Atomic Number	Integrated Forces (Hartrees/Bohr) X	Y	Z
1	6	-0.001989774	-0.003565863	-0.004296270
2	6	0.000536033	0.005932948	-0.002245236

3	7	0.004050683	0.003188859	0.005673449
4	6	-0.000724181	-0.003508224	-0.003080029
5	6	0.001784912	0.001746769	0.002449098
6	6	-0.002471415	-0.004860613	-0.001490592
7	1	-0.000148940	-0.005887618	0.003353437
8	1	0.002820745	0.001982803	0.006075031
9	1	-0.002355124	0.004510248	-0.005664613
10	1	-0.000685667	-0.004024665	-0.004176922
11	1	-0.000522065	0.002805225	-0.004984995
12	1	0.006464379	0.000930638	0.002218332
13	1	-0.004297540	0.001973173	0.004159851
14	8	0.002803549	0.000809869	0.000091459
15	8	0.007020268	-0.000812685	-0.003893796
16	6	-0.002778817	-0.001068986	0.000792832
17	6	0.001497044	-0.000277372	-0.000099709
18	6	-0.003562840	0.001034066	0.004057673
19	6	0.000750425	0.000178120	-0.000054320
20	6	-0.000222850	0.000316028	0.000508810
21	6	0.001255447	0.000863449	0.000241314
22	6	-0.000534799	-0.000354516	0.000298359
23	6	0.000636811	0.001153554	0.000111308
24	1	-0.000440172	-0.000076188	-0.000008054
25	1	-0.000615153	-0.000715038	-0.000329890
26	1	-0.000119285	0.000033376	0.000120364
27	1	-0.000032928	-0.000272310	-0.000006948
28	1	-0.000244186	-0.000357111	0.000059126
29	1	-0.000226508	-0.000360874	-0.000321782
30	8	-0.000900790	0.000322452	0.000147665
31	8	0.000263037	-0.001884107	0.001282590
32	6	0.001362379	-0.000334979	-0.000482439
33	6	-0.000397091	-0.000634412	0.000571992
34	6	0.000503731	0.000819123	-0.000780001
35	6	-0.000120305	0.000156850	0.000044268
36	6	0.000085202	0.000232542	-0.000110958
37	6	-0.000539685	0.000374002	-0.000168098
38	6	-0.000270194	-0.000404202	-0.000140110
39	6	-0.000015742	0.001122485	-0.000217037
40	1	-0.000025812	0.000174368	-0.000352236
41	1	0.000336929	-0.000595348	0.000260647
42	1	0.000096111	0.000060253	-0.000128098
43	1	0.000123400	-0.000299279	-0.000009050
44	1	0.000078462	-0.000391229	0.000344876
45	1	-0.000077463	-0.000319540	0.000006912
46	8	-0.000212276	0.000562360	-0.000298930
47	8	-0.000507066	0.000742510	0.000079812
48	6	0.000265165	-0.000173654	-0.000017076
49	6	-0.000369741	-0.000136719	0.000054090
50	6	0.001024462	-0.000669428	0.000048252
51	6	-0.000346772	0.000575687	0.000245735
52	6	-0.000017578	0.000180243	-0.000034873
53	6	0.000117882	0.000522970	0.000190133
54	6	-0.000153855	-0.000363128	0.000084689
55	6	-0.000435368	0.001115858	-0.000100606
56	1	0.000025800	0.000068365	-0.000008417
57	1	0.000245396	-0.000724883	-0.000301903
58	1	0.000031947	0.000003648	0.000094968
59	1	0.000376151	-0.000369217	-0.000010230
60	1	0.000085753	-0.000331357	0.000132941

61	1	0.000066436	-0.000311758	-0.000033282
62	8	0.001569271	0.000200319	0.001243602
63	8	0.001669846	0.000686803	-0.000018001
64	6	-0.001028454	0.000198700	-0.000944826
65	6	0.000965082	0.000447960	0.000558010
66	6	-0.002456946	-0.001333496	-0.000025245
67	6	0.001153124	0.001213208	-0.000313428
68	6	-0.000552869	0.000262134	-0.000408420
69	6	0.000449143	0.000107280	0.000190743
70	6	0.000190509	-0.000355388	-0.000265041
71	6	0.000650438	0.001236154	0.000147442
72	1	-0.000166078	-0.000020013	-0.000142559
73	1	-0.000335872	-0.000787725	0.000367357
74	1	-0.000170402	-0.000040200	-0.000062950
75	1	-0.000145485	-0.000395140	-0.000150200
76	1	-0.000101983	-0.000352614	0.000029935
77	1	-0.000386275	-0.000352278	0.000019341
78	8	0.000127895	0.000114199	-0.000295647
79	8	0.001302501	0.002375904	-0.001702853
80	6	0.000362330	0.000249149	0.000576412
81	6	0.000535125	0.000571338	-0.000787514
82	6	-0.000481704	-0.001040369	0.001624377
83	6	0.000305844	-0.000136011	-0.000469921
84	6	-0.000192523	0.000006389	0.000316515
85	6	-0.000125889	-0.000700086	-0.000179369
86	6	-0.000169078	0.000543434	0.000169255
87	6	-0.000241079	-0.001264697	-0.000067961
88	1	-0.000399421	-0.000297919	0.000368469
89	1	0.000476305	0.000639426	-0.000104050
90	1	0.000085707	-0.000070187	0.000131294
91	1	0.000198822	0.000397789	-0.000189500
92	1	-0.000017262	0.000370586	0.000044625
93	1	0.000105669	0.000354217	0.000126629
94	8	0.000500084	0.000095639	-0.000097002
95	8	-0.000227854	0.001085778	0.000810005
96	6	-0.000500568	0.000460357	-0.000205355
97	6	-0.000208545	-0.000301502	0.000312266
98	6	-0.000226761	-0.000063879	-0.000578382
99	6	0.000198486	-0.000496468	0.000089109
100	6	0.000262922	-0.000077885	-0.000066727
101	6	0.000327306	-0.000708862	-0.000081077
102	6	-0.000065063	0.000476770	-0.000387419
103	6	0.000437986	-0.001380918	0.000123847
104	1	0.000268549	-0.000007642	-0.000245558
105	1	-0.000478134	0.000689892	0.000111303
106	1	-0.000167504	-0.000037740	-0.000098167
107	1	-0.000260277	0.000465623	0.000189972
108	1	-0.000011122	0.000384138	-0.000056636
109	1	-0.000127517	0.000429099	-0.000139412
110	8	-0.000280280	-0.000906111	0.000921766
111	8	0.000431184	-0.000505131	0.000122172
112	6	0.000028921	0.000862589	-0.000488520
113	6	0.000165645	-0.000223941	0.000866605
114	6	-0.000089917	0.000569610	-0.000340525
115	6	0.000036127	-0.000614110	0.000025366
116	6	-0.000245371	-0.000159074	-0.000124615
117	6	0.000202649	-0.000954808	0.000084017
118	6	-0.000213277	0.000538922	-0.000044878

119	6	-0.000352817	-0.001318757	-0.000056919
120	1	-0.000106295	0.000003590	-0.000443961
121	1	0.000091060	0.000786459	0.000348485
122	1	0.000075645	-0.000024838	-0.000077514
123	1	0.000124321	0.000368031	-0.000091950
124	1	0.000024912	0.000390757	0.000011668
125	1	0.000311448	0.000388411	0.000175797
126	8	0.001928173	-0.000233820	-0.001318718
127	8	0.000455988	-0.000658095	-0.000261154
128	6	-0.001239277	-0.000097406	0.001067055
129	6	0.000487249	-0.000148356	-0.000983601
130	6	-0.000900696	0.001163264	0.000260923
131	6	0.000462386	-0.001213489	0.000067029
132	6	-0.000493352	-0.000047498	0.000310402
133	6	0.000318509	-0.000331744	-0.000404358
134	6	0.000034722	0.000517587	0.000401191
135	6	0.000488368	-0.001408595	-0.000126077
136	1	0.000033824	-0.000006892	0.000419959
137	1	-0.000129395	0.000891427	-0.000350169
138	1	-0.000115124	0.000010962	0.000067090
139	1	-0.000110110	0.000436617	0.000120882
140	1	-0.000054063	0.000380843	0.000007461
141	1	-0.000345998	0.000400379	-0.000123493
142	8	0.001390070	0.001532116	0.001037815
143	1	-0.000711612	-0.000938294	-0.000136578
144	1	-0.000411746	-0.000373527	-0.000795811
145	8	0.000166244	0.000379129	-0.000551475
146	1	-0.000896964	0.000709345	-0.000177810
147	1	-0.000005048	0.000027307	-0.000953490
148	8	-0.002757688	0.007644846	0.001229338
149	1	0.003493623	-0.004412709	-0.002906080
150	1	-0.000124554	-0.002341693	0.001306962
151	8	-0.000323914	0.000247206	0.000197201
152	1	-0.000269135	-0.000246760	0.001217571
153	1	-0.000864659	-0.000852349	0.000053664
154	8	-0.002521081	-0.005709965	-0.006924619
155	1	0.002696549	0.004303825	0.001801671
156	1	0.000572907	0.002583261	0.005490391
157	1	0.000391040	-0.000866357	-0.001051070
158	1	0.000175378	-0.000165450	0.000119697
159	1	-0.000019374	0.000186602	-0.000295355
160	1	0.000191336	-0.000795777	0.000126236
161	1	-0.000246925	-0.000153428	-0.000005634
162	8	0.000341436	0.000223974	0.000017523
163	8	0.002381908	-0.000317780	0.001885454
164	6	-0.000725664	-0.000613812	0.000342503
165	6	0.001018954	-0.000189096	0.000747793
166	6	-0.002259173	0.000100958	-0.001895515
167	6	0.000762486	-0.000367884	0.000297159
168	6	-0.000654781	-0.000074596	-0.000262613
169	6	0.000463059	0.000326487	-0.000840593
170	6	-0.000187702	0.000235163	0.000468844
171	6	-0.000000276	-0.000063906	-0.001273534
172	1	-0.000394804	0.000212999	-0.000329538
173	1	-0.000006565	-0.000400253	0.000735609
174	1	0.000051185	0.000126699	-0.000068044
175	1	-0.000121014	-0.000025807	0.000331924
176	1	0.000188833	-0.000116386	0.000431463

177	1	-0.000010528	0.000122511	0.000392813
178	8	0.000482019	0.001113611	0.001398410
179	8	-0.000255487	-0.000288938	0.000444963
180	6	-0.000546613	-0.000779648	-0.000553438
181	6	0.000030766	0.000496723	0.000699073
182	6	-0.000083660	0.000075551	0.000054777
183	6	0.000235829	0.000064982	-0.000689530
184	6	-0.000103937	-0.000252445	-0.000084235
185	6	0.000272005	0.000259871	-0.000308741
186	6	0.000015861	-0.000296039	0.000531594
187	6	0.000598278	0.000066613	-0.001224281
188	1	0.000108686	-0.000283900	-0.000434085
189	1	-0.000301693	0.000543626	0.000713520
190	1	-0.000043380	-0.000080481	-0.000002250
191	1	-0.000062428	0.000029449	0.000349875
192	1	-0.000410739	0.000094350	0.000337596
193	1	-0.000152456	-0.000105739	0.000363830
194	8	0.000135628	-0.001062165	0.000836727
195	8	-0.000526338	0.001162238	0.000136672
196	6	-0.000326815	0.000566156	-0.000281724
197	6	0.000301237	-0.000011813	0.000197988
198	6	-0.000050519	-0.000585789	0.000165181
199	6	0.000117543	0.000049693	-0.000633931
200	6	-0.000248646	-0.000050704	-0.000121178
201	6	0.000073259	-0.000155350	-0.000404560
202	6	-0.000207168	-0.000177958	0.000494894
203	6	0.000176289	-0.000178966	-0.001275249
204	1	-0.000149730	-0.000090815	-0.000167328
205	1	0.000345788	0.000175489	0.000820502
206	1	-0.000084940	-0.000017017	-0.000038613
207	1	-0.000001390	-0.000063320	0.000363449
208	1	0.000042814	0.000246867	0.000428551
209	1	-0.000160146	0.000029452	0.000344514
210	8	0.000228325	0.000471360	0.000296941
211	8	0.000552844	-0.000638948	-0.000121289
212	6	-0.000221788	-0.000188480	0.000198593
213	6	-0.000160275	0.000017676	-0.000100376
214	6	-0.000148246	0.000087201	0.000547776
215	6	0.000319492	-0.000071926	-0.000669997
216	6	0.000224219	0.000195747	-0.000026283
217	6	0.000315155	0.000096778	-0.000600254
218	6	-0.000269725	0.000343041	0.000450878
219	6	0.000579761	0.000120526	-0.001206064
220	1	0.000085025	0.000040180	-0.000024000
221	1	-0.000629423	-0.000230293	0.000607091
222	1	0.000070779	-0.000008853	0.000027495
223	1	-0.000049205	-0.000036869	0.000358009
224	1	-0.000180253	0.000072626	0.000330257
225	1	-0.000287682	-0.000266144	0.000329539
226	1	-0.000403574	0.000698871	0.000270651
227	1	-0.000445453	-0.000548042	0.000574360
228	1	-0.000286992	-0.001055983	-0.000048935
229	1	-0.000050514	0.000429568	0.000051832
230	1	-0.000862884	-0.000414413	-0.000748870
231	1	-0.000026494	0.000098943	-0.000174856
232	1	-0.000919503	-0.000873385	-0.000348992
233	1	-0.000090493	-0.000294767	0.000336482
234	1	-0.000157242	0.000416314	-0.000230848

235	1	-0.001235727	-0.000554606	-0.000746605
236	8	0.000366321	0.000367848	-0.000581371
237	8	0.002887122	0.000444396	-0.001011688
238	6	-0.000336475	0.000087943	-0.000116962
239	6	0.001272845	0.000353376	-0.000826773
240	6	-0.002974480	-0.000164283	0.001122205
241	6	0.000936163	0.000502487	-0.000087355
242	6	-0.000519619	0.000125427	-0.000039752
243	6	0.000159176	-0.000161681	0.000678315
244	6	0.000144148	-0.000485310	-0.000520914
245	6	-0.000511575	0.000276394	0.001140380
246	1	-0.000526203	-0.000269135	0.000404402
247	1	0.000239547	0.000361242	-0.000619978
248	1	-0.000051995	-0.000156677	0.000044133
249	1	0.000026132	-0.000005739	-0.000306003
250	1	0.000390637	0.000053269	-0.000315777
251	1	0.000130120	-0.000141038	-0.000362374
252	8	0.000514759	-0.000189509	-0.000197023
253	8	-0.001312314	-0.000254399	-0.000066232
254	6	-0.000192839	-0.000054287	-0.000308019
255	6	-0.000320763	-0.000465798	0.000231009
256	6	0.000736246	-0.000154528	-0.000223751
257	6	-0.000126596	0.000111288	0.000686006
258	6	0.000148094	0.000202312	0.000090507
259	6	0.000025878	0.000114134	0.000725321
260	6	0.000374433	0.000082956	-0.000494342
261	6	-0.000020413	0.000117592	0.001450153
262	1	0.000176747	0.000324160	-0.000011261
263	1	-0.000084361	-0.000443880	-0.000791290
264	1	-0.000006761	0.000104621	-0.000019584
265	1	0.000027408	0.000073389	-0.000417310
266	1	0.000123774	-0.000061931	-0.000381037
267	1	-0.000228724	-0.000168342	-0.000466922
268	8	0.000327031	-0.000103112	0.000296738
269	8	0.001104698	0.000982600	0.000180465
270	6	-0.000696617	-0.000262589	-0.000756930
271	6	0.000347972	0.000076736	-0.000007649
272	6	-0.000504959	-0.000325874	-0.000212581
273	6	0.000273767	0.000370140	0.000510341
274	6	-0.000167292	-0.000514398	0.000206773
275	6	0.000319890	0.000127789	0.001168026
276	6	-0.000382604	-0.000405787	-0.000567823
277	6	0.000213358	-0.000036645	0.001360155
278	1	0.000020867	0.000095124	-0.000035657
279	1	-0.000365764	0.000187321	-0.000886563
280	1	0.000024521	-0.000004397	0.000017812
281	1	0.000081228	0.000005590	-0.000319160
282	1	-0.000050359	-0.000104987	-0.000434553
283	1	-0.000124183	0.000238962	-0.000442292
284	8	0.000772014	0.001619650	-0.000837589
285	8	-0.000135587	-0.000039198	0.000272515
286	6	-0.000986521	-0.001002127	0.000236166
287	6	0.000455353	0.000463557	0.000084752
288	6	0.000154824	-0.000449337	-0.000859688
289	6	-0.000309186	0.000482468	0.000999631
290	6	-0.000597846	-0.000415589	-0.000107699
291	6	0.000364935	0.000460667	0.000278235
292	6	-0.000226818	0.000149730	-0.000563567

293	6	-0.000188782	0.000298188	0.001346354
294	1	-0.000127541	0.000000506	0.000028261
295	1	0.000591132	-0.000279835	-0.000642463
296	1	-0.000075026	-0.000028308	-0.000025655
297	1	0.000191135	-0.000333067	-0.000421045
298	1	-0.000030135	-0.000048236	-0.000431570
299	1	0.000115743	0.000011099	-0.000328291
300	1	-0.000091133	-0.000568164	-0.000715321
301	1	0.002867591	-0.000975225	-0.001077320
302	1	-0.000530849	-0.000054163	0.000512295
303	1	0.002024356	0.000543350	0.001881688
304	1	0.000806134	-0.001192911	-0.000582612
305	1	-0.000823149	0.000606053	0.001038663
306	1	0.000494359	-0.001161068	0.000729809
307	1	-0.000182952	-0.000710595	-0.001047390
308	1	0.001176280	0.000602568	-0.000213219
309	1	-0.003290231	-0.001683086	0.000566427
310	8	0.000418223	0.000609101	0.000468424
311	8	-0.002577959	0.002487915	0.000548009
312	6	-0.000763484	-0.000718868	-0.000112236
313	6	-0.000904672	0.001164356	0.000384743
314	6	0.001272338	-0.002174362	-0.000107586
315	6	-0.000066135	0.000741578	0.000294281
316	6	0.000019856	-0.000600224	0.000001138
317	6	0.000912417	0.000347966	0.000007442
318	6	-0.000506311	-0.000135904	-0.000275923
319	6	0.001228253	-0.000096655	0.000218707
320	1	0.000578073	-0.000508944	-0.000185677
321	1	-0.000805912	0.000139868	0.000313699
322	1	0.000021425	-0.000161023	0.000077747
323	1	-0.000421525	0.000249380	0.000012421
324	1	-0.000300148	-0.000022678	-0.000175168
325	1	-0.000374796	-0.000060393	0.000004466
326	8	0.000655630	0.000236487	-0.000594761
327	8	-0.001705681	-0.001404477	-0.001399186
328	6	-0.000480272	0.000097971	0.000373510
329	6	-0.000615052	-0.000731498	-0.000790429
330	6	0.000684507	0.001190718	0.000383022
331	6	0.000436095	-0.000150610	-0.000100390
332	6	0.000003339	0.000065743	0.000202248
333	6	0.000606402	-0.000043275	-0.000091281
334	6	-0.000638989	0.000128492	0.000257942
335	6	0.001307750	0.000138517	0.000198748
336	1	0.000472743	0.000398814	0.000422116
337	1	-0.000606783	-0.000148664	-0.000519615
338	1	0.000064376	0.000118410	-0.000051071
339	1	-0.000312165	0.000038755	-0.000135516
340	1	-0.000386175	-0.000318518	-0.000189124
341	1	-0.000401023	0.000010795	0.000047289
342	8	0.000934830	-0.001143864	-0.001495868
343	8	0.000057953	0.001318713	-0.000865203
344	6	-0.000565388	0.000868235	0.000976177
345	6	0.000261151	-0.000306546	-0.000459387
346	6	-0.000742373	-0.000491916	0.001154486
347	6	0.000952190	0.000544479	-0.000199311
348	6	0.000115246	0.000332748	0.000228514
349	6	0.000694431	-0.000212256	-0.000226731
350	6	-0.000363466	-0.000309063	-0.000461682

351	6	0.001332153	0.000547654	0.000121746
352	1	-0.000066435	0.000197862	0.000020374
353	1	-0.000799871	-0.000470732	0.000050267
354	1	-0.000041541	-0.000278970	0.000008367
355	1	-0.000338195	-0.000029838	-0.000069627
356	1	-0.000433604	-0.000185618	-0.000137468
357	1	-0.000452473	-0.000206070	0.000151722
358	8	-0.000178037	0.001849476	0.001835988
359	8	-0.000561269	-0.000687253	0.000660806
360	6	0.000413730	-0.001543804	-0.001220565
361	6	-0.000283271	0.000749644	0.000401694
362	6	-0.000122504	0.000197283	-0.001164792
363	6	0.000817995	-0.000245926	0.000577352
364	6	0.000058411	-0.000451881	-0.000312986
365	6	0.000236973	0.000546974	0.000442518
366	6	-0.000550423	0.000015759	0.000102652
367	6	0.001194392	-0.000316000	0.000492575
368	1	0.000143279	-0.000345690	0.000126068
369	1	-0.000616141	0.000517901	-0.000389752
370	1	-0.000044930	0.000170412	0.000056165
371	1	-0.000333119	0.000138149	-0.000376467
372	1	-0.000320347	-0.000003244	-0.000087992
373	1	-0.000381257	0.000147115	-0.000076757
374	1	0.000044446	-0.000114557	0.000703642
375	1	-0.001776506	-0.000373380	0.000481196
376	1	0.000108086	-0.000487985	-0.000009542
377	1	-0.000795437	0.000540098	-0.001054453
378	1	0.000172977	-0.000350374	0.000825082
379	1	0.000273644	0.000349126	-0.000625353
380	1	-0.000569108	0.000477667	0.000003535
381	1	0.000781788	0.000516949	-0.000440617
382	1	-0.000989063	-0.000790933	-0.000546933
383	1	-0.000693606	0.002015748	-0.000899135
384	8	0.000115967	-0.000360068	-0.000842901
385	8	0.000920810	0.000518065	-0.000433254
386	6	-0.000273594	0.000330933	0.000359977
387	6	0.000757848	0.000033094	-0.000479371
388	6	-0.000057402	0.000167864	0.000016607
389	6	-0.000655123	-0.000152546	-0.000023561
390	6	-0.000160592	0.000094050	0.000170138
391	6	-0.000178893	-0.000008309	-0.000278645
392	6	0.000499399	-0.000141316	0.000373958
393	6	-0.001408899	-0.000199623	-0.000240283
394	1	-0.000431142	-0.000197280	0.000211906
395	1	0.000796243	0.000156411	-0.000265634
396	1	0.000038180	-0.000029279	-0.000094171
397	1	0.000392900	-0.000020469	0.000169163
398	1	0.000419397	-0.000040310	0.000001015
399	1	0.000436586	0.000249622	-0.000024056
400	8	-0.000408642	0.000101145	0.000588869
401	8	0.000409132	-0.000370826	0.000448307
402	6	0.000149658	-0.000121749	-0.000144196
403	6	0.000063858	-0.000036867	0.000237630
404	6	0.000022479	-0.000226909	0.000122045
405	6	-0.000564639	0.000168131	-0.000228585
406	6	-0.000057857	-0.000075834	-0.000142591
407	6	-0.000412728	0.000094440	-0.000003184
408	6	0.000618687	0.000145488	-0.000092601

409	6	-0.001366393	0.000275824	-0.000345249
410	1	0.000007793	0.000164761	-0.000169228
411	1	0.000670586	-0.000269699	0.000553431
412	1	-0.000017417	0.000085720	0.000076695
413	1	0.000411429	-0.000028136	-0.000002064
414	1	0.000373600	0.000020523	0.000151650
415	1	0.000366954	-0.000305636	0.000206185
416	8	0.000483125	0.000079528	-0.000554376
417	8	0.000433194	-0.000214139	0.000284530
418	6	0.000503278	0.000015945	0.000304651
419	6	0.000092932	0.000360872	-0.000236234
420	6	0.000372224	0.000376719	0.000503651
421	6	-0.000719410	-0.000299115	-0.000206943
422	6	-0.000107360	-0.000193663	-0.000042602
423	6	-0.000680300	-0.000084853	-0.000353030
424	6	0.000580167	0.000061151	-0.000205388
425	6	-0.001363829	-0.000213963	-0.000318054
426	1	-0.000284015	-0.000394807	-0.000163140
427	1	0.000665512	0.000489008	0.000304640
428	1	0.000031917	0.000151314	-0.000060619
429	1	0.000447619	0.000098654	-0.000003745
430	1	0.000360954	0.000179302	0.000315448
431	1	0.000364041	-0.000049528	0.000043192
432	8	-0.000748814	-0.000140379	0.000377082
433	8	0.000575882	0.000966797	0.000662829
434	6	0.000365473	-0.000366726	-0.000126935
435	6	0.000495396	0.000491786	0.000300661
436	6	0.000149868	-0.000565216	-0.000927235
437	6	-0.000587369	0.000448420	0.000125088
438	6	-0.000117538	0.000189640	-0.000000791
439	6	-0.000677406	0.000133356	0.000019719
440	6	0.000390497	-0.000138862	0.000393320
441	6	-0.001333291	0.000430025	-0.000201152
442	1	-0.000289681	0.000019462	0.000012995
443	1	0.000717078	-0.000474608	-0.000006006
444	1	-0.000012245	-0.000223404	0.000015632
445	1	0.000449294	-0.000196368	-0.000146568
446	1	0.000374360	0.000008217	0.000120493
447	1	0.000421257	-0.000181142	0.000150830
448	1	0.000732596	0.000333896	-0.000343369
449	1	-0.000427632	-0.000413183	0.000609874
450	1	-0.000334311	-0.000452632	-0.000078777
451	1	-0.000163128	-0.000059302	-0.000071894
452	1	-0.001091382	-0.000650267	-0.000706058
453	1	-0.001593037	-0.002506738	0.000693277
454	1	-0.001013217	0.000373598	0.000541757
455	1	-0.000033941	-0.000085265	0.000039533
456	1	0.000462048	0.000114854	-0.000466622
457	1	0.000130420	0.000262572	0.000354229
458	6	0.001625993	0.002939548	-0.001602699
459	6	-0.000043920	0.000445340	-0.000123925
460	6	0.000069492	-0.000013256	-0.000844994
461	6	-0.000616322	-0.004982121	0.002855815
462	6	-0.000002782	-0.000124020	0.000176562
463	6	0.000428898	0.000614384	-0.000329028
464	1	-0.000643574	0.000974128	-0.006352513
465	1	-0.002069793	-0.005973721	-0.002653222
466	1	-0.000399184	-0.001100600	0.006886659

467	1	0.001370989	0.005611315	0.003381649
468	17	0.000635051	0.002200556	0.001026258
469	1	-0.000111974	0.000189749	0.000095297
470	1	-0.000451780	0.000701858	-0.000116921
471	1	-0.000512524	-0.000180583	0.000170930
472	8	-0.001082579	0.001704961	-0.001325611
473	1	0.000554136	-0.000954452	0.000349612
474	1	0.000278826	-0.000328820	0.000790848
475	8	0.000921250	0.000917789	0.000496285
476	1	-0.000257484	-0.000737961	0.000062379
477	1	0.000028313	-0.000380994	-0.000145589
478	8	-0.002031658	-0.001081534	0.000995699
479	1	0.000754696	0.000688591	-0.000286353
480	1	0.001034032	0.000030081	-0.000941486
481	6	-0.004982873	0.001479855	0.000508470
482	8	0.000985162	-0.001258602	-0.002334008
483	7	-0.001281743	-0.001606232	0.001812168
484	8	-0.000610294	-0.001417755	-0.002899965
485	8	0.001004650	0.001763865	0.002100716

Sum of electronic and thermal Free Energies= -1488.923904

1 imaginary frequency: -388.02

6. References

[1]Tunstad, L. M.; Tucker, J. A.; Dalcanale, E.; Weiser, J.; Bryant, J. A.; Sherman, J. C.; Helgeson, R. C.; Knobler, C. B.; Cram, D. J. Host-Guest Complexation. 48. Octol Building Blocks for Cavitands and Carcerands *J. Org. Chem.* **1989**, *54*, 1305-1312.

[2]La Manna, P.; Talotta, C.; Floresta, G.; De Rosa, M.; Soriente, A.; Rescifina, A.; Gaeta, C.; Neri, P. Mild Friedel–Crafts Reactions inside a Hexameric Resorcinarene Capsule: C−Cl Bond Activation through Hydrogen Bonding to Bridging Water Molecules. *Angew. Chem. Int. Ed.* **2018**, *57* (19), 5423–5428. https://doi.org/10.1002/anie.201801642.

[3]Taylor, J. E.; Jones, M. D.; Williams, J. M. J.; Bull, S. D. Friedel−Crafts Acylation of Pyrroles and Indoles Using 1,5-Diazabicyclo[4.3.0]Non-5-Ene (DBN) as a Nucleophilic Catalyst. *Org. Lett.* **2010**, *12* (24), 5740–5743. https://doi.org/10.1021/ol1025348.

[4]Shi, X.; Chen, X.; Wang, M.; Zhang, X.; Fan, X. Regioselective Synthesis of Acylated *N* -Heterocycles via the Cascade Reactions of Saturated Cyclic Amines with 2-Oxo-2-Arylacetic Acids. *J. Org. Chem.* **2018**, *83* (12), 6524–6533. https://doi.org/10.1021/acs.joc.8b00805.

[5]Tjutrins, J.; Arndtsen, B. A. An Electrophilic Approach to the Palladium-Catalyzed Carbonylative C–H Functionalization of Heterocycles. *J. Am. Chem. Soc.* **2015**, *137* (37), 12050–12054. https://doi.org/10.1021/jacs.5b07098.

[6]Liu, Y.; Kaiser, A. M.; Arndtsen, B. A. Palladium Catalyzed Carbonylative Generation of Potent, Pyridine-Based Acylating Electrophiles for the Functionalization of Arenes to Ketones. *Chem. Sci.* **2020**, *11* (32), 8610–8616. https://doi.org/10.1039/D0SC03129A

[7]Gaussian 16, Revision C.01, Frisch, M. J.; Trucks, G. W.; Schlegel, H. B.; Scuseria, G. E.; Robb, M. A.; Cheeseman, J. R.; Scalmani, G.; Barone, V.; Petersson, G. A.; Nakatsuji, H.; Li, X.; Caricato, M.; Marenich, A. V.; Bloino, J.; Janesko, B. G.; Gomperts, R.; Mennucci, B.; Hratchian, H. P.; Ortiz, J. V.; Izmaylov, A. F.; Sonnenberg, J. L.; Williams-Young, D.; Ding, F.; Lipparini, F.; Egidi, F.; Goings, J.; Peng, B.; Petrone, A.; Henderson, T.; Ranasinghe, D.; Zakrzewski, V. G.; Gao, J.; Rega, N.; Zheng, G.; Liang, W.; Hada, M.; Ehara, M.; Toyota, K.; Fukuda, R.; Hasegawa, J.; Ishida, M.; Nakajima, T.; Honda, Y.; Kitao, O.; Nakai, H.; Vreven, T.; Throssell, K.; Montgomery, J. A., Jr.; Peralta, J. E.; Ogliaro, F.; Bearpark, M. J.; Heyd, J. J.; Brothers, E. N.; Kudin, K. N.; Staroverov, V. N.; Keith, T. A.; Kobayashi, R.; Normand, J.; Raghavachari, K.; Rendell, A. P.; Burant, J. C.; Iyengar, S. S.; Tomasi, J.; Cossi, M.; Millam, J. M.; Klene, M.; Adamo, C.; Cammi, R.; Ochterski, J. W.; Martin, R. L.; Morokuma, K.; Farkas, O.; Foresman, J. B.; Fox, D. J. Gaussian, Inc., Wallingford CT, 2016.

[8]Ruff, F.; Farkas, O. Concerted SN2 mechanism for the hydrolysis of acid chlorides: comparisons of reactivities calculated by the density functional theory with experimental data. *J. Phys. Org. Chem*. **2011**, 24 480–491. https://doi.org/10.1002/poc.1790.

[9]Bentley, T.W.; Llewellyn, G.; McAlister, J.A. SN2 Mechanism for Alcoholysis, Aminolysis, and Hydrolysis of Acetyl Chloride. *J. Org. Chem*. **1996**, 61, 7927-7932. https://doi.org/10.1021/jo9609844.
